# Bisphosphine ligand conformer selection to enhance descriptor database representation: improving statistical modelling outcomes

**DOI:** 10.1039/d5sc04691b

**Published:** 2025-09-29

**Authors:** Jamie A. Cadge, Sierra D. Hart, Richard C. Walroth, Kyle A. Mack, Matthew S. Sigman

**Affiliations:** a Department of Chemistry, University of Utah Salt Lake City Utah 84112 USA sigman@chem.utah.edu; b Department of Chemistry, University of Bath Bath BA2 7AY UK; c Department of Small Molecule Process Chemistry, Genentech Inc. San Francisco California 94080 USA

## Abstract

A foundational consideration in the development of computationally derived molecular feature libraries is the generation and selection of conformers. It has been shown that several feature values have a degree of conformer depencency – which may have significant mechanistic implications, partiticulary in the field of homogeneous enantioselective catalysis. However, the computational cost of calculating conformers often prohibits this analysis from being performed, especially when large flexible systems are involved. We report here a practical, chemically-intutive conformer selection tool for bisphosphine-ligated palladium(ii) dichloride complexes that provide a good balance between representation and computational cost. Conformer-weighted features generated from this method were applied to two previous statistical modelling case studies, where weighted features improve model quality with respect to predictive power. This selection methodology has the potential to be applied to a range of complex molecular systems beyond bisphosphine-ligated organometallic complexes.

## Abstract 2

Computational workflows for the generation of conformationally-weighted bisphosphine ligand descriptors are examined. Selection of relevant conformers allows for ensemble representation and improved model metrics while balancing computational costs.

## Graphical abstract

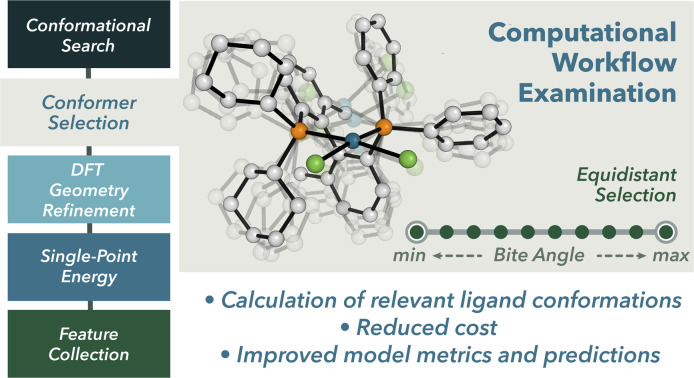

## Introduction

**Fig. 1:**
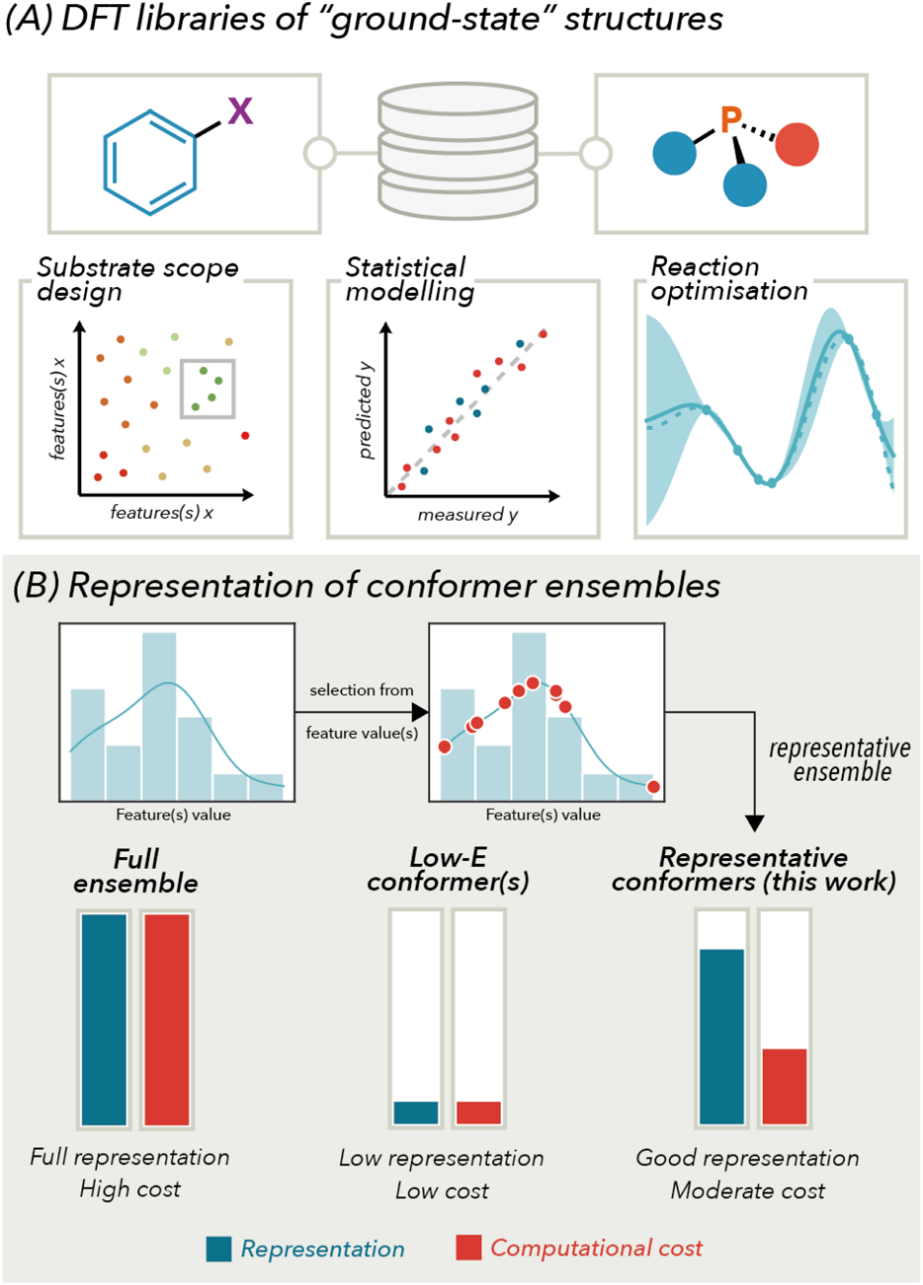
Introduction.

The use of diverse computationally derived molecular descriptor libraries has been crucial for interfacing data science tools with organic reaction development.^1^ Recent examples of such libraries from our group and others include organic compounds such as aryl halides,^2^ carboxylic acids and amines^3^ as well as monodentate^4^ and bidentate^5^ ligands in organometallic chemistry. The extracted molecular features can be used in downstream machine learning (ML) and statistical modelling efforts. These applications can include the deconvolution of reaction mechanisms, definition of structure function relationships, and prediction of optimal reaction conditions.^6^ These libraries often consist of “ground-state” computed structures from which a series of molecular features can be extracted (Fig. 1A). A key assumption in these efforts is that ground-state features can be used to explain trends in reactivity or selectivity akin to the use of Hammett plots and other linear free-energy relationships in physical organic chemistry.^7^

A further consideration when constructing feature libraries is the inclusion of conformationally derived descriptors as both steric and electronic properties can be sensitive to dynamics and conformation. For example, Sterimol^8^ (a steric measurement of the maximum and minimum lengths perpendicular to a pre-defined axis)^9^ and *V*_min_ (an electronic feature commonly used to describe the σ-donating ability of monodentate phosphine ligands)^10^ can vary depending on the conformation a molecule adopts. Reaction energy barriers can have a range of up to 10 kcal mol^−1^, as a result of structure conformation, and it is challenging to know *a priori* which conformer(s) are required to explain reactivity, *i.e.*, in a transition state or intermediate involved in the turnover-limiting or selectivity-determining step.^11^ This phenomenon is of particular significance in the field of homogeneous catalysis.

In this context, incorporation of conformational information into a descriptor library building campaign is dependent on two key factors: ease of automation and computational costs. The former has been enabled by packages such as AutoQChem,^12^ AQME^13^ and molli^14^ from the Doyle, Paton and Denmark labs, respectively, as well as a recent workflow developed by our group.^3^ These methods often utilize density functional theory (DFT) to facilitate accurate featurization. However, as molecules increase in size and complexity (*e.g.*, organometallic species), DFT incurs a significant computational cost that can be limiting on scale.^15^ This becomes especially challenging when incorporating conformational ensembles. Our previous approach to featurized bisphosphine ligands used a force-field approach followed by ensemble pruning to five conformers based on RMSD atomic positions. From these five conformers, the lowest energy conformer defined through DFT was used for downstream featurization and modelling efforts.^5*e*^ While this feature library has been successful in various optimisation and experimental design campaigns,^2*c*,5*e*^ we hypothesised this approach may be inadequate for the representation of the energetically accessible ligand conformer landscape. Additionally, this may limit the predictive ability and/or the domain of applicability of modelling tasks (Fig. 1B).

When considering the design of a DFT feature library for systems of high molecular complexity and conformational flexibility, there is clearly a need for effective conformer selection. Herein, we report the development of a feature-based approach for effectively sampling the bisphosphine ligand conformational landscape. Specifically, a privileged geometric feature, bite angle, was used to select conformers derived from a library of bisphosphine-ligated palladium(ii) dichloride complexes. This strategy functions as an effective compromise between ensemble representation and computational cost. To evaluate this approach, two previous challenging statistical modelling campaigns were examined, where inclusion of conformer-derived features was found to improve predictive power. We anticipate that with the appropriate selection of a feature or features, they could be used for the sampling of other systems of high complexity.

## Selection and calculation of complexes

Several strategies towards conformer selection have been reported, including tools such as CREGEN from Grimme *et al.*,^16^ COSMOconf from Klamt and co-workers,^17^ the ReSCoSS workflow (Udvarhelyi, Rodde and Wilcken)^18^ and CONFPASS from Goodman and co-workers.^19^ Many of these methods rely on sampling conformers by energy, RMSD atom deviations or changes in dihedral angles. For complex organometallic structures, this can result in impractically large ensembles depending on computational resources. To further down select and lower the computational cost, we hypothesized that conformer selection should be informed by a chemically intuitive feature(s). We chose to examine the accuracy trade-off between the computational approaches used, and thereby the cost incurred for calculation of conformation ensembles (*vide infra*).

**Fig. 2:**
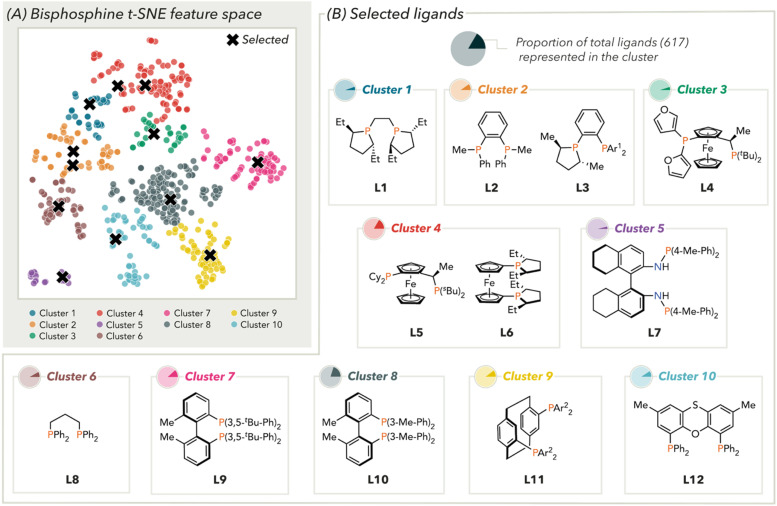
(A) Selection of representative ligand using clustered chemical space and (B) selected ligands by cluster. Ar^1^ = 3,5-^*t*^Bu-4-OMe-Ph; Ar^2^ = 4-(CF-di-CF_3_)-Ph.

To accomplish this, 12 palladium(ii) dichloride complexes were selected from our group's previously computed bisphosphine descriptor library,^5*e*^ to represent scaffold variance across chemical space. This was visualized using *t*-distributed stochastic neighbour embedding (*t*-SNE) and hierarchal clustering (*n* = 10) (Fig. 2A).^20^ This feature space was constructed with predominantly free ligand descriptors as this was presumed to be more generally representative of ligand diversity compared to metal complex descriptors. Pleasingly, these dimensionality reduction and clustering techniques intuitively categorized ligand backbones together and informed the subsequent selection of 12 bisphosphine ligands for calculation.^21^Fig. 2B shows the diversity of ligands used in the following analyses, including ligands with alkyl backbones (L1, L8), aromatic backbones (L2, L3, L7 and L9–L12), as well as those based on ferrocene (L4–L6).

**Fig. 3:**
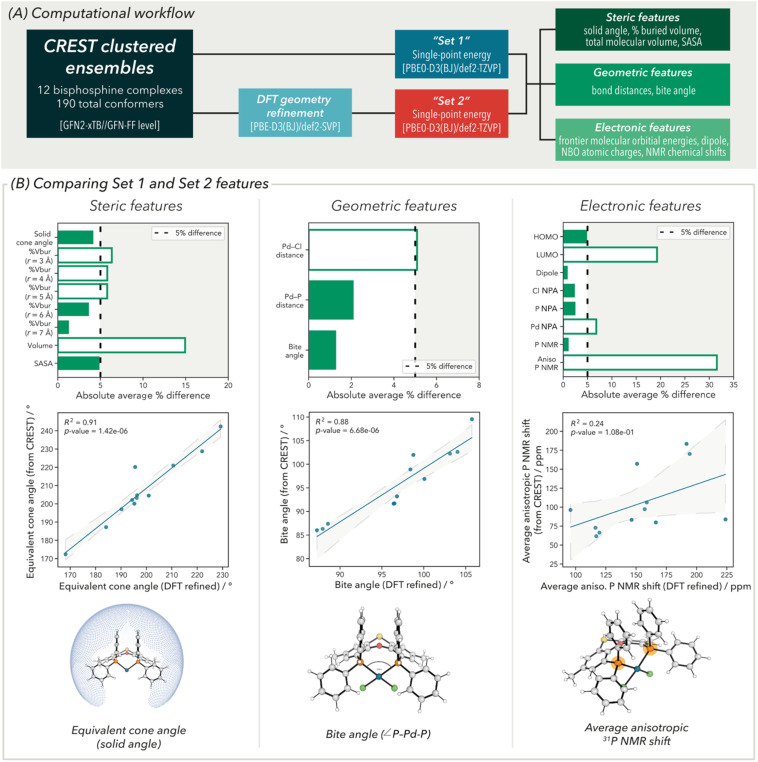
(A) Computational methods for generating “Set 1” and “Set 2” structures and bisphosphine ligand features collected and (B) comparison of “Set 1” and “Set 2” features: (top) comparison of absolute average % differences in feature values; (middle) linear regression models comparing DFT-refined and non-DFT-refined structure feature values and (bottom) description of the three features used in the linear regression models. For a description of all other features used here, see the SI.

Using the initial library of DFT computed structures as starting coordinates, conformer ensembles for these ligands were generated using the open-source CREST program (where conformers are differentiated using rotational constants).^16^ A 5 kcal mol^−1^ energy window using the GFN2-xTB//GFN-FF composite method (Fig. 3A) was applied to each conformer search.^22^ This method was chosen as structure optimization at the GFN-FF level has been shown to produce geometries with high accuracy when compared to other force field approaches.^23^ Structurally redundant conformers were removed using a PCA/*k*-means clustering technique of relevant dihedral angles (as implemented in CREST).^24^

We then probed whether the geometries produced using this workflow were sufficiently accurate for generating ligand features, which would remove the costly DFT geometry refinement step. To evaluate this, subsequent DFT single point correction (SPC) calculations, required for property collection, were performed on the CREST geometries at the PBE(0)-D3(BJ)/def2-TZVP level of theory. The Perdew–Burke–Ernzerhof (PBE) functionals^25^ were selected as they have been previously used in our library building campaigns^4*b*,5*e*^ and demonstrated to effectively predict ^31^P NMR chemical shifts.^26^ These calculations form “Set 1”. These were then compared to calculations defined as “Set 2”, in which the CREST generated structures were calculated at the PBE0-D3(BJ)/def2-TZVP//PBE-D3(BJ)/def2-SVP level of theory prior to featurization.

For each Set, a representative series of steric, geometric, and electronic descriptors were collected for comparison.^27^ In the case of steric features, ligand equivalent cone angle (derived from a solid angle calculation), percentage buried volume (%*V*_bur_) at Pd (with radii, *r*, between 2 Å and 7 Å, in increments of 1 Å), total molecular volume, and the solvent accessible surface area (SASA) at Pd were selected. For geometric descriptors, Pd–Cl and Pd–P bond lengths, and bite angle (∠P–Pd–P) were selected. Frontier molecular Kohn–Sham orbital (HOMO and LUMO) energies and molecular dipole were chosen as examples of global electronic descriptors. Atom-specific properties included atomic partial charges (on Pd, P and Cl atoms) derived from a natural population analysis (NPA) calculation and isotropic and anisotropic ^31^P NMR chemical shifts. In instances where there can be more than one possible value for a descriptor (*e.g.*, Pd–P bond length or P NPA charge), feature values were averaged.

This comparison provides information on whether the time-intensive geometry refinement step of this computational workflow could be circumvented, which would allow for more conformations to be computed.

### Effect of DFT geometry refinement on GFN-FF optimized structures

To compare the quality of the features from calculation Set 1 and Set 2, the average (over the 12 ligands) percentage difference between the descriptors were initially evaluated. Fig. 3B (top) shows the comparison of lowest energy DFT-PBE0 feature values. Similar analyses for Boltzmann-weighted average descriptors (298 K) as well as the maximum and minimum values are given in the SI.

For steric and geometric features, there is generally good agreement between Set 1 and Set 2, where average differences are approximately 5% or less. In contrast, there is less agreement between feature values from Set 1 and Set 2 for electronic descriptors. For example, LUMO energy and anisotropic ^31^P NMR chemical shift show significant differences in their feature values (>15%). This indicates that both the global and atom-level electronic structure are poorly described in structures that did not undergo DFT geometry refinement.

While the absolute electronic feature values between the two calculation Sets are different, their relative values may have better agreement. To test this, the collinearity between the two Sets was considered (Fig. 3B, middle). However, in all cases, poor correlations (*R*^2^ < 0.5) were observed between feature values in Set 1 and Set 2. Notably, the anisotropic ^31^P NMR chemical shift gave an *R*^2^ value of 0.24. Similar results were obtained with HOMO energy, LUMO energy and isotropic ^31^P NMR chemical shift (giving *R*^2^ = 0.64, 0.56, and 0.46, respectively). We suspect these differences result from a mismatch in the electronic structure theory methods used (*i.e.*, different optimized local minima between semi-empirical *vs.* DFT and/or inaccurate bond lengths/angles with semi-empirical methods) to determine the complex geometries. Resultant minor structural changes between Set 1 and Set 2 are enough to cause divergences in the electronic structure of the metal complex and, therefore, electronic features obtained. Unsurprisingly, correlations observed with steric and geometric features were significantly better. These show a good linear relationship between descriptors in Set 1 and Set 2 (*R*^2^ > 0.8, see SI for full set).

Given these significant differences observed between electronic descriptors obtained from DFT-level geometry refinement and geometries obtained from CREST, structures from the latter (Set 1) were deemed inappropriate, even in relative terms. Additionally, the accuracy of electronic features is crucial as these have been demonstrated to be key in previous modelling efforts for both mono-^28^ and bisphosphine ligands.^5*d*,*e*^ However, due to the size and complexity of the bisphosphine complexes used in this study, as mentioned above, DFT-level geometry refinement on all complexes generated from a conformer search would incur significant computational cost if this approach was extended to the entire bisphosphine ligand library of >600 ligands. As the steric and geometric features obtained from CREST were in good agreement with those derived from a subsequent DFT refinement, we considered whether ensembles could be pruned based on one (or a combination of) steric and geometric features. Notably, a similar approach was taken in the *kraken* monophosphine ligand library, where representative conformers were selected based on a number of steric descriptors.^4*b*^

### Selection of conformers using geometric and steric features

In choosing features to use in the selection of conformers for calculation at the DFT level, we took inspiration from previous descriptors used for bisphosphine ligands in the literature. For example, bite angle has been an informative descriptor of bisphosphine ligands inspiring the development of many transition metal-catalysed processes such as hydroformylation.^29^ Additionally, this geometric descriptor encompasses both an electronic and steric effect. With increasing bite angle, the symmetry and energy of the coordinated metal's frontier orbitals change leading to a subsequent destabilization of a metal complex.^30^ This destabilization, in several cases, enhances reactivity such as a higher propensity to undergo oxidative addition. Bite angle also impacts the steric profile of the metal centre that substrates can access during catalysis (akin to a binding pocket).^31^ Narrow bite angles can be used to alleviate steric interactions which, for example, can lead to increased transition state stability. Other descriptors often used are the equivalent cone angle and %*V*_bur_. The equivalent cone angle was originally developed by Weigand and co-workers and serves as a useful analogue to Tolman's cone angle at bisphosphine-ligated metal complexes.^32^ %*V*_bur_ pioneered by Nolan and Cavallo *et al.* has also been used to effectively describe the steric environment of both mono- and bisphosphine ligands.^33^

**Fig. 4:**
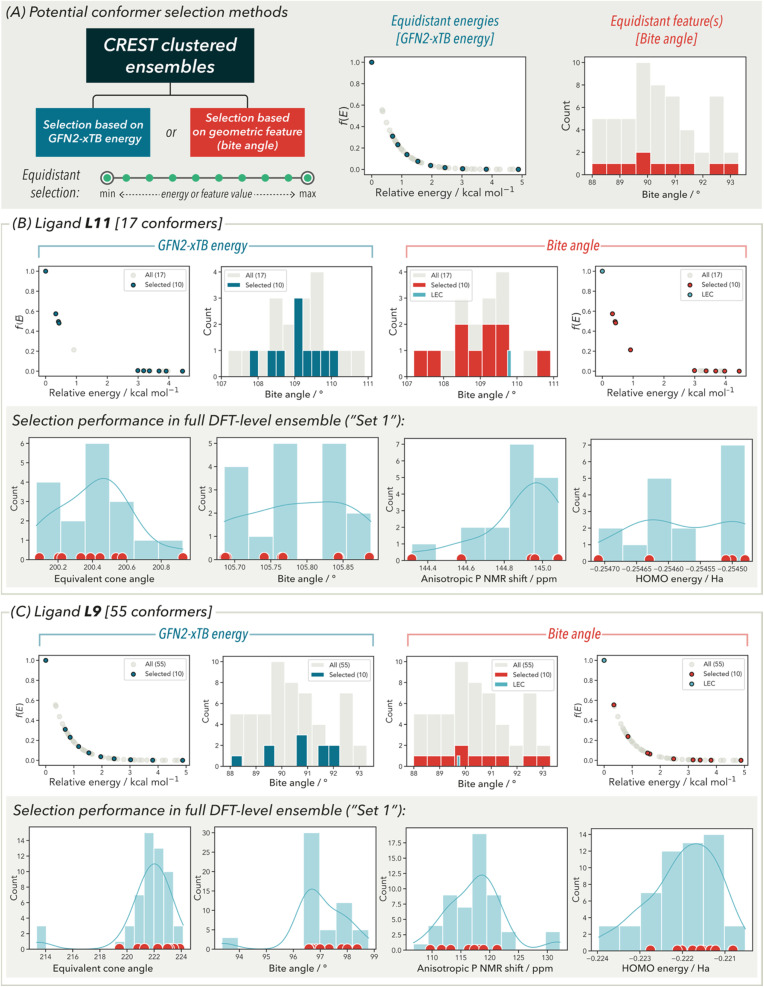
(A) Depiction of possible conformer selection methods investigated here and analysis of both methods for two ligands: (B) L11 with a total of 17 conformers with (top) selection based on GFN2-xTB energy and bite angle and (bottom) performance in the full calculated DFT ensemble. (C) Shows equivalent analysis with L9 with a total of 55 conformers.

Before examining steric and geometric features for choosing conformers, selection based on the GFN2-xTB energy was first investigated. This would provide a good comparison with selection based on steric and geometric features for all 12 bisphosphine palladium(ii) dichloride complexes (Fig. 4A). Conformers were selected based on their equidistant GFN2-xTB energy values, *i.e.*, taking the minimum and maximum values as well as evenly distributed points in between. For practical purposes, up to ten conformers in each selection was used to acquire the maximum conformer diversity at a reasonable computational cost. The use of five conformers was also investigated but gave inadequate representation – especially with larger conformer ensembles.

Selections for complexes of ligand L11 and L9 are depicted in Fig. 4B and C, respectively as illustrative examples, with the average and largest conformer ensemble sizes, respectively (for analysis of the remaining ten ligands, see the SI). Selection based on GFN2-xTB energies gives a structurally limited ensemble. For example, the distribution of bite angles obtained is limited compared to the entire ensemble *i.e.*, the range of the pruned ensemble is less than that of the full ensemble (4° *vs.* 6° for L9). Similar observations are made with ligand %*V*_bur_ values. To obtain better structural diversity, a similar selection methodology was applied using bite angle. In addition to increased structural diversity in the new conformer ensemble, this approach also resulted in a good distribution of GFN2-xTB energies across the full 5 kcal mol^−1^ window. It was determined that the lowest GFN2-xTB energy conformer would also be included (if not one of those selected) to further increase the conformer energy span.^34^

As this strategy provided a good coverage of the conformer ensemble in terms of energy and structural diversity, we then examined the effects of this treatment in the full DFT-generated conformer ensembles in calculation Set 2 (Fig. 4B and C, bottom). Given the diversity of ligands used in this study, this method provided a simulation if this selection method would be applied to the entire bisphosphine ligand library. DFT-level feature distributions for the Set 2 ensembles of ligands L11 and L9 are again analysed. Equivalent cone angle, bite angle, anisotropic ^31^P NMR chemical shift and the HOMO energy were chosen as representative features (see SI for analysis with all features). Dots highlighted on the distributions in Fig. 4B and C (bottom) show the feature values of bite angle-selected conformers to directly compare with the full DFT conformer ensemble. In the case of ligand L11 with an average size ensemble, there is an adequate distribution of selected conformers for all four features indicated by their ranges being nearly identical. However, for the larger ensemble of ligand L9, there is reduced coverage of the DFT ensemble by the selected conformers where some of the extrema of feature values are missed upon selection. For example, smaller values of solid cone angle and bite angle are not included in the selection method. Nevertheless, maintaining a practical view on what calculations are performed in the context of a large ligand library building campaign, we view this as a reasonable compromise between conformer feature representation and computational cost. For the ligands examined in this workflow preparation, a comparison was made between DFT optimization times required for the full ensemble of ligands and the representative selection of ligands. By reducing the conformational ensemble size to 11 or fewer ligands, the total time required for DFT geometry refinement was reduced by 70.1%.

### Application of selection method in bisphosphine modelling campaigns

With a conformer selection methodology established, we generated new ligand features analogous to those from our previously published bisphosphine library which now include conformer-weighting. Conformer-weighted features include lowest energy conformer value, minimum and maximum feature values as well as Boltzmann-weighted averaged features (at 298 K) and conformer arithmetic mean values giving a total of 2088 features. This set was reduced to features that were conformationally dependent (*i.e.*, those showing variance in feature value across an ensemble). For features that showed low variance across an ensemble, only the Boltzmann-weighted average value was retained, reducing the feature set to less than 1300 descriptors.^35^ To test the performance of the new ligand features, they were applied in two bisphosphine modelling case studies previously reported by our group. Both of these examples represent small dataset sizes where model building can be a challenge but is often the reality the chemical sciences.^36^

### Hayashi–Heck cross-coupling regioselectivity

**Fig. 5:**
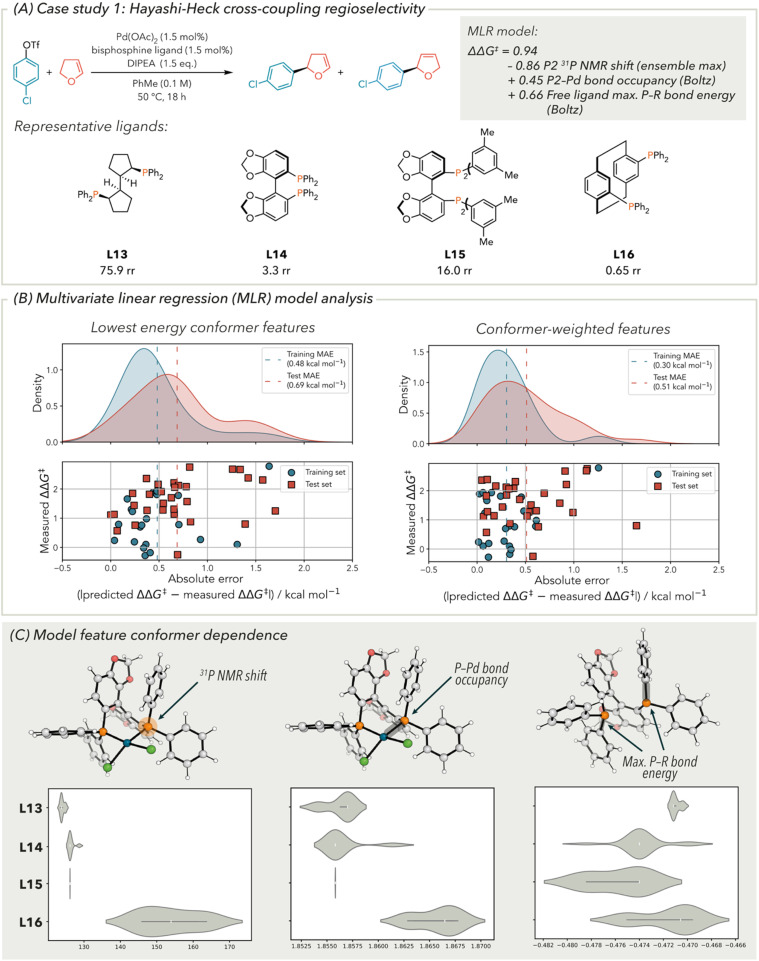
(A) MLR model for Hayashi–Heck cross-coupling regioselectivity including examples of representative ligands, (B) analysis of model performance with and without conformer-weighted features represented as distributions of individual ligand absolute prediction error and (C) analysis of feature conformer dependence. For a full definition of model features see the SI.

The first case study for feature comparison concerns a palladium-catalysed Hayashi–Heck cross-coupling reaction (Fig. 5A).^5*e*^ Here, the arylation regioselectivity (ΔΔ*G*^‡^) was modelled using multivariate linear regression (MLR). However, prediction of validation ligands led to mixed results with some examples giving a mean absolute error (MAE) of up to 1.8 kcal mol^−1^. The reason we previously presented for these types of poor predictions was due to the structures being outside the domain of model applicability. Therefore, we hypothesised that our new conformer-weighted ligand features could enhance the predictive ability of validation ligands. With the full set of ensemble features (a combination of conformer-weighted and lowest energy conformer features), a three step forward stepwise model search was conducted using 22 ligands to train models and tested on 30 ligands, which were defined in the previous study.^5*e*^ This gave a model where ΔΔ*G*^‡^ is described by the conformer maximum ^31^P NMR chemical shift and the Boltzmann-weighted average of the Pd–P σ-bonding occupancy (from a NPA calculation). Interestingly, both of these terms are derived from the phosphorus atom with the smallest %*V*_bur_ (*r* = 3.0 Å). Additionally, the P–R σ-bonding energy from the free ligand is also present in the model equation. Together these features indicated a strong electronic influence of the ligand on the observed regioselectivity similar to the previously reported statistical model. Moreover, the steric dependence incorporated into the workflow to define these features may infer that experimental regioselectivity is based on binding or donating ability of the smaller of the two phosphorus donors. To assess the overall statistical model quality, we used the *R*^2^, mean absolute error (MAE) and root mean squared error (RMSE) of both the training and test set ligands. Overall, the generated three-term MLR model gave adequate training (*R*^2^ = 0.76, MAE = 0.30 kcal mol^−1^, RMSE = 0.41 kcal mol^−1^) and validation statistics (*R*^2^ = 0.57, MAE = 0.51 kcal mol^−1^, RMSE = 0.64 kcal mol^−1^). The prediction error (the absolute difference between predicted and measured ΔΔ*G*^‡^) of the training and test split is also described in Fig. 5B, left. Ligands in the training and test sets are represented as a distribution (from a kernel density estimation) to allow ready visualization of the error of individual datapoints. Unsurprisingly, this analysis revealed a wider distribution of the test set of ligands compared to those in the training set.

To investigate the effect of conformer-weighting on model performance, a model was generated using the analogous lowest energy features. Compared to the model containing the conformer-weighted features, the statistics of the training set were somewhat preserved when using the lowest energy conformer features (*R*^2^ = 0.46, MAE = 0.48 kcal mol^−1^, RMSE = 0.61 kcal mol^−1^). However, when evaluating the validation set the model statistics are significantly poorer (*R*^2^ = 0.29, MAE = 0.69 kcal mol^−1^, RMSE = 0.82 kcal mol^−1^). This can also be seen in the prediction error distribution (Fig. 5B, right). Compared to the error distribution of the model generated from conformer-weighted features, there are a greater number of datapoints that have a higher prediction error.

From the overall model statistics and examining the individual prediction errors of ligands, there is improvement when including conformer-weighting into bisphosphine ligand features. To understand the origin of the conformational impact of the features on model performance, violin plots of the feature values were constructed (Fig. 5C). Four representative ligands are depicted with the highest and lowest ΔΔ*G*^‡^ as well as two mid-range ΔΔ*G*^‡^ values. For electronic features, one would expect a lower conformational dependency compared to steric or geometric features, which is consistent with the observed distributions. Nevertheless, there are modest distributions of feature values across the ensembles, in keeping with observations in Fig. 4B and C. In the case of the ^31^P NMR chemical shift, there is a small distribution of feature values for the most selective ligand (L13), where the least selective ligand has a range of chemical shift of ∼30 ppm. Similar trends are apparent with the P σ-bonding occupancy and P–R σ-bonding energy features. The conformational dependence of these features provided further evidence that incorporating dynamic information improves the MLR model performance.

### Sulfonimidamide aryl carbonylation enantioselectivity

**Fig. 6:**
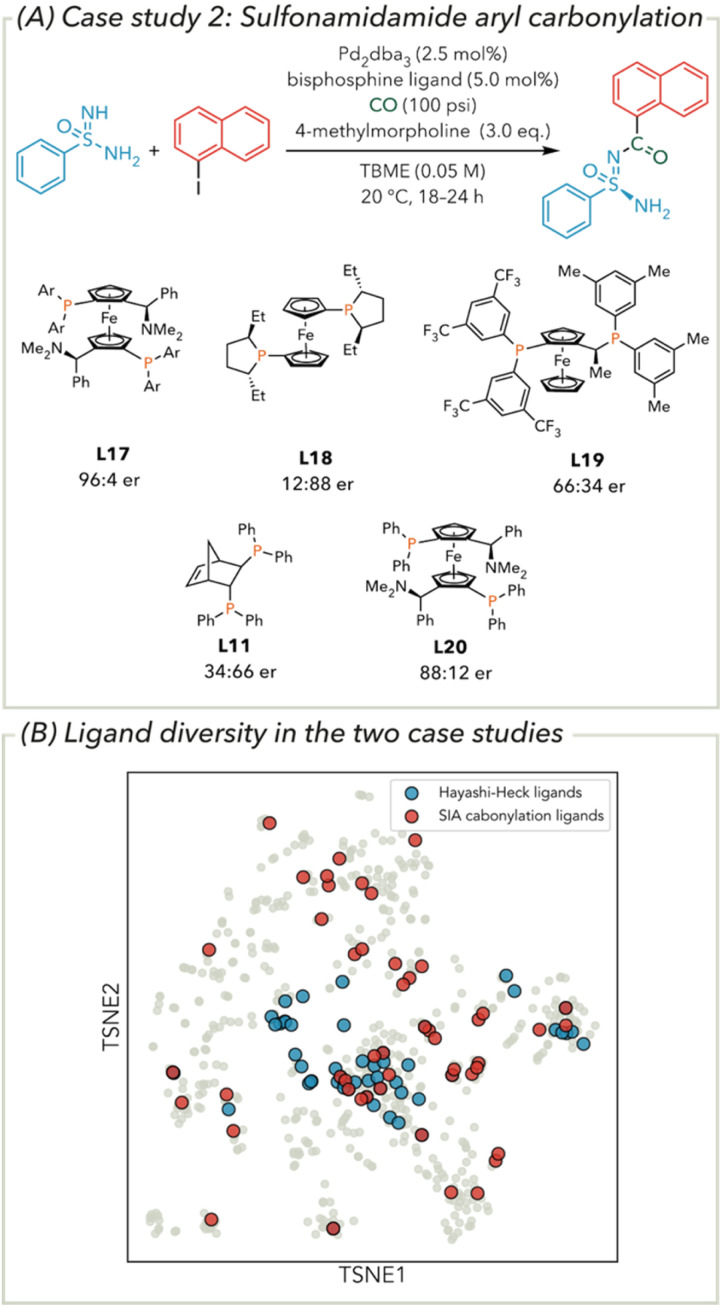
(A) Sulfonamidamide aryl carbonylation reaction used for modelling case study 2 with representative ligands show. (B) A comparison of the ligand diversity in the Hayashi–Heck and sulfonamidamide carbonylation datasets using the *t*-SNE chemical space from Fig. 2. Ar = 3,5-bis(trifluoromethyl)phenyl.

Following the increase in predictivity of an MLR model by including conformer-weighted features in the Hayashi–Heck cross-coupling case study, we turned our attention to a more complex statistical modelling challenge. In this second case study, a data-driven ligand selection was used to design a training set for evaluation against an enantioselective aryl carbonylation of sulfonimidamides (Fig. 6A). From this initial screen, a MandyPhos-type ligand L17 was identified to produce 100% conversion and excellent enantioselectivity (96 : 4 er). However, a linear statistical model to explain the resultant enantioselectivity (and perhaps prediction of a better performer) was not found. *In lieu* of this, a second round of ligands was also evaluated using similarity to the best performing ligand as determined through chemical space analysis. A considerably more diverse set of bisphosphine ligands were selected for this reaction compared to the previous Hayashi–Heck example, the comparison of the two case study datasets represented in *t*-SNE chemical space (introduced in Fig. 2) is shown in Fig. 6B. Perhaps as a result of the increased complexity of this dataset, a linear statistical model to explain the resultant enantioselectivity was not found. A total of 53 ligands were evaluated for the reaction, and still with this larger dataset, no linear statistical models were found, which we assumed was a result of the increased complexity of the dataset. This provided us with an opportunity to directly compare how the newly developed conformer-weighted features would perform on a challenging dataset.

**Fig. 7:**
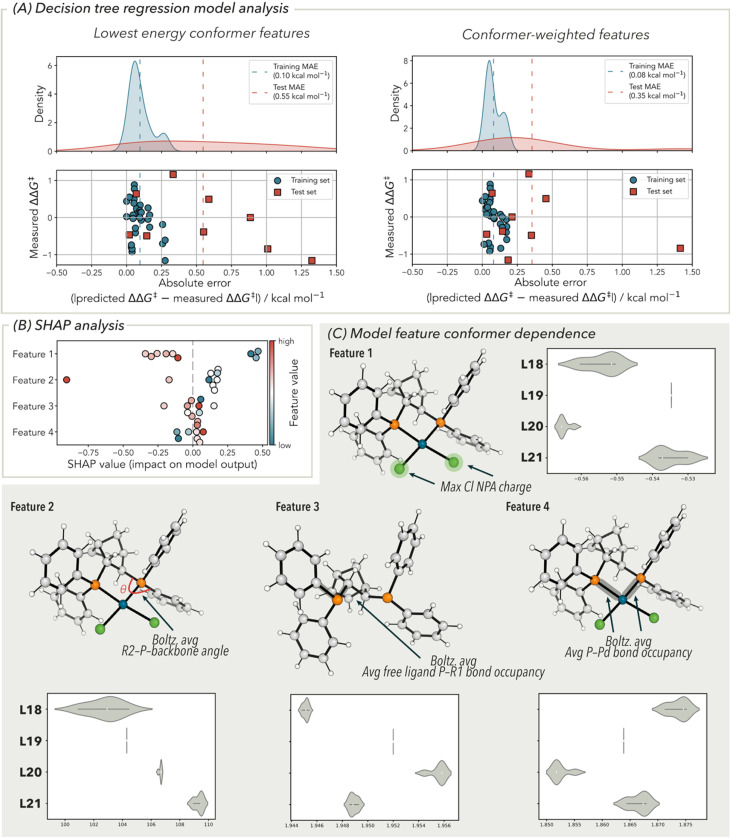
(A) Analysis of the decision tree regression model used for the sulfonamidamide carbonylation dataset. (B) SHAP analysis highlighting the relative importance of each feature. (C) Description of the features used in the decision tree regression model and analysis of their conformer dependence.

Recent analysis of the bisphosphine ligands in the sulfonamidamide carbonylation dataset showed that eight ligands originally used had oxidised (the majority giving low enantioselectivity) and these were removed from our analysis (see SI). Using the enantioselectivity data for the remaining 42 bisphosphine ligands with 4-methylmorpholine base, we opted for a decision tree regression modelling strategy (Fig. 7A). We speculated that the use of a non-linear modelling architecture would be able to provide a correlation required to include the high diversity of ligand structural types. This model was constructed following feature reduction from the original 1242 features down to four using the permutation feature importance metric (on the test set) and subsequent hyperparameter tuning (see SI for full details) with a randomized 33 : 9 train:test split. The four features used in this model are: the Boltzmann-weighted average of the chloride NPA charge (maximum value of the two possible chloride ligands); the Boltzmann-weighted average of the phosphorus-backbone angle; the Boltzmann-weighted average of the P–Pd bond occupancy (average value of the two phosphine donors) and the conformer minimum value of the free ligand P–R bond occupancy (depicted in Fig. 7C). This combination of features indicates that there is an electronic and geometric dependence on the enantioselectivity for the evaluated reaction. For example, the chloride NPA partial charge provides an electronic readout of the phosphorus donor atom *trans* to the chloride. The two phosphorus-backbone angles could be related to the flexibility of the phosphorus donors with respect to the backbone that enforce the chiral metal environment. Details on the feature importance from the decision tree regression model can be gleaned by performing a SHAP analysis (Fig. 7B).^37^ This ranks features in order of importance: Boltzmann-weighted average of the chloride NPA charge; Boltzmann-weighted average of the phosphorus–backbone angle; Boltzmann-weighted average of the average P–Pd bond occupancy and the conformer minimum value of the free ligand P–R bond occupancy

Using the same metrics used for the MLR model analysis with the Hayashi–Heck cross-coupling reaction, the decision tree regression model provided excellent statistics for the training set (*R*^2^ = 0.96, MAE = 0.08 kcal mol^−1^, RMSE = 0.10 kcal mol^−1^). Aside from a clear outlier in the test set, the validation statistics were adequate (*R*^2^ = 0.45, MAE = 0.36 kcal mol^−1^, RMSE = 0.53 kcal mol^−1^). Analysis was performed on this outlier ligand, compared to the better predicted ligands in the test set with respect to their SHAP values and decision tree paths (see SI, Section 6.2.3). Unfortunately, this did not provide conclusive reasoning for the origin of this outlier. Removing this outlier gave *R*^2^ = 0.86, MAE = 0.22 kcal mol^−1^ and RMSE = 0.26 kcal mol^−1^. Conscious of the decision tree regression model overfitting the data, we performed a series of cross-validation analyses. A leave-one-out (LOO) cross-validation gave *R*^2^ = 0.49, MAE = 0.31 kcal mol^−1^ and RMSE = 0.31 kcal mol^−1^. Additionally, a five-fold cross-validation gave an average *R*^2^ = 0.31, MAE = 0.39 kcal mol^−1^ and RMSE = 0.51 kcal mol^−1^. Following these cross-validation tests, the effect of the random state and the training:test split ratio was examined. Using 100 randomly generated starts, an average test MAE = 0.38 kcal mol^−1^ was obtained. Changing the split ratio from 0.1 to 0.5 in 0.1 increments, gave an average test MAE = 0.43 kcal mol^−1^. While these analyses may suggest some degree of overfitting the data, we determined this model was adequate for the examination of conformer ensemble importance in bisphosphine ligand feature generation.

As before, the equivalent decision tree regression model was generated with the same hyperparameters and training:test split ratio was generated with the analogous lowest energy conformer features. In this model, almost identical training set statistics were obtained (*R*^2^ = 0.94, MAE = 0.10 kcal mol^−1^, RMSE = 0.12 kcal mol^−1^). However, the test set statistics showed significant deterioration (*R*^2^ = 0.06, MAE = 0.56 kcal mol^−1^, RMSE = 0.70 kcal mol^−1^). This can also be shown by comparing the absolute error distributions of both models in Fig. 7A. This indicated that use of lowest energy conformer features, compared to their conformer-weighted congeners, gave rise to a poorly predictive model.

Next, we investigated the degree of conformational dependence on the four features used in the decision tree regression model (Fig. 7C). This analysis used ligands L18, L19, L11 and L20, which are representative of the range of enantioselectivities obtained (full analysis of all ligands used in the data set is provided in the SI). With the exception of ligand L19, all ligands exhibit conformational flexibility. This is consistent with the hypothesis that including conformational flexibility in feature design can enhance model performance.

## Conclusions

In conclusion, we have developed a method of selecting conformers for bisphosphine-ligated palladium(ii) dichloride complexes that provides balance between representation and practicality in terms of computational time. In addition to this, strategy relies on chemical intuition by utilizing bite angle, a historically important stereo-electronic bisphosphine feature for conformer down selection. This was accomplished by the finding that geometries from the CREST conformer search were sufficiently accurate, with respect to their steric and geometric features, compared to DFT-refined geometries. Balance between representation and computational cost as well as providing a chemically intuitive means of conformer selection are critical considerations when constructing a DFT feature library. The new descriptor set was applied to statistical modelling campaigns of two previously analysed palladium-catalysed reactions which employ bisphosphine ligands. An MLR model was generated for a Hayashi–Heck cross-coupling case study which showed improved performance with the use of conformer-weighted features compared to their lowest energy conformer equivalents. The increased diversity in ligand sampling for the SIA carbonylation case study, compared to the Hayashi–Heck cross-coupling example, presented additional challenges in modelling campaigns and required the use of a decision tree regressor to describe ligand performance. Using this non-linear modelling technique, we were also able to showcase the improved model performance when using conformer-weighted features. Additional work on enhanced bisphosphine ligand training set design and featurisation are currently underway to address further challenges one might encounter when undertaking modelling campaigns with bisphosphine ligands (*e.g.*, improved feature design) and will be reported in due course. Nevertheless, this chemically intuitive feature-based conformer selection methodology has the potential to be applied to complex molecular systems (*i.e.*, one which is large and flexible) beyond bisphosphine ligands where an important steric or geometric feature is anticipated.

## Author contributions

J. A. C., S. D. H. and M. S. S. conceptualised the project. J. A. C. and S. D. H. developed the methodology, ran the calculations and modelled the reaction data. J. A. C., S. D. H., R. C. W., K. A. M. and M. S. S. analysed the data and refined the methodology. J. A. C. wrote the initial draft that was reviewed and edited by J. A. C., S. D. H., R. C. W., K. A. M. and M. S. S. All authors have given approval to the final version of the manuscript.

## Conflicts of interest

There are no conflicts of interest to declare.

## Supplementary Material

SC-OLF-D5SC04691B-s001

## Data Availability

Data for this article, including Python code used for data analysis, conformer selection and modelling, are available on GitHub in this repository: https://github.com/SigmanGroup/BisphosphineConformerSelection. DFT calculation files used in both modelling case studies are available on Zenodo: https://doi.org/10.5281/zenodo.15690854. Supplementary information is available containing full calculation, modelling details and additional plots depicting conformer selection. See DOI: https://doi.org/10.1039/d5sc04691b.
