# Supplementary material for: Bisphosphine ligand conformer selection to enhance descriptor database representation: improving statistical modelling outcomes
Source: Chem Sci. 2025 Sep 29;16(43):20473–85. doi: 10.1039/d5sc04691b (PMC12498247; doi:10.1039/d5sc04691b)
Supplement: SC-016-D5SC04691B-s001 [file SC-016-D5SC04691B-s001.pdf]

Supplementary Information (SI) for Chemical Science.
This journal is © The Royal Society of Chemistry 2025

Bisphosphine Ligand Conformer Selection to Enhance Descriptor Database Representation: Improving Statistical Modelling Outcomes

Supporting Information

Jamie A. Cadge,† Sierra D. Hart,† Richard C. Walroth,‡ Kyle A. Mack,‡ and Matthew S. Sigman*,†

†*Department of Chemistry, University of Utah, Salt Lake City, Utah 84112, United States*

‡*Department of Small Molecule Process Chemistry, Genentech Inc., San Francisco, California 94080, United States*

E-mail: matt.sigman@utah.edu

Contents

1 Computational details **S4**

1.1 Conformational searches S4

1.2 DFT calculations S4

1.3 Feature collection and definitions S5

1.3.1 Steric features S5

1.3.2 Geometric features S6

1.3.3 Electronic features S7

1.4 Features used for modelling S8

2 Representative ligand selection **S9**

2.1 *t*-Distributed stochastic neighbor embedding (*t*-SNE) S9

2.2 Principal component analysis (PCA) S11

2.3 Final ligand selection S12

3 Conformer generation **S13**

3.1 Effect of energy window S14

3.2 Effect of conformer ensemble clustering S17

4 DFT-refined *vs* non-DFT-refined features **S29**

4.1 Lowest energy conformer features S30

4.2 Boltzmann weighted averaged features S33

4.3 Minimum and maximum conformer feature values S37

5 Conformer selection **S47**

5.1 Selection of conformers with equidistant feature values S47

5.2 Selection from GFN2-xTB energies S48

5.2.1 Generation of equidistant conformers S48

5.3 Selection from steric and geometric features S48

5.3.1 Generation of equidistant conformers S48
5.3.2 Effect of selection based on GFN2-xTB energy on the DFT ensemble conformers S73
5.3.3 Effect of selection based on bite angle on the DFT ensemble conformers S73
5.3.4 Effect of selection based on percent buried volume on the DFT ensemble conformers S73

6 Conformer-weighted features in modelling campaigns S110

6.1 Hayashi-Heck cross-coupling S111
6.1.1 Initial feature reduction S111
6.1.2 Regioselectivity dataset multivariate linear regression model S112
6.1.3 Analysis of feature conformer dependence S116
6.1.4 Mulivariate linear regression models with *only* lowest energy conformer features S121
6.1.5 Model comparison and discussion S124
6.2 Sulfonimidamide aryl carbonylation S124
6.2.1 Data curation S124
6.2.2 Initial model search S125
6.2.3 Enantioselectivity decision tree regression model and feature importance S129
6.2.4 Model overfitting checks S134
6.2.5 Effect of model hyperparameters S137
6.2.6 Analysis of feature conformer dependence S143
6.2.7 Decision tree regression models with *only* lowest energy conformer features S143
6.2.8 Alternative multivariate linear regression models S151
6.2.9 Model comparison and discussion S154

References S155

1 Computational details

Starting coordinates for (P,P)$PdCl_2$ complexes were obtained from lowest energy conformer DFT-optimized geometries from the previously reported bisphosphine library from Sigman, Mack *et al.*[1] The method of selection of complexes from the library is outlined in detail in Section 2.

1.1 Conformational searches

All conformer ensembles were generated using the *Conformer-Rotomer Ensemble Sampling Tool (CREST) version 2.12* from Grimme and co-workers.[2] Conformer searches were conducted using the default iMTD-GC algorithm (ignoring initial topology checks) in a 5 kcal mol^{-1} or 10 kcal mol^{-1} energy window. To minimize computational cost, the GFN2-xTB//GFN-FF composite method (using *xTB version 6.4.0*)[3] was used where sampling and structures are optimized at the GFN-FF level[4] followed by a single-point energy calculation at the GFN2-xTB level.[5] To preserve the square-planar geometry of the (P,P)$PdCl_2$, constaints were applied to all angles at Pd (*i.e.*, ∠P–Pd–P, ∠P–Pd–Cl and ∠Cl–Pd–Cl) with a force constant of 1.0. All other atoms were allowed to move without restriction. Clustering of generated conformer ensembles was performed using PCA/k-means clustering with dihedral angles as implemented in *CREST*.

1.2 DFT calculations

All density functional theory (DFT) calculations were performed using *Gaussian16, Revision C.01*[6] (referred to as *Gaussian16*) at standard temperature and pressure (298.15 K and 1 atm.) in the gas phase.

The PBE functional[7,8] including Grimme's D3 dispersion correction[9] with Becke-Johnson damping[10] was used for geometry refinements with Ahlrichs' double-ζ def2-SVP basis set.[11] The 28-electron def2 pseudopotential was used for Pd.[12–14] The nature of stationary points

was confirmed by analysis of the harmonic vibrational frequencies.

The PBE0 functional[15,16] including Grimme's D3 dispersion correction[9] with Becke-Johnson damping[10] was used for single-point energy calculations with Ahlrichs' triple-ζ def2-TZVP basis set.[11] The 28-electron def2 pseudopotential was used for Pd.[12–14] Natural bond orbital (NBO) analyses were performed using *NBO version 7.0* as implemented in *Gaussian16*.[17]

1.3 Feature collection and definitions

Steric, geometric and electronic features were either extracted directly from *Gaussian16* output files or using the *MORFEUS* Python package[18] as indicated below. In instances where steric and geometric features were collected directly from a *CREST* conformational search, features were collected directly from the output XYZ coordinate files using *MORFEUS*.

Although some of the below features may be categorized as 'stereoelectronic", they have been broadly classified as steric, geometric and electronic for convenience.

1.3.1 Steric features

Solid cone angle (Θ): Derived from a ligand solid angle (Ω) calculation in *MORFEUS*.[18,19] Ω is calculated from the shadow cast from a hypothetical light source originating at the Pd center. Chloride ligands are removed prior to calculation. Additionally, Θ is analogous to Tolman's cone angle commonly used as a steric description of monophosphine ligands.

Percent buried volume ($\%V_{bur}$): The percentage of a sphere volume of a defined radius occupied by a ligand centered at Pd.[20] Radii used here are 2–7 Å in increments of 1 Å and are calculated using *MORFEUS*. Chloride ligands and hydrogen atoms are removed from the complex prior to the determination of $\%V_{bur}$.

Molecular volume: Derived from a DFT single point energy calculation in *Gaussian16*[6] us-

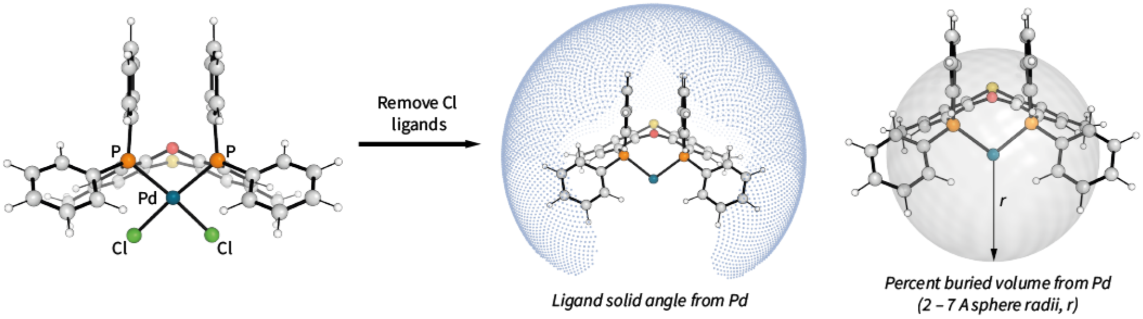

Figure S1: Derivation of ligand solid angle and buried volume features.

ing the `volume` keyword. Defined as the volume inside a contour of 0.001 electrons/Bohr^3.

Solvent accessible surface area (SASA): A measure of how much area of a molecule is available to solvent as implemented in *MORFEUS*.[18,21] In this case, the atomic SASA[22] is measured at Pd representing the steric availablity at that atom. Chloride ligands are removed prior to calculation of SASA.

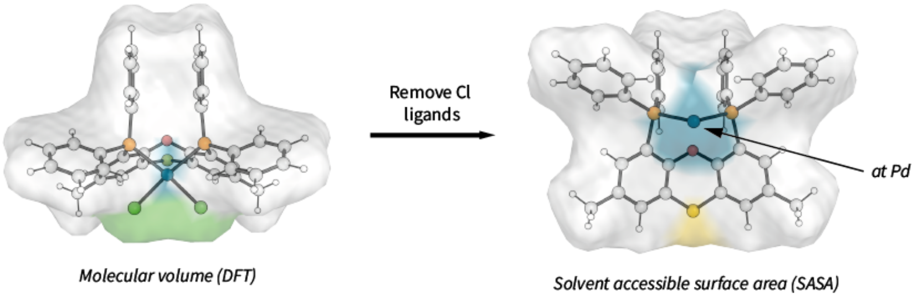

Figure S2: Derivation of molecular volume and solvent accessible surface area (SASA) features.

1.3.2 Geometric features

Bond distances: Calculated directly from the DFT-optimized Cartesian coordinates following Equation 1. The two sets of Cartesian coordinates for atom 1 and atom 2 are defined as (X_1, Y_1, Z_1) and (X_2, Y_2, Z_2), respectively. Calculations were conducted using the NumPy Python package.

$$d = \sqrt{(x_2 - x_1)^2 + (y_2 - y_1)^2 + (z_2 - z_1)^2} \quad (1)$$

Bite angle: Defined as the P–Pd–P bond angle and calculated using *MORFEUS*.

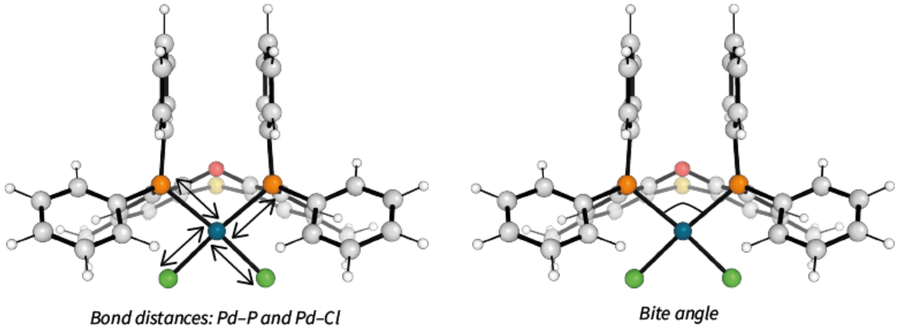

Figure S3: Derivation of ligand bond length and bite angle features.

1.3.3 Electronic features

Frontier molecular orbitals: Defined as the highest occupied and lowest unoccupied Kohn-Sham molecular orbital (HOMO and LUMO, respectively) energies. These are derived from a *Gaussian16*[6] single point energy calculation at the PBE0/def2-TZVP level of theory.

Dipole: Molecule dipole moment calculated in a *Gaussian16* single point energy calculation using the `polar` keyword.

NPA charges: Atomic charges derived from a Natural Population Analysis in *NBO 7.0* as implemented in *Gaussian16* using the `pop=nbo7` keyword. Charges were extracted from the *Gaussian16* output file for Pd, P and Cl atoms.

NMR chemical shifts: Isotropic and anisotropic NMR chemical shifts were calculated in a *Gaussian16* single point energy calculation. The `nmr` keyword was used to generate NMR properties using the Gauge-Independent Atomic Orbital (GIAO) method.[23,24] Chem-

ical shifts were extracted directly from the *Gaussian16* output file.

1.4 Features used for modelling

Features generated for modelling of reaction data were generated using the approach previously published by our group.[1] Examples of scripts used to generate features can be found on GitHub: https://github.com/SigmanGroup/Multiobjective_Optimization. Descriptions of features may also be found in the Supporting Information of this work (https://pubs.acs.org/doi/suppl/10.1021/jacs.2c08513/suppl_file/ja2c08513_si_003.pdf). These features were then weighted according to their conformer ensembles generated using the methodology outlined above to include the lowest energy conformer value, mathematic minimum and maximum values, Boltzmann-weighted average (298 K) and arithmetic mean.

2 Representative ligand selection

Data availability:

- Scripts used to process the bisphosphine ligand features from the original library and dimensionality reduction described in the below sections can be found on this publication's GitHub repository: https://github.com/SigmanGroup/BisphosphineConformerSelection.

In order to select a representative set of ligands for testing the effects of feature conformer-weighting, chemical space maps were derived using the techniques outlined below to give maximum representation across our existing bisphosphine ligand library.[1] The original library was curated to contain only free ligand features (with the exception of those based on complexes such as buried volume) as it was hypothesized that it would provide better overall representation. This brought the total number of features down to 91. On this reduced feature set, a co-linearity cutoff was applied with a Pearson correlation coefficient of 0.95 as a further curation step (leaving 74 features). Finally, before applying the desired dimensionality reduction technique, the curated feature set was scaled using the standard scaler as implemented in scikit-learn.[25,26]

2.1 *t*-Distributed stochastic neighbor embedding (*t*-SNE)

The chemical space map generated with *t*-SNE was generated using the reduced in scikit-learn with two components, a perplexity of 40, 1000 iterations with a random state set to 10. This space was clustered using a hierarchical clustering method with the Ward variance minimization algorithm to give 10 clusters (Figure S4). This number of clusters was selected based on the silhouette score of which the maximum was defined as eight clusters (Figure S5). An additional two clusters were included to increase the overall ligand diversity. These clusters group ligand structures together in a chemically intuitive manner (*i.e.*, by similar backbone structures).

In order to select ligands from each cluster, the ligand closest to the cluster centroid was used. The centroids of each cluster were determined by taking the mean position of all the points in the cluster according to Equation 2. Here, $\sum(x, y)$ is the sum of all the Cartesian (x, y) t-SNE coordinates and n is the total number of points in the cluster.

$$\text{cluster centroid} = \frac{\sum(x, y)}{n} \tag{2}$$

As this approach gave the mathematical centroid of the cluster, it was necessary to determine the closest *real* ligand to that centroid. This was accomplished by finding the five nearest ligands in Euclidean space to this centroid that provided a means of selecting a ligand from each cluster.

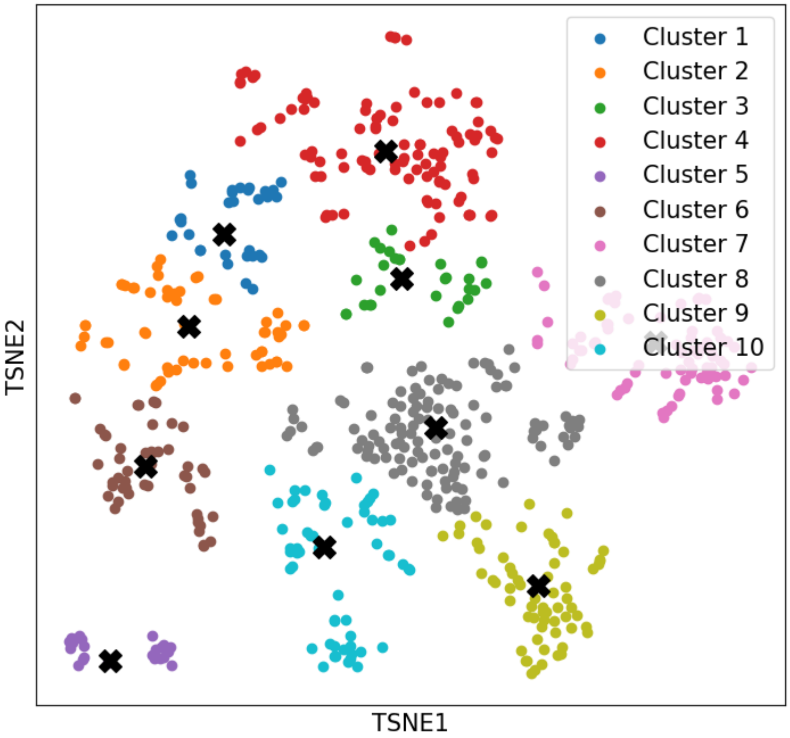

Figure S4: Clustered t-SNE chemical space with centroids.

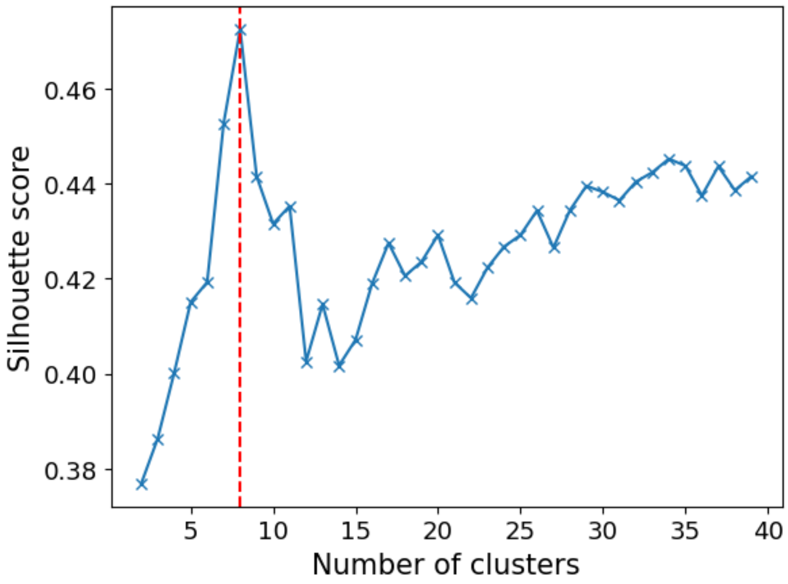

Figure S5: Hierarchical clustering silhouette score for the t-SNE chemical space.

2.2 Principal component analysis (PCA)

To confirm the approach using t-SNE, PCA was also used to generate a chemical space map. Using the PC reducer in scikit-learn, five PCs were used to generate the chemical space which represented 67% of the total variance. Variance of the individual PCs is given in Table S1. This space was also clustered using the k-means technique to give 13 clusters, based loosely on a plot of sum of squared distances *vs* number of clusters (Elbow method). Ligands closest to the cluster centroids were determined using a method similar to that outlined in Equation 2. This space is shown in Figure S6.

Table S1: Variance of individual PCs in the bisphosphine ligand PCA chemical space.

Component	% Variance
PC1	20.6
PC2	17.5
PC3	13.5
PC4	9.1
PC5	6.2

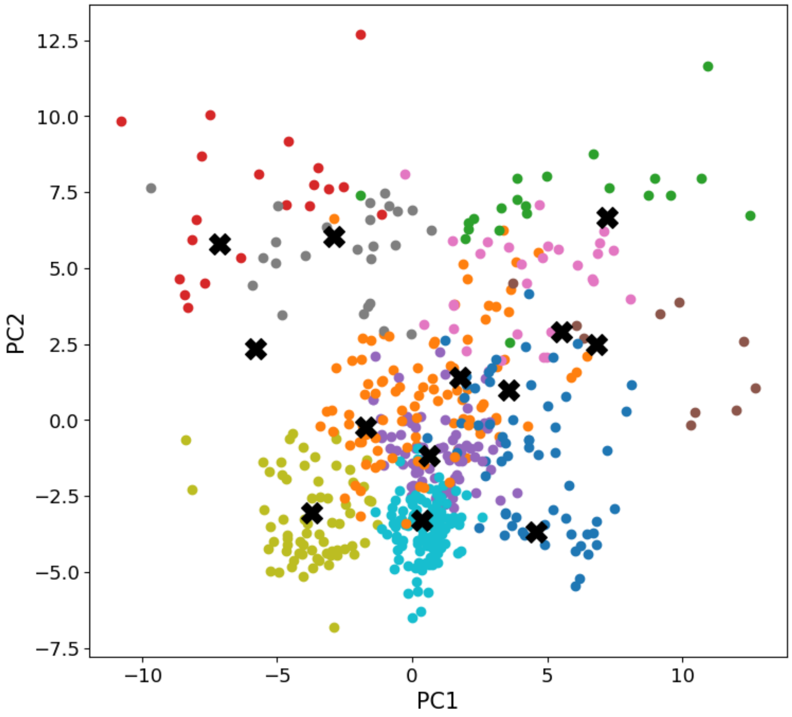

Figure S6: Clustered PCA chemical space with centroids.

2.3 Final ligand selection

Final ligand selection was determined using the *t*-SNE and PCA feature spaces shown above. As the clusters in *t*-SNE generally categorised ligand scaffolds more intuitively than PCA, the centroids from this clusters (ten ligands) were initially selected to examine workflow performance. The PCA space was also used to enhance the selection - giving a total of 12 ligands selected depicted in Figure S7.

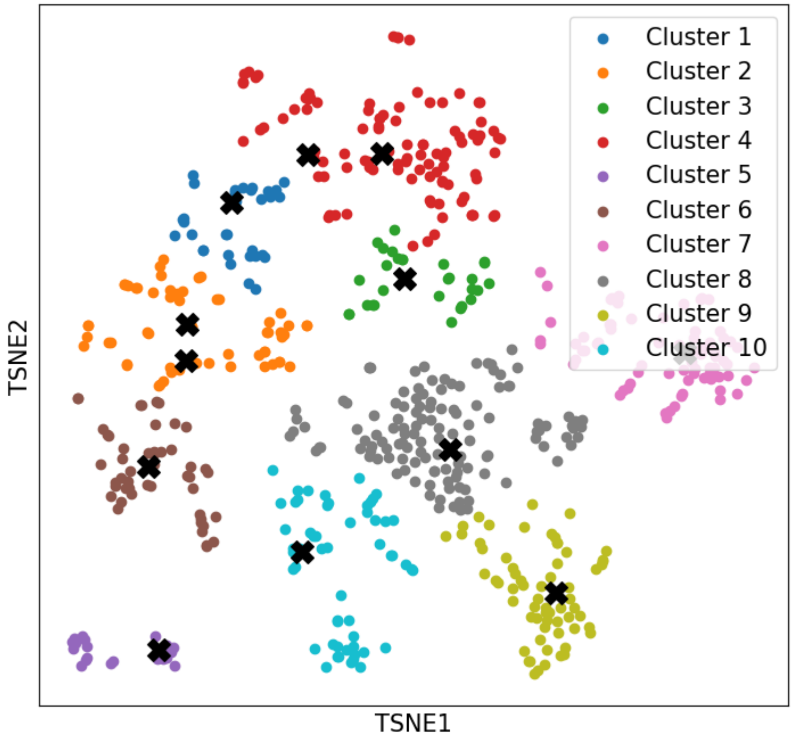

Figure S7: Final bisphosphine ligand selection (12 ligands in total) shown in the t-SNE representation in t-SNE clusters.

3 Conformer generation

Data availability:

- Scripts used to generate plots in the below sections as well as calculation of RMSD values and features can be found in this publication's GitHub repository: https://github.com/SigmanGroup/BisphosphineConformerSelection.
- XYZ coordinate files for the structures generated from CREST conformer searches can be found in the `./calculation_files` directory in the above GitHub repository.

Conformer ensembles were generated using *CREST 2.12* as described in Section 1.1. Below the effect of performing conformer searches at different energy windows (5 kcal mol^{-1} and 10 kcal mol^{-1}) was investigated in terms of number of conformers generated and the resultant distribution of molecular features. Additionally, within these two energy windows, the effect of filtering conformer ensembles using the PCA/k-means clustering extension was

examined.

3.1 Effect of energy window

Unsurprisingly, the number of conformers increased when the energy window is increased from 5 kcal mol^{-1} to 10 kcal mol^{-1} (Figure S8). On average the numbers of conformers increased from 116 to 352 per complex. As the Boltzmann weight for conformers with an energy of over 3 kcal mol^{-1} is likely to contribute very little to features at 298.15 K. Therefore, an energy window of 10 kcal mol^{-1} likely results in a number of inaccessible conformers in a catalytic scenario. Therefore, an energy window of 5 kcal mol^{-1} was defined to strike balance of number of energetically accessible conformers between these two values.

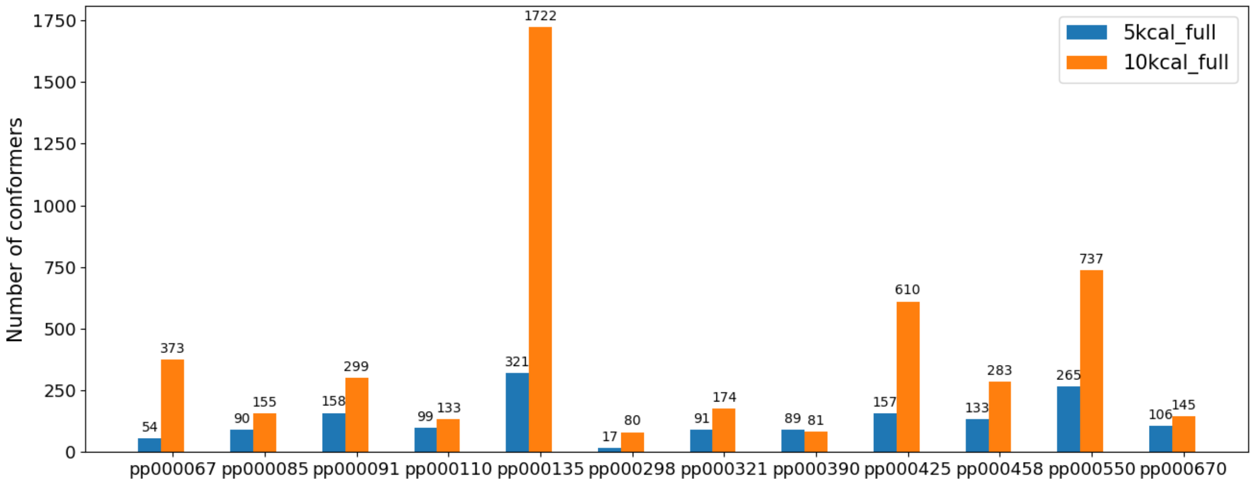

Figure S8: Number of conformers by ligand in ensembles generated with 5 kcal mol^{-1} and 10 kcal mol^{-1} energy windows.

In addition to the energetic accessibility of certain conformers, the structural diversity of conformers in the 5 kcal mol^{-1} and 10 kcal mol^{-1} windows was also examined. Firstly, the non-weighted root mean squared deviation (RMSD) of atomic positions across the conformer ensemble, with the lowest energy conformer as the reference molecule (RMSD = 0 Å). This analysis was performed using the *MDAnalysis* Python package.[27,28] For each conformer histograms were plotted for all the RMSD positions describing the overall movement of the complex across the 5 kcal mol^{-1} and 10 kcal mol^{-1} ensembles (Figure S9). The mean,

standard deviation, and range for the atomic position RMSDs for each complex are given in Table S2.

Table S2: Mean, standard deviation and mean non-weighted atomic RMSD positions in Å for conformer ensembles with 5 kcal mol^{-1} and 10 kcal mol^{-1} energy windows.

	RMSD / Å					
	5 kcal mol^{-1}			10 kcal mol^{-1}		
Complex	Mean	Standard deviation	Range	Mean	Standard deviation	Range
**pp000067**	0.784	0.300	1.306	1.218	0.295	1.819
**pp000085**	0.710	0.480	3.225	1.231	1.006	4.197
**pp000091**	2.535	0.986	3.927	2.180	0.491	3.750
**pp000110**	0.838	0.424	2.434	1.049	0.380	1.949
**pp000135**	2.936	0.908	5.026	3.322	0.969	5.032
**pp000298**	0.817	0.423	1.461	0.730	0.359	1.365
**pp000321**	1.352	0.650	2.157	1.851	0.484	2.554
**pp000390**	1.430	0.435	2.236	2.421	0.447	2.936
**pp000425**	1.239	0.705	3.982	2.046	0.802	4.594
**pp000458**	1.039	0.451	2.701	1.600	0.638	2.779
**pp000550**	1.963	0.785	6.126	3.103	1.195	6.573
**pp000670**	1.379	0.517	3.112	1.029	0.622	3.211

For many of the complexes, the distributions of RMSD atomic positions across the conformer ensembles with 5 kcal mol^{-1} and 10 kcal mol^{-1} energy windows show little differences. Notable exceptions are complexes **pp000067** and **pp000390**. To examine this further, the RMSD atomic positions for both ligands were plotted as a function of conformer number (Figure S10) for the full complex as well as only the phosphorus atoms. In both cases, using these RMSD plots and visual examination of the structural changes showed that the relatively rigid structures of **pp000067** and **pp000390** led to a modest structural variation at 5 kcal mol^{-1}. At 10 kcal mol^{-1}, there is an increased amount of flexibility at this higher energy window that is reflected in the plots in Figure S10. Similarities between the two energy windows can also been seen in the RMSD statistics in Table S2. The mean RMSD values are generally higher with the 10 kcal mol^{-1} window indicating additional overall ligand flexibility; however, the ranges of RMSD values between the two windows are similar. In general, this indicates that there is no additional structural diversity achieved by doubling

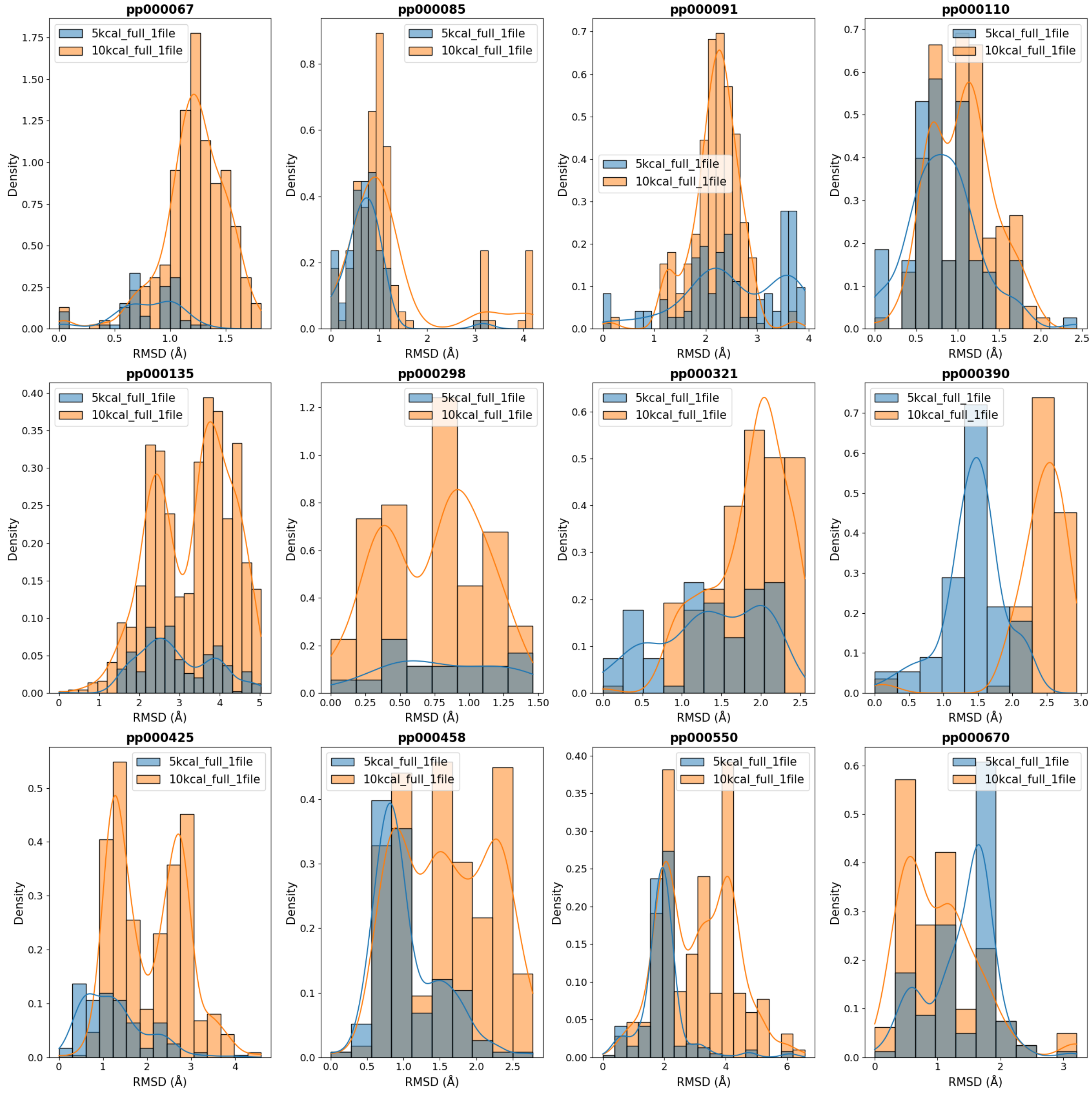

Figure S9: Comparison of non-weighted atomic RMSD positions in Å across the full and clustered conformer ensembles (5 kcal mol^{-1}).

the conformer search energy window from 5 kcal mol^{-1} to 10 kcal mol^{-1}.

To further examine this hypothesis, the changes in bite angle, percent buried volume and equivalent cone angle were analyzed across all conformer ensembles. The feature distributions for 5 kcal mol^{-1} and 10 kcal mol^{-1} are given in Figures S11, S12 and S13, respectively. As with the RMSD atomic positions **pp000067** and **pp000390** show differences in their descriptor distributions, likely as a result of the expansion of conformer space from the increased energy window. Differences between the 5 kcal mol^{-1} and 10 kcal mol^{-1} feature histograms are also noted for **pp000085** and **pp000298**.

3.2 Effect of conformer ensemble clustering

Even by using an energy window of 5 kcal mol^{-1}, in some case a large number of conformers are generated. In practical terms, this would give a large number of complexes to calculate at DFT level of theory. To further reduce the number of conformers, the effect of clustering the ensembles using the PCA/k-means clustering method as implemented in *CREST* was examined. The bar graph in Figure S15 shows the difference in the number of conformers before and after clustering. On average across the 12 complexes, clustering reduced the number of conformers from 116 to 13. Following the same approach as in Section 3.1, the differences in non-weighted RMSDs of atomic positions between the full and clustered ensembles were investigated. The mean, standard deviation and range for each complex are given in Table S3. Histograms displaying the RMSD data for each complex are given in Figure S16. For all complexes, there is good overlap in the histograms given in Figure S16. Additionally, similar consistency in the RMSD statistics in Table S3 is shown.

Next, the effect of ensemble clustering on bite angle, percent buried volume and solid cone angle feature values were investigated. These results are given as histograms in Figures S17, S18 and S19. As seen with the RMSD atomic positions, good overlap of feature values in the histograms are shown for several complexes. *I.e.,* the ranges of feature values and the shape of the distribution density (kernel density estimation as implemented in the *Seaborn*[29]

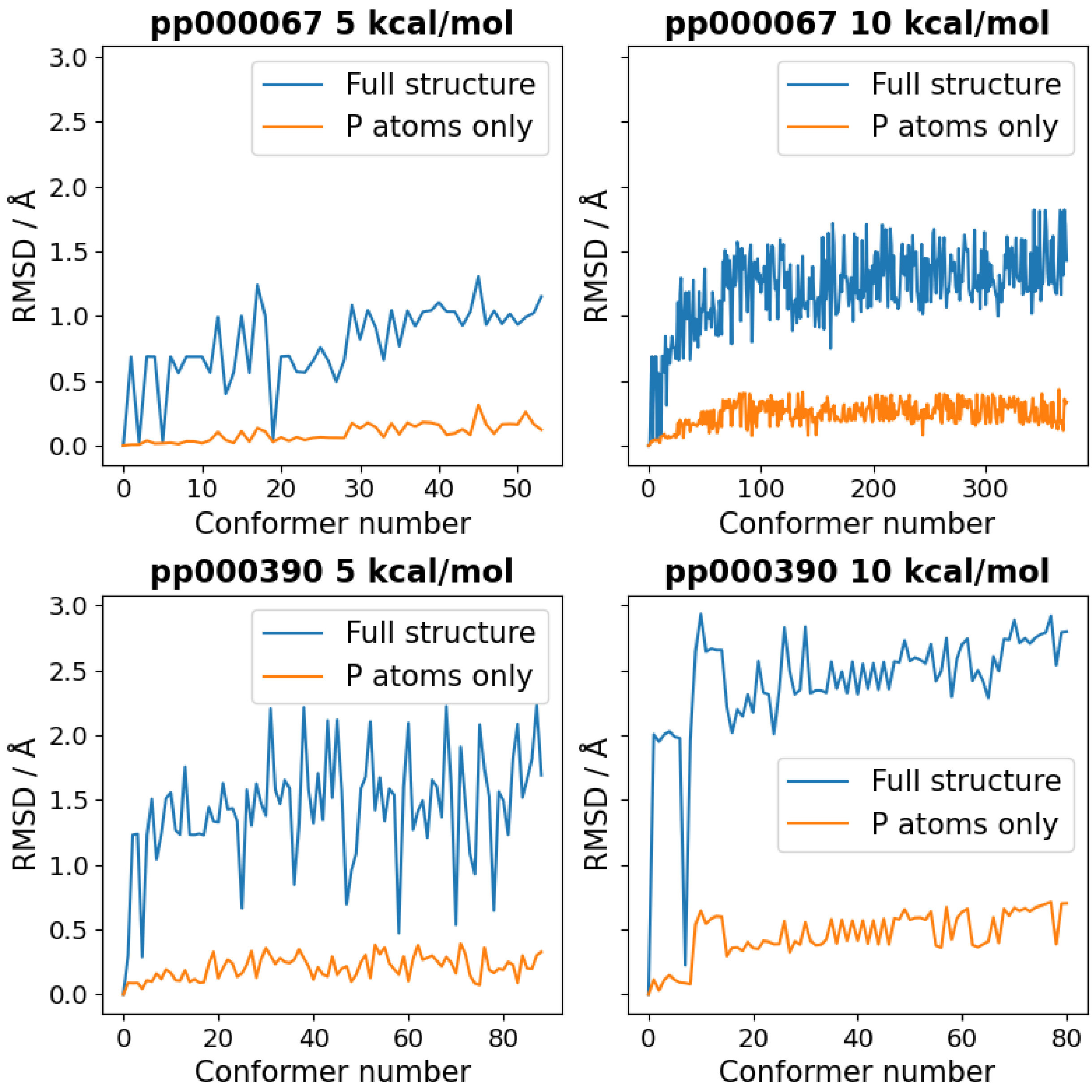

Figure S10: Individual RMSD analyses for **pp000067** and **pp000390** for their respective 5 kcal mol^{-1} and 10 kcal mol^{-1} conformer ensembles.

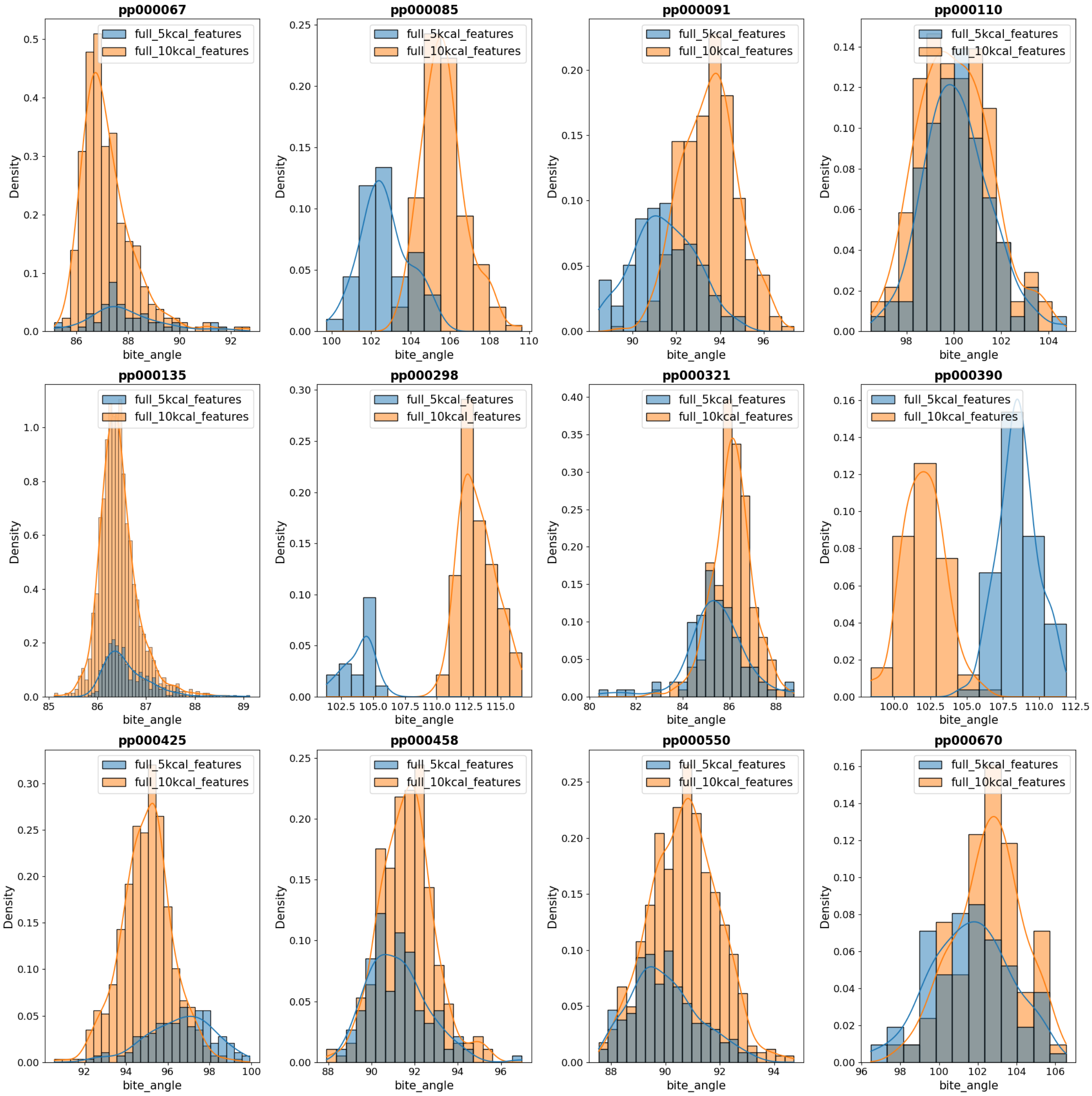

Figure S11: Comparison of bite angle distributions between the conformer ensembles with 5 kcal mol^{-1} and 10 kcal mol^{-1} energy windows.

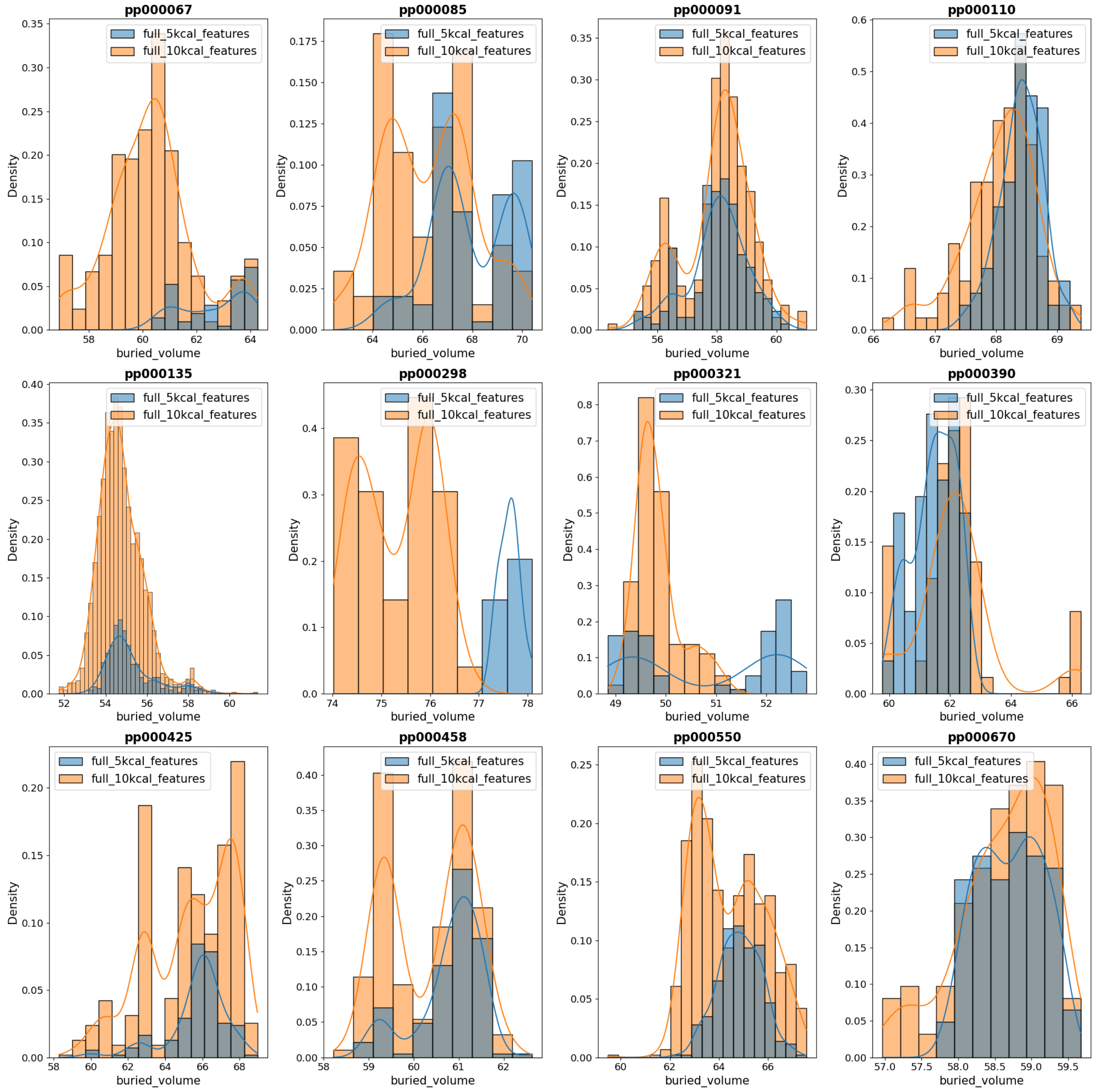

Figure S12: Comparison of percent buried volume distributions between the conformer ensembles with 5 kcal mol^{-1} and 10 kcal mol^{-1} energy windows.

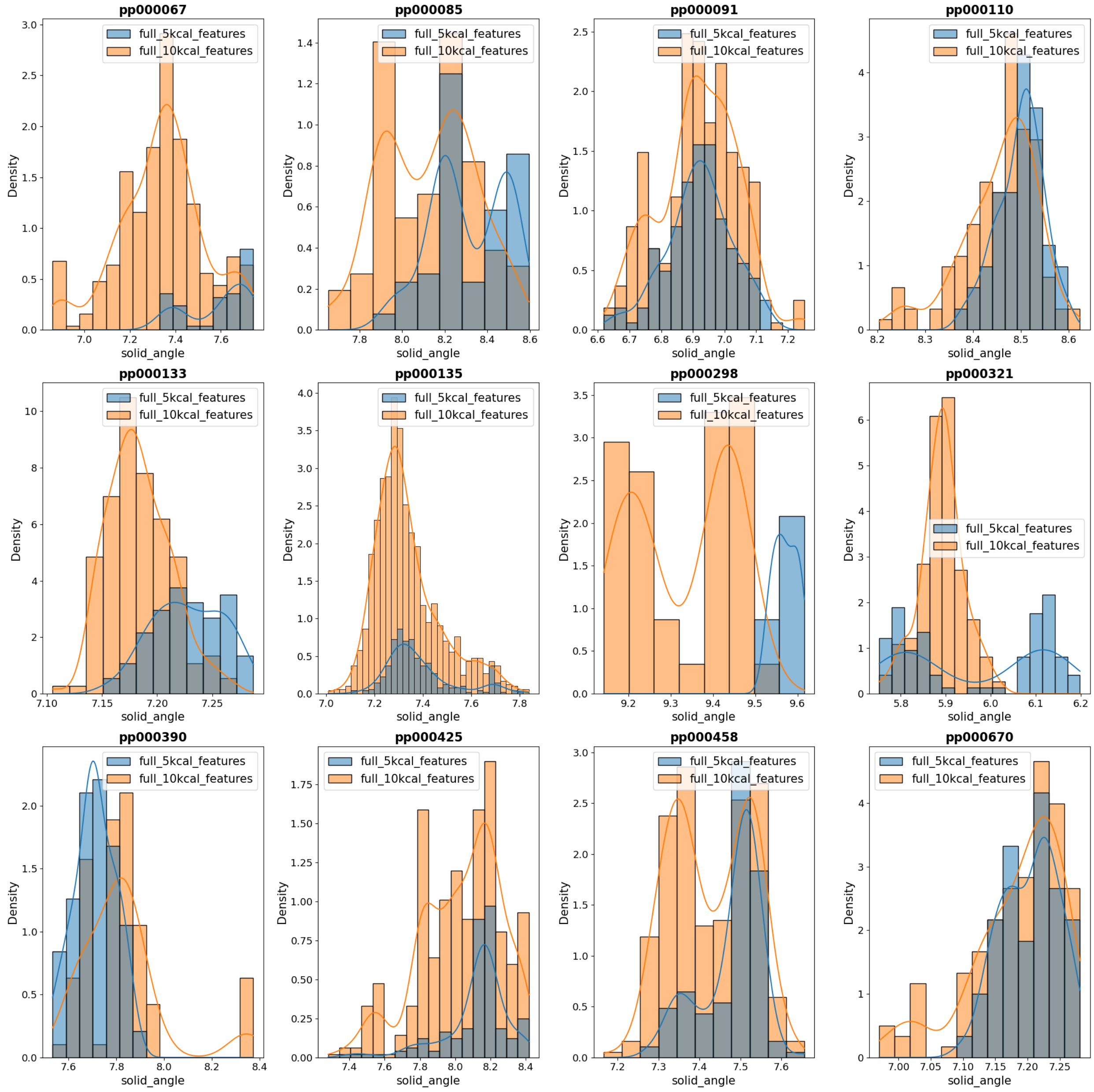

Figure S13: Comparison of equivalent cone angle distributions between the conformer ensembles with 5 kcal mol^{-1} and 10 kcal mol^{-1} energy windows.

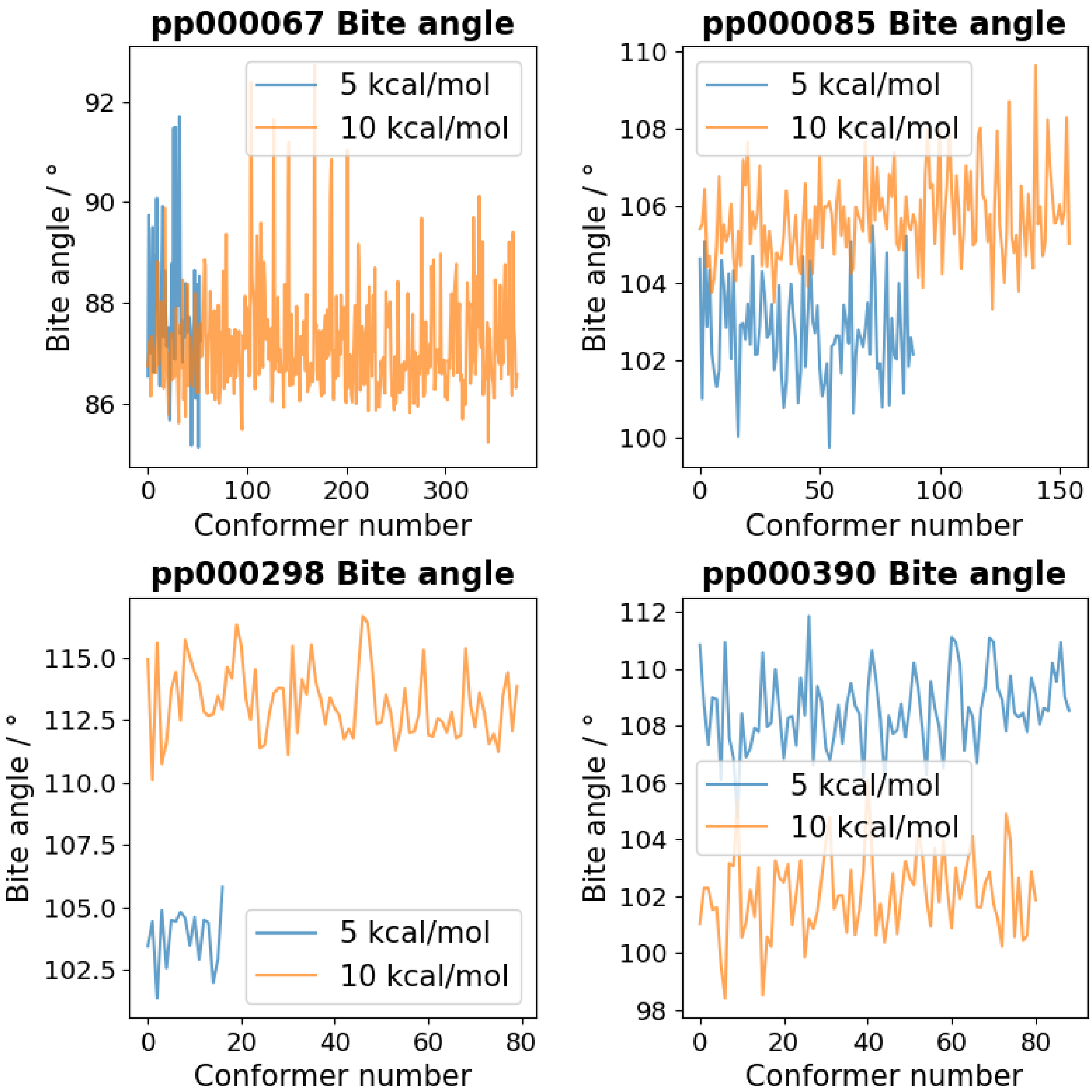

Figure S14: Individual bite angle analyses (as a function of conformer) for **pp000067**, **pp000085**, **pp000298** and **pp000390** for their respective 5 kcal mol^{-1} and 10 kcal mol^{-1} conformer ensembles.

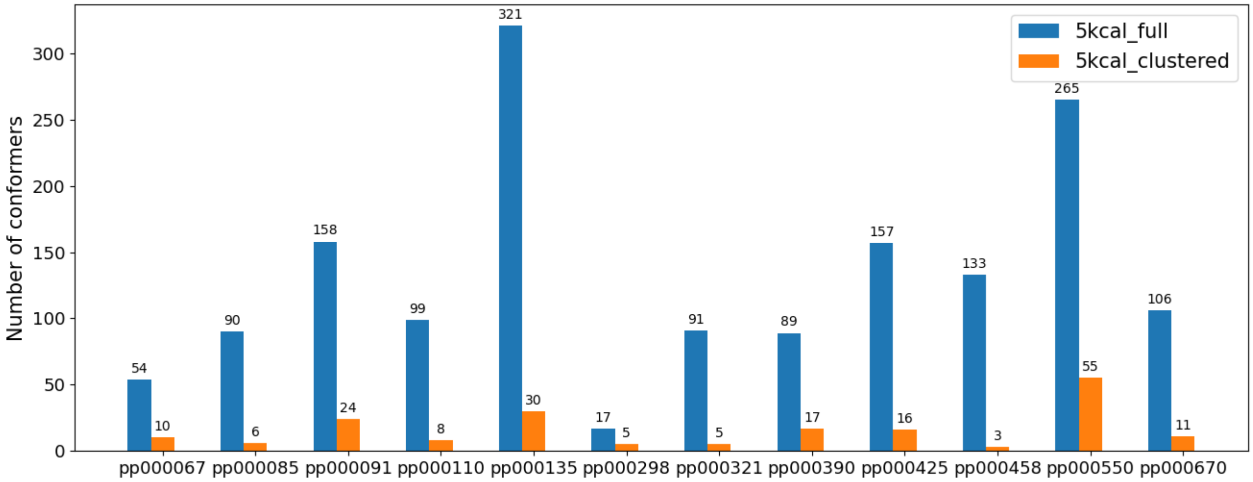

Figure S15: Number of conformers by ligand in ensembles generated with a 5 kcal mol^{-1} energy window before and after clustering using the PCA/k-means clustering method as implemented in *CREST*.

Table S3: Mean, standard deviation and mean non-weighted atomic RMSD positions in Å for full and clustered conformer ensembles.

	RMSD / Å					
	Full ensemble			Clustered ensemble		
Complex	Mean	Standard deviation	Range	Mean	Standard deviation	Range
**pp000067**	0.784	0.300	1.306	0.920	0.387	1.306
**pp000085**	0.710	0.480	3.225	1.422	1.382	3.225
**pp000091**	2.535	0.986	3.927	2.817	0.936	3.801
**pp000110**	0.838	0.424	2.434	1.327	0.706	2.434
**pp000135**	2.936	0.908	5.026	3.008	1.193	4.792
**pp000298**	0.817	0.423	1.461	0.755	0.559	1.380
**pp000321**	1.352	0.650	2.157	1.442	0.844	2.106
**pp000390**	1.430	0.435	2.236	1.369	0.616	2.215
**pp000425**	1.239	0.705	3.982	1.657	0.787	5.065
**pp000458**	1.039	0.451	2.701	0.892	0.846	1.684
**pp000550**	1.963	0.785	6.126	2.132	0.687	4.707
**pp000670**	1.379	0.517	3.112	1.600	0.779	3.113

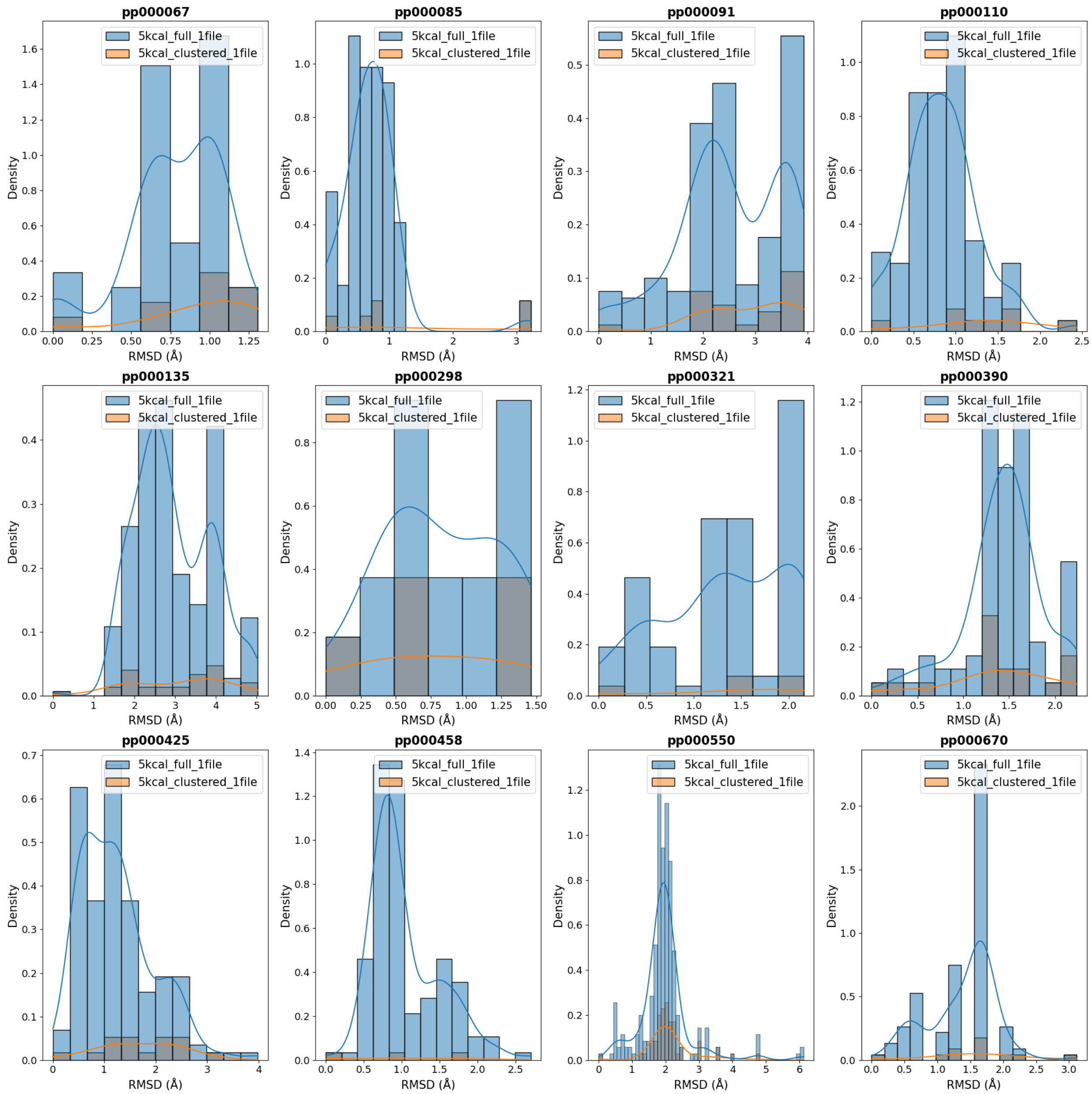

Figure S16: Comparison of non-weighted atomic RMSD positions in Å across the full and clustered conformer ensembles (5 kcal mol^{-1}).

Python package) are broadly consistent between clustered and non-clustered ensembles. Minor exceptions are observed for complexes **pp000321**, **pp000458** and **pp000670**.

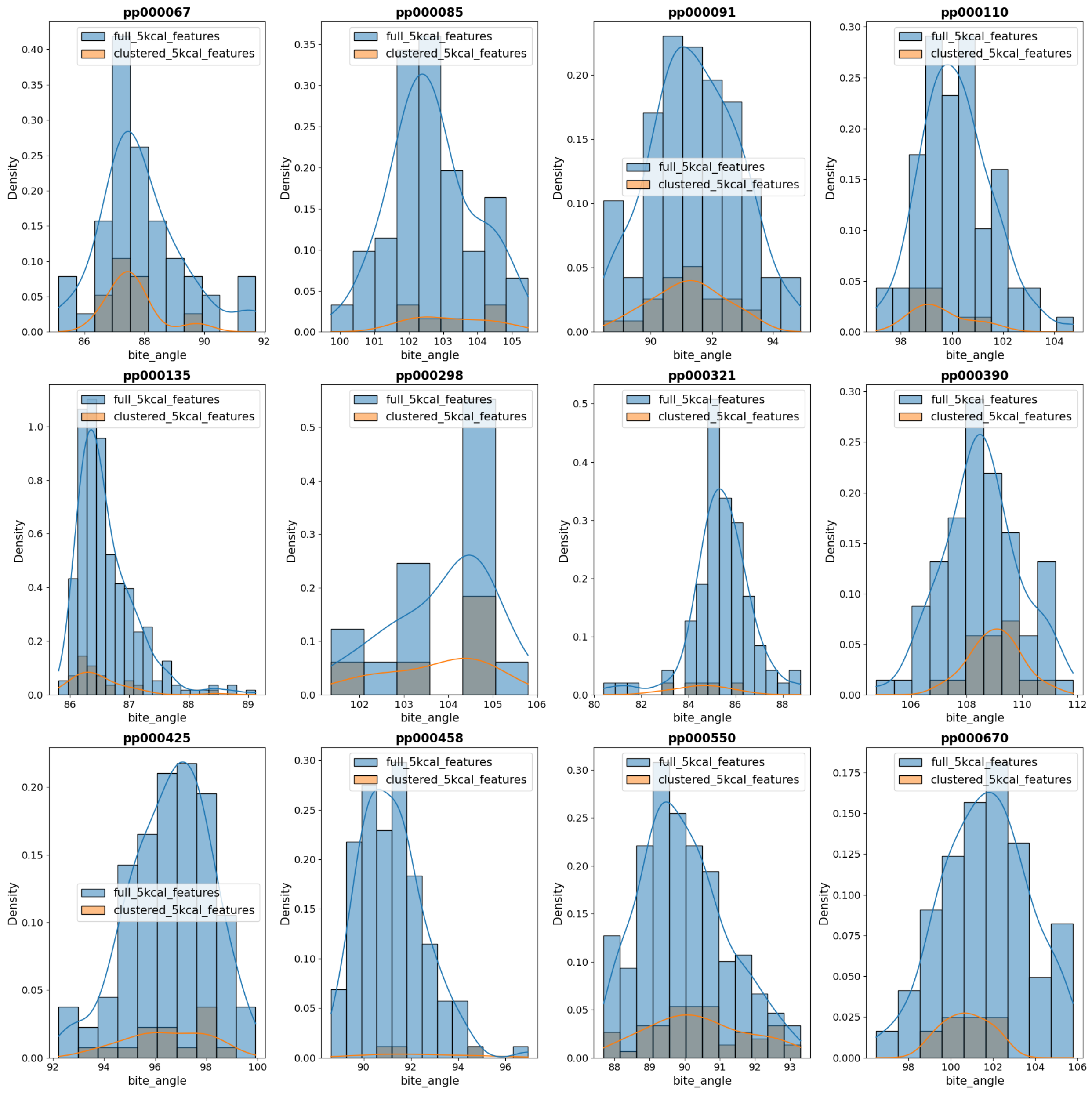

Figure S17: Comparison of bite angle across the full and clustered conformer ensembles (5 kcal mol^{-1} energy window).

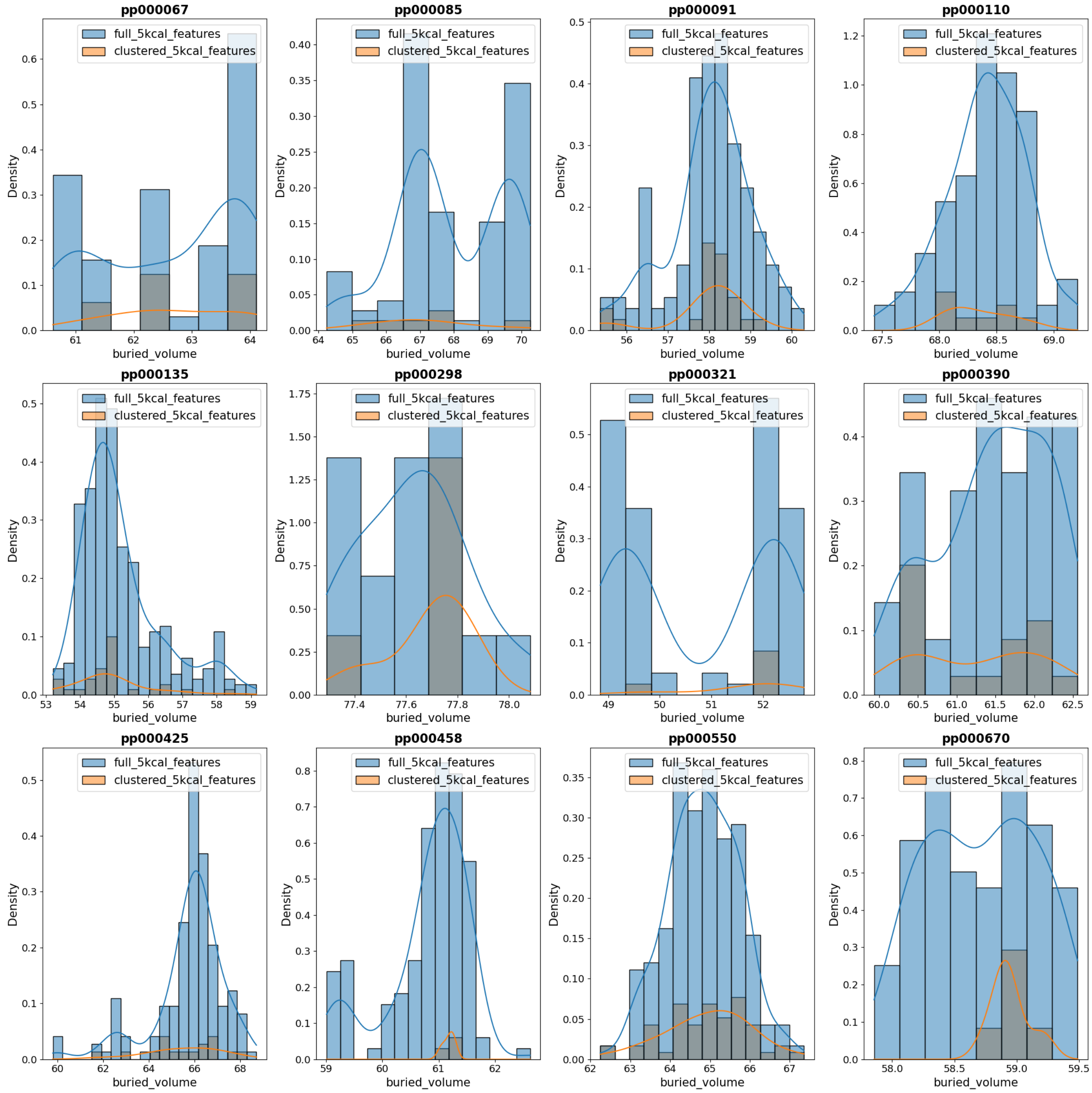

Figure S18: Comparison of buried volume across the full and clustered conformer ensembles (5 kcal mol^{-1}) energy window.

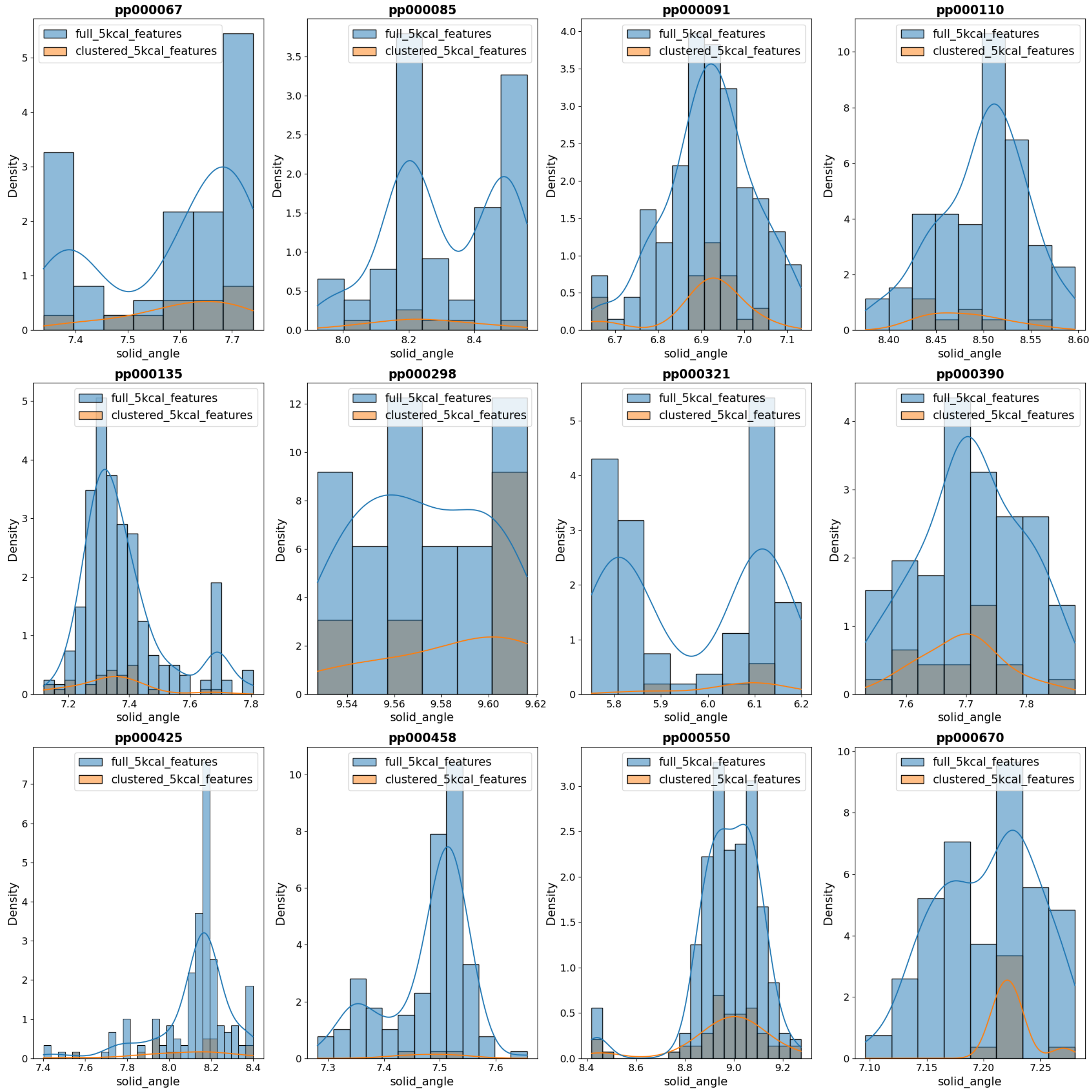

Figure S19: Comparison of equivalent cone angle across the full and clustered conformer ensembles (5 kcal mol^{-1}) energy window.

4 DFT-refined *vs* non-DFT-refined features

Data availability:

- Scripts used to generate plots in the below sections can be found in this publication's GitHub repository: https://github.com/SigmanGroup/BisphosphineConformerSelection. Feature values for each conformer for all 12 complexes can also be found as CSV files in the aforementioned repository.

Feature values were compared between complexes that were obtained with and without a DFT-level refinement at the PBE/def2-SVP level, labeled as "Set 1" and "Set 2", respectively in the main text. Features were obtained following a single point energy calculation at the PBE0/def2-TZVP level of theory. For more details see Section 1.

In the following Sections, conformer-weighted feature values (lowest energy conformer, Boltzmann weighted average, minimum feature value and maximum feature value) are compared between the two Sets. The percentage differences, absolute differences and co-linearity between the two Sets are used as metrics for assessment of comparison. Features are divided into steric, geometric and electronic categories as described in Section 1.3. Absolute feature values were compared for "frontier molecular orbitals" (HOMO and LUMO energy), "natural bond orbitals" (Cl, P and Pd NBO atomic charges), "NMR chemical shifts" (isotropic and anisotropic ^{31}P NMR chemical shifts). Boltzmann-weighted average feature values were determined from the uncorrected DFT electronic energy. Note that due to the conformer filtering and selection methods described above, the conformer thermodynamic ensembles are incomplete. Therefore, the Boltzmann-weighted averages obtained are only an approximation of the actual value. The Boltzmann-weighted averages were determined according to Equation 3. Maximum and minimum electronic feature values may be harder to interpret compared to steric and geometric feature values, but have been included for completeness.

$$\langle x \rangle_{Boltzmann} = \frac{\sum_{i=1}^{N} x_i \exp(-E_i/k_B T)}{\sum_{i=1}^{N} \exp(E_i/k_B T)} \qquad (3)$$

4.1 Lowest energy conformer features

The bar graphs at the top of Figure S20 show that a number of steric and geometric features have an absolute average percent difference within 5% indicating a general low error value between the DFT-refined structures and geometries obtained directly from the conformer search in *CREST*. A notable exception to this the molecular volume (labelled "Volume"). This may be explained by the volume calculation being derived directly from the DFT single point energy calculation (*vide infra*). In the case of the electronic features, a greater number of features ≥5% absolute average percent difference (*e.g.*, LUMO energy, Pd NBO charge and anisotropic ^{31}P NMR chemical shift). These results, especially in the case of the Pd NBO charge, may indicate that the electronic structure is being insufficiently described. We suspect this is due to a mismatch in the electronic structure theories used in the optimization/refinement of the complexes (*i.e.*, between xTB and DFT levels).

When evaluating the collinearity between the two sets of features, steric features in general show good linear relationships between the DFT-refined and non-DFT-refined Sets (Figure S21. Exceptions to this are the volume terms, likely due to reasons indicated above. Geometric features, with the exception of Pd–Cl bond distances show good co-linearity (Figure S22). In general, electronic features show decreased co-linearity between the two Sets of calculations. Again, we suspect these are a result of the mismatch between two methods of electronic structure theory used to optimize the geometry of the complexes.

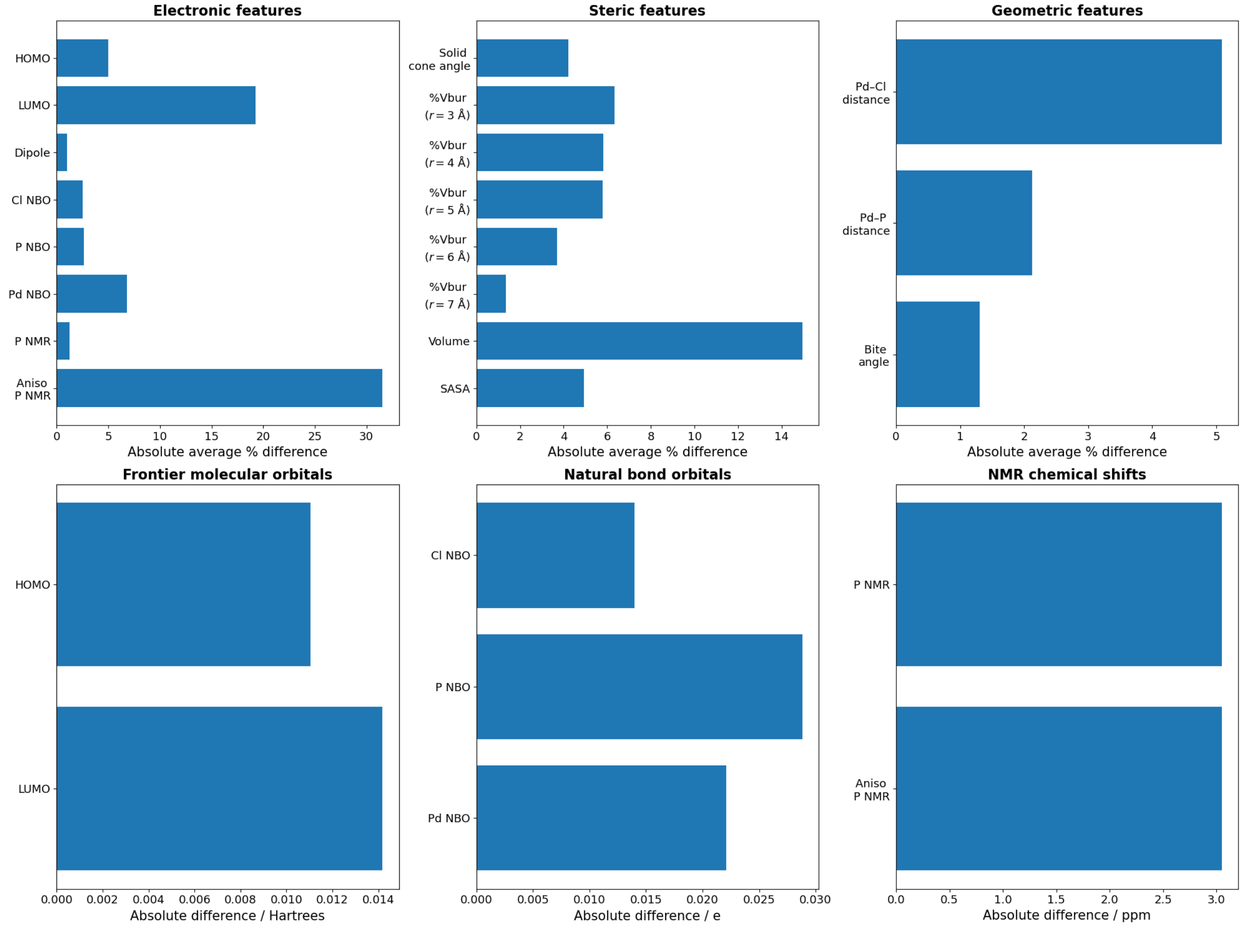

Figure S20: Percentage and absolute difference in feature values between the DFT-refined and non-DFT-refined calculation sets (lowest energy conformer values).

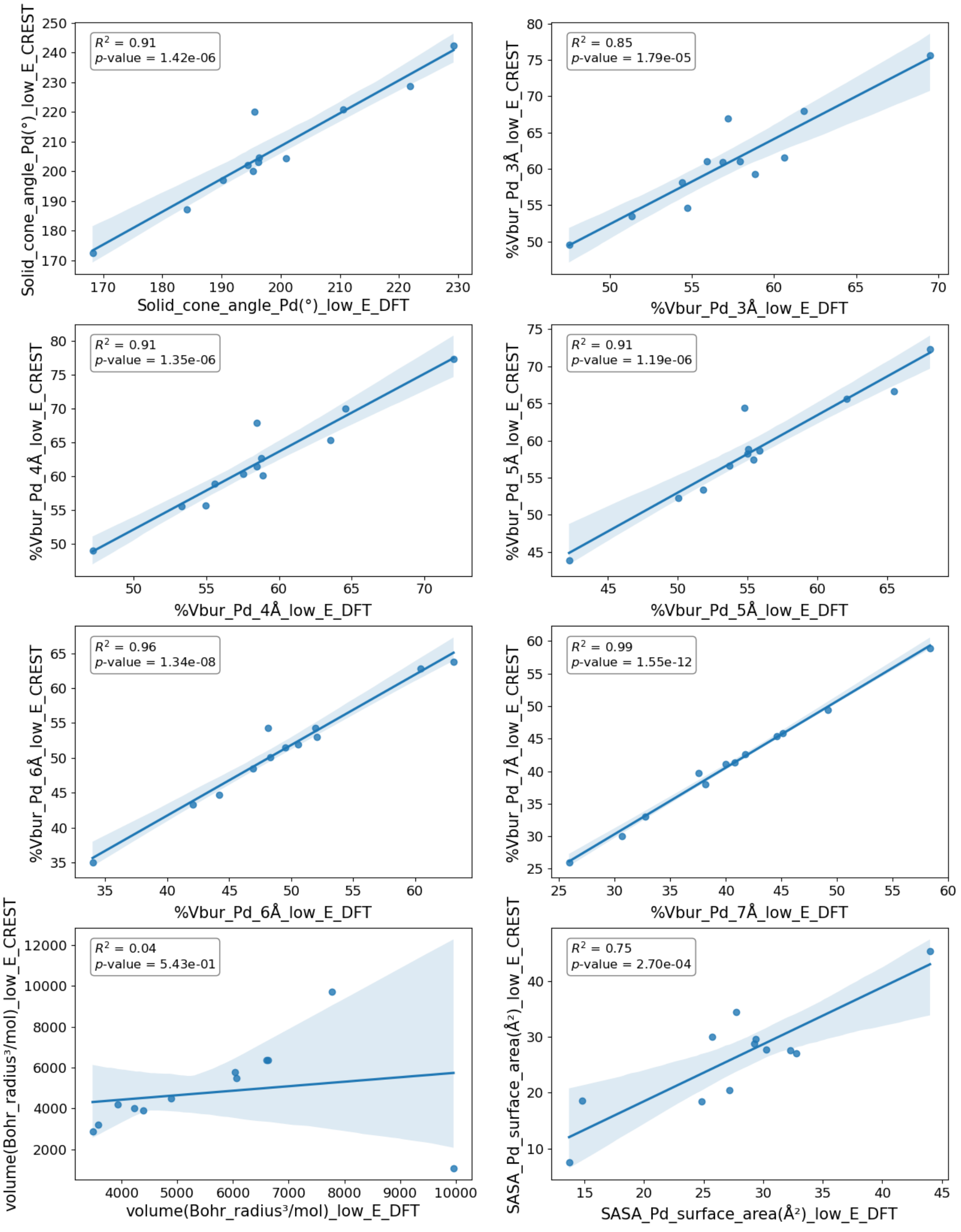

Figure S21: Linear relationships between steric feature values from the DFT-refined and non-DFT-refined calculation sets (lowest energy conformer values).

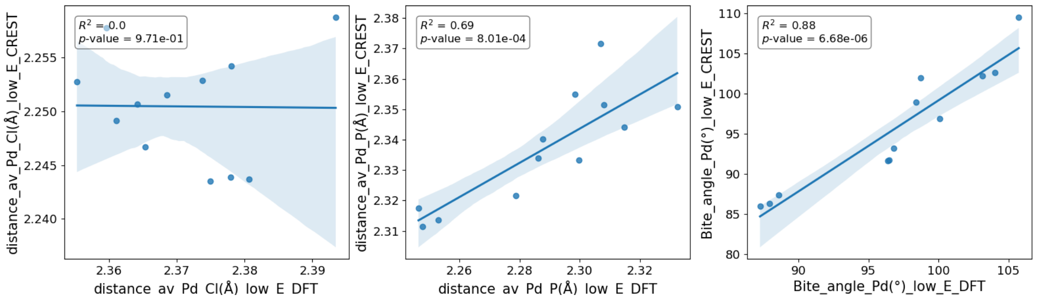

Figure S22: Linear relationships between geometric feature values from the DFT-refined and non-DFT-refined calculation sets (lowest energy conformer values).

4.2 Boltzmann weighted averaged features

The bar graphs given in Figure S24 show similar trends to the comparison between feature values for the lowest energy conformer in Section 4.1. Interestingly the error in molecular volume decreased here compared to the lowest energy conformer.

In the co-linearity checks between two calculation sets (Figures S25, S26 and S27) show similar trends to regression plots in Section 4.1.

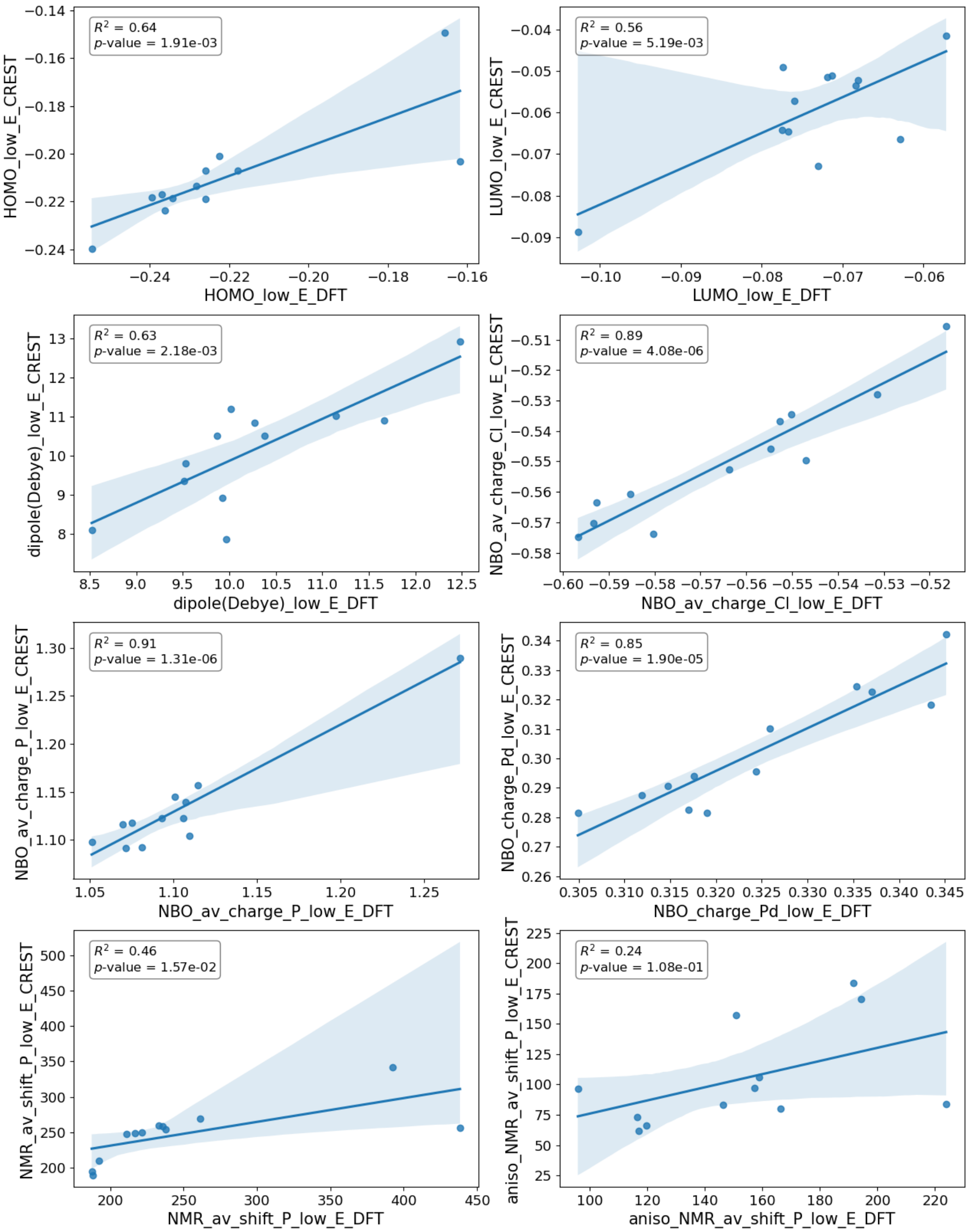

Figure S23: Linear relationships between electronic feature values from the DFT-refined and non-DFT-refined calculation sets (lowest energy conformer values).

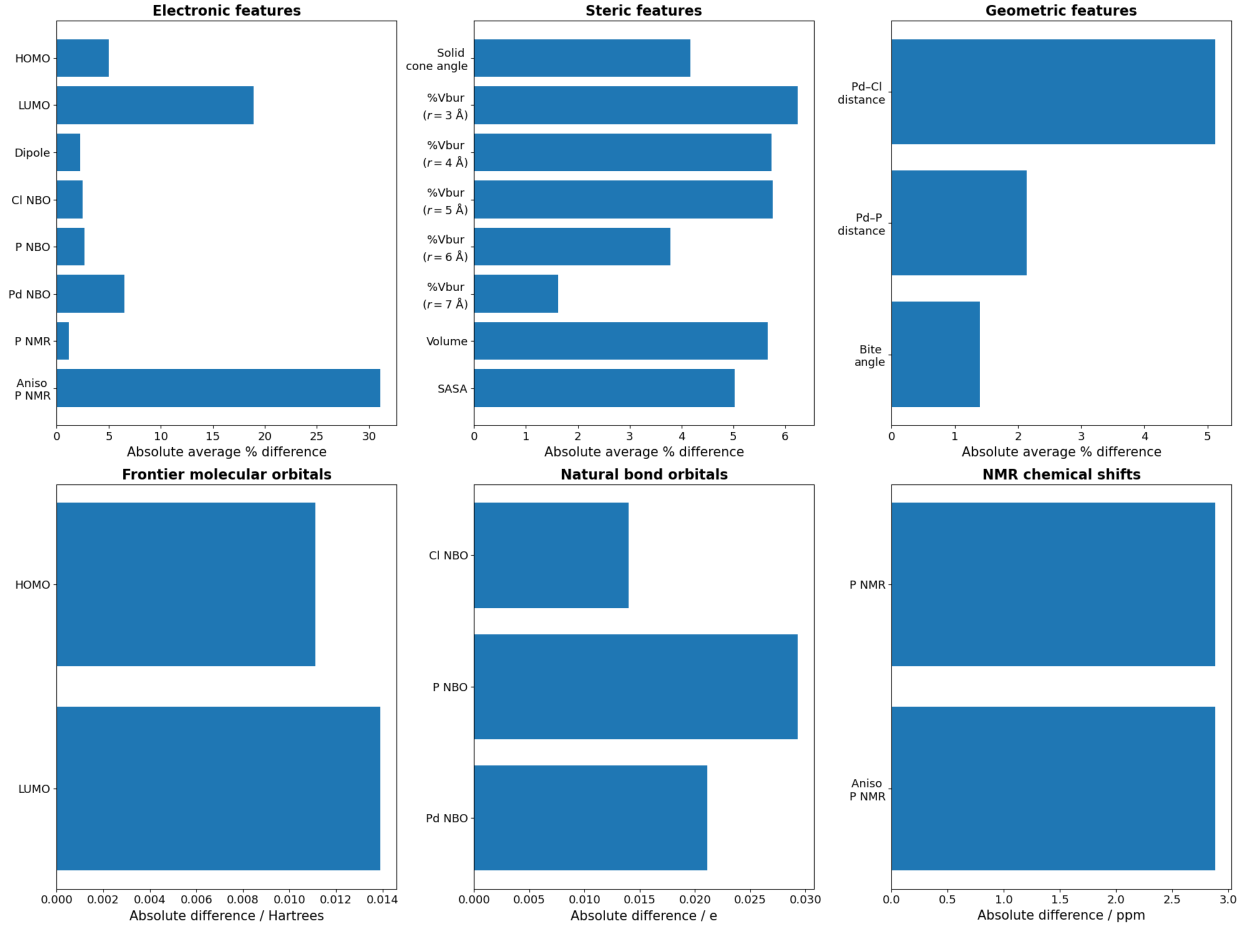

Figure S24: Percentage and absolute difference in feature values between the DFT-refined and non-DFT-refined calculation sets (Boltzmann-weighted averaged values).

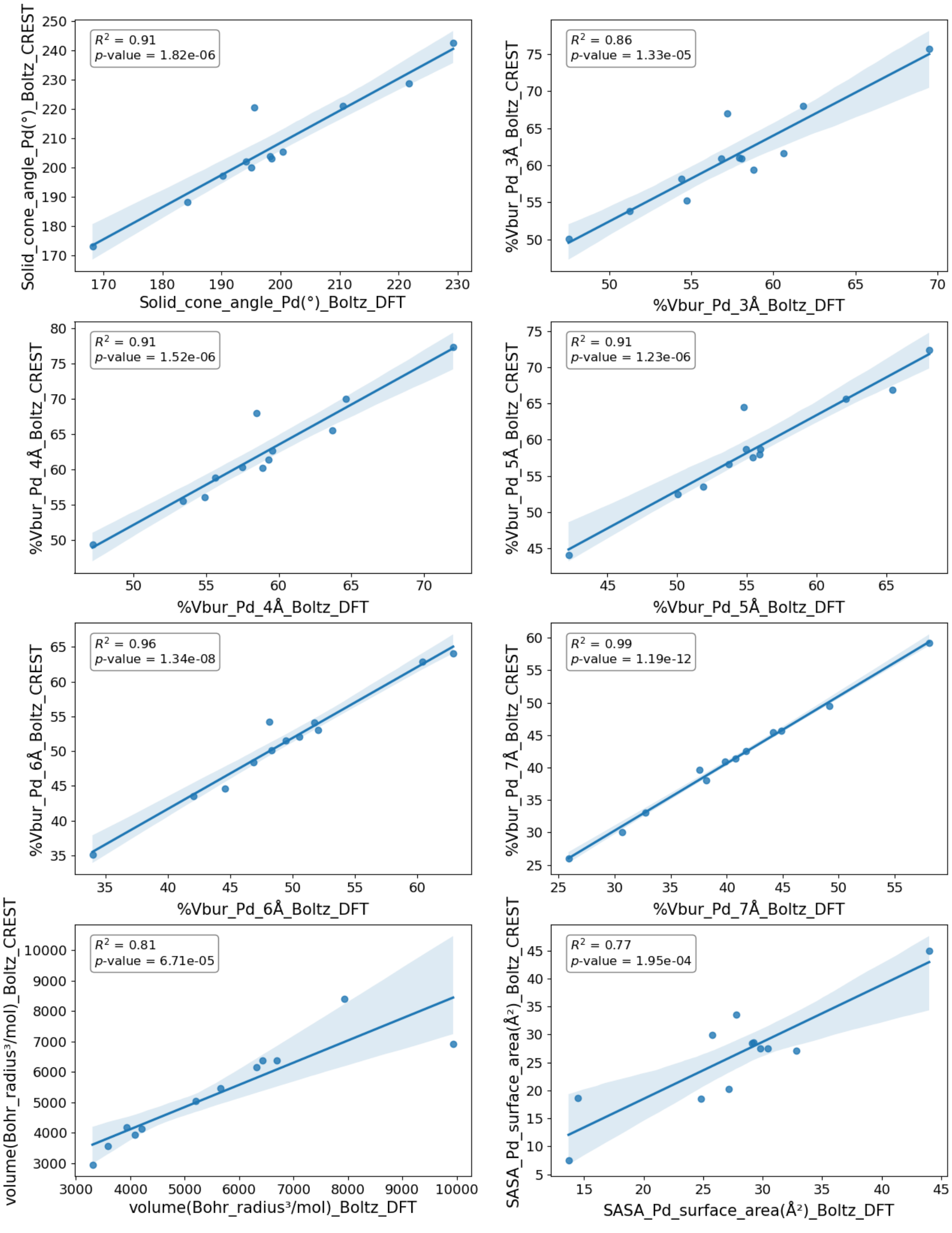

Figure S25: Linear relationships between steric feature values from the DFT-refined and non-DFT-refined calculation sets (Boltzmann-weighted averaged values).

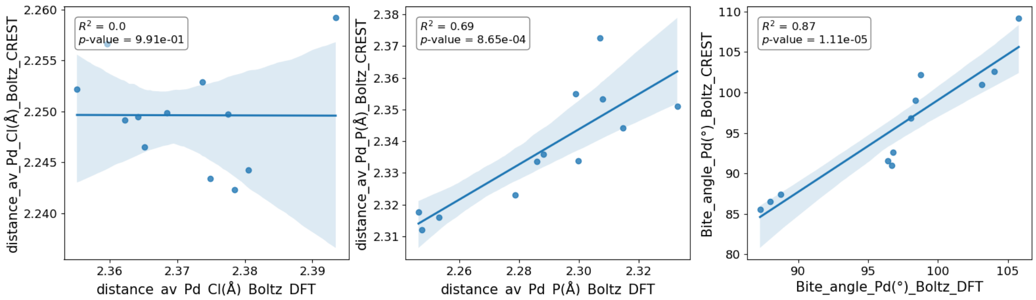

Figure S26: Linear relationships between geometric feature values from the DFT-refined and non-DFT-refined calculation sets (Boltzmann-weighted averaged values).

4.3 Minimum and maximum conformer feature values

In the cases of maximum and minimum conformer feature values, the observed trends between the DFT-refined and non-DFT-refined sets are similar. The Figures showing the correlations are provided below:

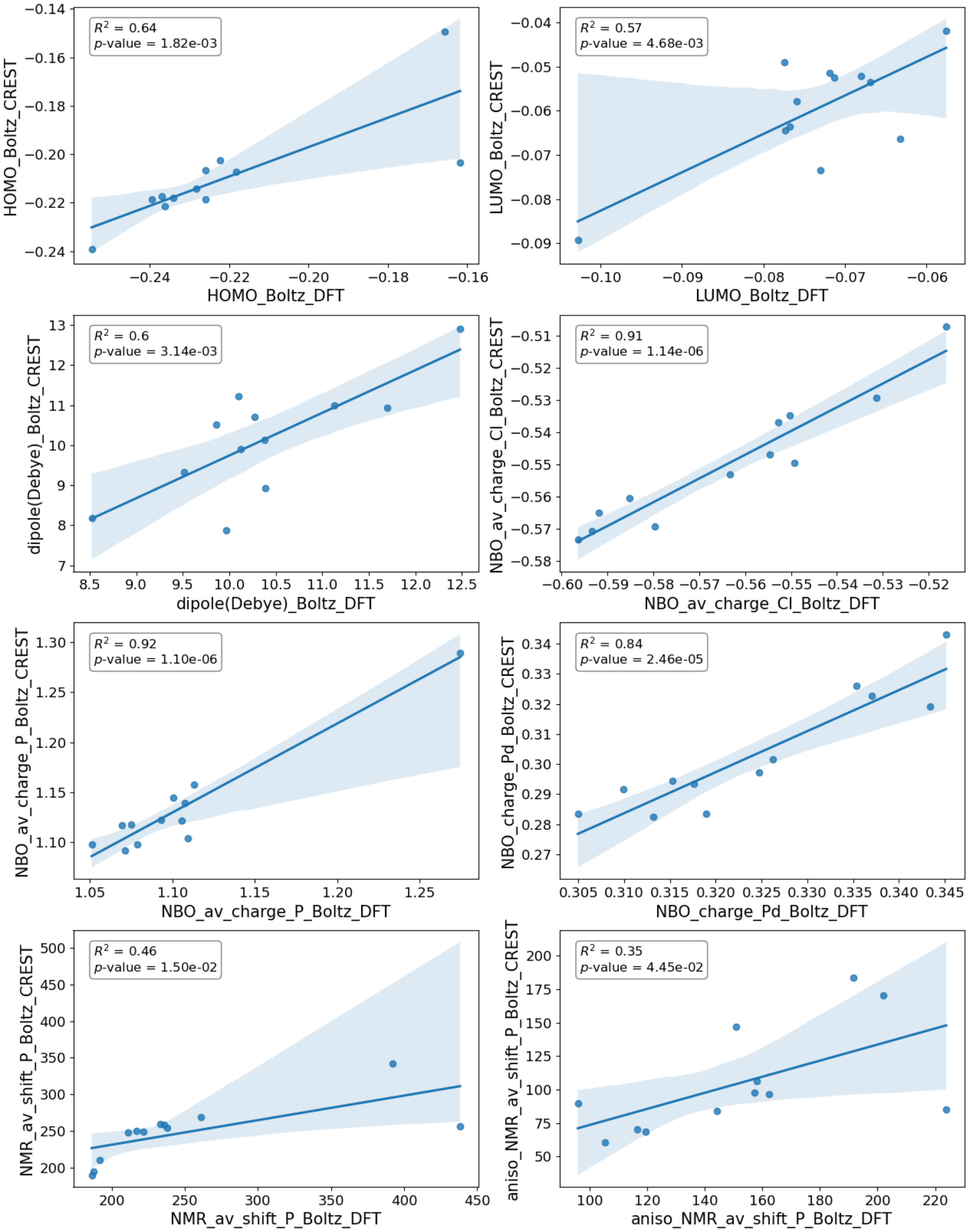

Figure S27: Linear relationships between electronic feature values from the DFT-refined and non-DFT-refined calculation sets (Boltzmann-weighted averaged values).

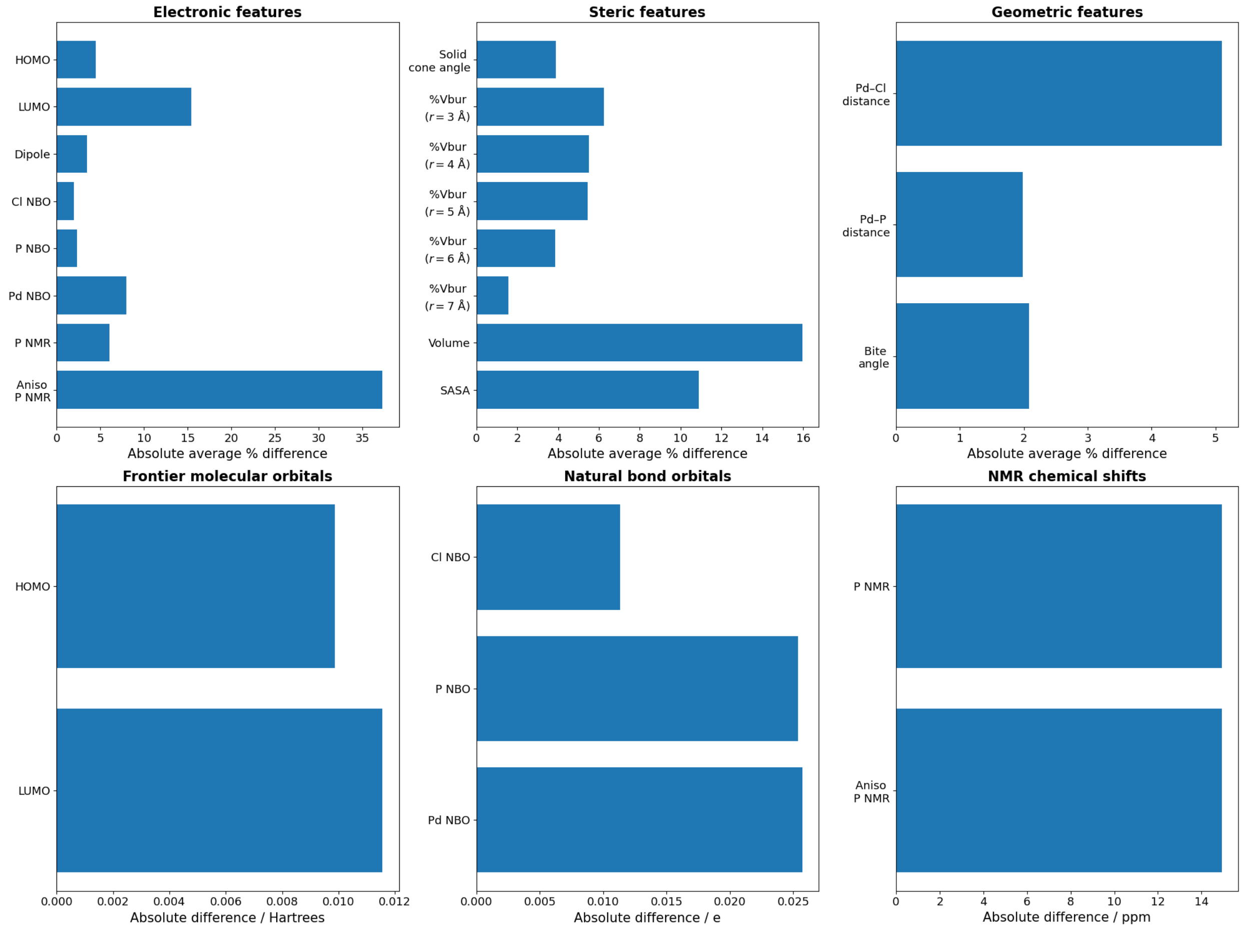

Figure S28: Percentage and absolute difference in feature values between the DFT-refined and non-DFT-refined calculation sets (Minimum feature values).

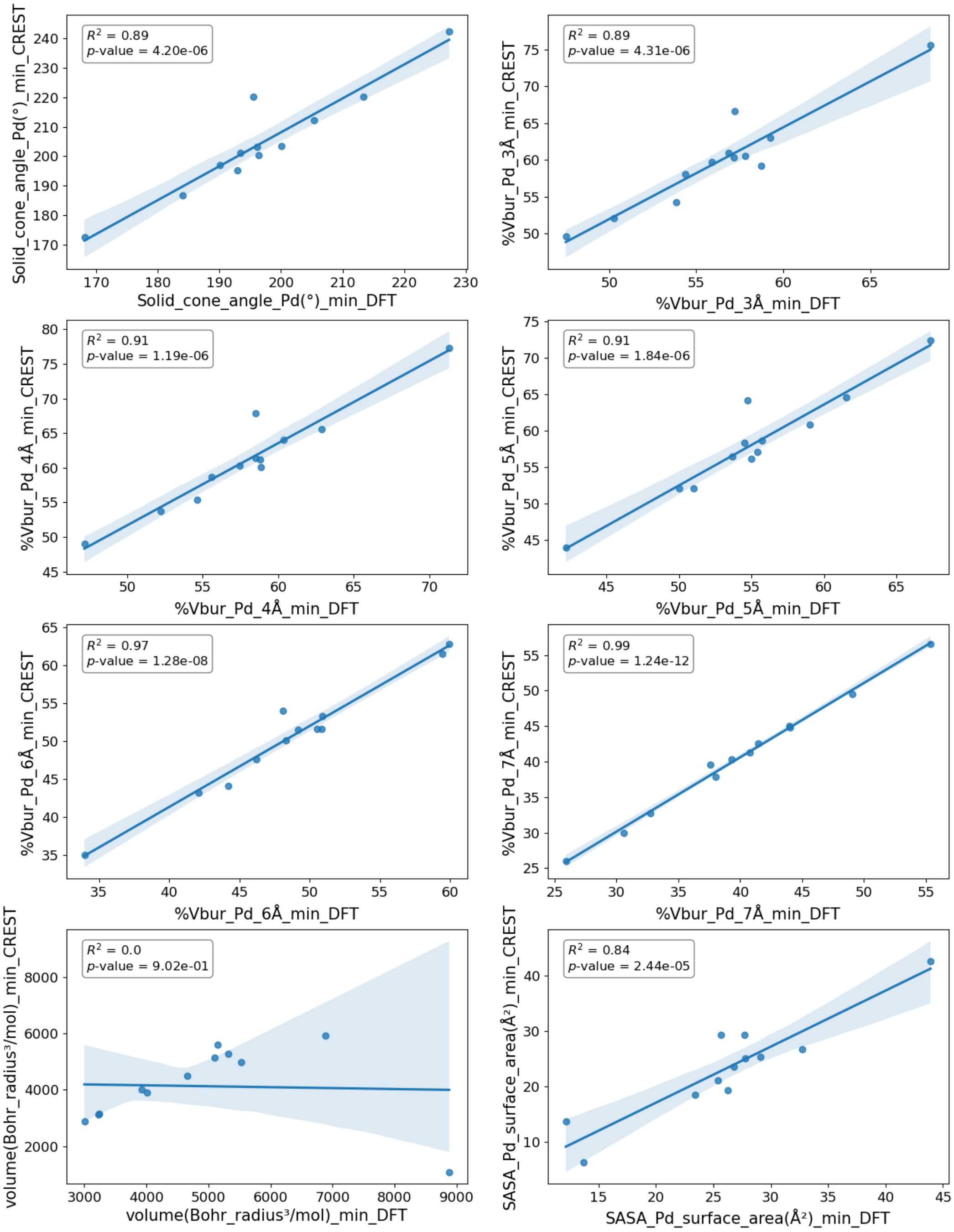

Figure S29: Linear relationships between steric feature values from the DFT-refined and non-DFT-refined calculation sets (Minimum feature values).

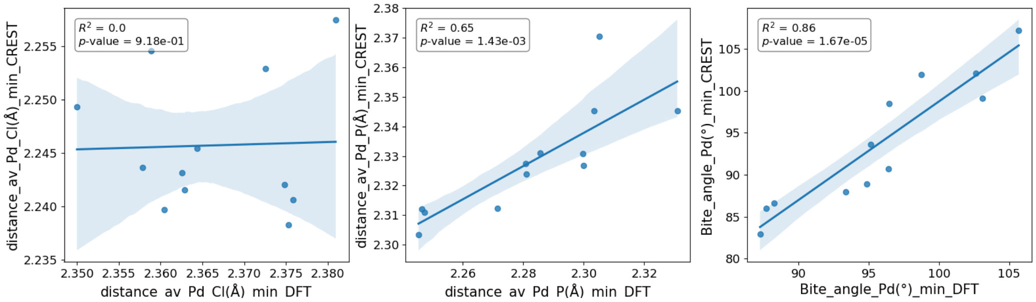

Figure S30: Linear relationships between geometric feature values from the DFT-refined and non-DFT-refined calculation sets (Minimum feature values).

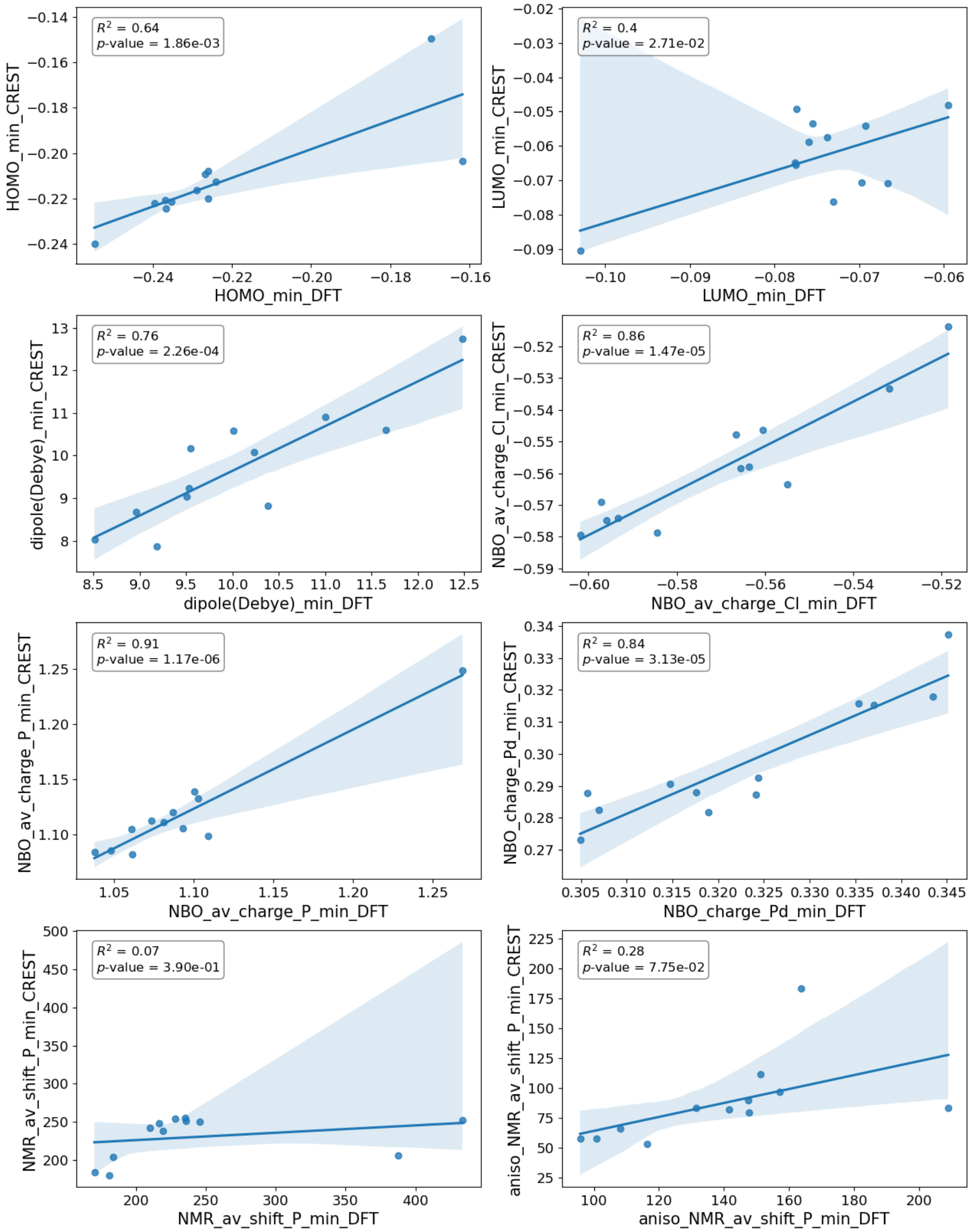

Figure S31: Linear relationships between electronic feature values from the DFT-refined and non-DFT-refined calculation sets (Minimum feature values).

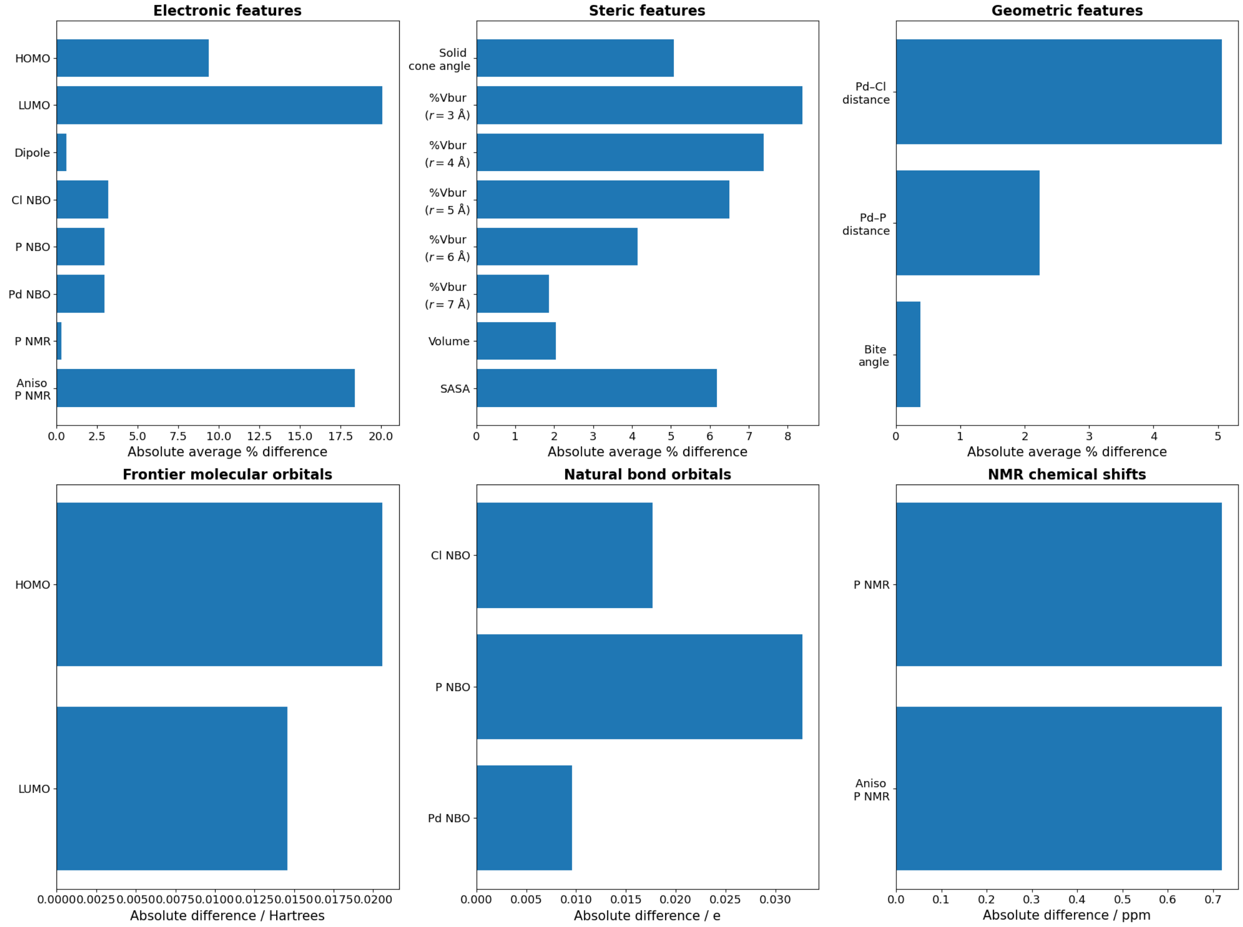

Figure S32: Percentage and absolute difference in feature values between the DFT-refined and non-DFT-refined calculation sets (Maximum feature values).

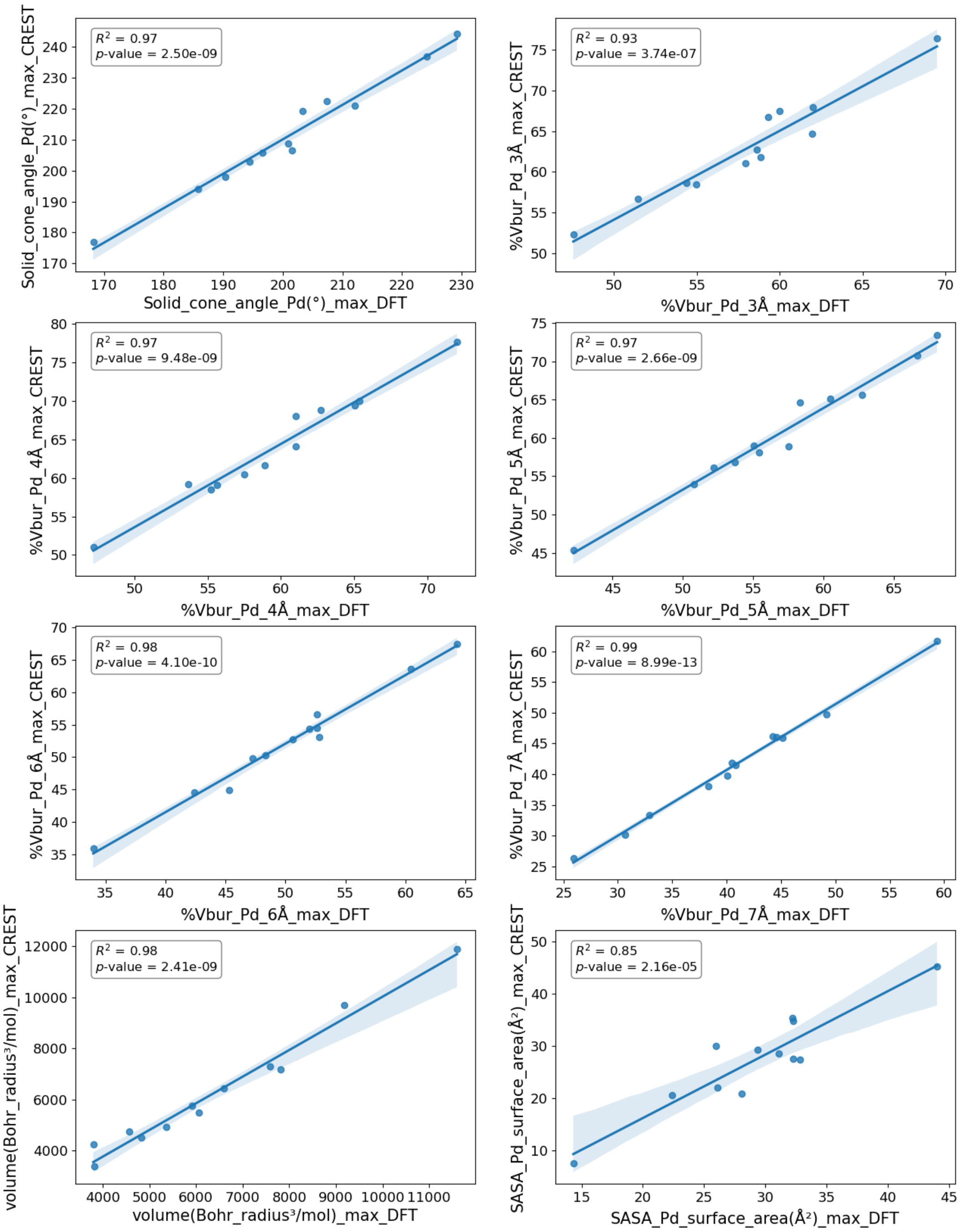

Figure S33: Linear relationships between steric feature values from the DFT-refined and non-DFT-refined calculation sets (Maximum feature values).

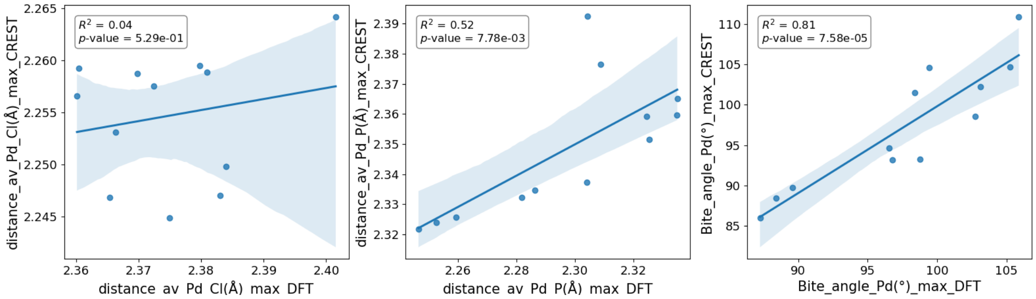

Figure S34: Linear relationships between geometric feature values from the DFT-refined and non-DFT-refined calculation sets (Maximum feature values).

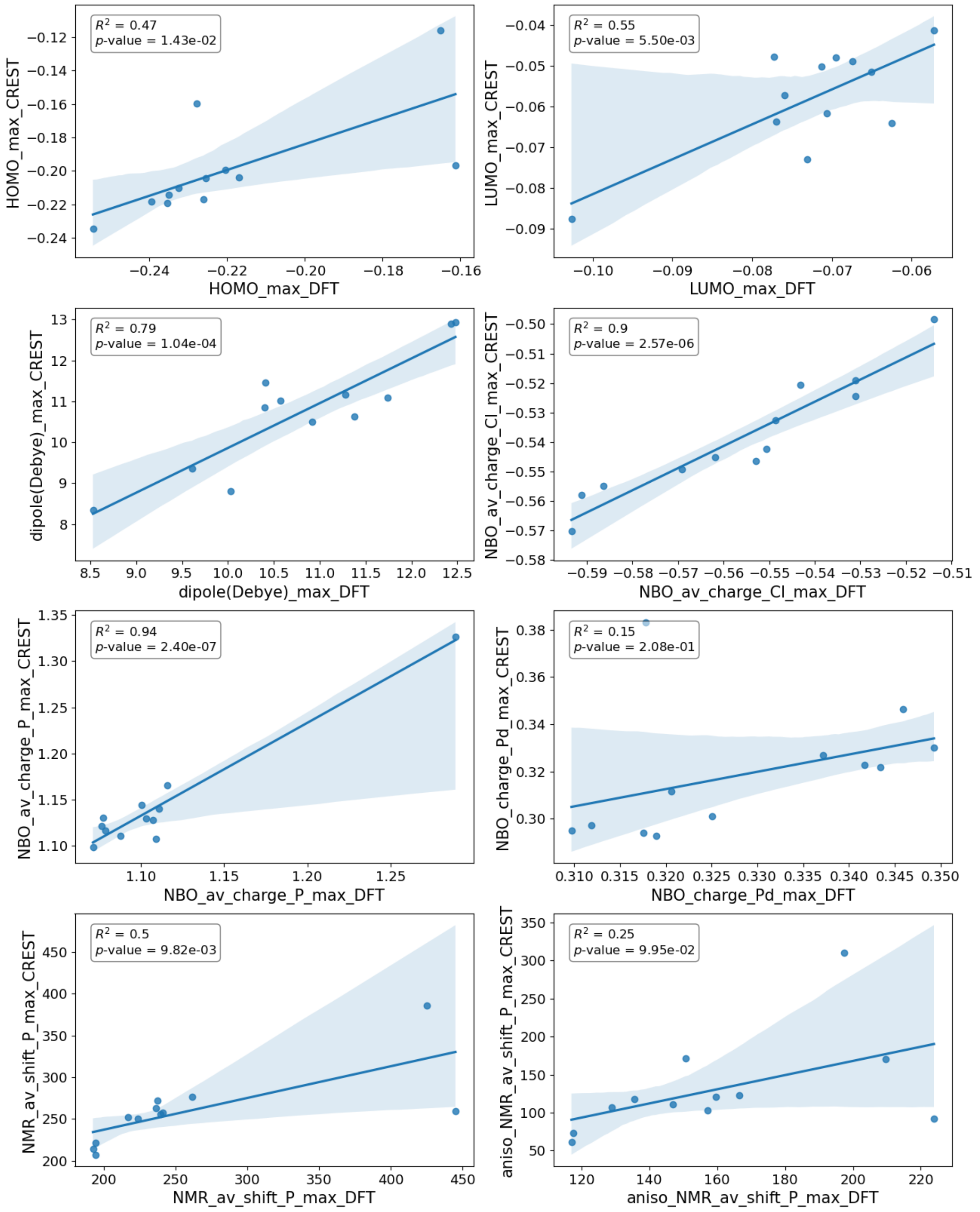

Figure S35: Linear relationships between electronic feature values from the DFT-refined and non-DFT-refined calculation sets (Maximum feature values).

5 Conformer selection

Data availability:

- Scripts used to generate plots in the below sections as well as determination of features can be found in this publication's GitHub repository: https://github.com/SigmanGroup/BisphosphineConformerSelection.

5.1 Selection of conformers with equidistant feature values

In order to maximize the spread of selected conformers for a subsequent DFT-level geometry refinement and single-point energy calculation, we envisioned that an equidistant selection based on a given feature value would achieve this. The equidistant selection works by taking an evenly spaced selection of feature values using the `linspace` function in the NumPy Python library from a sorted Pandas dataframe. See the below code snippet for an example of how this works in practice (full code can be found in `./functions/utils.py` in the GitHub repository). `df` is the specified Pandas dataframe containing all the GFN2-xTB energies and steric/geometric feature values. This is then sorted in ascending order by the selected feature (`column`). The equidistant indices of the sorted values are determined (number in the selection is defined by the user as `y`) and are returned as `selected_values`. Equivalent code is used to select the lowest energy GFN2-xTB conformer where `y` is set to 1.

```
sorted_df = df.sort_values(column)
indices = np.linspace(0, len(sorted_df) - 1, y, dtype=int)
selected_values = sorted_df.iloc[indices]
```

Features examined for selection were the GFN2-xTB energy, bite angle, equivalent cone angle and a combination of bite angle and equivalent cone angle (determined by multiplication of the two feature values).

5.2 Selection from GFN2-xTB energies

5.2.1 Generation of equidistant conformers

In the following series of plots (Figures S36 - S47, one for each complex used in this study), conformers from the clustered ensemble (5 kcal mol^{-1} window) are selected based on equidistant GFN2-xTB energies. A total of 10 conformers were selected (represented at the top left as $f(E)$, according to Equation 4). The effect of this selection is also shown on geometric/steric feature values in the ensemble as scatter plots and histograms (bottom six plots).

$$f(E) = \frac{1}{\exp(E/k_B T)} \tag{4}$$

5.3 Selection from steric and geometric features

5.3.1 Generation of equidistant conformers

In the following series of plots (Figures S48 - S59, one for each complex used in this study), conformers from the clustered ensemble (5 kcal mol^{-1} window) are selected based on three steric and/or geometric features. These are bite angle, percent buried volume ($r = 3.5\mathring{A}$) and a combination of bite angle and percent buried volume (determined by multiplication of the two feature values). A toal of 10 conformers were selected, which are represented as the three histograms on the top row of the plots and as scatter plots on the middle row. The effect of this selection on the conformer energies (plotted as $f(E)$, see Equation 4) is also shown on the bottom row.

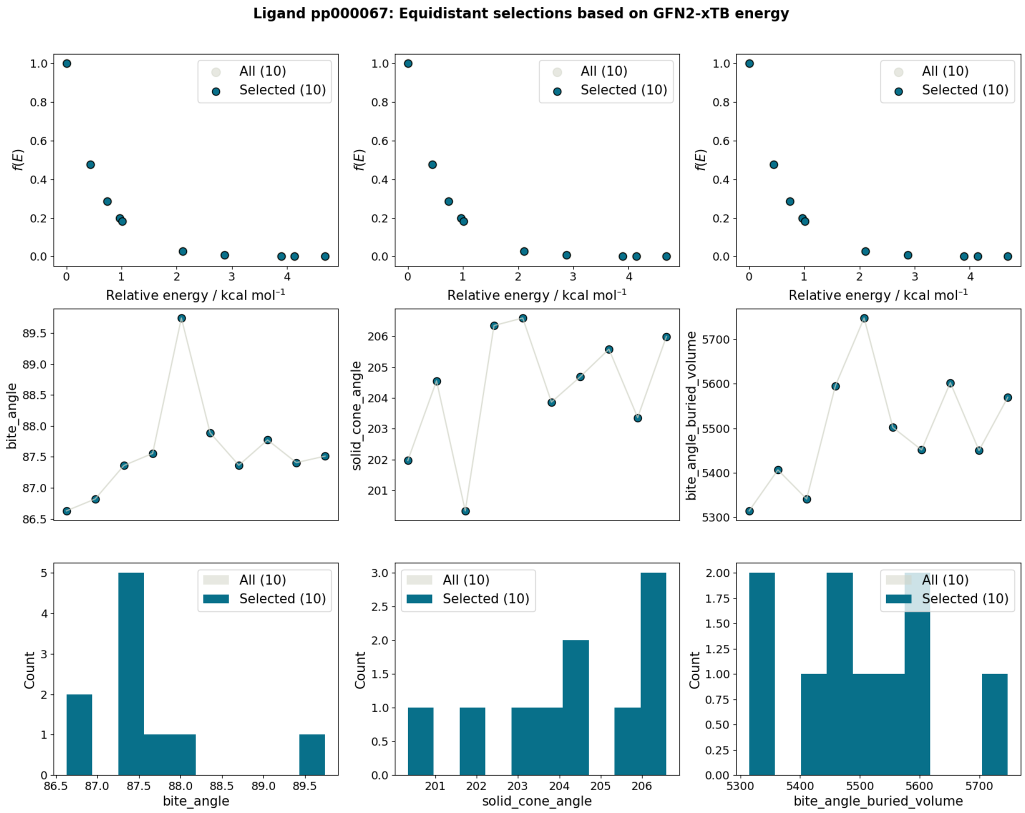

Figure S36: Equidistant selection of conformers (10 total) for complex **pp000067** based on GFN2-xTB energy.

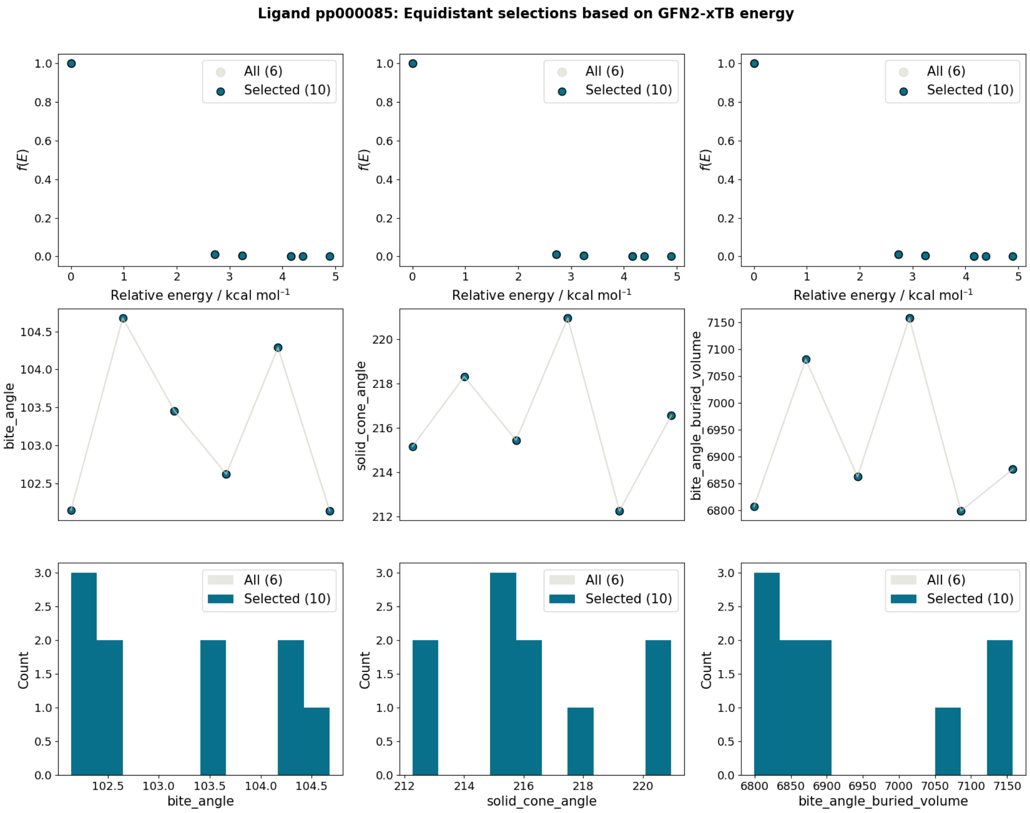

Figure S37: Equidistant selection of conformers (10 total) for complex **pp000085** based on GFN2-xTB energy.

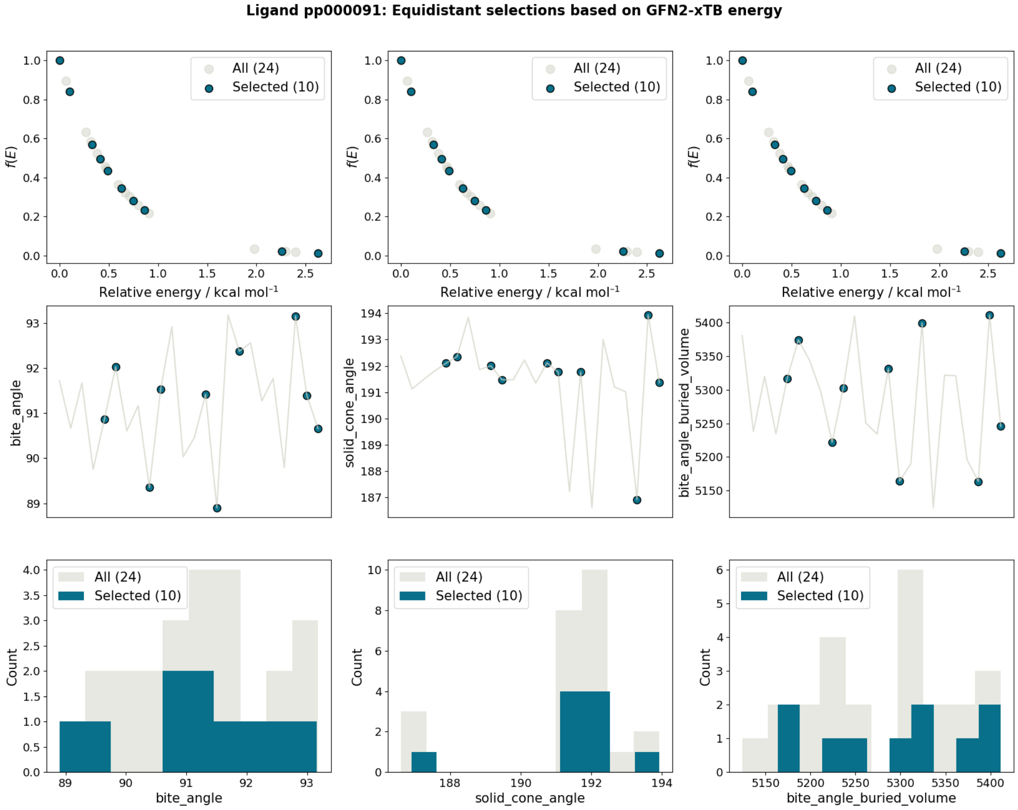

Figure S38: Equidistant selection of conformers (10 total) for complex **pp000091** based on GFN2-xTB energy.

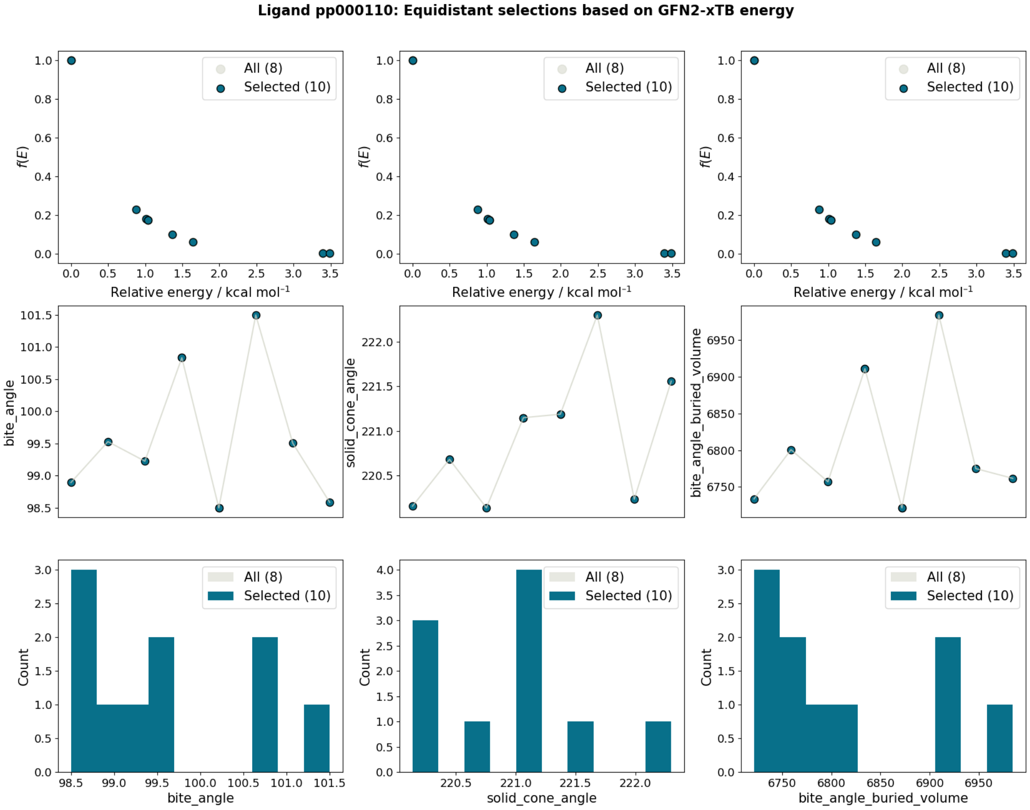

Figure S39: Equidistant selection of conformers (10 total) for complex **pp000110** based on GFN2-xTB energy.

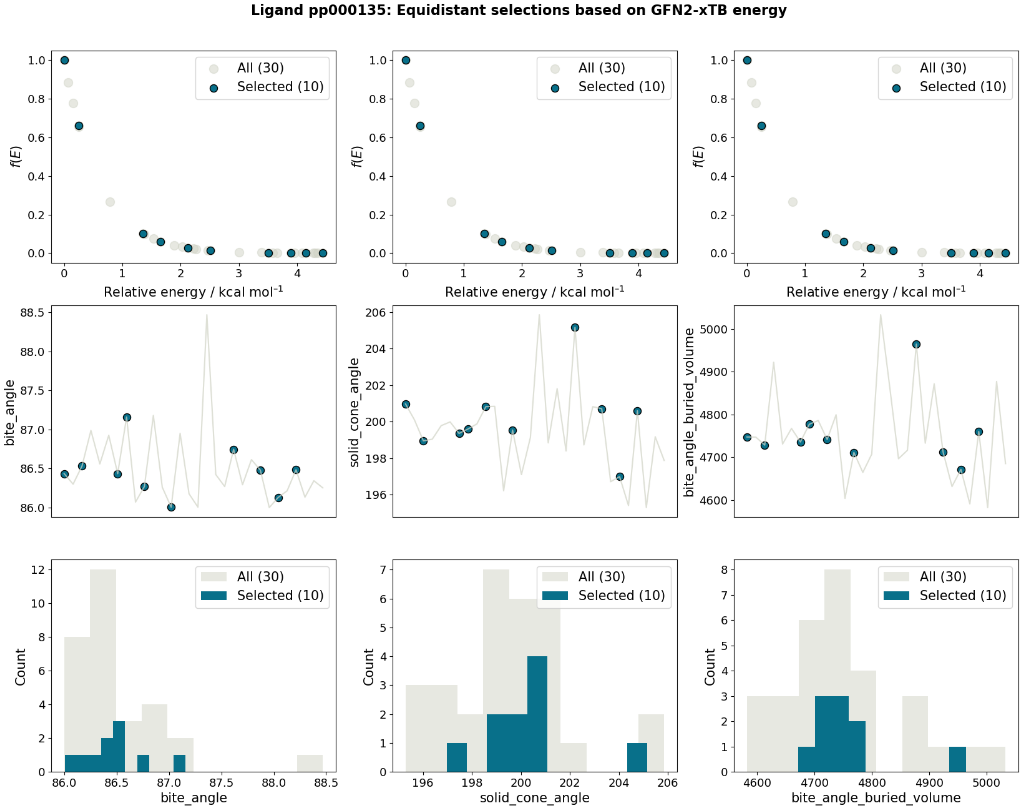

Figure S40: Equidistant selection of conformers (10 total) for complex **pp000135** based on GFN2-xTB energy.

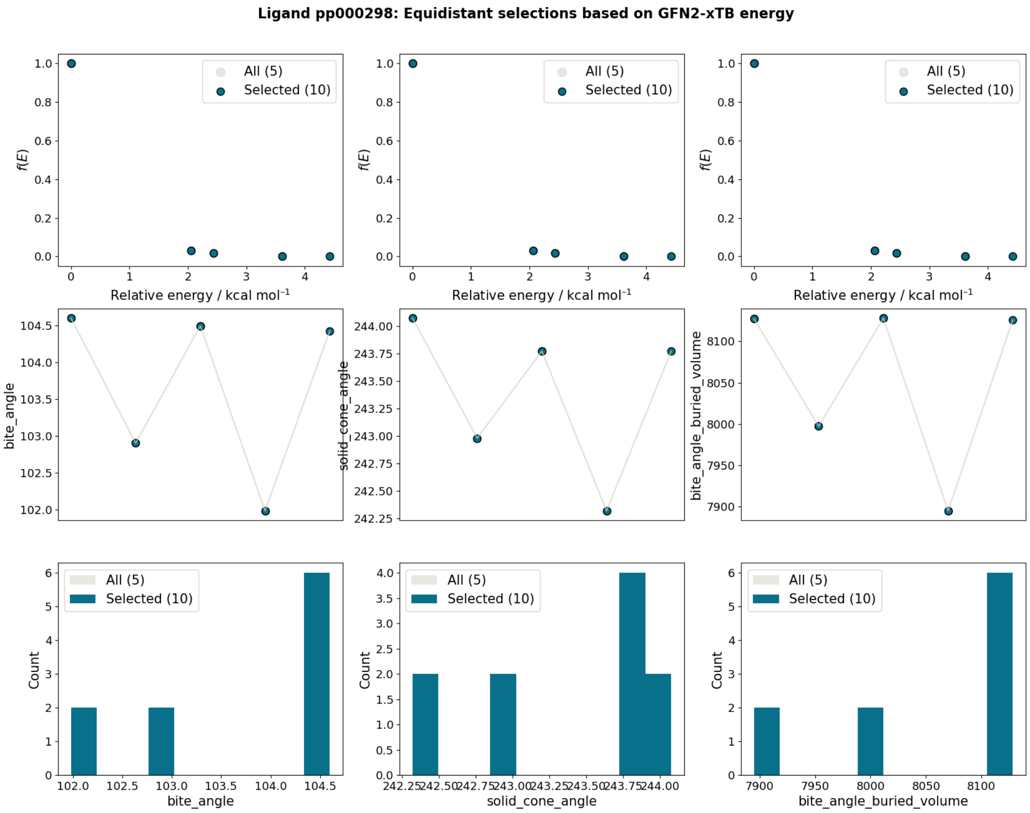

Figure S41: Equidistant selection of conformers (10 total) for complex **pp000298** based on GFN2-xTB energy.

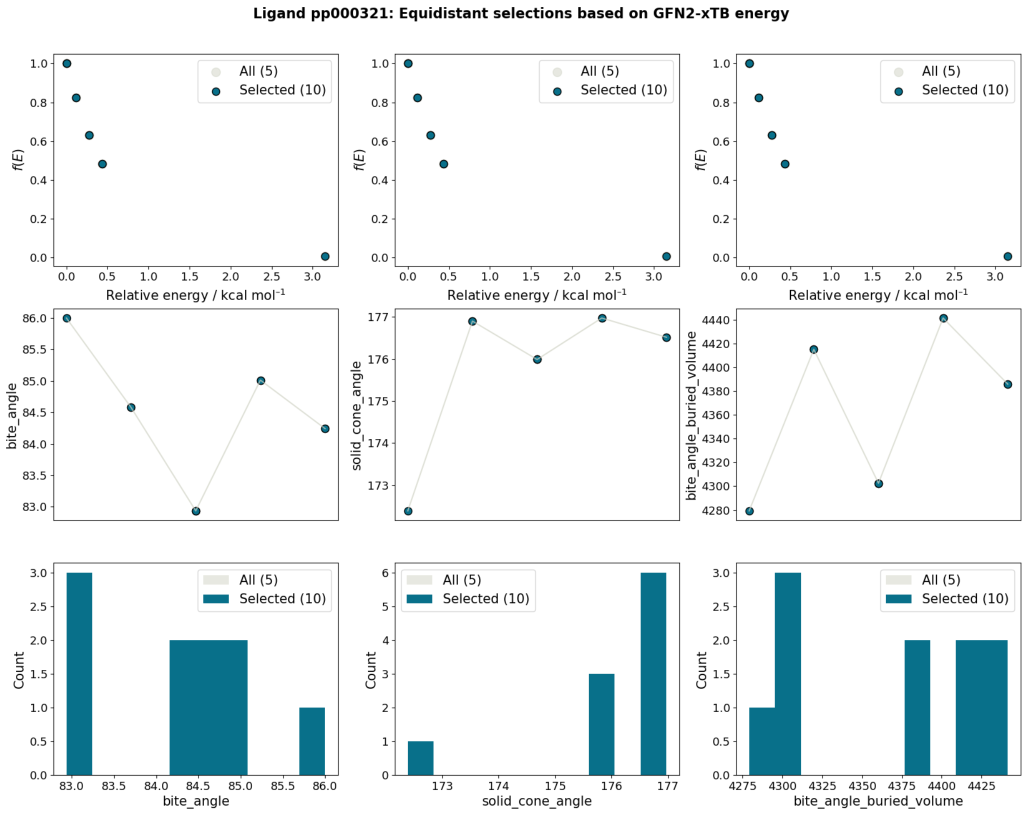

Figure S42: Equidistant selection of conformers (10 total) for complex **pp000321** based on GFN2-xTB energy.

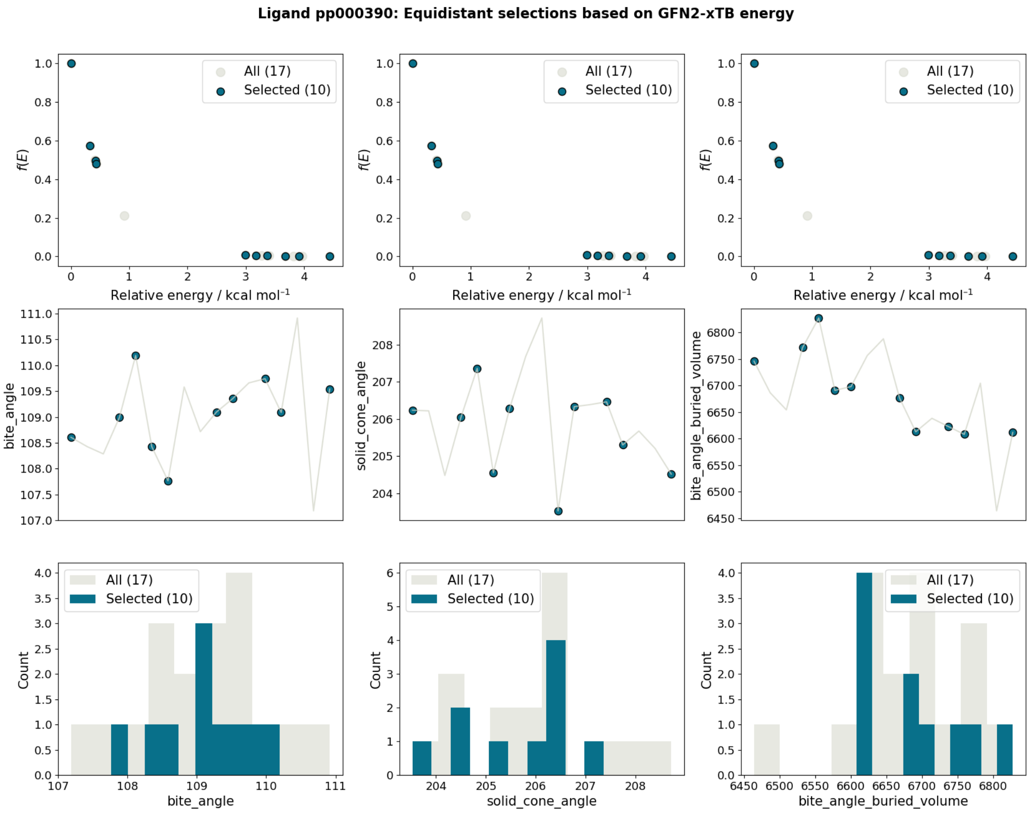

Figure S43: Equidistant selection of conformers (10 total) for complex **pp000390** based on GFN2-xTB energy.

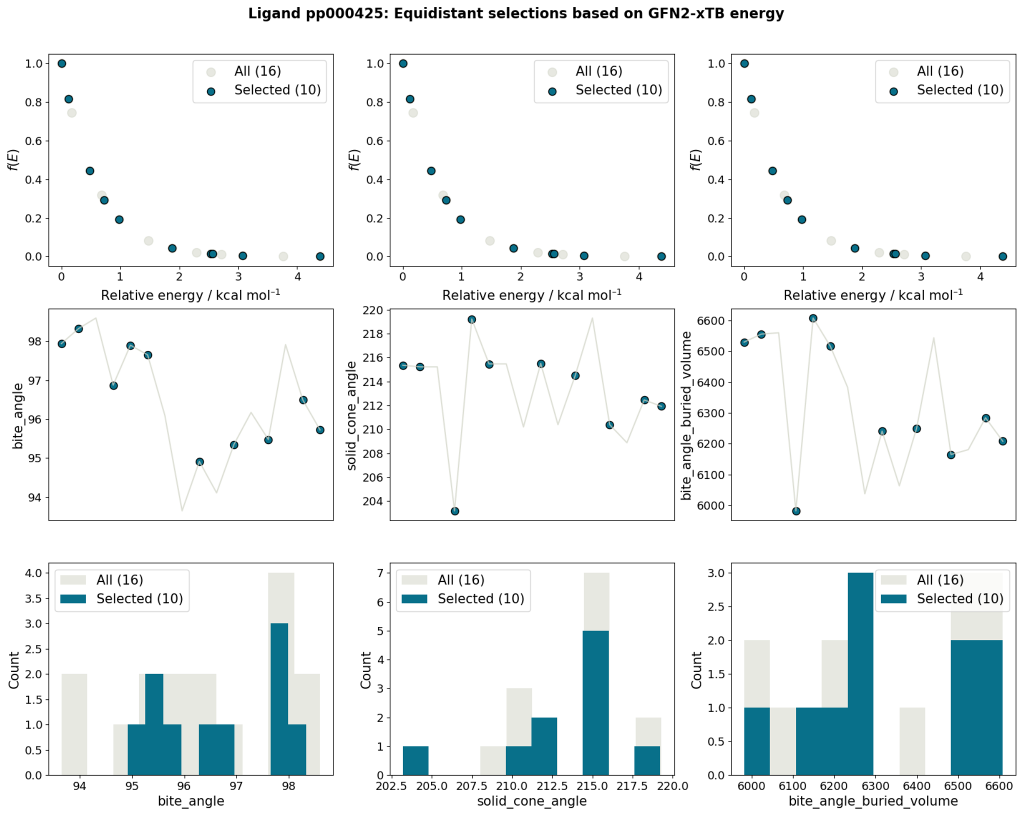

Figure S44: Equidistant selection of conformers (10 total) for complex **pp000425** based on GFN2-xTB energy.

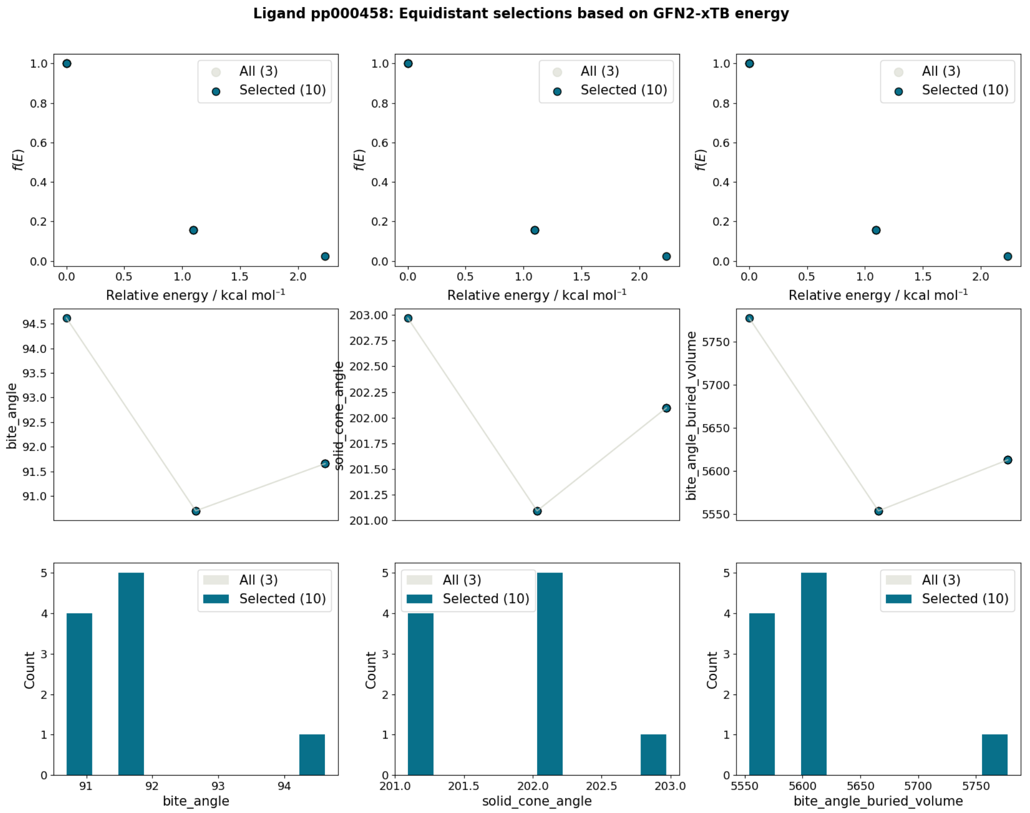

Figure S45: Equidistant selection of conformers (10 total) for complex **pp000458** based on GFN2-xTB energy.

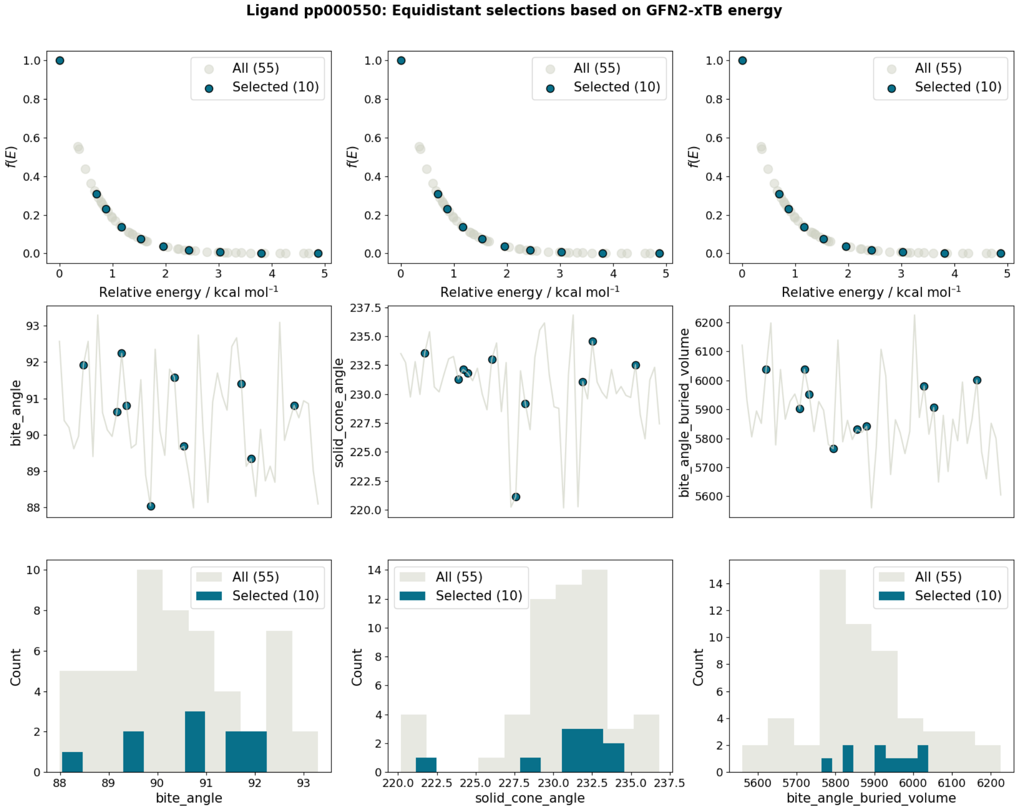

Figure S46: Equidistant selection of conformers (10 total) for complex **pp000550** based on GFN2-xTB energy.

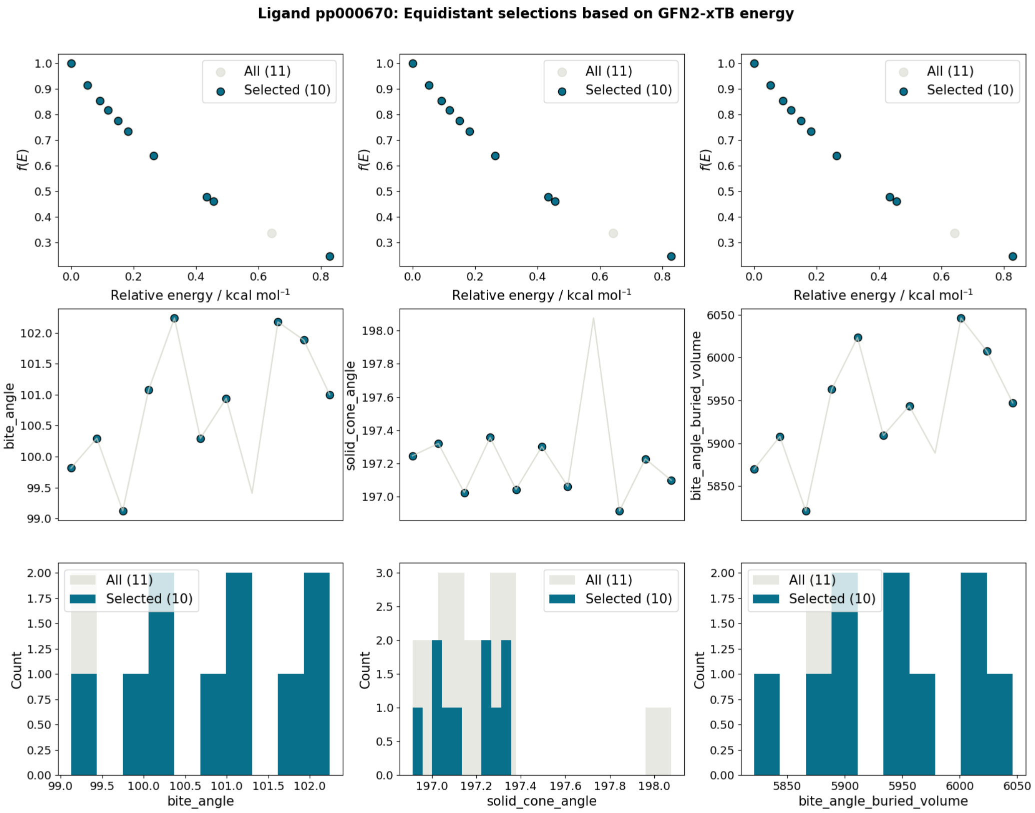

Figure S47: Equidistant selection of conformers (10 total) for complex **pp000670** based on GFN2-xTB energy.

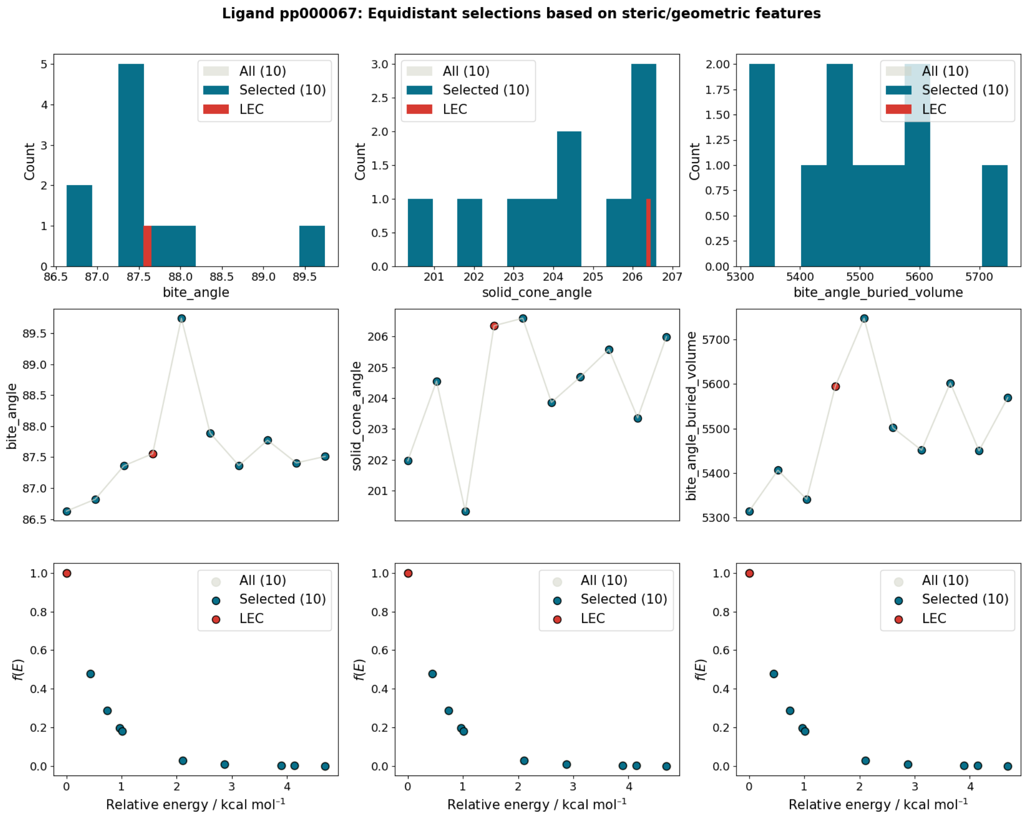

Figure S48: Equidistant selection of conformers (10 total) for complex **pp000067** based on steric and/or geometric features.

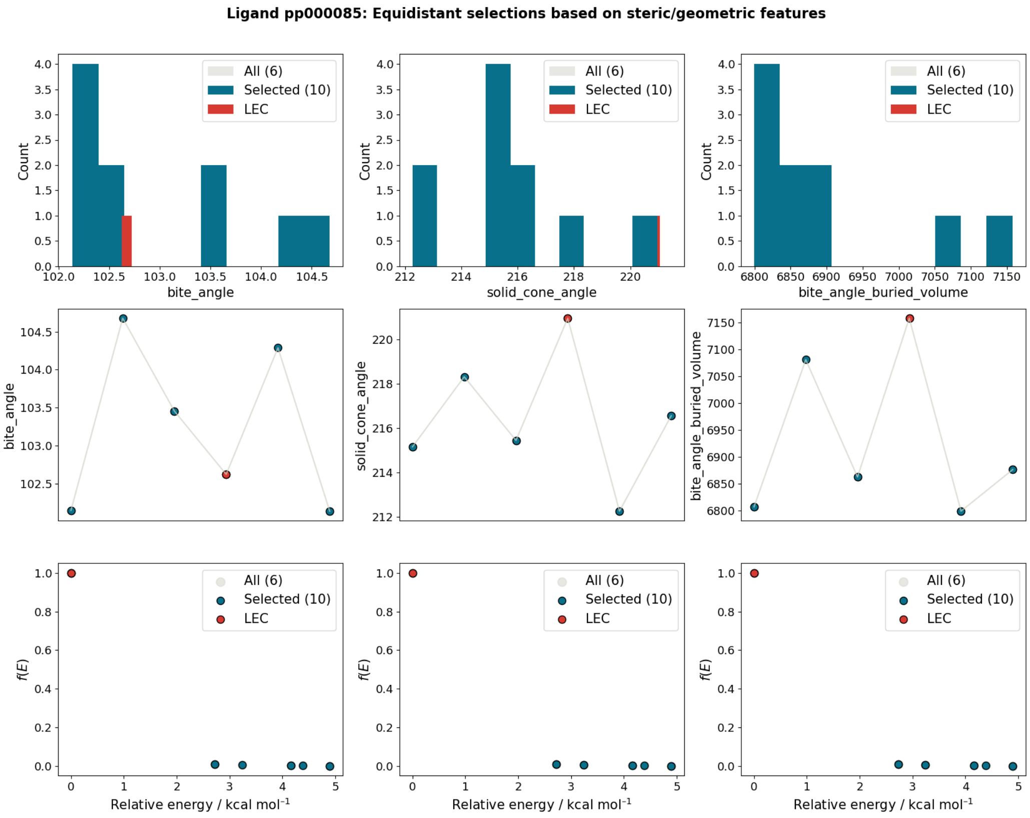

Figure S49: Equidistant selection of conformers (10 total) for complex **pp000085** based on steric and/or geometric features.

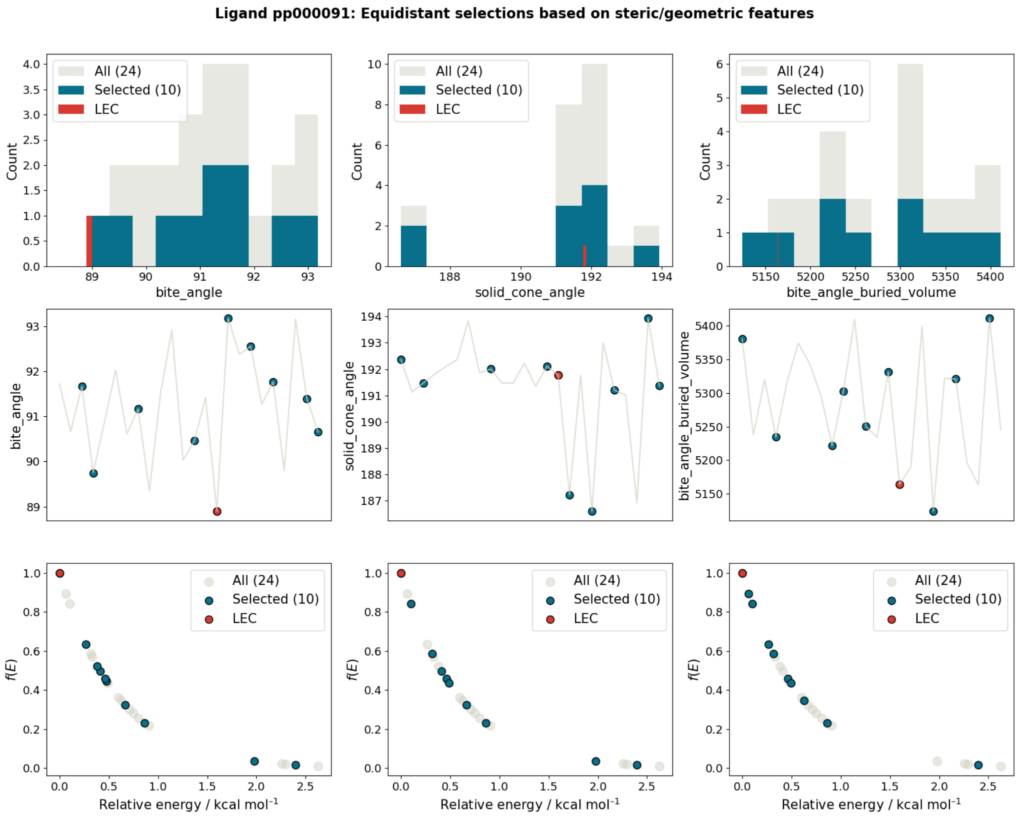

Figure S50: Equidistant selection of conformers (10 total) for complex **pp000091** based on steric and/or geometric features.

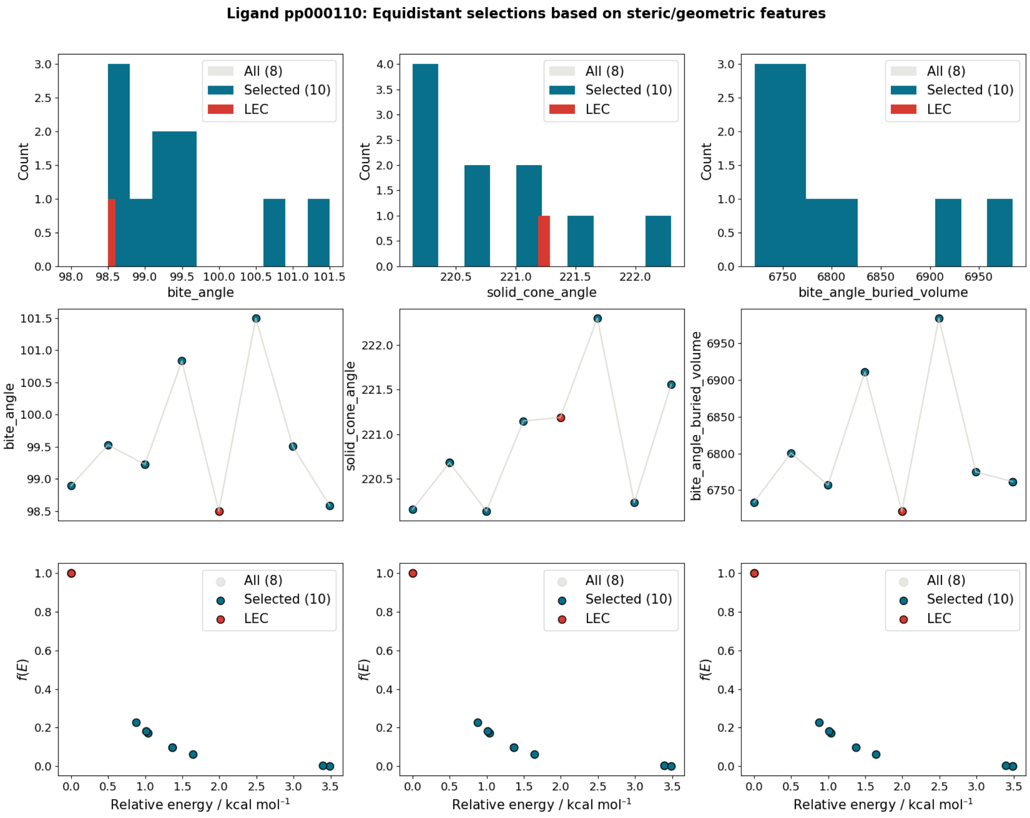

Figure S51: Equidistant selection of conformers (10 total) for complex **pp000110** based on steric and/or geometric features.

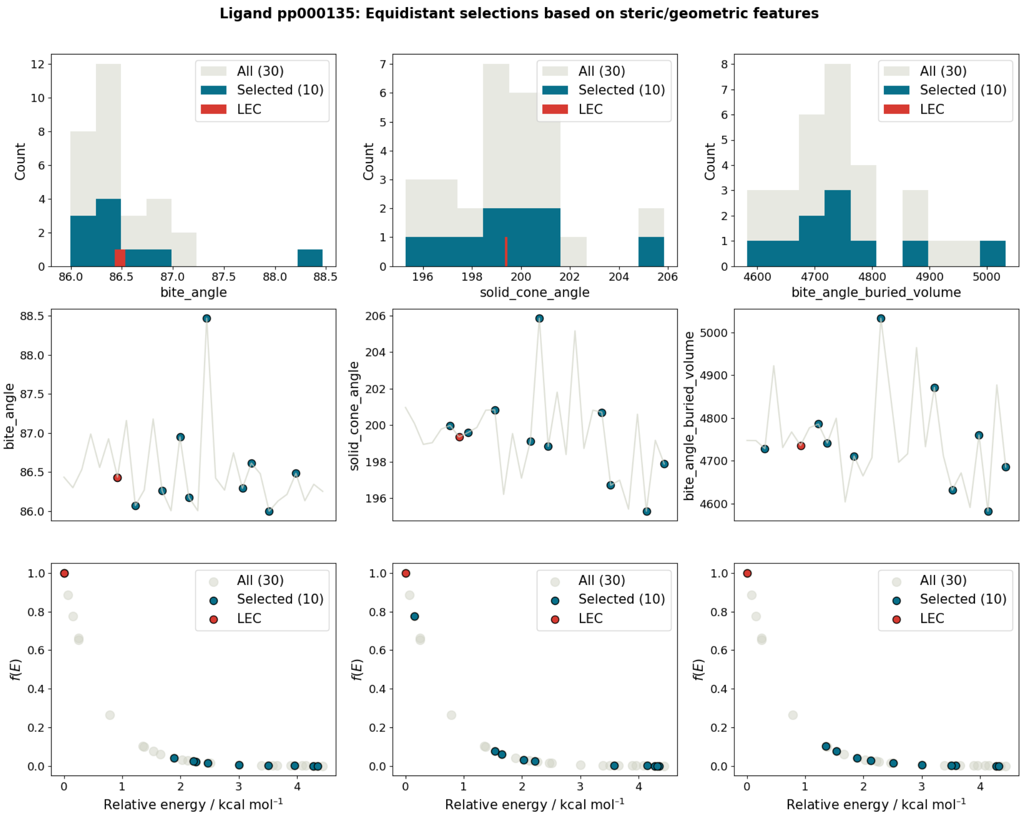

Figure S52: Equidistant selection of conformers (10 total) for complex **pp000135** based on steric and/or geometric features.

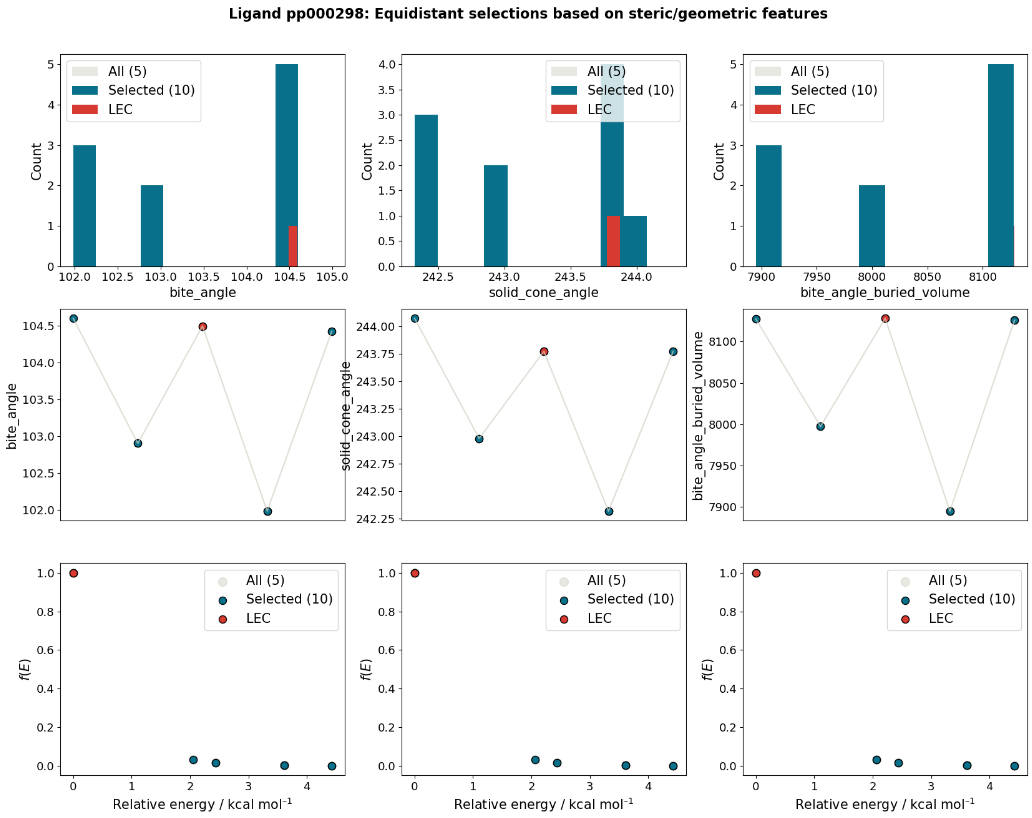

Figure S53: Equidistant selection of conformers (10 total) for complex **pp000298** based on steric and/or geometric features.

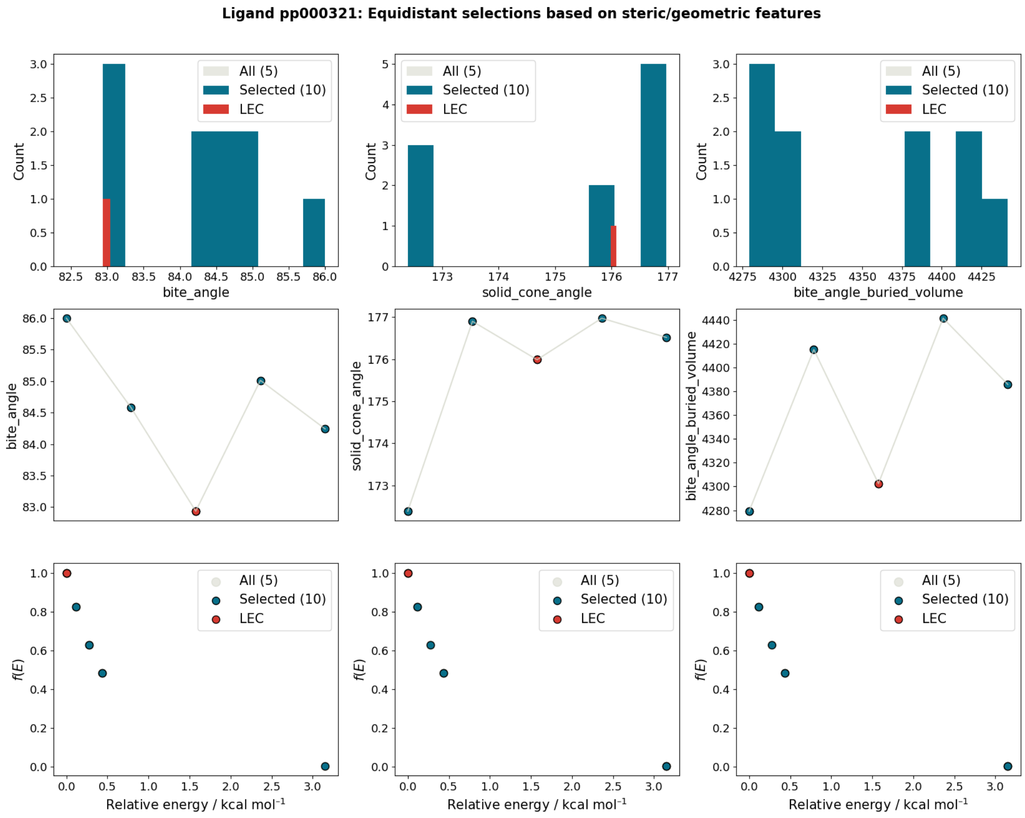

Figure S54: Equidistant selection of conformers (10 total) for complex **pp000321** based on steric and/or geometric features.

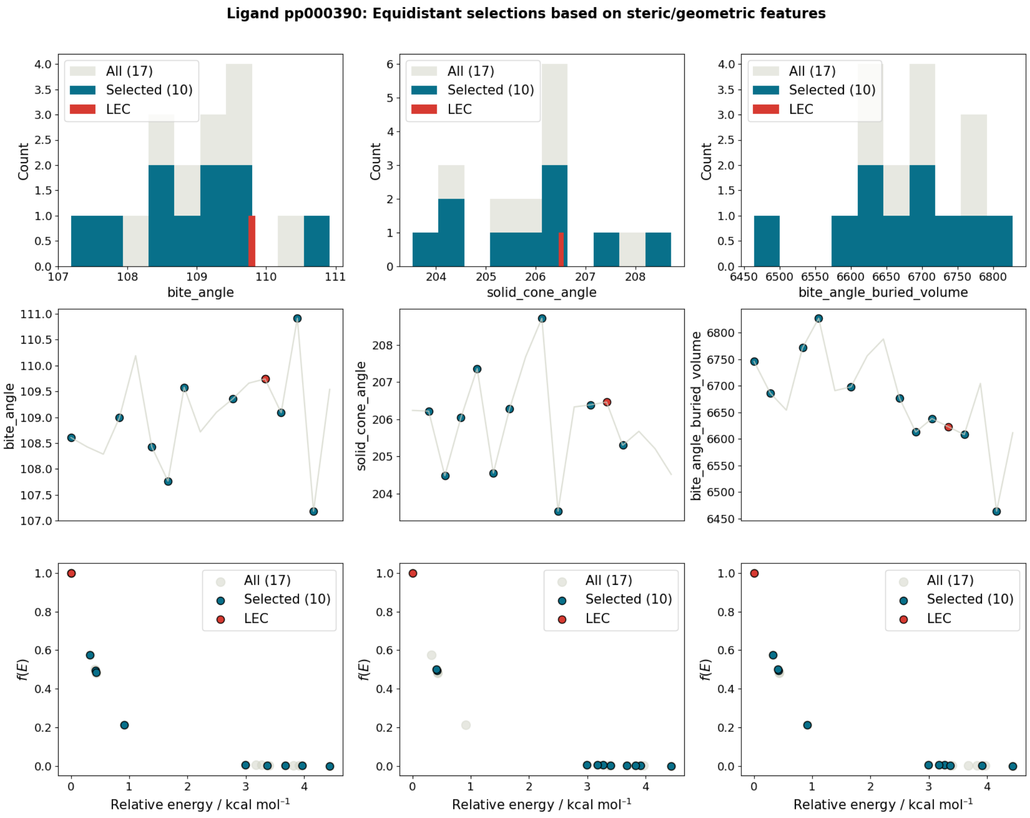

Figure S55: Equidistant selection of conformers (10 total) for complex **pp000390** based on steric and/or geometric features.

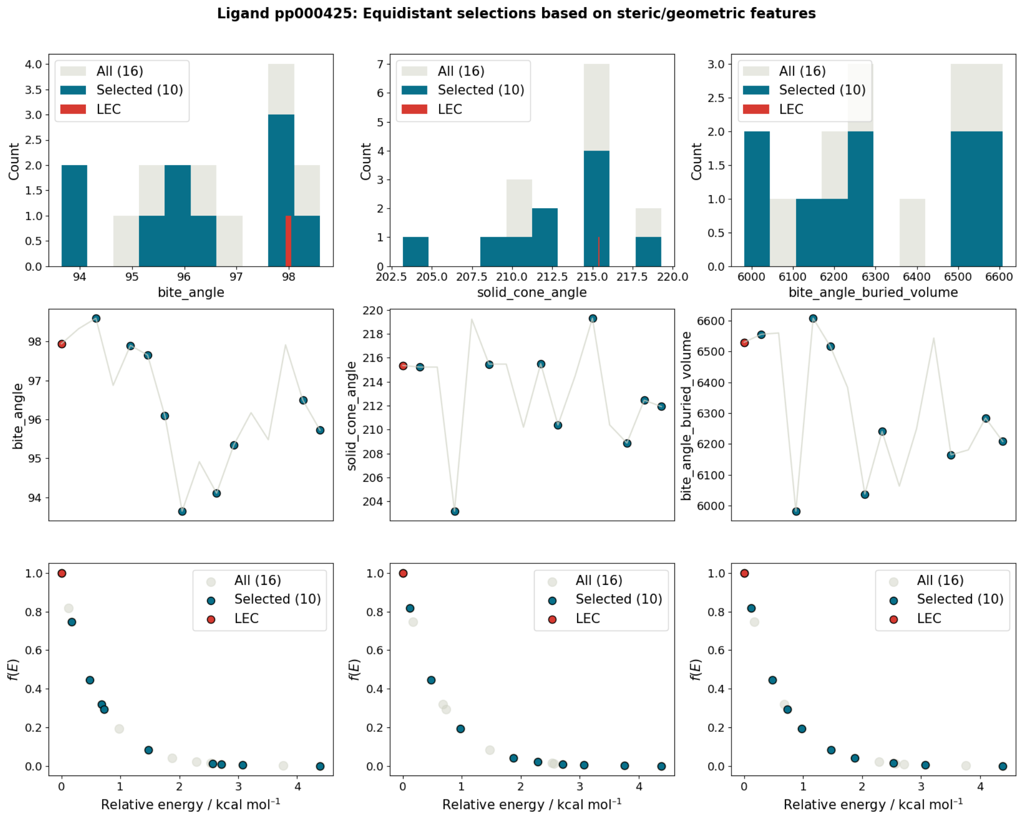

Figure S56: Equidistant selection of conformers (10 total) for complex **pp000425** based on steric and/or geometric features.

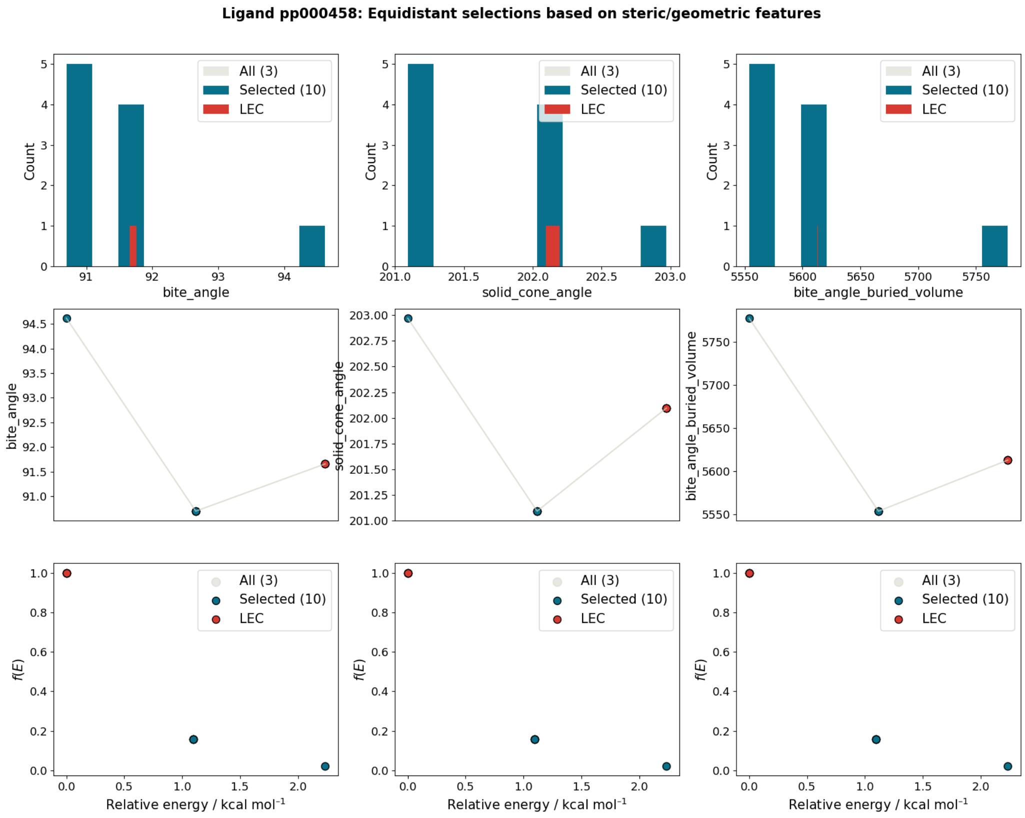

Figure S57: Equidistant selection of conformers (10 total) for complex **pp000458** based on steric and/or geometric features.

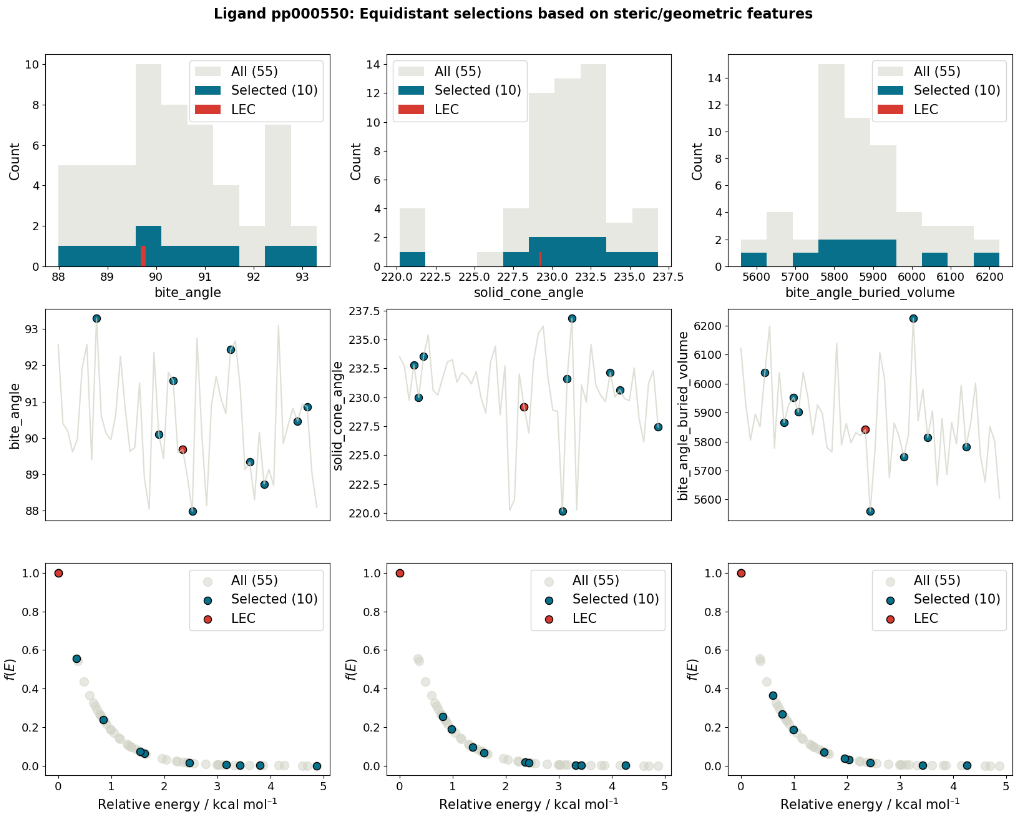

Figure S58: Equidistant selection of conformers (10 total) for complex **pp000550** based on steric and/or geometric features.

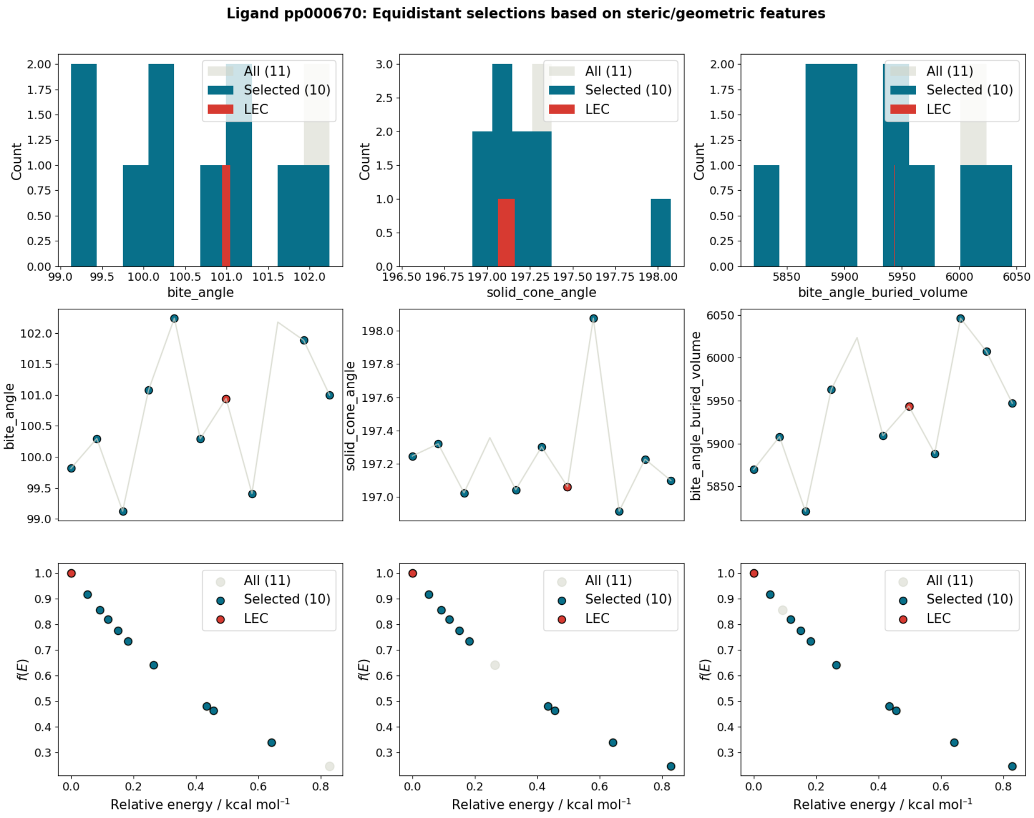

Figure S59: Equidistant selection of conformers (10 total) for complex **pp000670** based on steric and/or geometric features.

5.3.2 Effect of selection based on GFN2-xTB energy on the DFT ensemble conformers

In the following series of plots (Figures S84 - S95, one for each complex used in this study), the clustered ensembles calculated at the DFT-level for a series of features are given. The full ensemble was calculated to simulate the results of the conformer selection. The red dots on each of the plots indicated which conformers are picked based on their GFN2-xTB energies.

5.3.3 Effect of selection based on bite angle on the DFT ensemble conformers

In the following series of plots (Figures S72 - S83, one for each complex used in this study), the clustered ensembles calculated at the DFT-level for a series of features are given. The full ensemble was calculated to simulate the results of the conformer selection. The red dots on each of the plots indicated which conformers are picked based on bite angle values.

5.3.4 Effect of selection based on percent buried volume on the DFT ensemble conformers

In the following series of plots (Figures S84 - S95, one for each complex used in this study), the clustered ensembles calculated at the DFT-level for a series of features are given. The full ensemble was calculated to simulate the results of the conformer selection. The red dots on each of the plots indicated which conformers are picked based on percent buried volume ($r = 3.5\mathring{A}$) values.

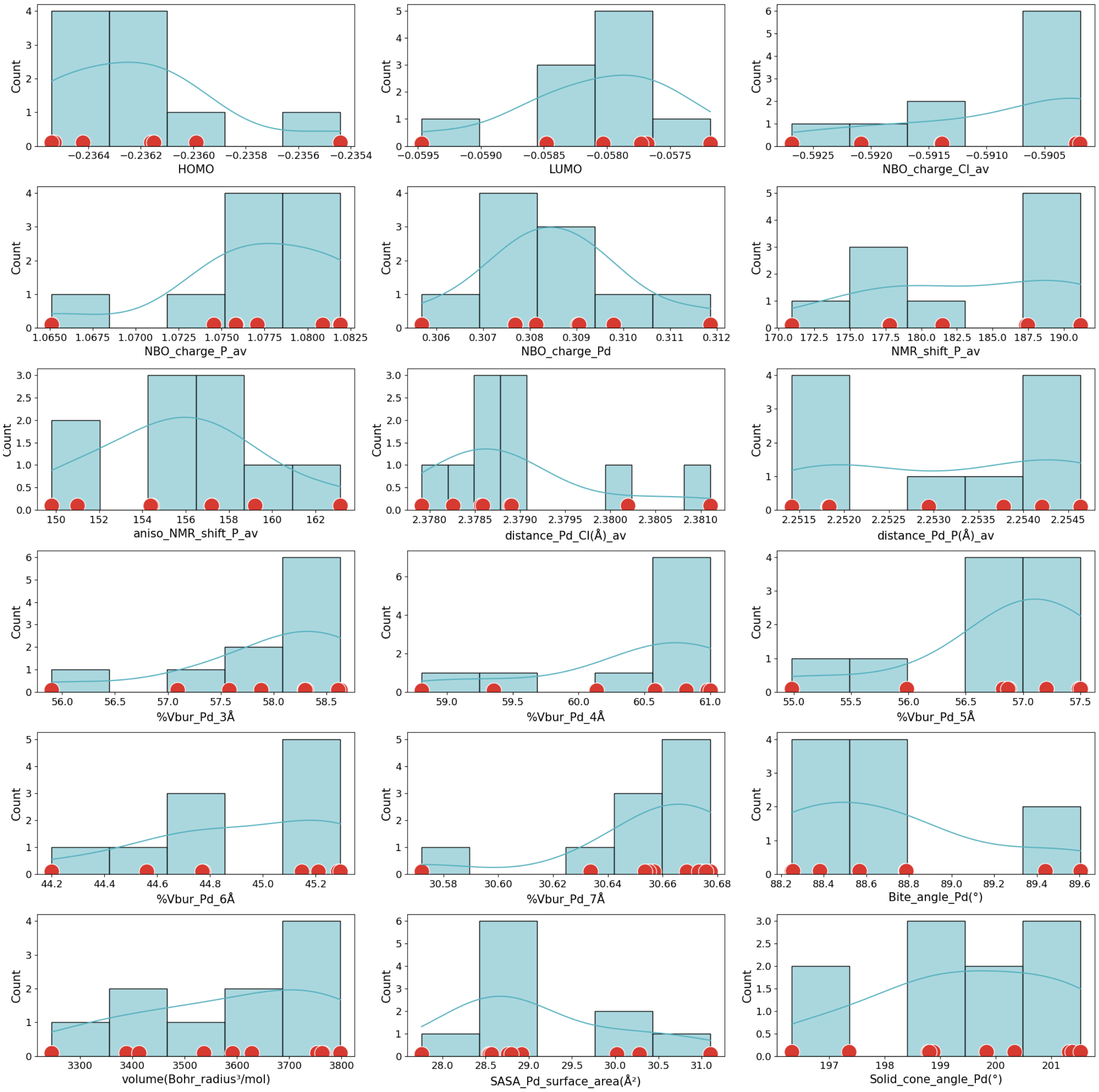

Figure S60: DFT-level feature distributions for ligand **pp000067**. Selected conformers by GFN2-xTB energy are indicted with red dots.

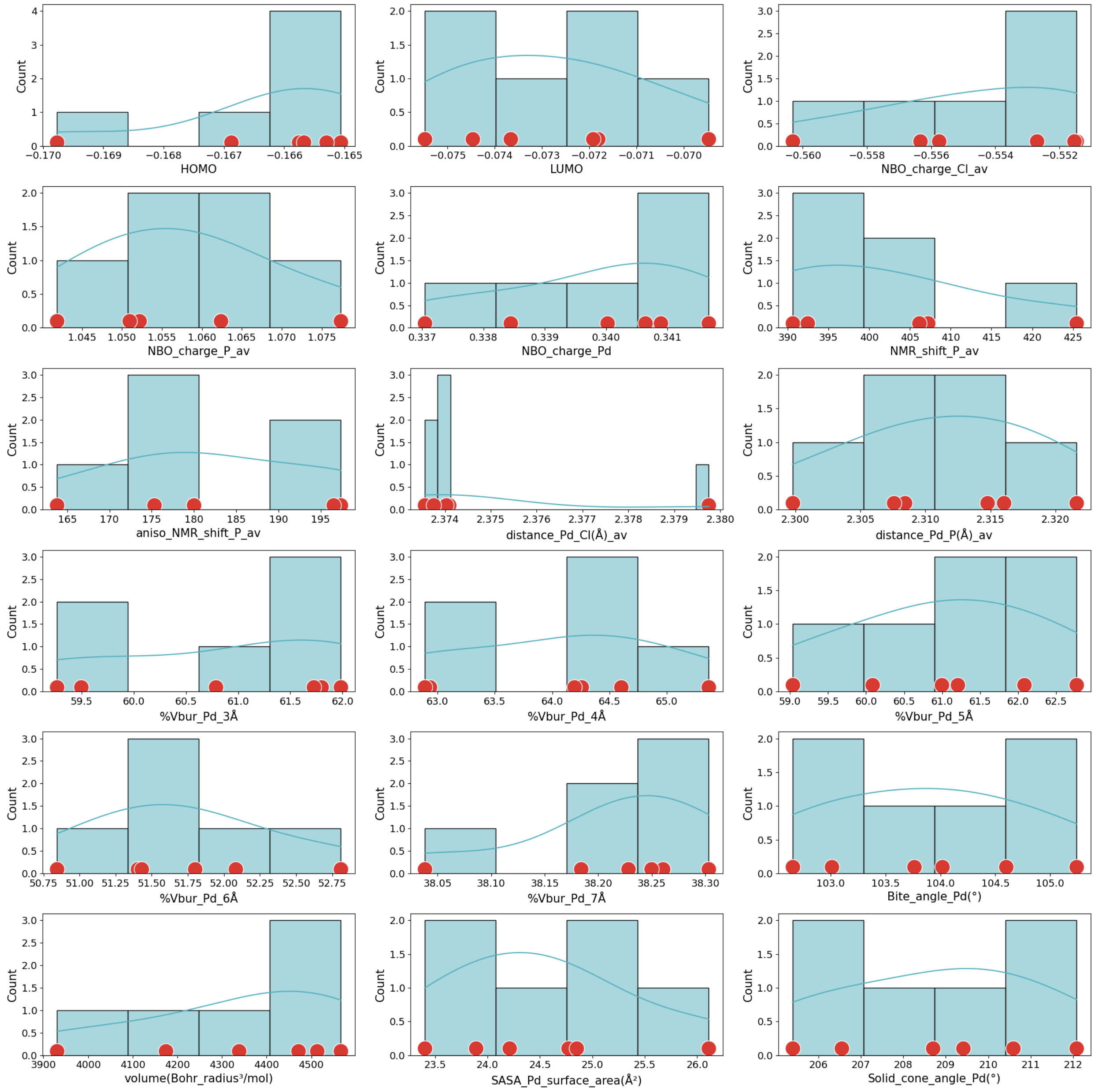

Figure S61: DFT-level feature distributions for ligand **pp000085**. Selected conformers by GFN2-xTB energy are indicted with red dots.

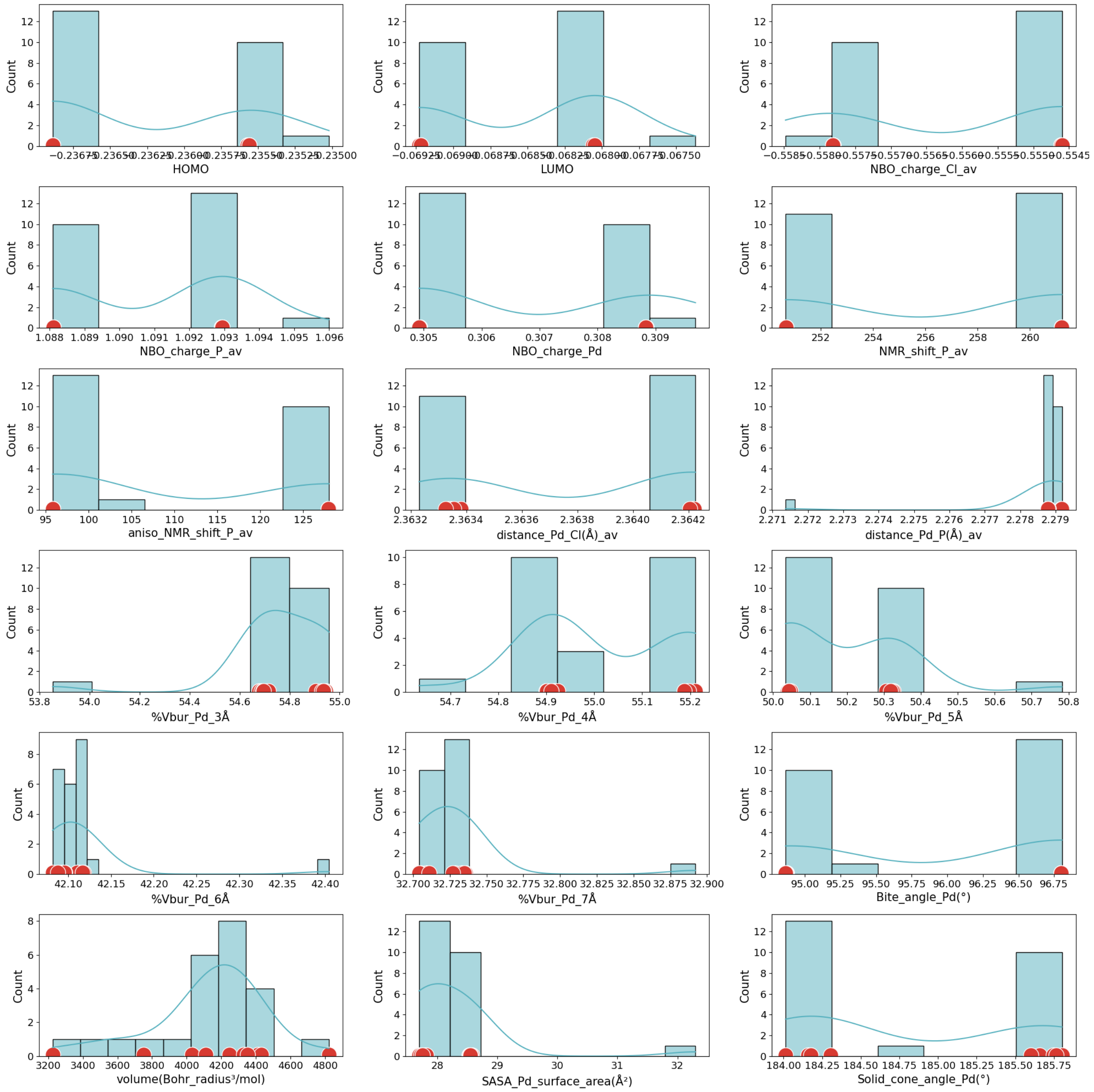

Figure S62: DFT-level feature distributions for ligand **pp000091**. Selected conformers by GFN2-xTB energy are indicted with red dots.

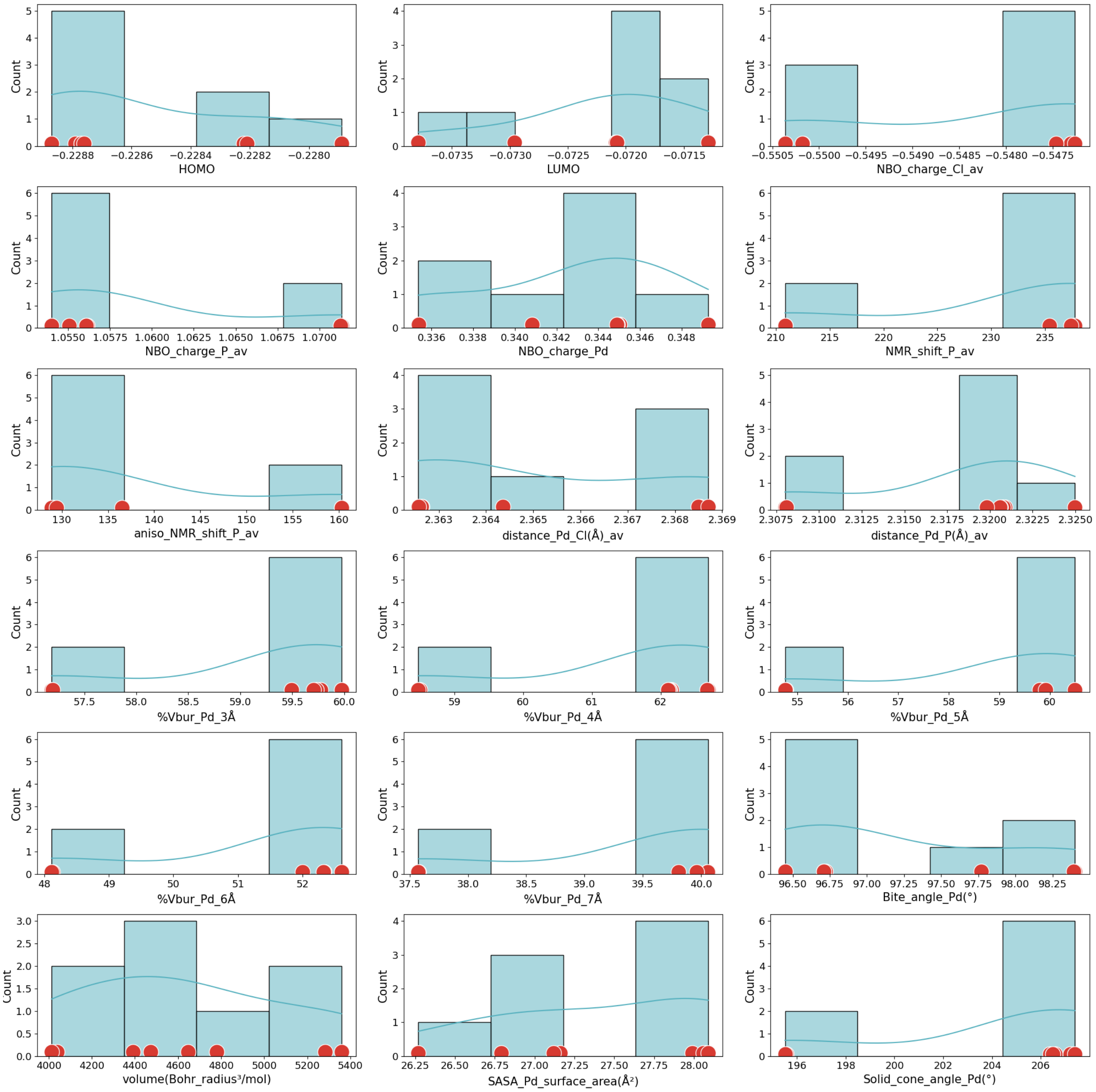

Figure S63: DFT-level feature distributions for ligand **pp000110**. Selected conformers by GFN2-xTB energy are indicted with red dots.

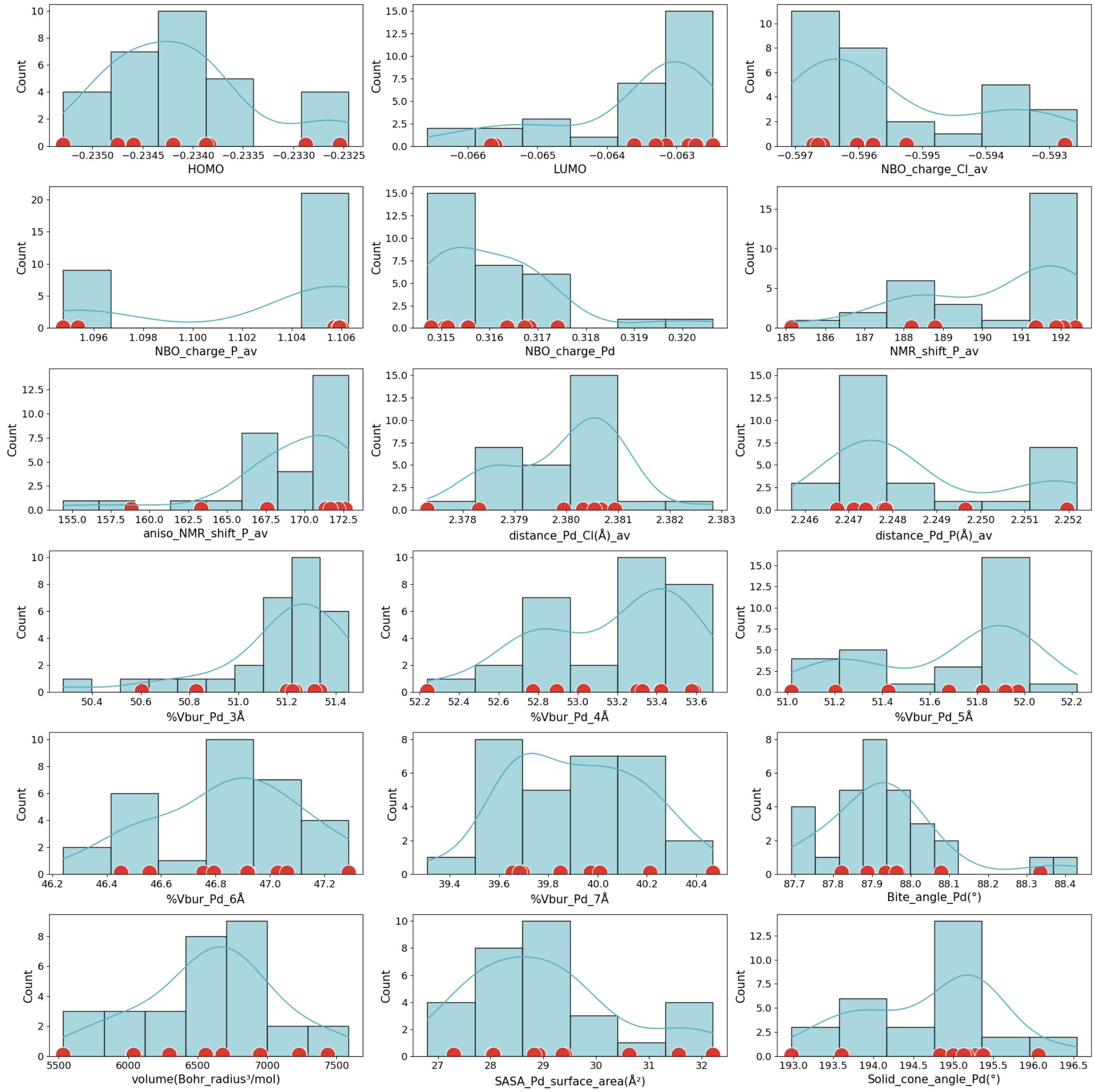

Figure S64: DFT-level feature distributions for ligand **pp000135**. Selected conformers by GFN2-xTB energy are indicted with red dots.

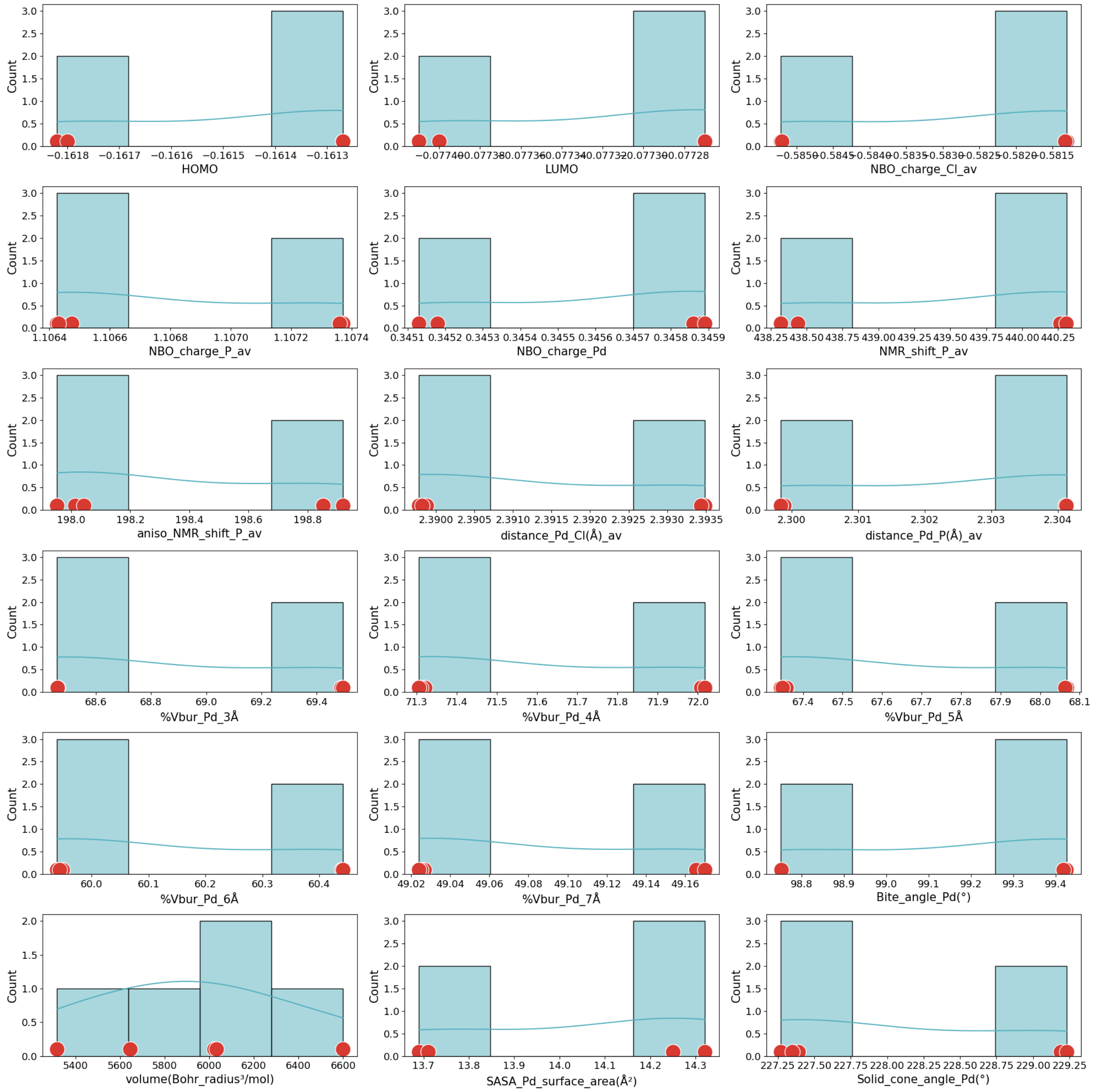

Figure S65: DFT-level feature distributions for ligand **pp000298**. Selected conformers by GFN2-xTB energy are indicted with red dots.

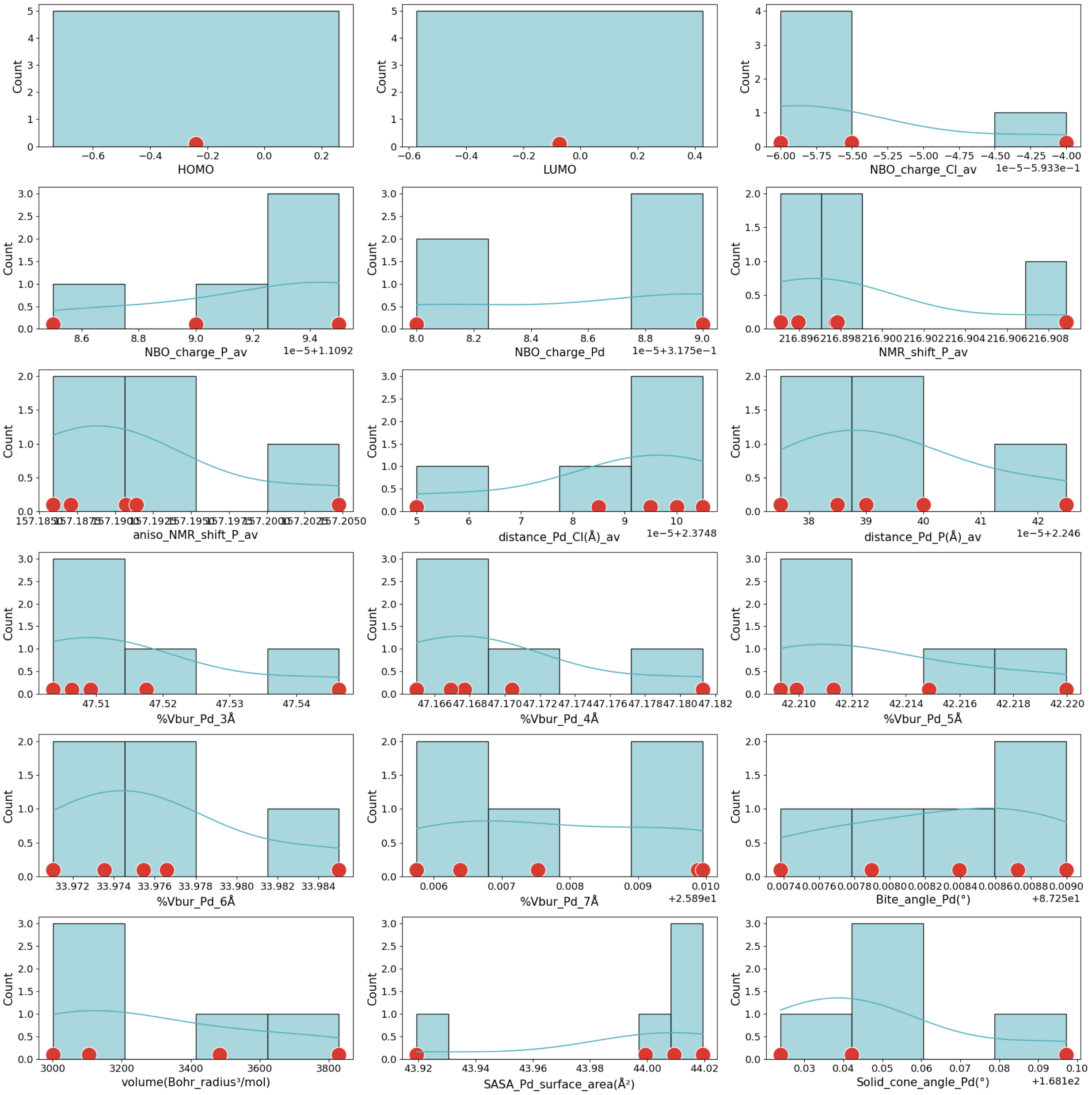

Figure S66: DFT-level feature distributions for ligand **pp000321**. Selected conformers by GFN2-xTB energy are indicted with red dots.

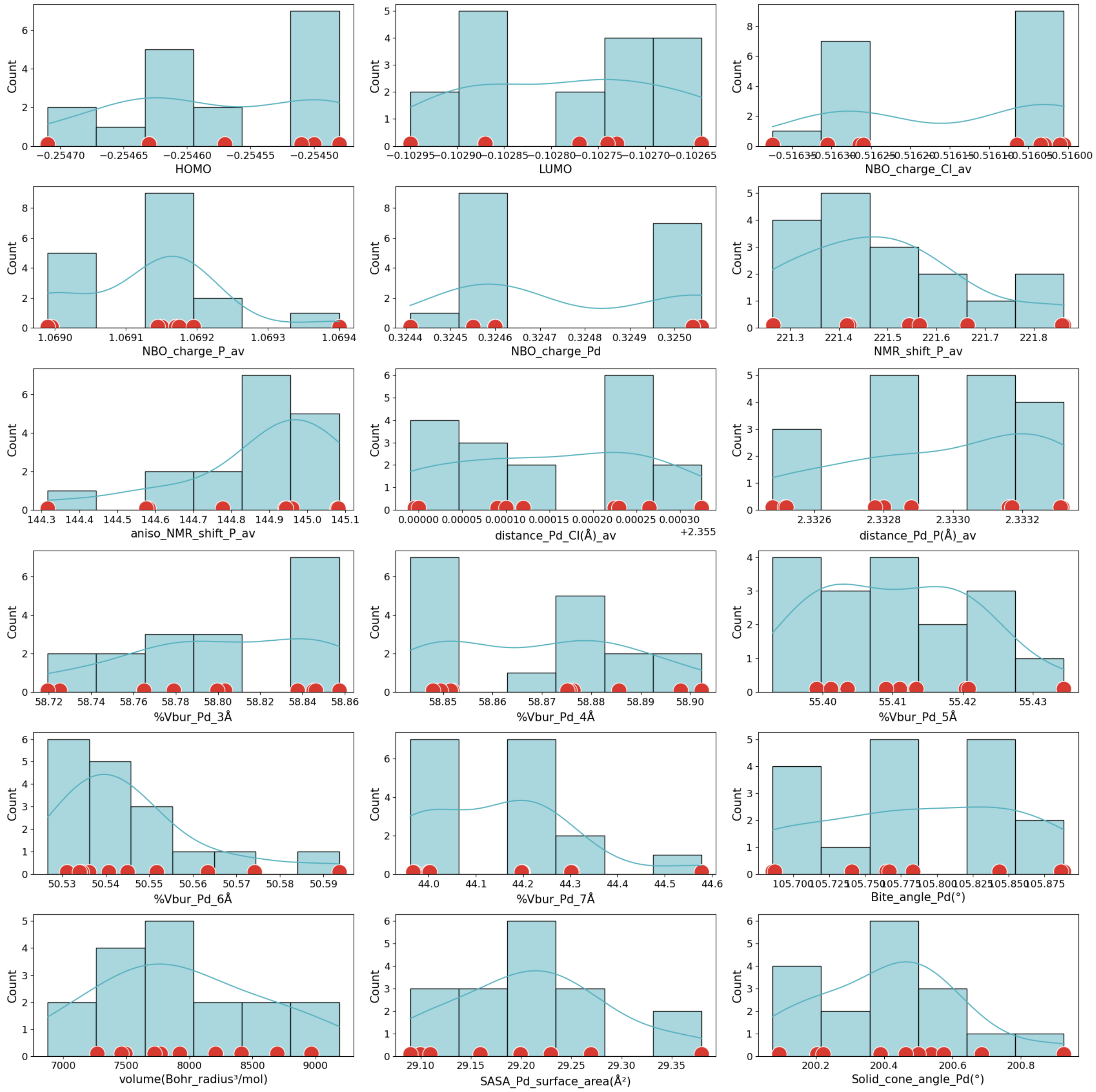

Figure S67: DFT-level feature distributions for ligand **pp000390**. Selected conformers by GFN2-xTB energy are indicted with red dots.

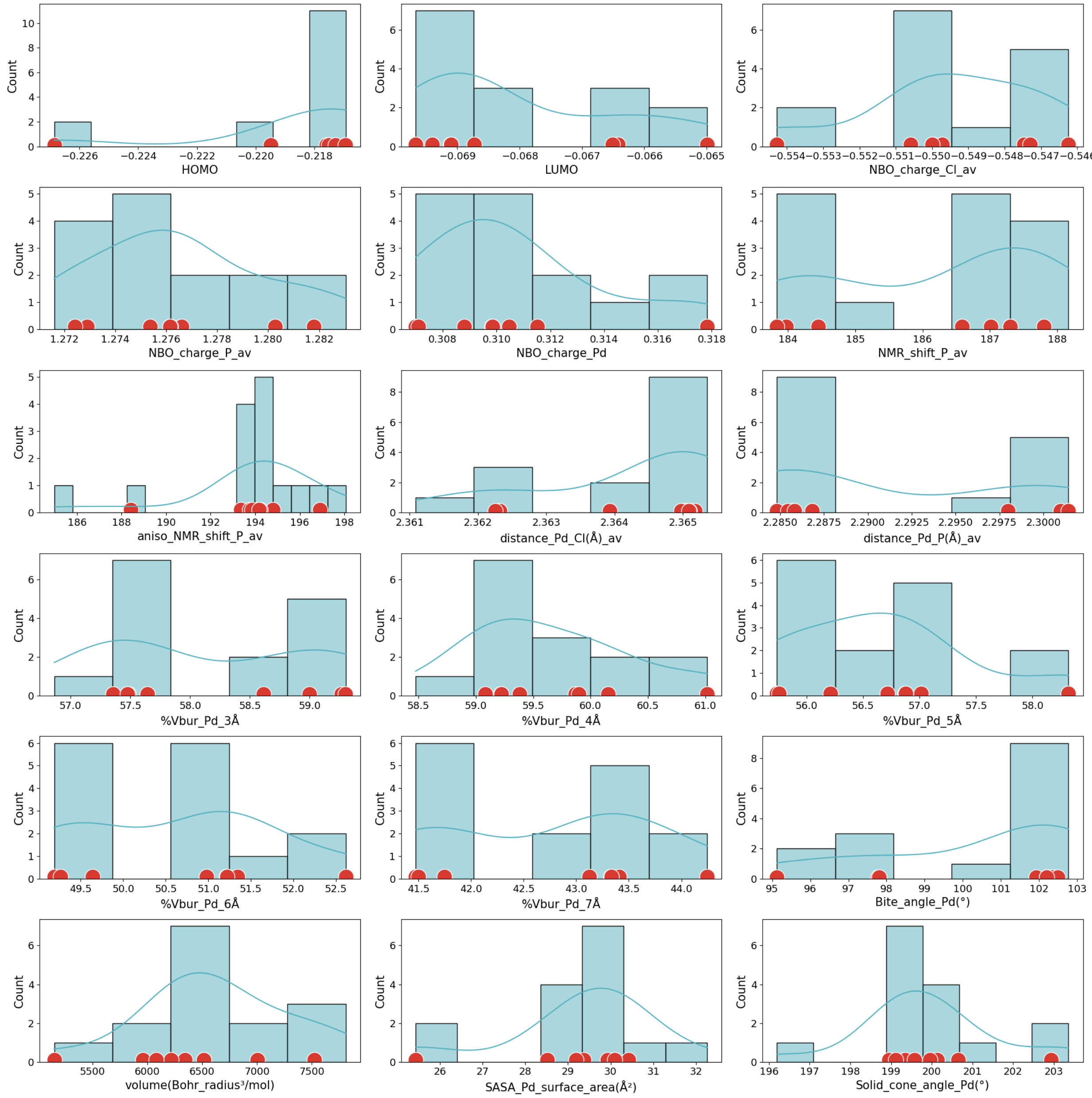

Figure S68: DFT-level feature distributions for ligand **pp000425**. Selected conformers by GFN2-xTB energy are indicted with red dots.

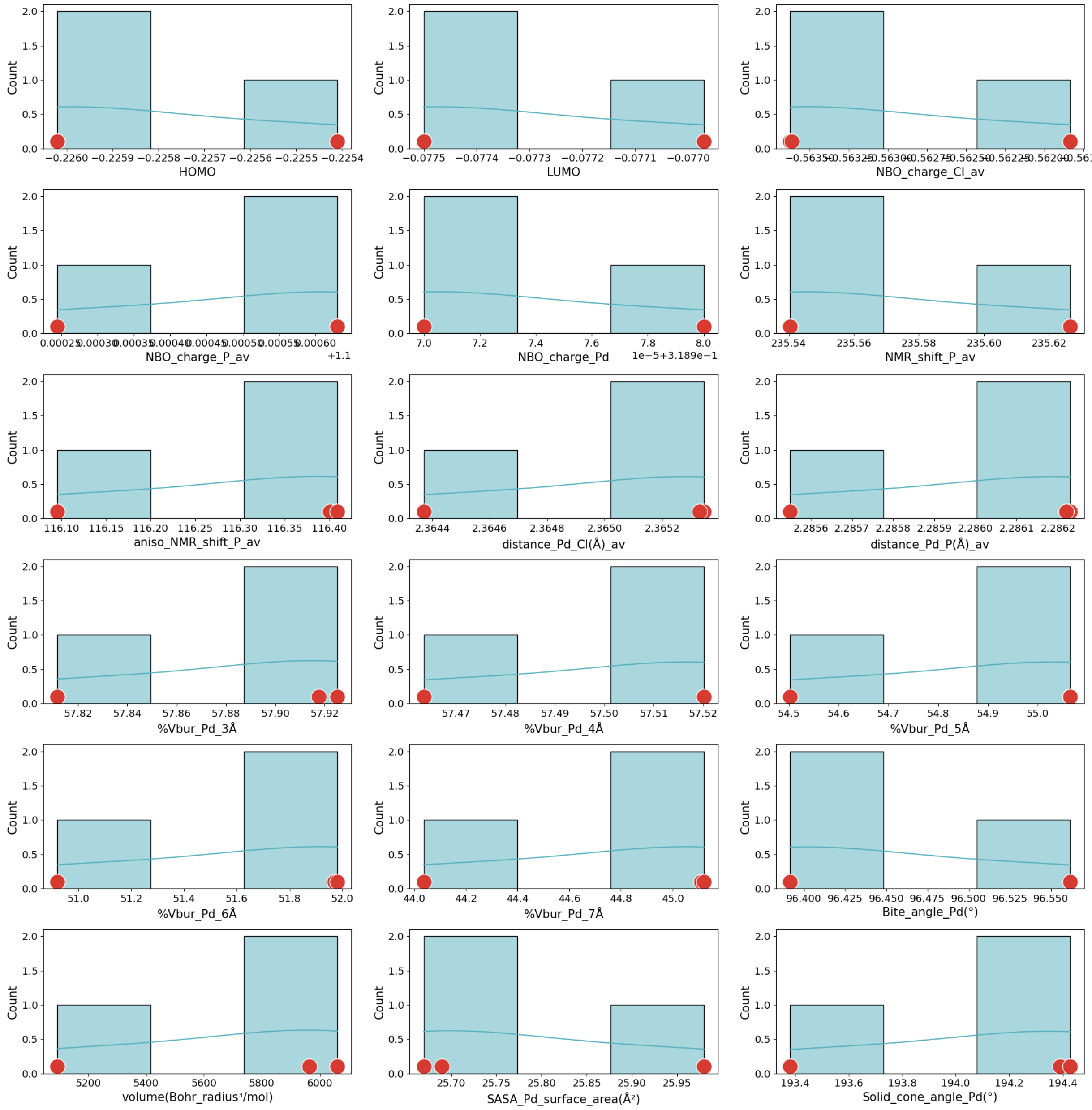

Figure S69: DFT-level feature distributions for ligand **pp000458**. Selected conformers by GFN2-xTB energy are indicted with red dots.

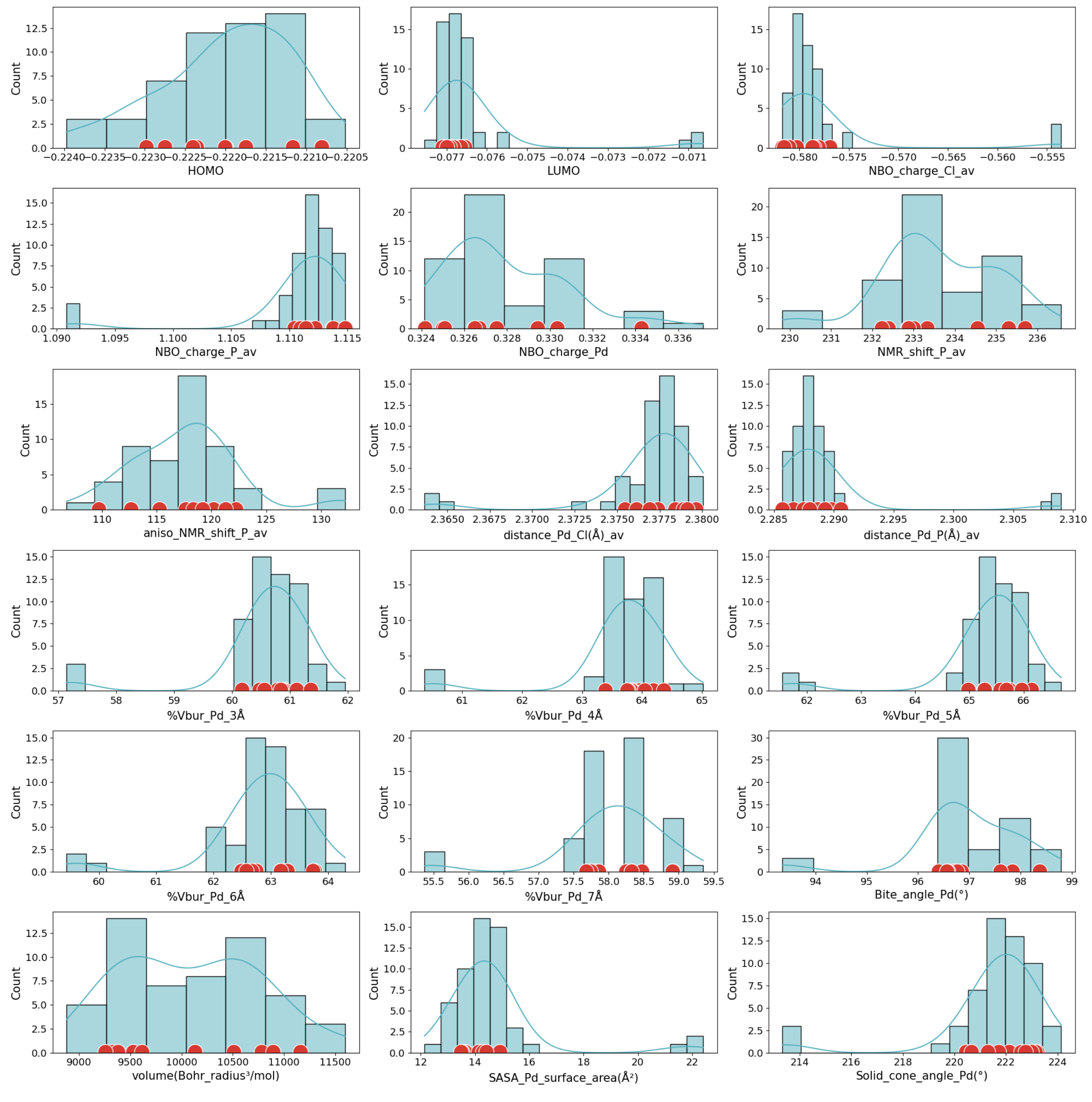

Figure S70: DFT-level feature distributions for ligand **pp000550**. Selected conformers by GFN2-xTB energy are indicted with red dots.

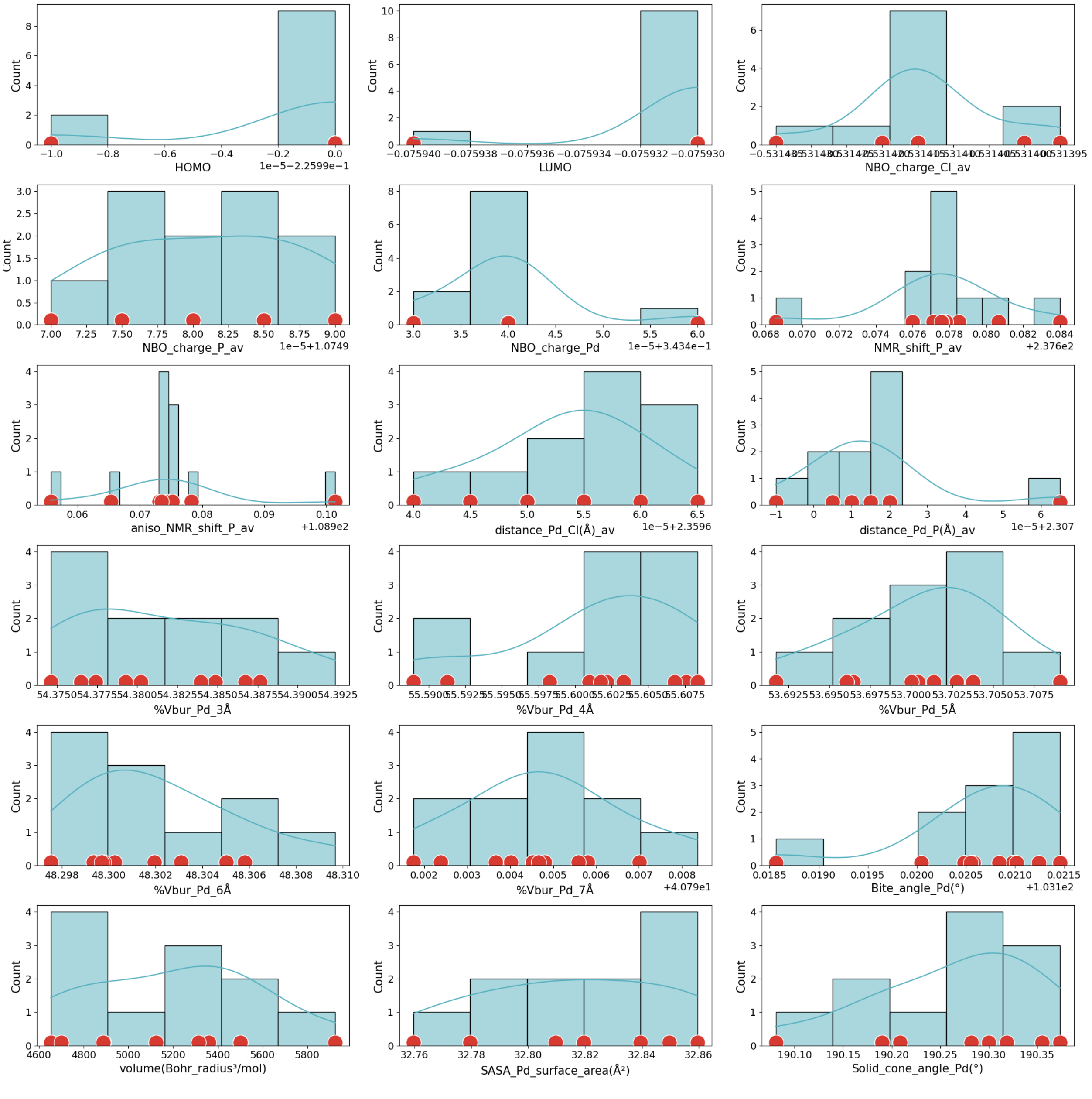

Figure S71: DFT-level feature distributions for ligand **pp000670**. Selected conformers by GFN2-xTB energy are indicted with red dots.

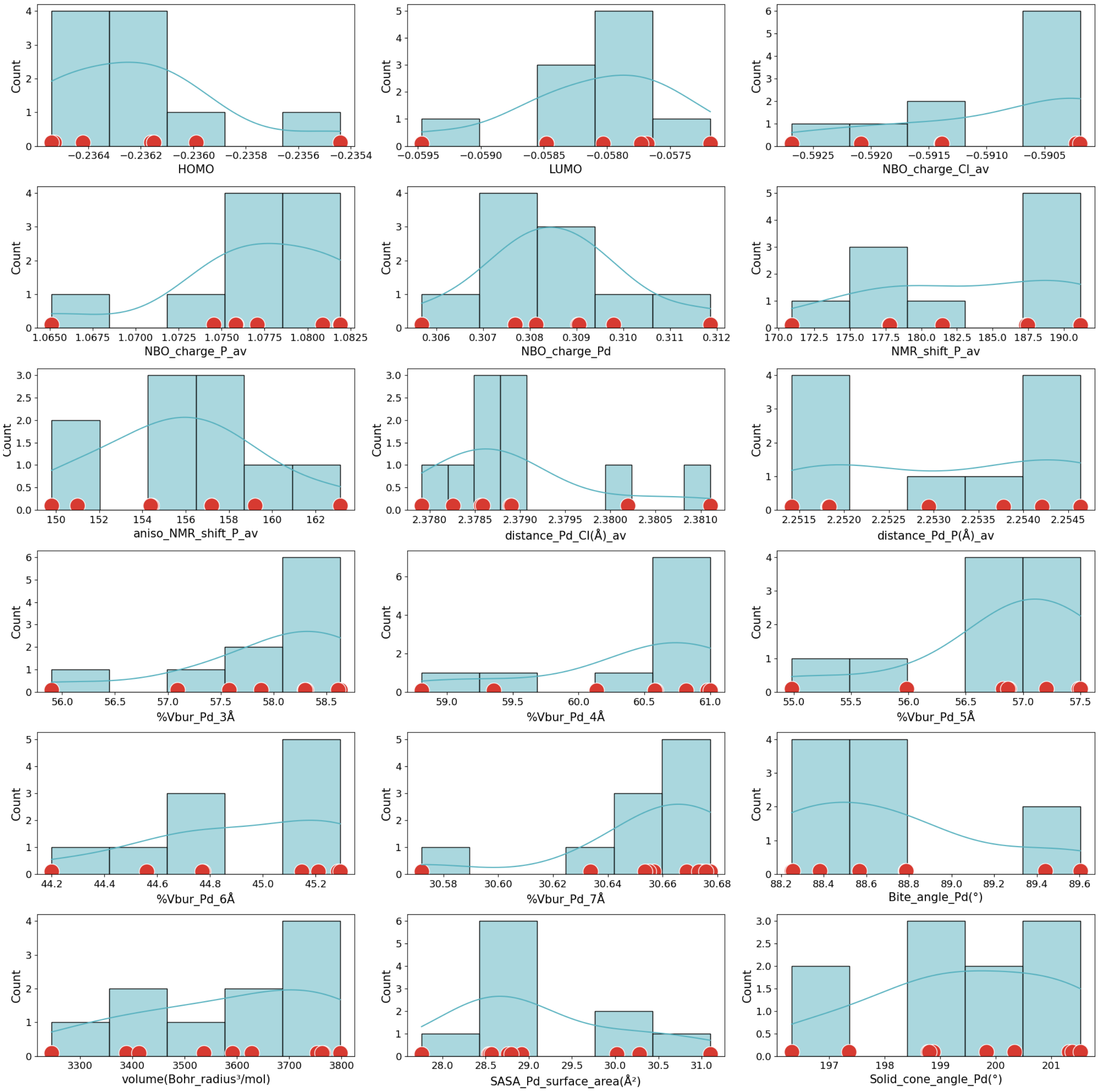

Figure S72: DFT-level feature distributions for ligand **pp000067**. Selected conformers by bite angle are indicted with red dots.

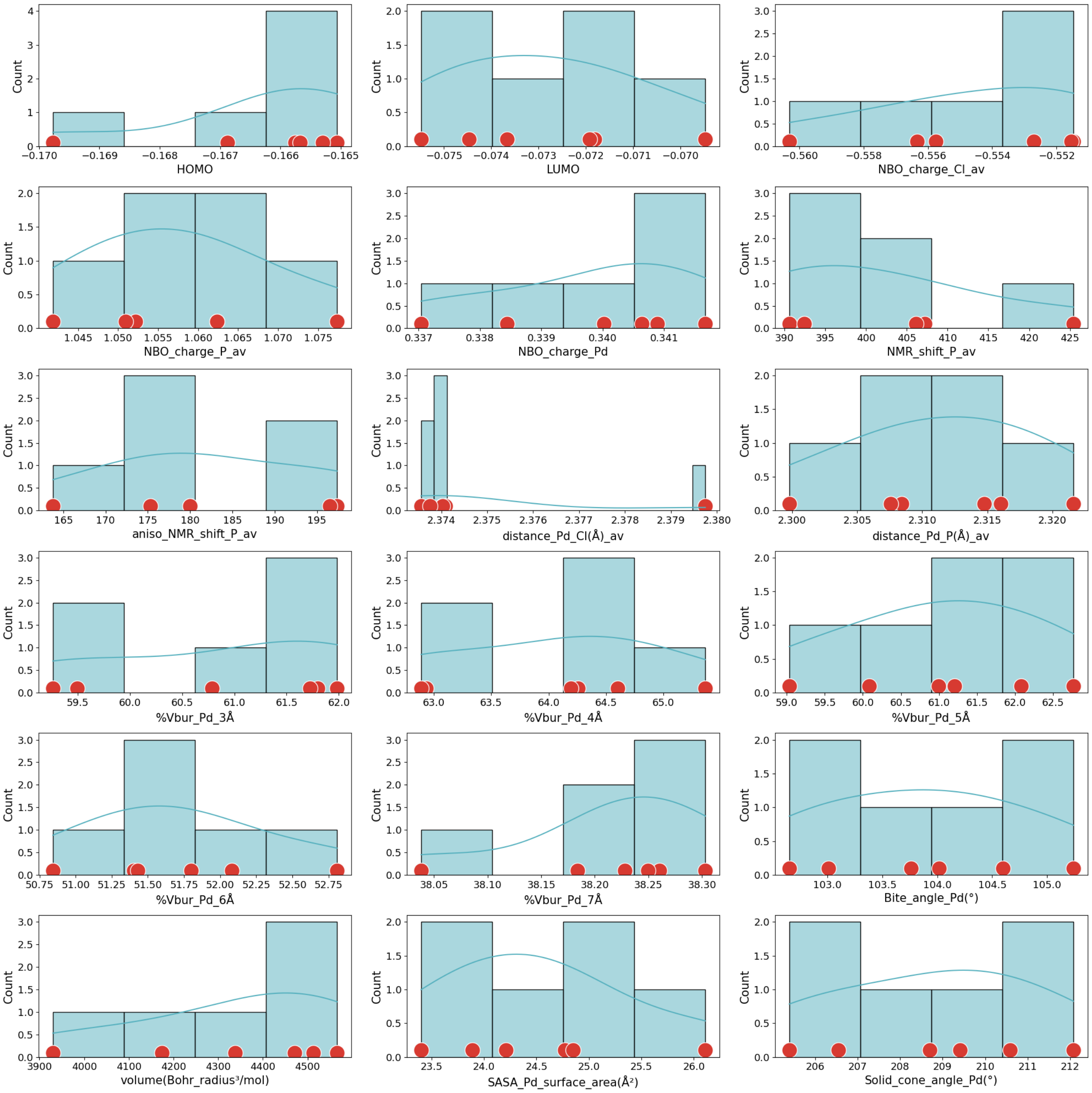

Figure S73: DFT-level feature distributions for ligand **pp000085**. Selected conformers by bite angle are indicted with red dots.

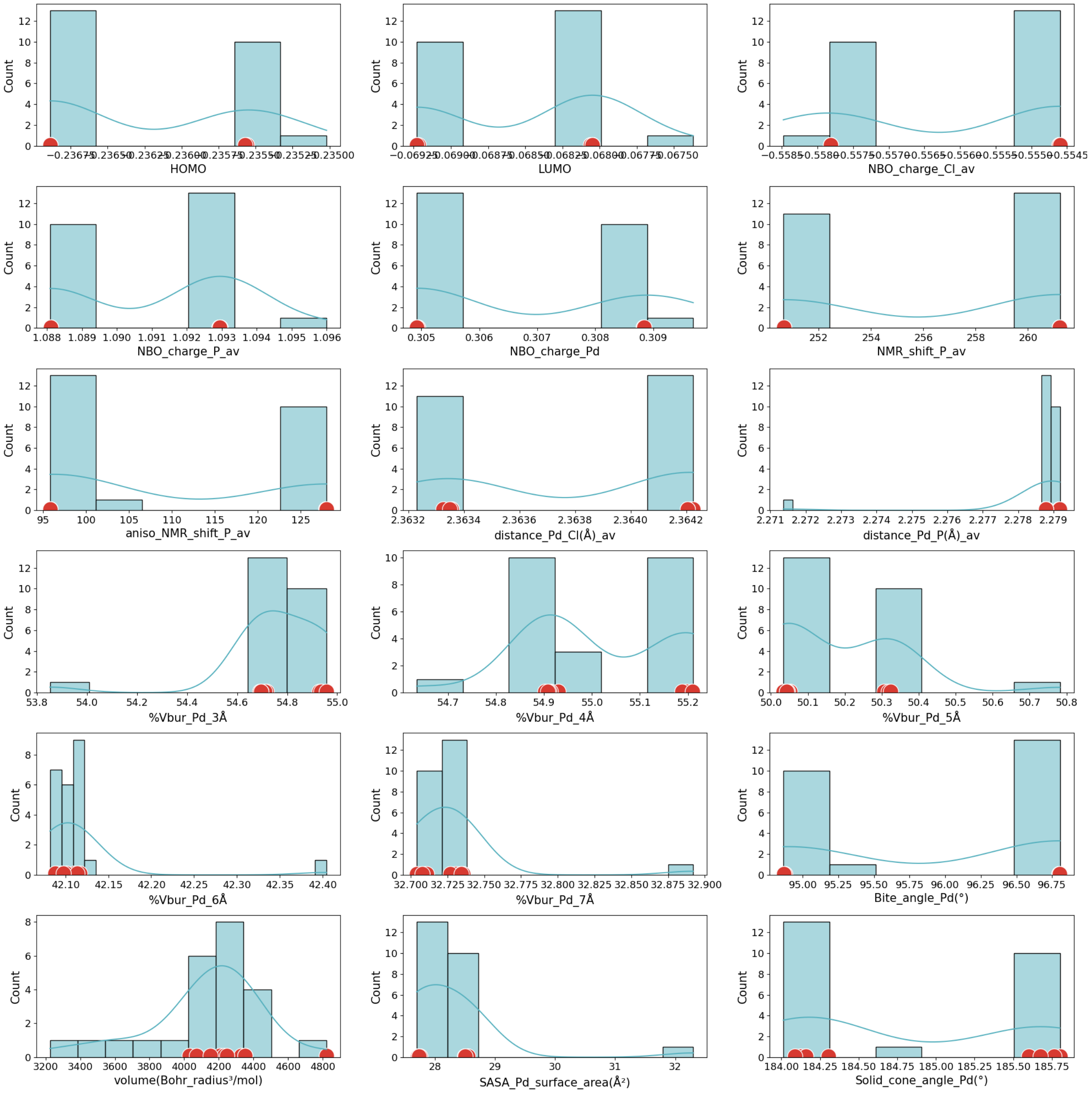

Figure S74: DFT-level feature distributions for ligand **pp000091**. Selected conformers by bite angle are indicted with red dots.

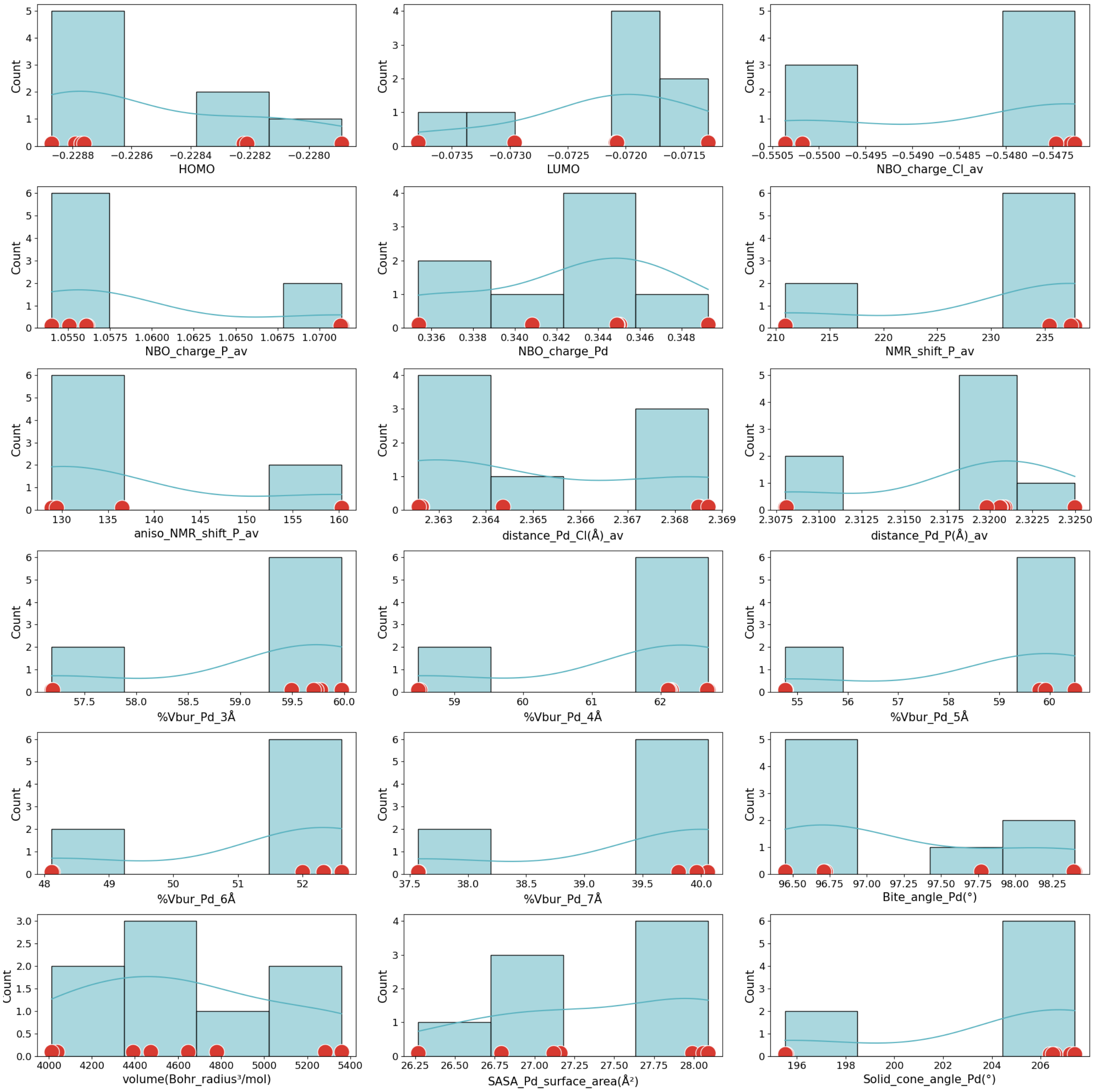

Figure S75: DFT-level feature distributions for ligand **pp000110**. Selected conformers by bite angle are indicted with red dots.

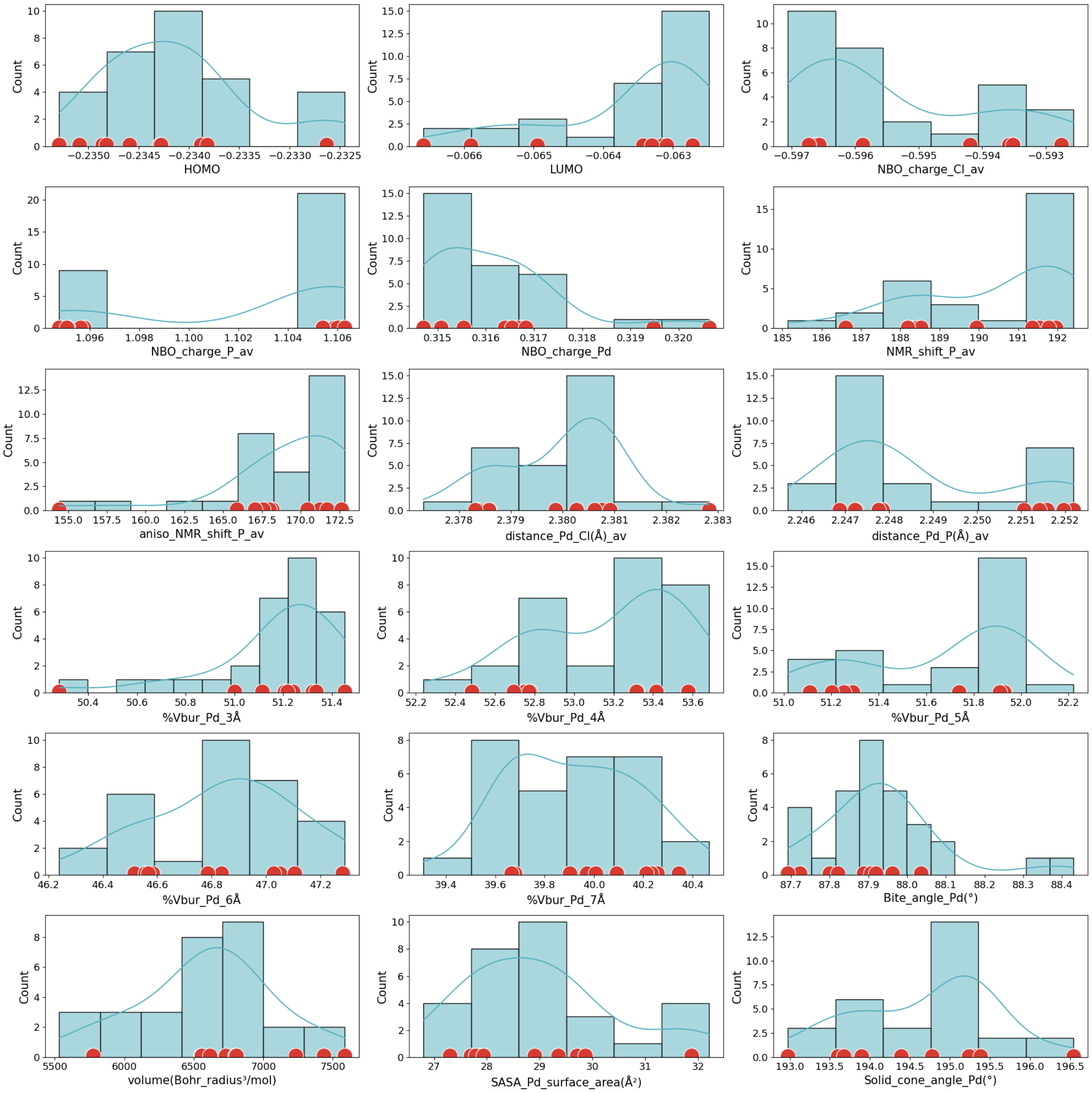

Figure S76: DFT-level feature distributions for ligand **pp000135**. Selected conformers by bite angle are indicted with red dots.

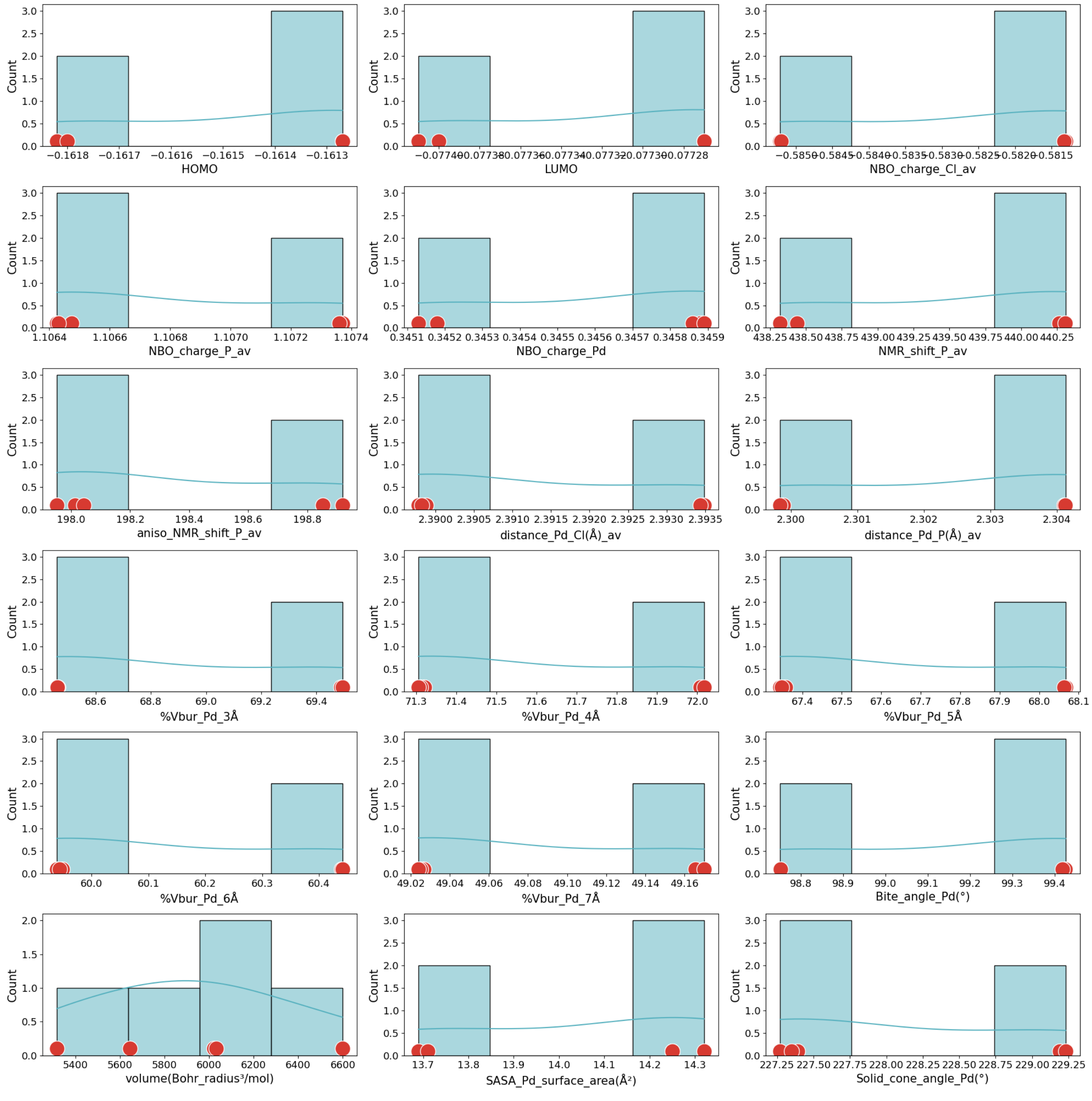

Figure S77: DFT-level feature distributions for ligand **pp000298**. Selected conformers by bite angle are indicted with red dots.

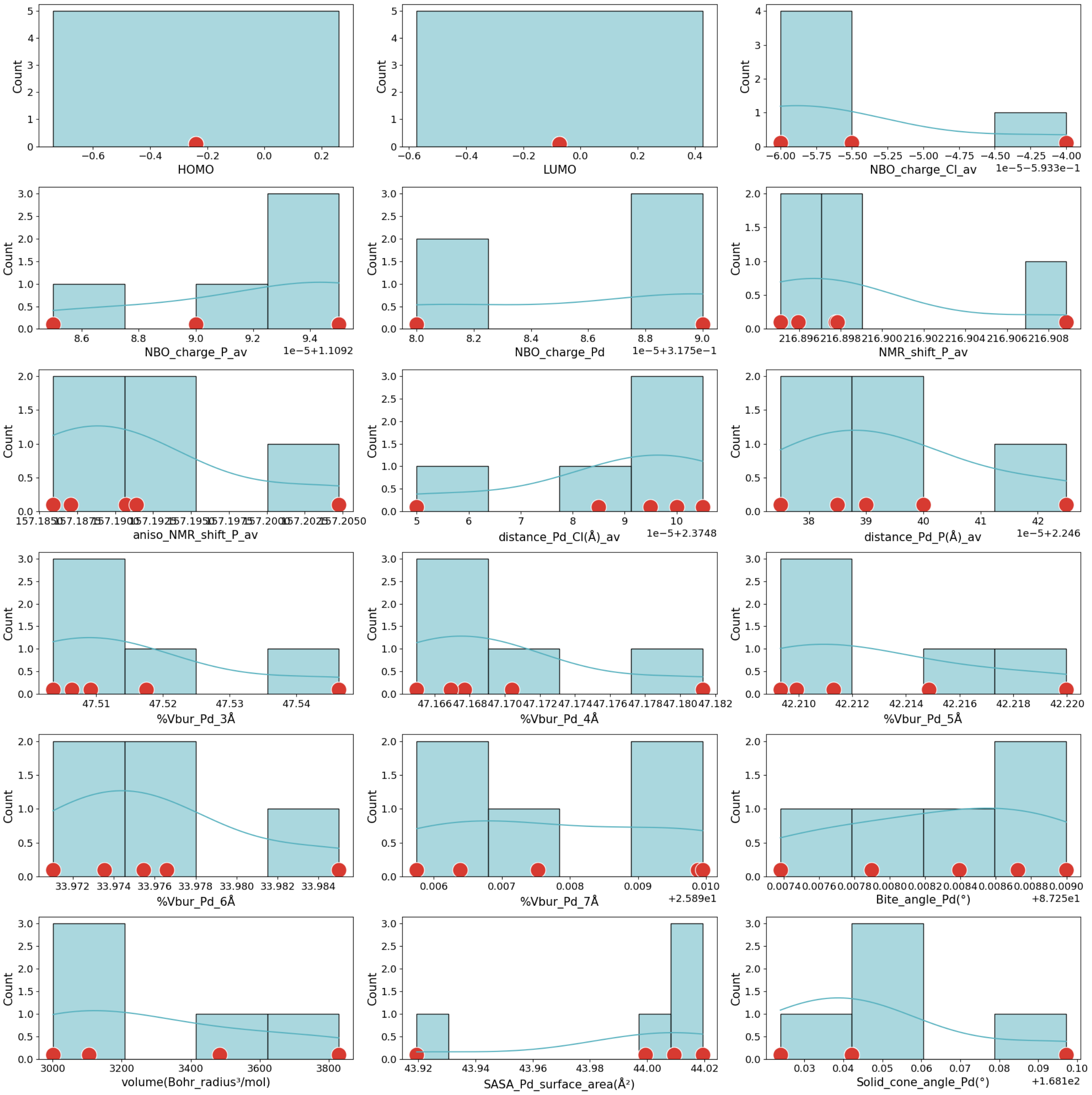

Figure S78: DFT-level feature distributions for ligand **pp000321**. Selected conformers by bite angle are indicted with red dots.

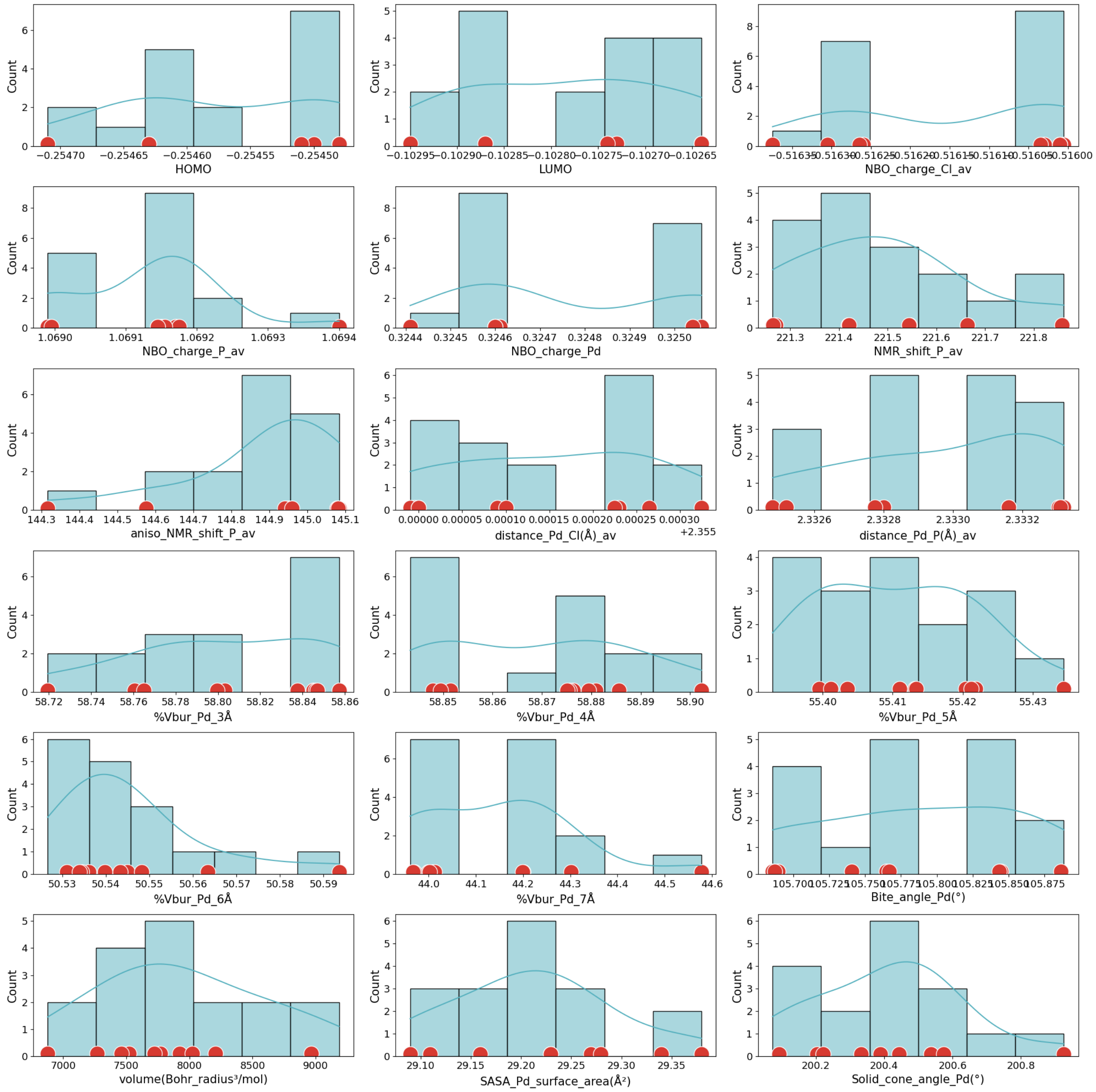

Figure S79: DFT-level feature distributions for ligand **pp000390**. Selected conformers by bite angle are indicted with red dots.

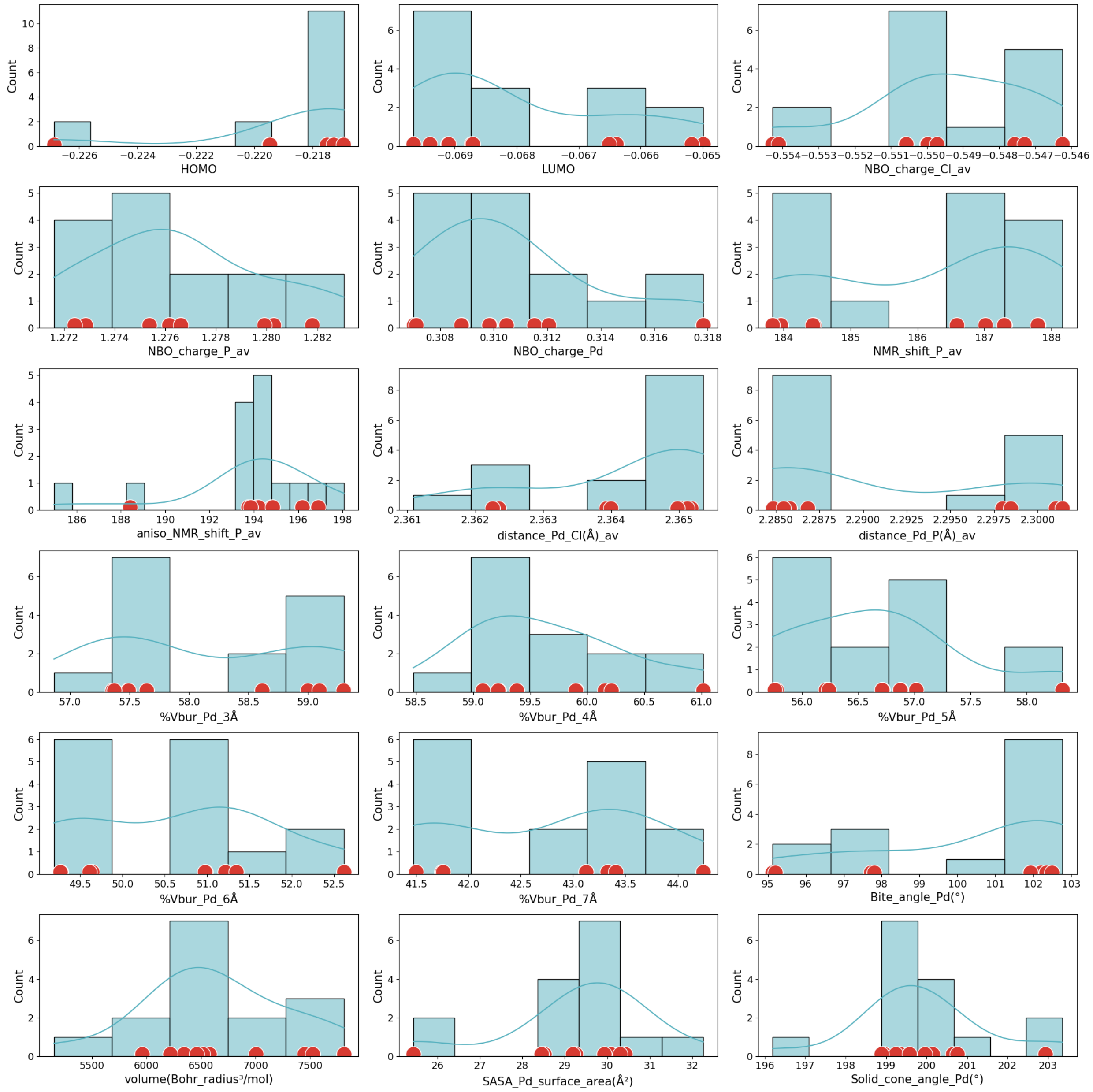

Figure S80: DFT-level feature distributions for ligand **pp000425**. Selected conformers by bite angle are indicted with red dots.

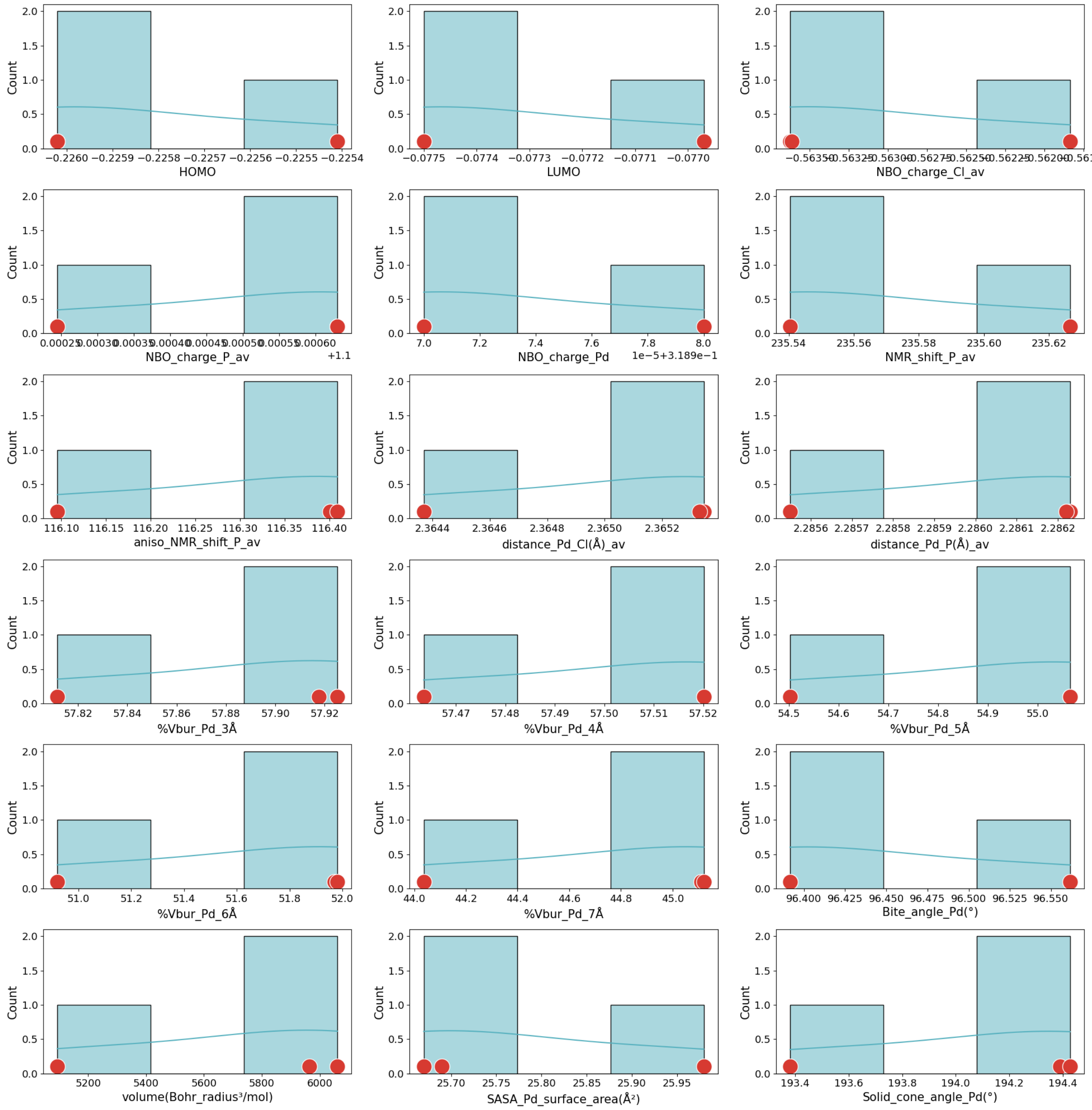

Figure S81: DFT-level feature distributions for ligand **pp000458**. Selected conformers by bite angle indicted with red dots.

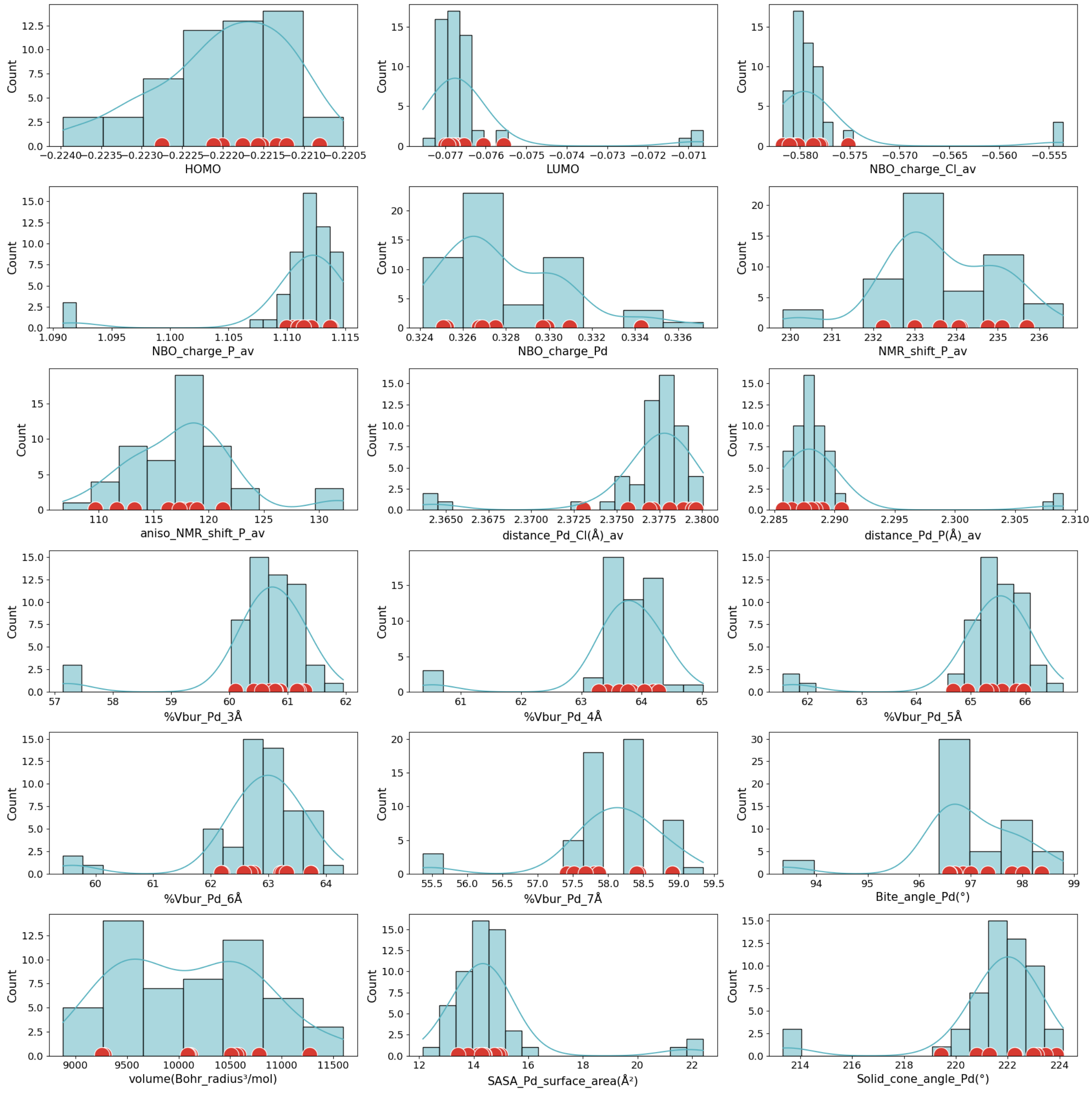

Figure S82: DFT-level feature distributions for ligand **pp000550**. Selected conformers by bite angle indicted with red dots.

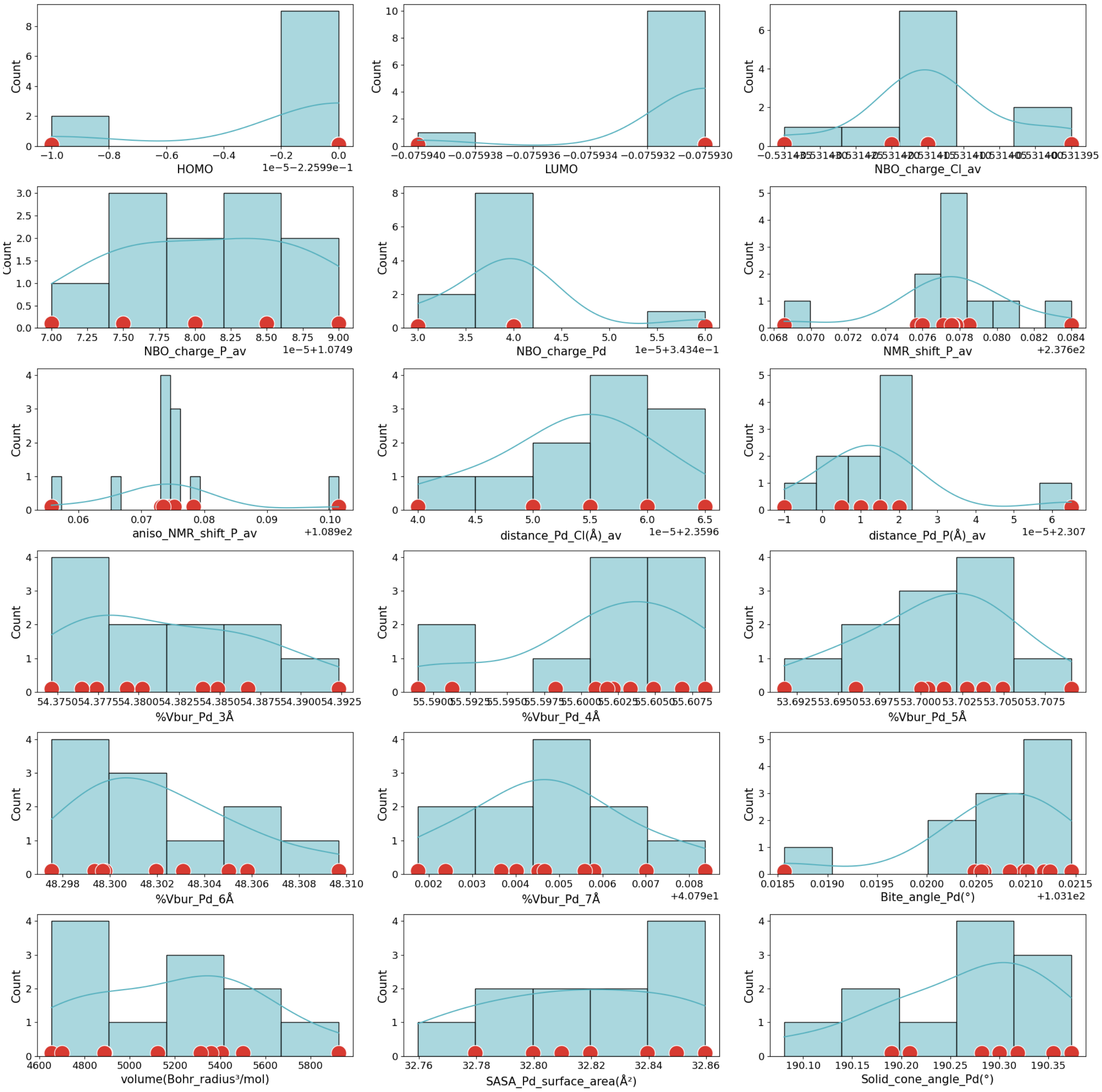

Figure S83: DFT-level feature distributions for ligand **pp000670**. Selected conformers by bite angle are indicted with red dots.

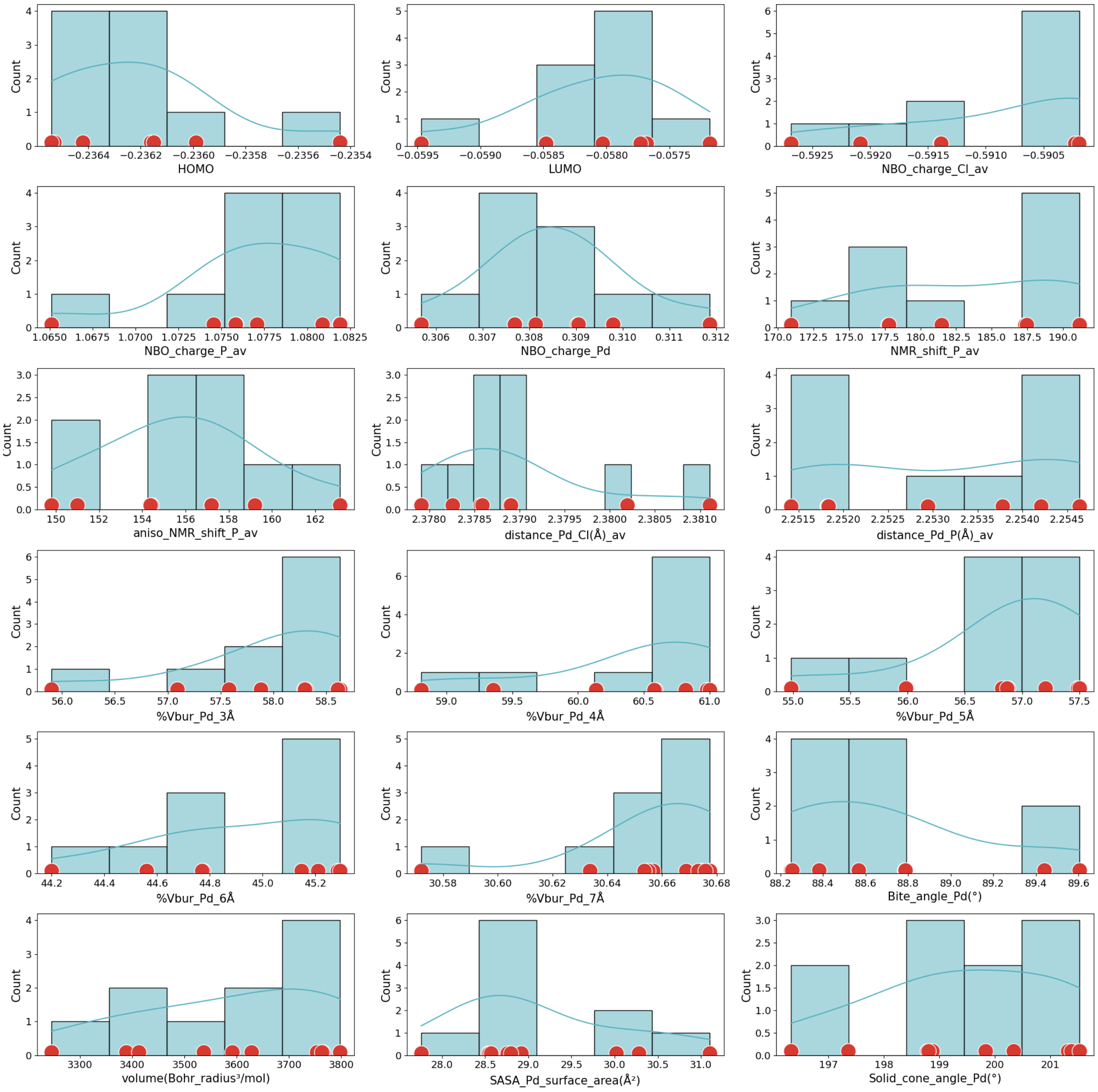

Figure S84: DFT-level feature distributions for ligand **pp000067**. Selected conformers by percent buried volume are indicted with red dots.

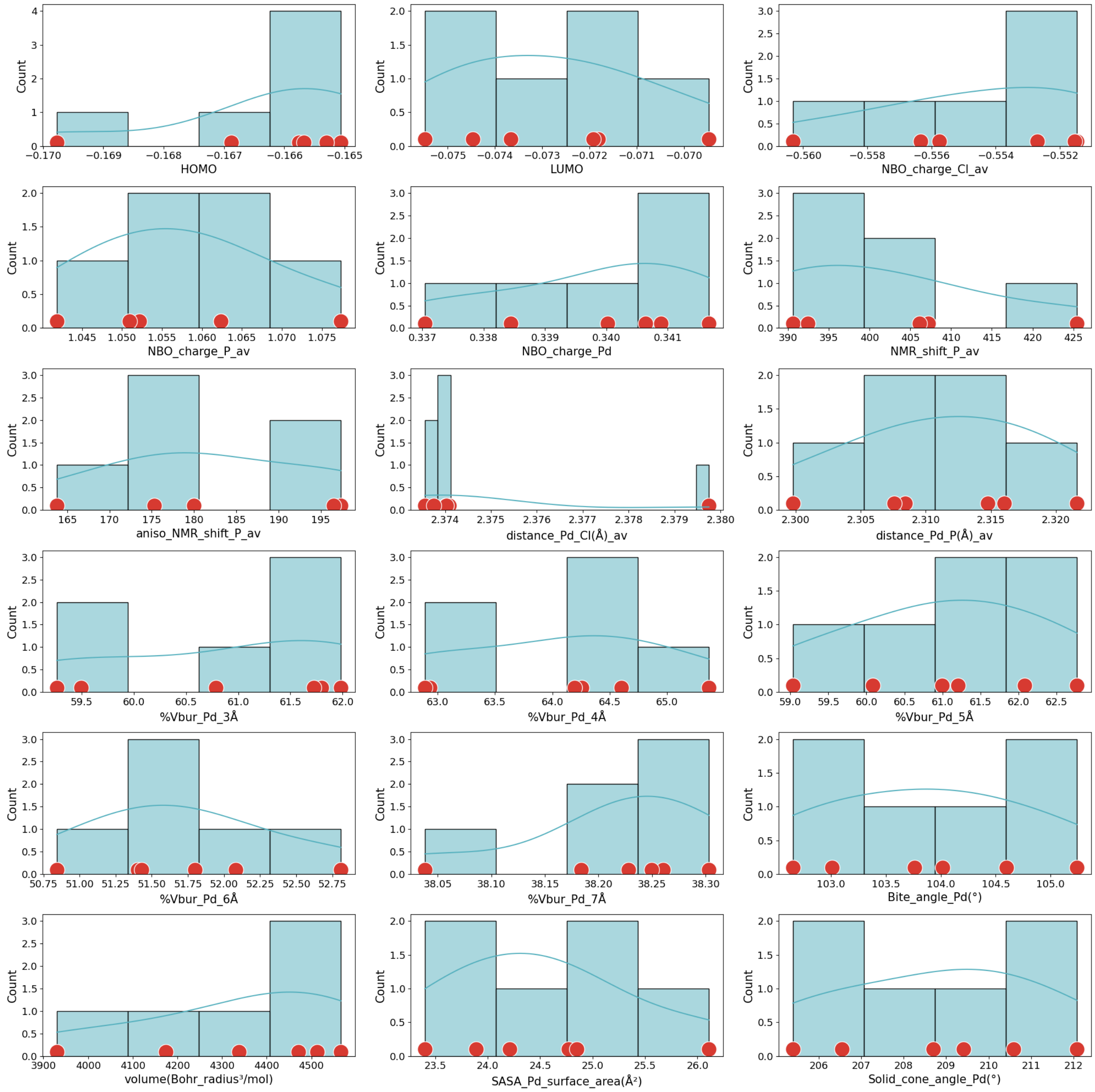

Figure S85: DFT-level feature distributions for ligand **pp000085**. Selected conformers by percent buried volume are indicted with red dots.

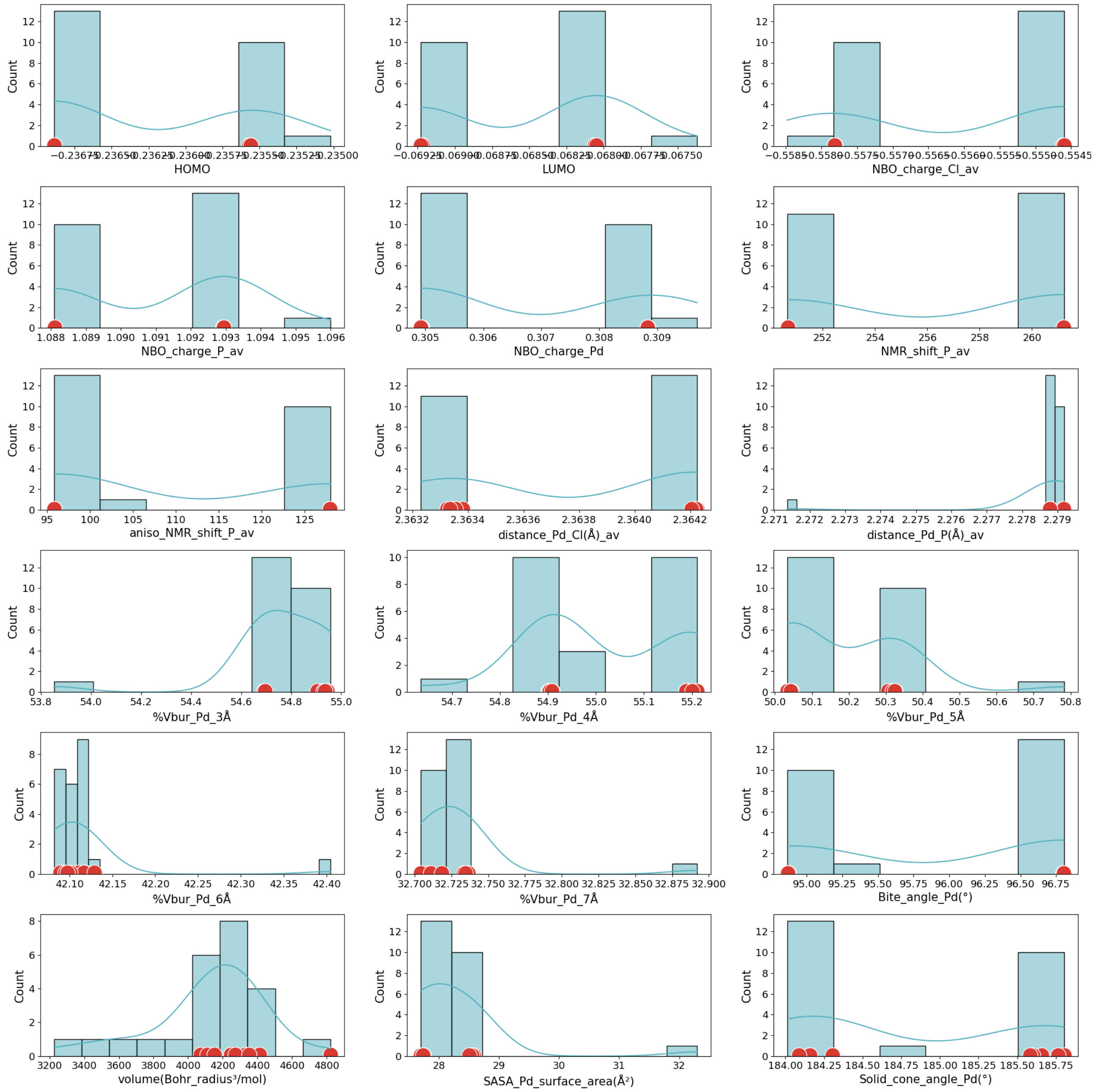

Figure S86: DFT-level feature distributions for ligand **pp000091**. Selected conformers by percent buried volume are indicted with red dots.

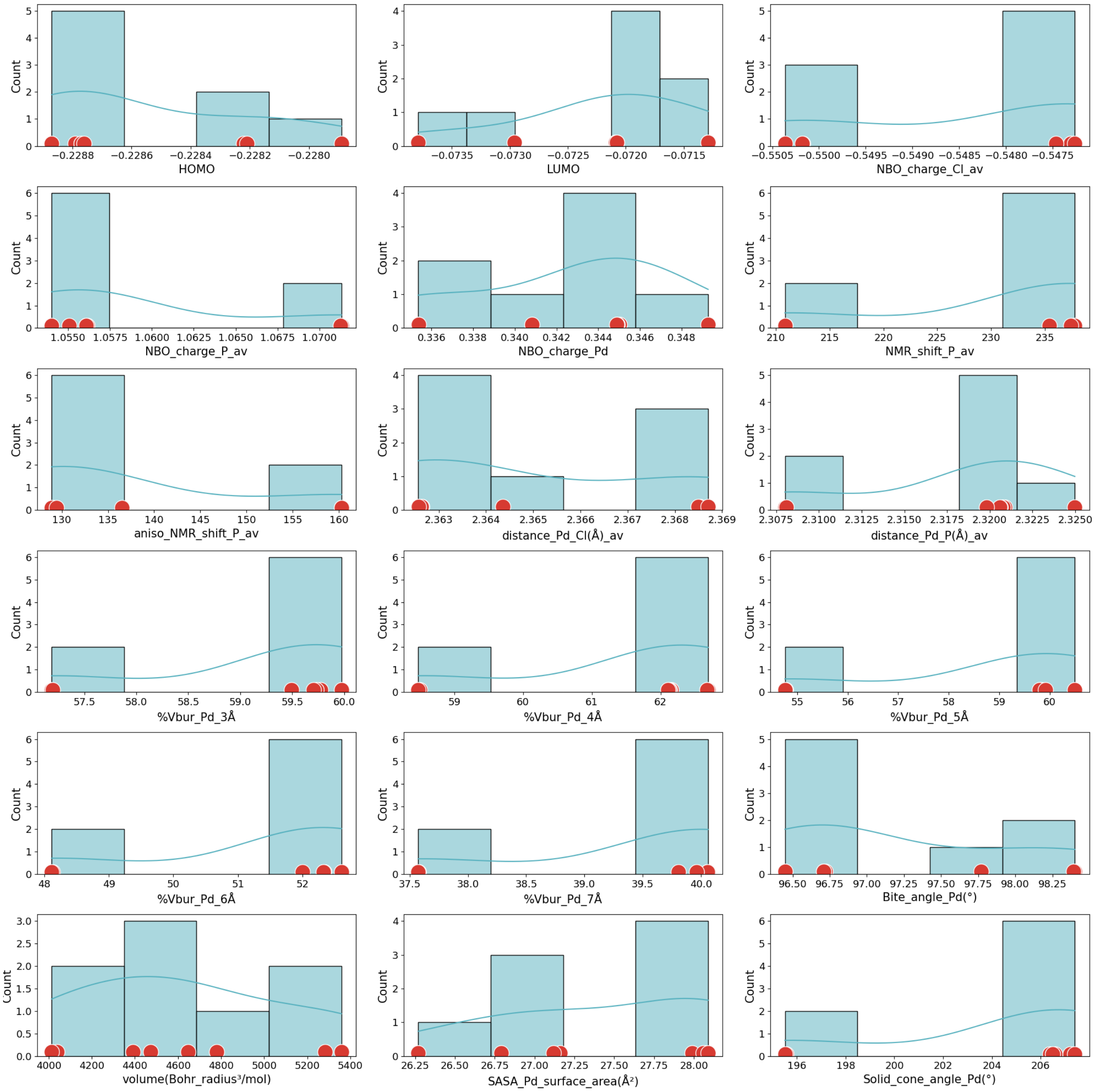

Figure S87: DFT-level feature distributions for ligand **pp000110**. Selected conformers by percent buried volume are indicted with red dots.

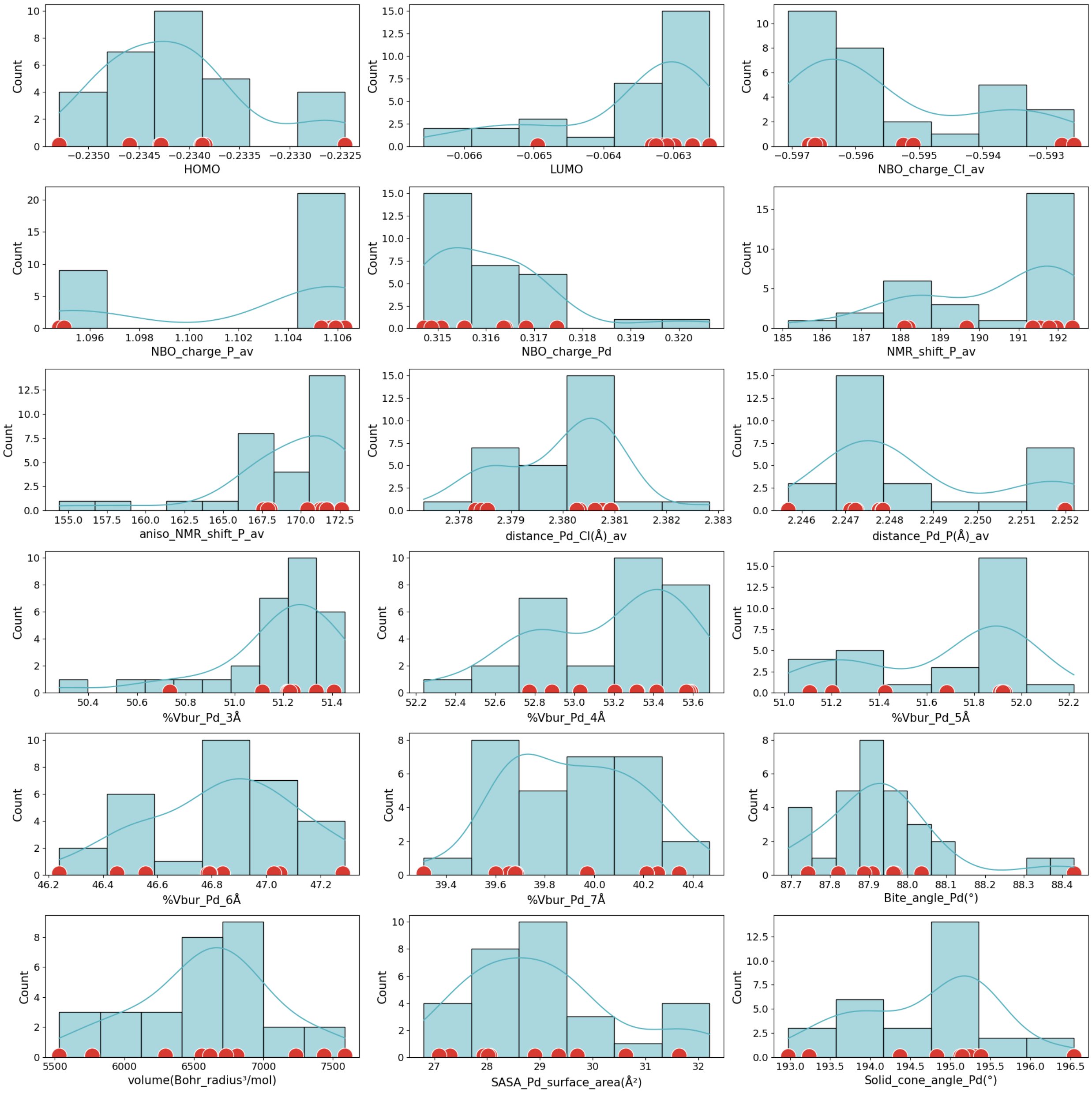

Figure S88: DFT-level feature distributions for ligand **pp000135**. Selected conformers by percent buried volume are indicted with red dots.

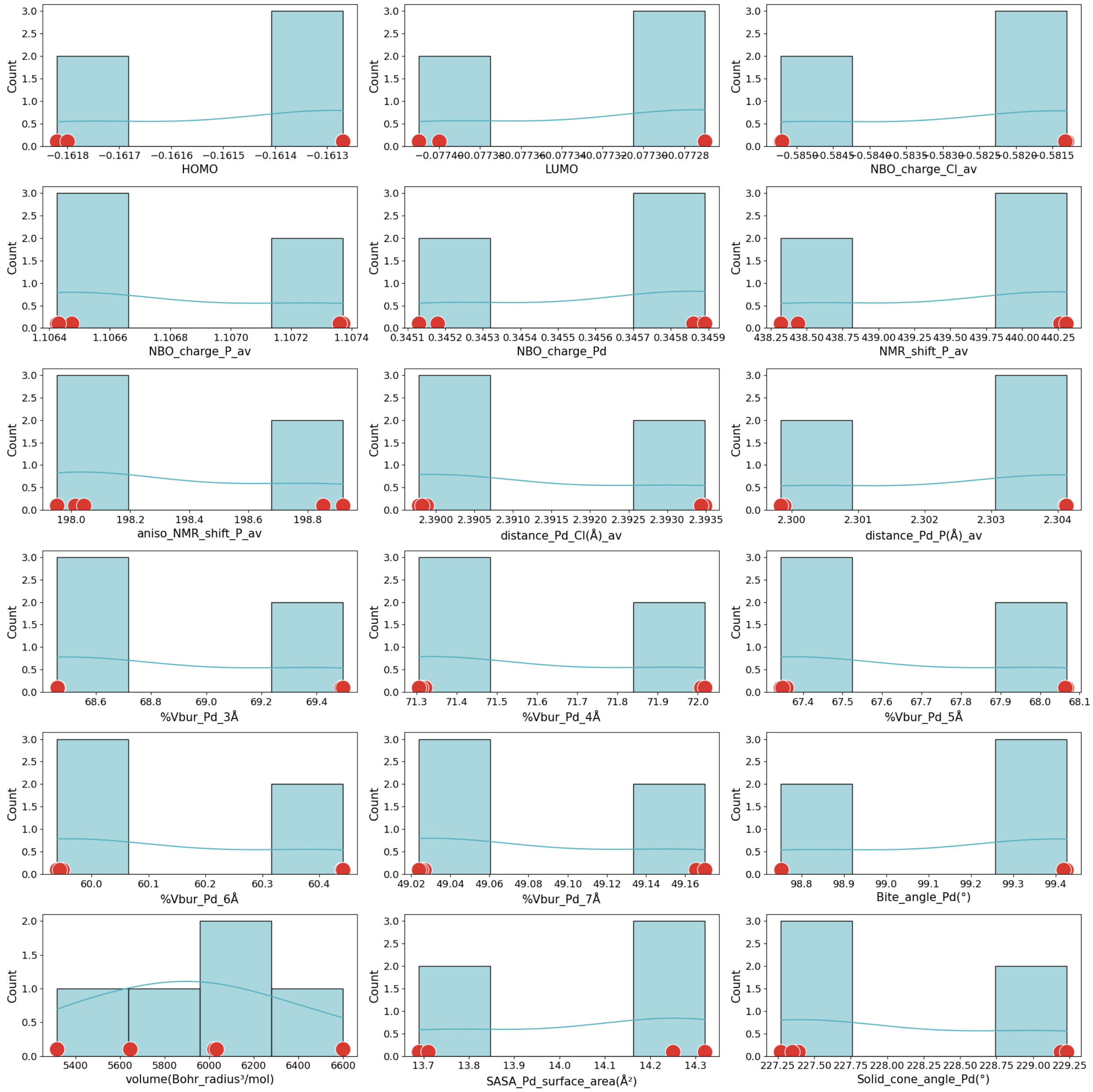

Figure S89: DFT-level feature distributions for ligand **pp000298**. Selected conformers by percent buried volume are indicted with red dots.

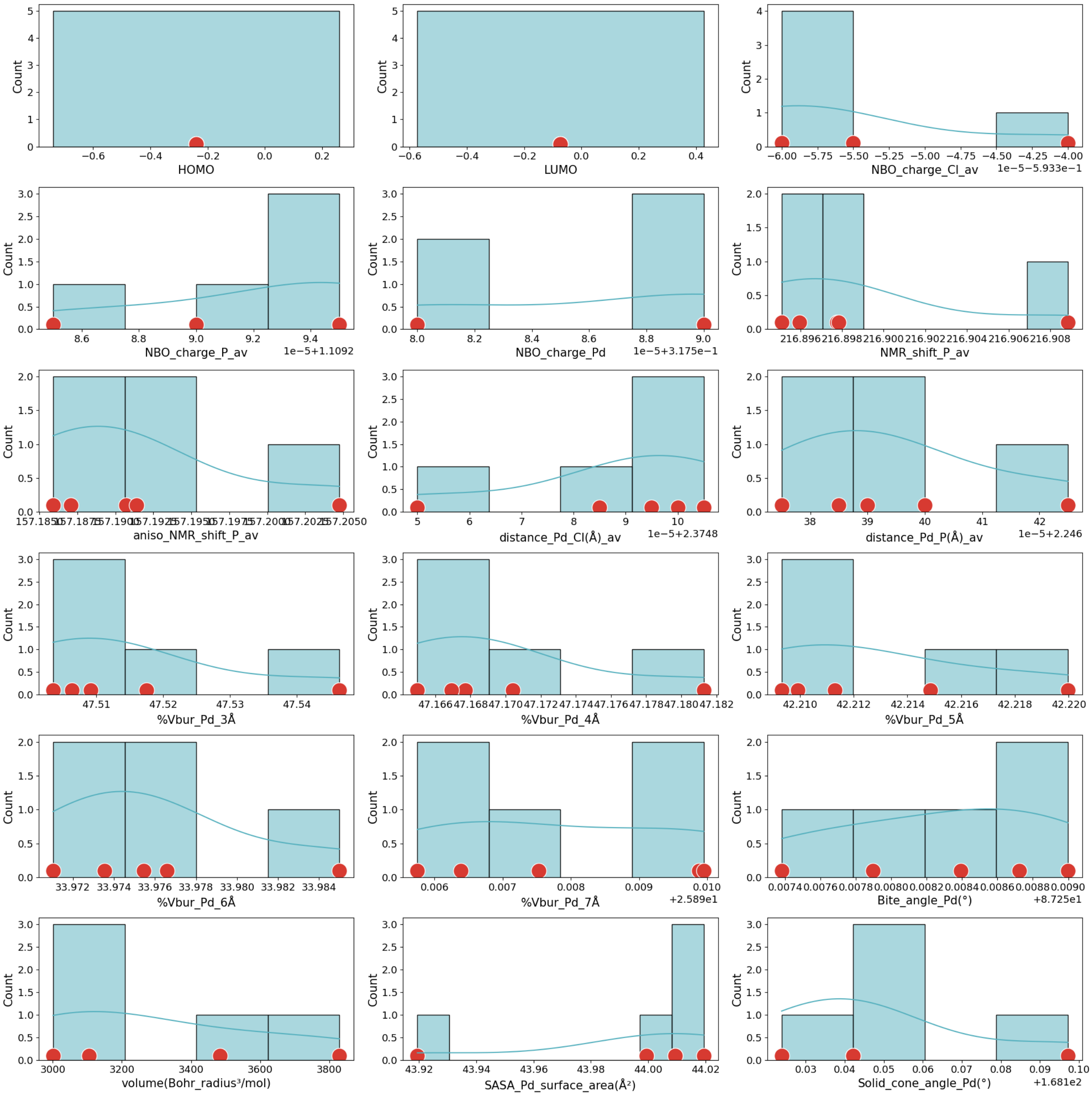

Figure S90: DFT-level feature distributions for ligand **pp000321**. Selected conformers by percent buried volume are indicted with red dots.

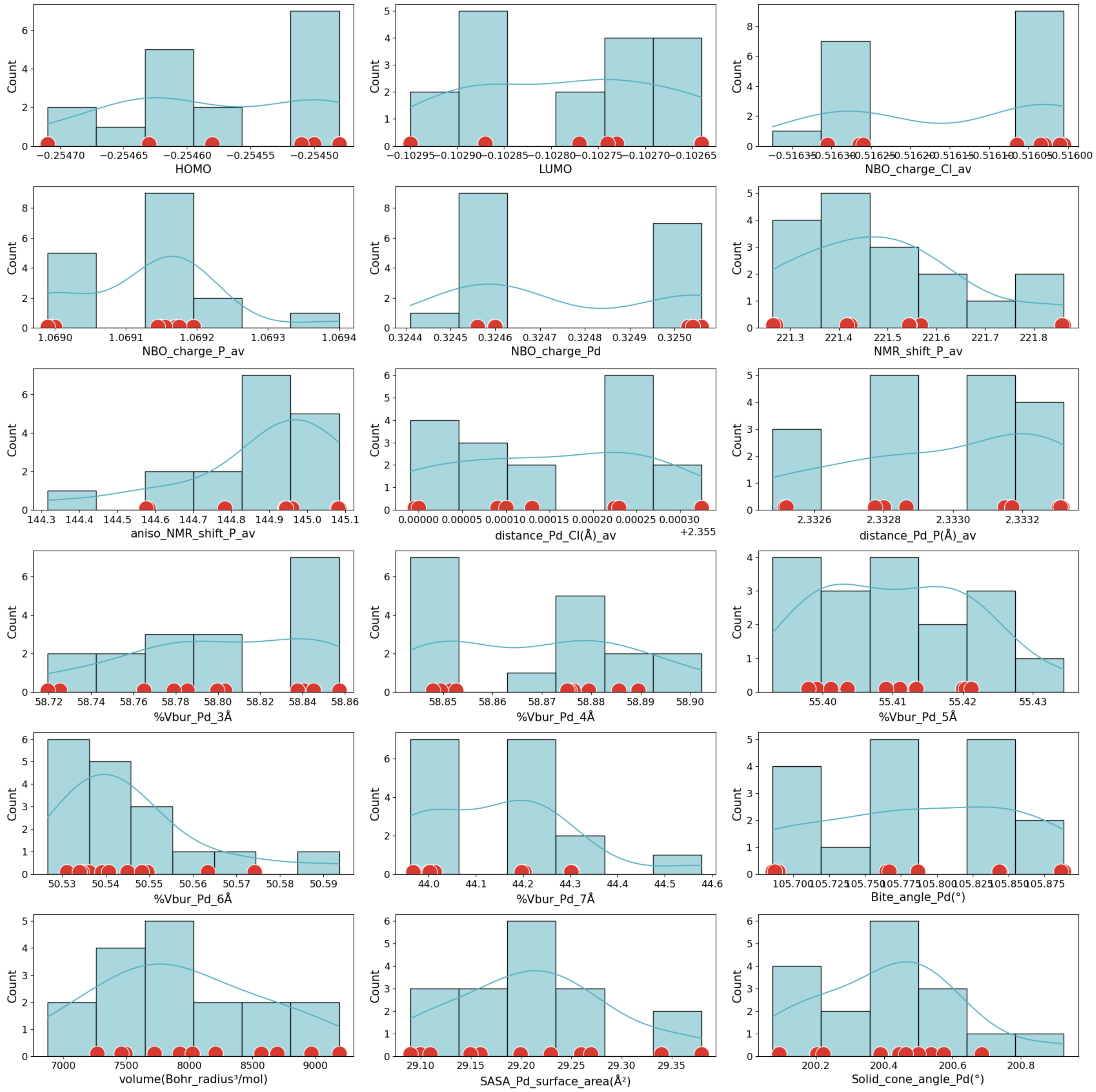

Figure S91: DFT-level feature distributions for ligand **pp000390**. Selected conformers by percent buried volume are indicted with red dots.

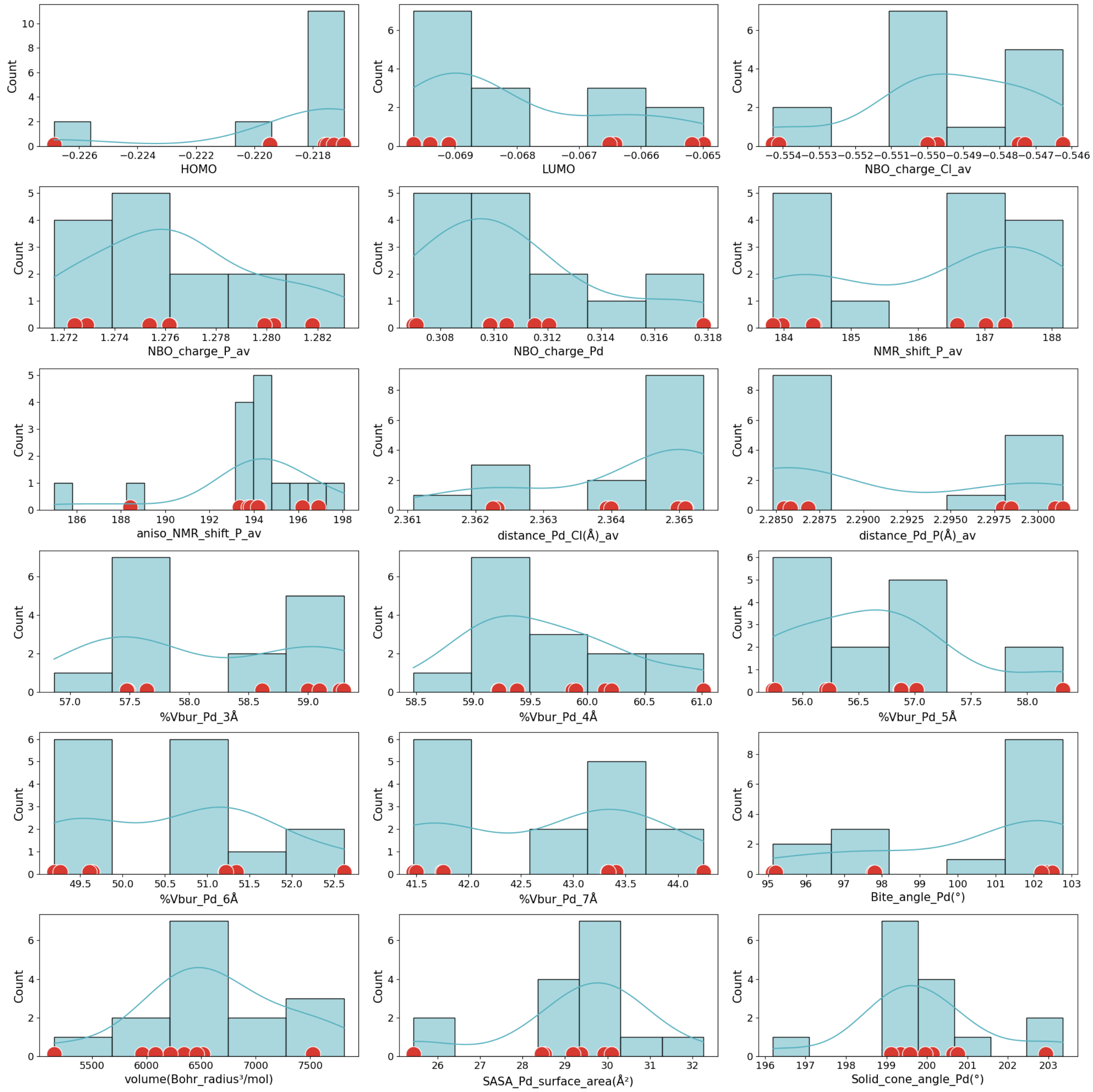

Figure S92: DFT-level feature distributions for ligand **pp000425**. Selected conformers by percent buried volume are indicted with red dots.

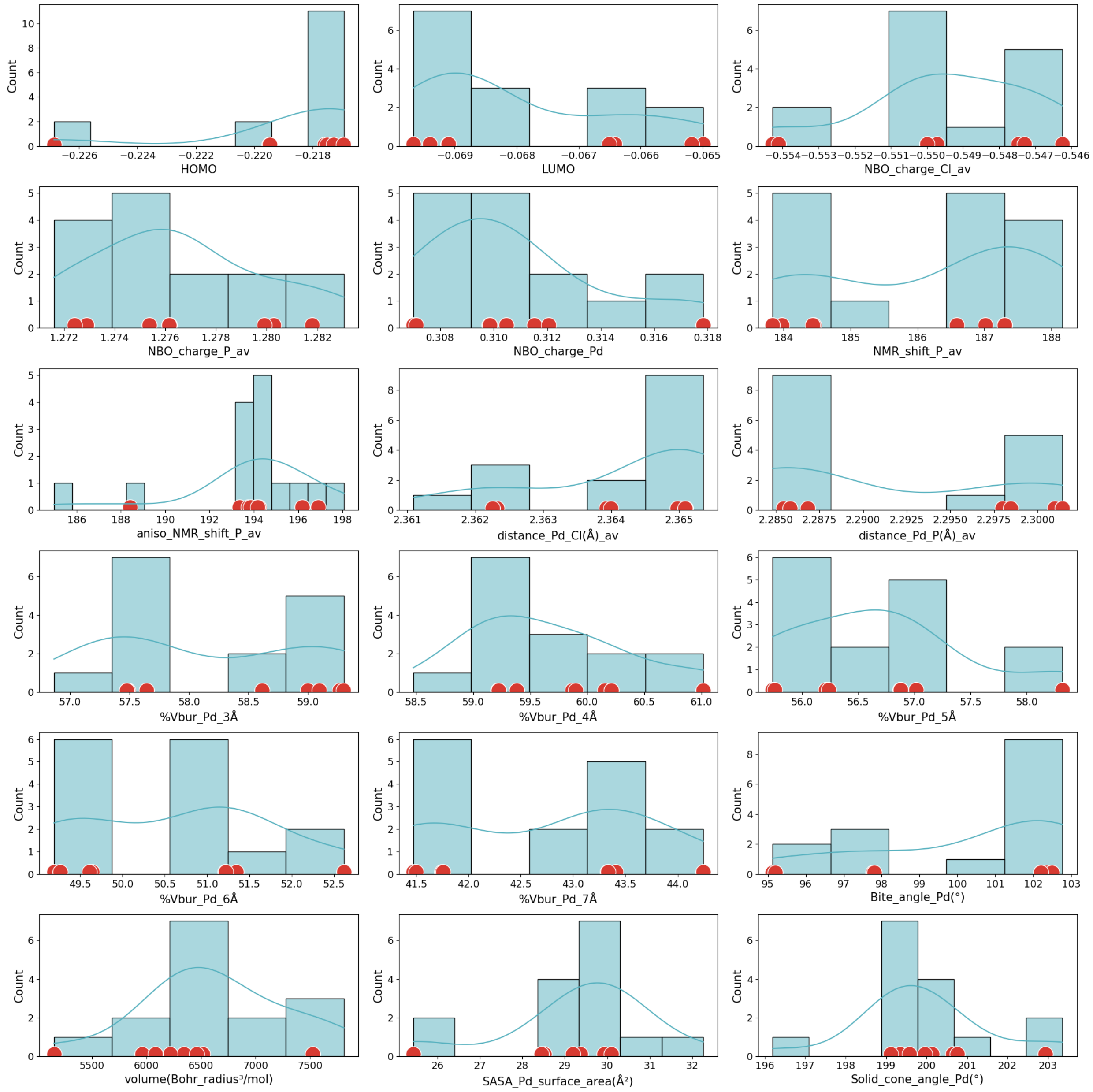

Figure S93: DFT-level feature distributions for ligand **pp000458**. Selected conformers by percent buried volume are indicted with red dots.

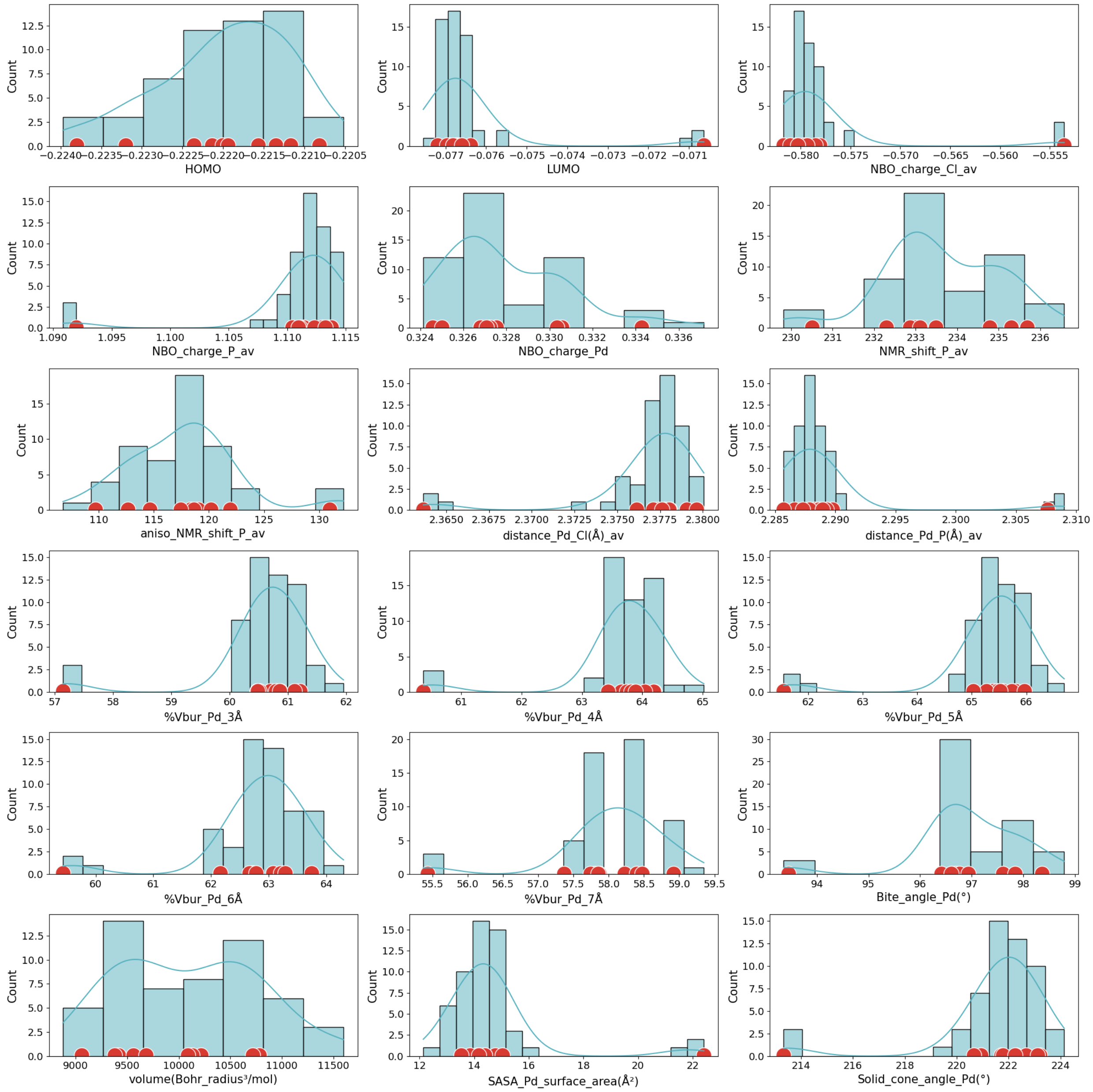

Figure S94: DFT-level feature distributions for ligand **pp000550**. Selected conformers by percent buried volume are indicted with red dots.

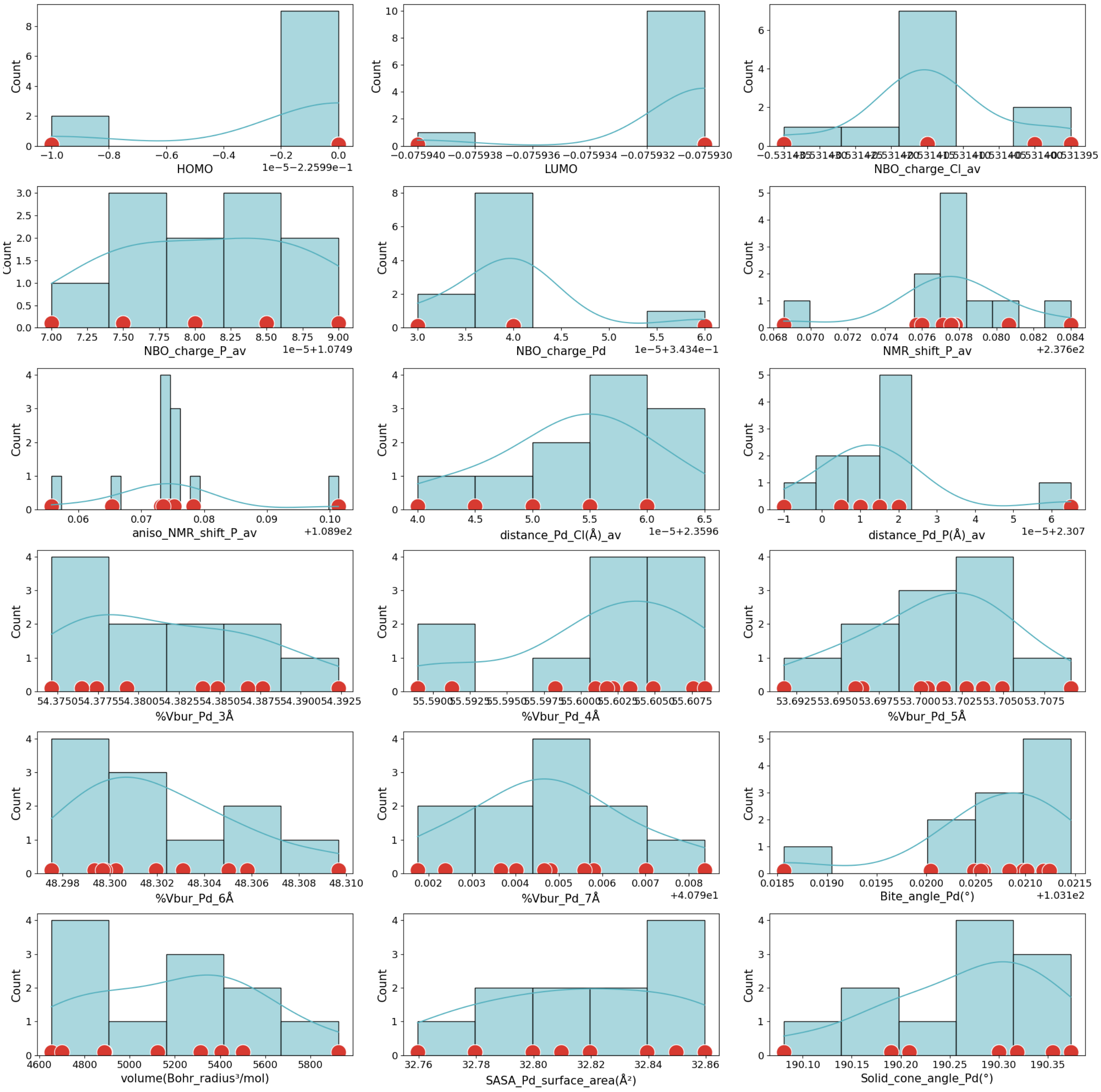

Figure S95: DFT-level feature distributions for ligand **pp000670**. Selected conformers by percent buried volume are indicted with red dots.

6 Conformer-weighted features in modelling campaigns

Data availability:

- DFT calculations used for the generation of the bisphosphine ligand parameters in both sets of calculations are available on Zenodo: https://doi.org/10.5281/zenodo.15690854.
- Data for the Hayashi-Heck cross-coupling regioselectivity and the sulfonamidamide aryl carbonylation are available in the `./modeling_data` folder of this publication's GitHub repository: https://github.com/SigmanGroup/BisphosphineConformerSelection.
- Scripts used to generate models are available in this publication's GitHub repository.

To examine the effects of including conformer-weighting into statistical modelling campaigns using bisphosphine ligands, two datasets were selected from work previously reported by our group. These datasets focus on the regioselectivity of a Hayashi-Heck cross-coupling reaction (Section 6.1)[1] and the enantioselectivity of a sulfonimidamide aryl carbonylation (Section 6.2).[30] Regioselectivity and enantiomeric ratios were converted to their thermally-weighted effective difference in transition state energies, $\Delta\Delta G^{\ddagger}$ (in kcal mol^{-1}) using Equation 5.

$$\Delta\Delta G^{\ddagger} = -RT\ln(\text{e.r.}) \tag{5}$$

All ligands described in the below sections were subjected to the computational workflow as described in this manuscript and subsequent featurization was performed as detailed in Section 1.4.

To describe the quality of the generated models below, the coefficient of determination (or R^2 score), the mean absolute error (MAE) and root mean squared error (RMSE) were used for both the training and test splits. Equation 6 describes the definition of R^2 ($R^2(y, \hat{y})$) used as implemented in SciPy[31] where $\hat{y}_i$ is the predicted value of the i-th datapoint, y_i is it's corresponding true value. The total number of datapoints is given as n, $\sum_{i=1}^{n}(y_i - \hat{y}_i)^2 =$

$\sum_{i=1}^{n} \epsilon_i^2$ (ϵ_i is how far from the regression line the i-th data point is) and $\bar{y} = \frac{1}{n}\sum_{i=1}^{n} y_i$. The R^2 value for the test set (R^2_{test}) was calculated according to Equation 7 which takes the training set mean $\bar{y}^{\text{train}}$ into account.[32]

$$R^2(y, \hat{y}) = 1 - \frac{\sum_{i=1}^{n}(y_i - \hat{y}_i)^2}{\sum_{i=1}^{n}(y_i - \bar{y})^2} \tag{6}$$

$$R^2_{\text{test}}(y^{\text{test}}, \hat{y}^{\text{test}}) = 1 - \frac{\sum_{i=1}^{n^{\text{test}}}(y_i^{\text{test}} - \hat{y}^{\text{test}})^2}{\sum_{i=1}^{n^{\text{test}}}((y_i^{\text{test}} - \bar{y}^{\text{train}})^2} \tag{7}$$

The mean absolute error $\text{MAE}(y, \hat{y})$ is given by Equation 8 estimated over the total number of data points n. The root mean squared error $\text{RMSE}(y, \hat{y})$ is given by Equation 9 estimated over the total number of data points n. Mean squared error (MSE) is the square of RMSE. These metrics were used as implemented in scikit-learn.[25] This analysis was equivalent for both the training and test sets.

$$\text{MAE}(y, \hat{y}) = \frac{1}{n}\sum_{i=0}^{n-1} |y_i - \hat{y}_i| \tag{8}$$

$$\text{RMSE}(y, \hat{y}) = \sqrt{\frac{1}{n}\sum_{i=0}^{n-1}(y_i - \hat{y}_i)^2} \tag{9}$$

6.1 Hayashi-Heck cross-coupling

6.1.1 Initial feature reduction

Using this calculation and featurization workflow, the number of features increased dramatically from 348 in the original workflow to 2088. This clearly meant that before attempting a MLR model search, the number of features had to be reduced. In order accomplish this, features were down selected by determining the conformational dependence of each feature within an ensemble and across the selection of ligands. This was done using a method previously reported by our group.[33] Conformer dependence for one ligand z is defined by Equation

10 where $\sigma_y^{\text{ensemble}}$ is the standard deviation of a feature for one ligand y in a conformer ensemble, $\bar{x}_y^{\text{ensemble}}$ is the mean of conformer ensemble feature values for one ligand, $\bar{x}^{\text{set}}$ is the mean feature value across all ligands in the data set and n is the total number of ligands. Conformer dependence across all ligands in the data set is taken from the mean of all values of z as shown in Equation 11.

$$z = \frac{\sigma_y^{\text{ensemble}}}{\sqrt{\frac{\sum(\bar{x}_y^{\text{ensemble}} - \bar{x}^{\text{set}})^2}{n-1}}} \tag{10}$$

$$\text{mean conformer dependence} = \frac{\sum z_i}{n} \tag{11}$$

Using this methodology, a threshold for conformer dependence of 0.2 was used to differentiate conformationally dependent and independent features. Features that classified as conformationally independent, or having little feature variance across a conformer ensemble, only the Boltzmann-weighted average feature was retained. For the regioselectivity data set described in Section 6.1.2, the original set of 2088 features was reduced to 1242. The enantioselectivity data set described in Section 6.2.2 was also reduced to a total 1242.

6.1.2 Regioselectivity dataset multivariate linear regression model

The regioselectivity data was divided into training and test sets in a 42:58 ratio (22:30 datapoints) as described in the original publication.[1] These data and the overall distribution are given in Table S4 and Figure S96, respectively.

Table S4: Hayashi-Heck regioselectivity data used for modelling.

Ligand ID	$\Delta\Delta G^{\ddagger}$ / kcal mol^{-1}	Set
**pp000006**	1.78	Train
**pp000008**	1.89	Train
**pp000009**	1.81	Train
**pp000015**	0.78	Train

**pp000027**	0.79	Train
**pp000033**	0.70	Train
**pp000036**	1.82	Test
**pp000039**	0.76	Train
**pp000052**	0.10	Train
**pp000061**	1.51	Test
**pp000062**	2.21	Test
**pp000068**	-0.28	Train
**pp000076**	1.66	Train
**pp000098**	1.30	Train
**pp000103**	0.10	Train
**pp000105**	0.80	Test
**pp000118**	1.14	Test
**pp000130**	2.08	Test
**pp000132**	2.78	Train
**pp000149**	0.27	Train
**pp000248**	2.75	Test
**pp000429**	-0.25	Test
**pp000436**	0.76	Test
**pp000437**	1.85	Test
**pp000443**	2.12	Test
**pp000444**	2.36	Test
**pp000446**	2.67	Test
**pp000447**	2.06	Test
**pp000449**	2.68	Test
**pp000458**	1.31	Test
**pp000462**	2.38	Test

**pp000473**	1.70	Test
**pp000477**	1.91	Test
**pp000478**	0.88	Test
**pp000479**	1.23	Train
**pp000485**	1.57	Test
**pp000515**	2.16	Test
**pp000532**	0.57	Test
**pp000533**	1.44	Test
**pp000534**	1.27	Test
**pp000544**	1.11	Test
**pp000565**	1.25	Test
**pp000567**	1.13	Test
**pp000569**	1.28	Test
**pp000570**	-0.18	Train
**pp000571**	-0.02	Train
**pp000572**	0.19	Train
**pp000573**	0.98	Train
**pp000574**	1.93	Train
**pp000575**	1.93	Train
**pp000576**	0.24	Train
**pp000597**	2.31	Test

The training and test feature sets were then scaled using the `StandardScaler` as implemented in scikit-learn. Following this, an exhaustive forward stepwise (up to three model components) linear regression model search was performed (using the `LinearRegression` function in scikit-learn) that carried all models forward from the previous steps. This resulted in the model given in the equation below:

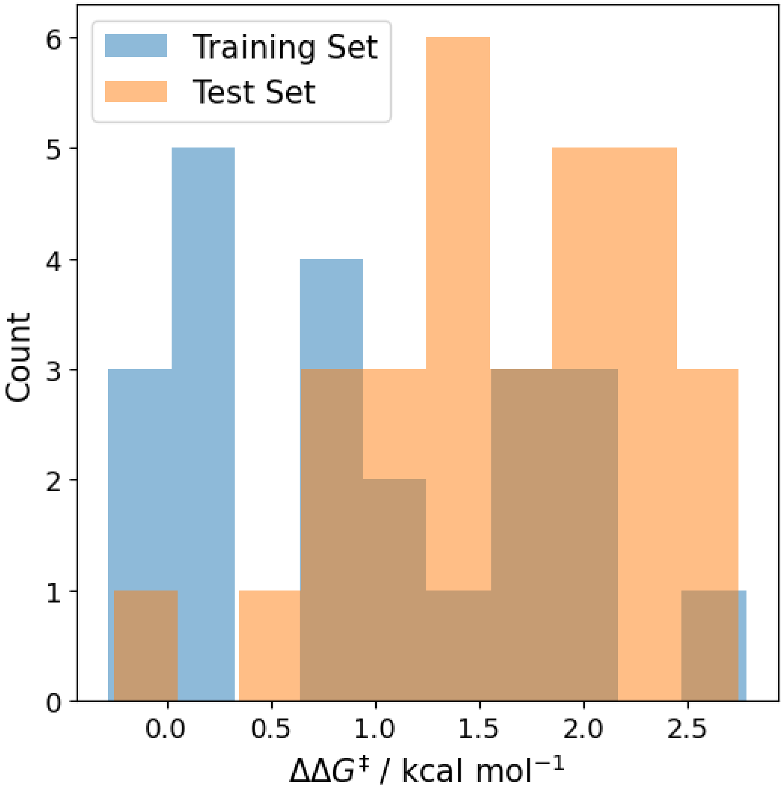

Figure S96: Hayashi-Heck regioselectivity data distribution.

$$
\begin{aligned}
\Delta\Delta G^{\ddagger} = \quad & 0.94 \\
& -0.89 \quad \cdot\texttt{P2\_NMR\_cmax} \\
& +0.45 \quad \cdot\texttt{P2\_Pd\_bond\_occ\_boltz} \\
& +0.66 \quad \cdot\texttt{NoPd\_P\_R2\_bond\_eng\_Max\_boltz}
\end{aligned}
$$

- `P2_NMR_cmax`: P2 ^{31}P NMR shift (ensemble maximum)
- `P2_Pd_bond_occ_boltz`: P2–Pd bond occupancy (Boltzmann-weighted average)
- `NoPd_P_R2_bond_eng_Max_boltz`: Free ligand max. P–R bond energy (Boltzmann-weighted average)

The conformational dependence of these features is assessed in Section 6.1.3.

This model is displayed in Figure S97. The training statistics for this model are: R^2 = 0.76; MAE = 0.30 kcal mol^{-1} and RMSE = 0.41 kcal mol^{-1}. The test statistics are: R^2 = 0.57; MAE = 0.51 kcal mol^{-1} and RMSE = 0.64 kcal mol^{-1}. Plots displaying the model errors in Figure 5 of the main manuscript were generated by taking the absolute differences in prediction and measured $\Delta\Delta G^{\ddagger}$ values and plotted as kernel density estimation distributions as implemented in seaborn.[29]

6.1.3 Analysis of feature conformer dependence

In addition to the conformational dependence of the ligands in Figure 5 of the manuscript, the equivalent information for all of the ligands is provided in Figures S98-S100.

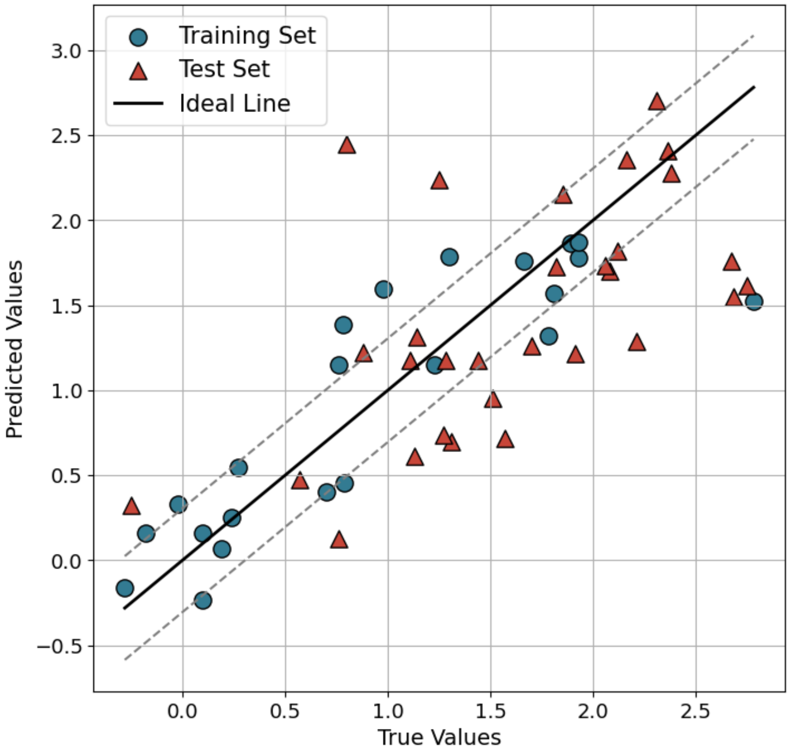

Figure S97: Hayashi-Heck cross-coupling regioselectivity multivariate linear regression (MLR) model.

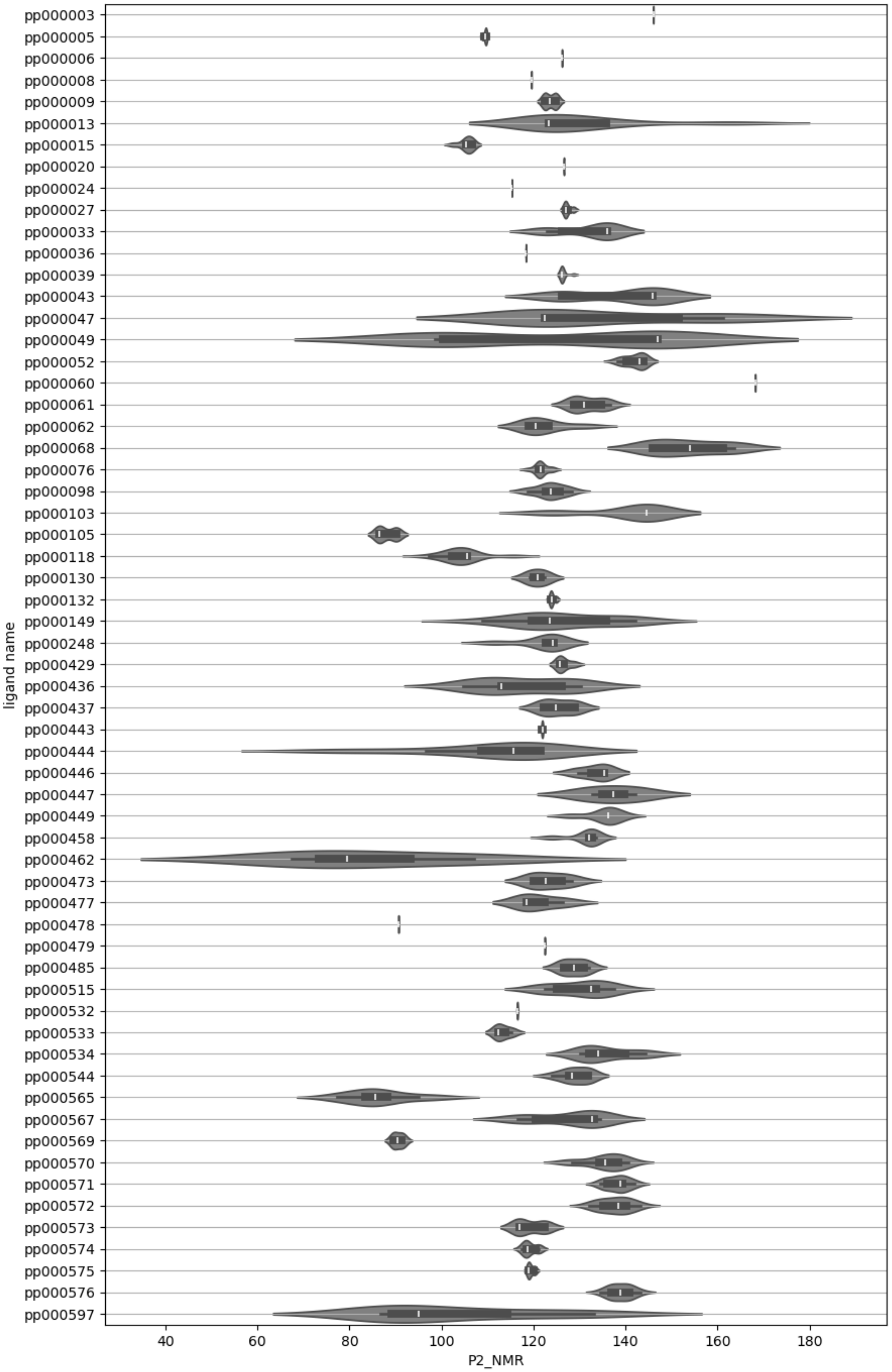

Figure S98: P2_NMR conformer dependence.

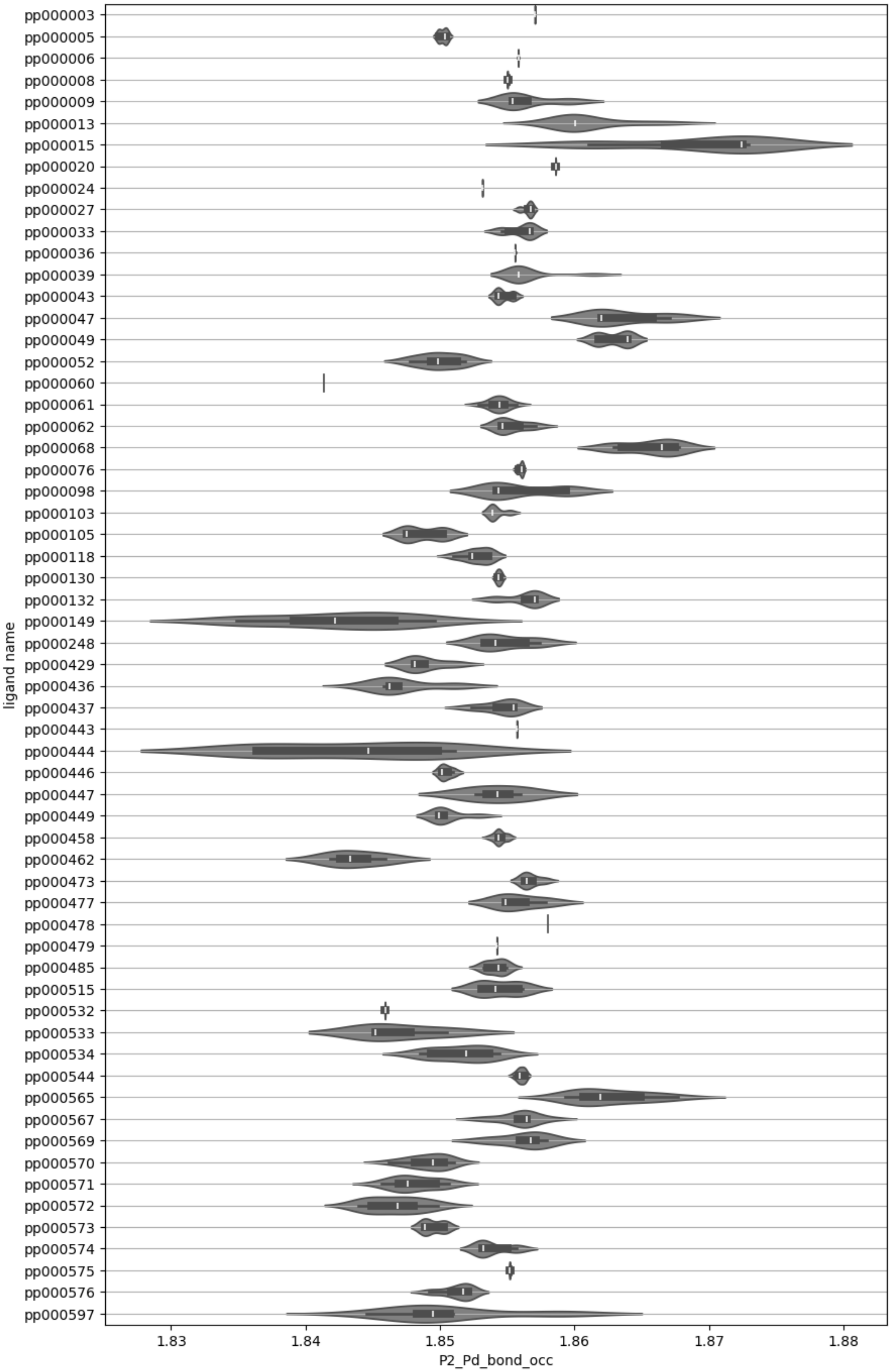

Figure S99: P2_Pd_bond_occ conformer dependence.

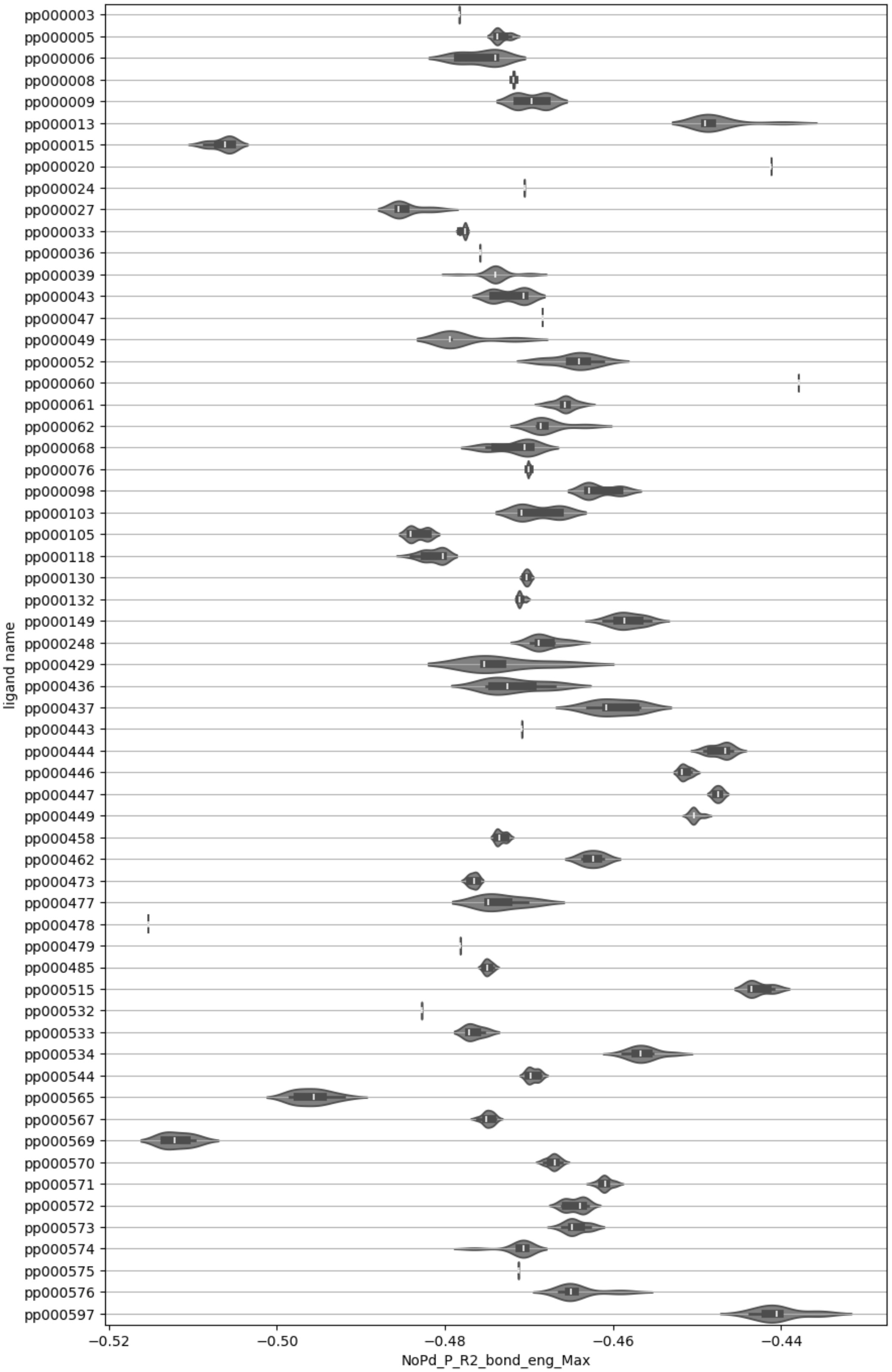

Figure S100: NoPd_P_R2_bond_eng_Max conformer dependence.

6.1.4 Mulivariate linear regression models with *only* lowest energy conformer features

The MLR model generated in Section 6.1.2 was regenerated using only the lowest energy conformer features. This model was used to help determine the effect of applying conformer-weighting to bisphosphine ligand features and is given in Figure S101 with the model equation below:

$$\begin{aligned}\Delta\Delta G^{\ddagger} = \quad & 0.94 \\ & -0.61 \quad \cdot\texttt{P2\_NMR} \\ & +0.37 \quad \cdot\texttt{P2\_Pd\_bond\_occ} \\ & +0.34 \quad \cdot\texttt{NoPd\_P\_R2\_bond\_eng\_Max}\end{aligned}$$

The training statistics for this model were: $R^2 = 0.48$; MAE = 0.48 kcal mol^{-1}; RMSE = 0.60 kcal mol^{-1}. The test statistics for this model were: $R^2 = 0.21$; MAE = 0.70 kcal mol^{-1}; RMSE = 0.84 kcal mol^{-1}.

In addition to regenerating the model using the equivalent terms used in 6.1.2, the model search was re-run with only lowest energy conformer features and the resulting model is shown in Figure S102 with the equation below:

$$\begin{aligned}\Delta\Delta G^{\ddagger} = \quad & 0.94 \\ & -1.02 \quad \cdot\texttt{Aniso\_P\_NMR\_Avg} \\ & -0.69 \quad \cdot\texttt{P\_Pd\_bond\_occ\_range} \\ & +0.44 \quad \cdot\texttt{P1-Pd\_distance}\end{aligned}$$

The training statistics for this model were: $R^2 = 0.91$; MAE = 0.19 kcal mol^{-1}; RMSE

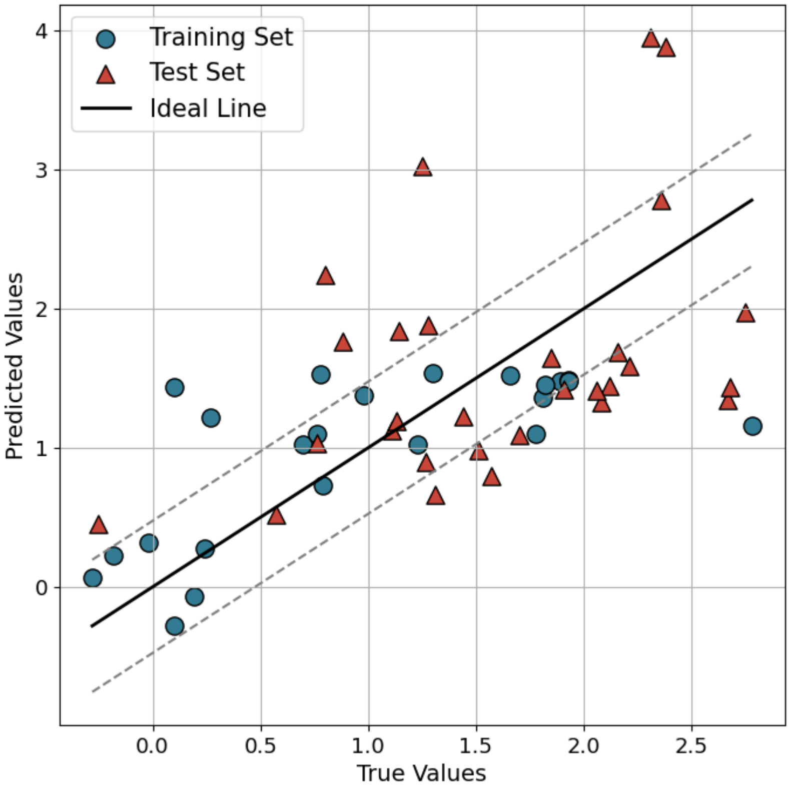

Figure S101: Hayashi-Heck cross-coupling regioselectivity multivariate linear regression (MLR) model using lowest energy conformer features.

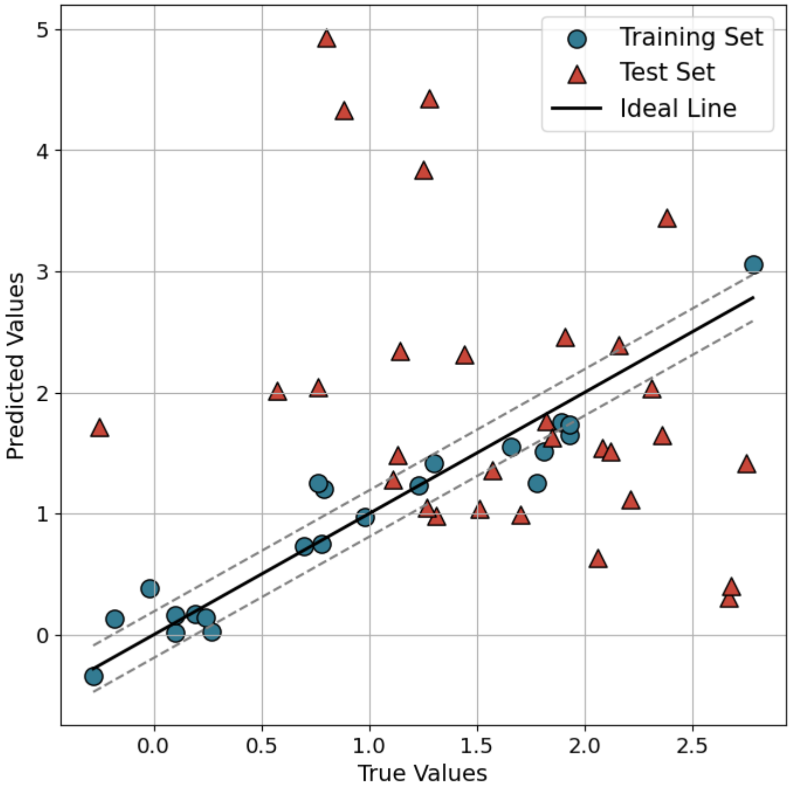

Figure S102: Hayashi-Heck cross-coupling regioselectivity multivariate linear regression (MLR) model using lowest energy conformer features.

= 0.25 kcal mol^{-1}. The test statistics for this model were: $R^2 = -1.64$; MAE = 1.18 kcal mol^{-1}; RMSE = 1.58 kcal mol^{-1}.

6.1.5 Model comparison and discussion

For all the models genererated in Sections 6.1.2-6.1.4, the test set statistics are summarised in Table S5 for comparison. The MLR model using conformer-weighted features (entry 1) compared to the MLR models generated using lowest energy conformer features (entries 2 and 3) shows greater predictive power, exemplified by the test statistics.

Table S5: Hayashi-Heck cross-coupling regioselectivity model comparison from Sections 6.1.2-6.1.4. MLR = multivariate linear regression.

Model	Model type	Figure	Test R^2	Test MAE / kcal mol^{-1}	Test RMSE / kcal mol^{-1}
**1**	**MLR**	S97	**0.57**	**0.51**	**0.64**
2	MLR	S101	0.21	0.70	0.84
3	MLR	S102	-1.64	1.18	1.58

6.2 Sulfonimidamide aryl carbonylation

6.2.1 Data curation

A selection of ligands (19) from the original sulfonamidamide enantioselectivity ligand screen were subjected to quality control at Genentech using ^{31}P NMR spectroscopy to identify the amount of ligand that had been oxidized (to the phosphine oxide). From this analysis, the phosphine ligands were defined as being unoxidised (>90% unoxidised) and oxidised (<90% unoxidised). These data are provided in Table S6 and displayed on the enantioselectivity data as histograms (Figure S103). Figure S103 clearly shows (except for one ligand), that the bisphosphine ligands that had been oxidised are centered around $\Delta\Delta G^{\ddagger} = 0$, *i.e.*, not enantioselective. It was speculated that, while the presence of a given quantity of oxidised bisphosphine may give reactivity towards the sulfonamidamide aryl carbonylation, this may

lead to diminished enantioselectivity. As a result, the eight ligands identified as being oxidised were removed from subsequent analysis.

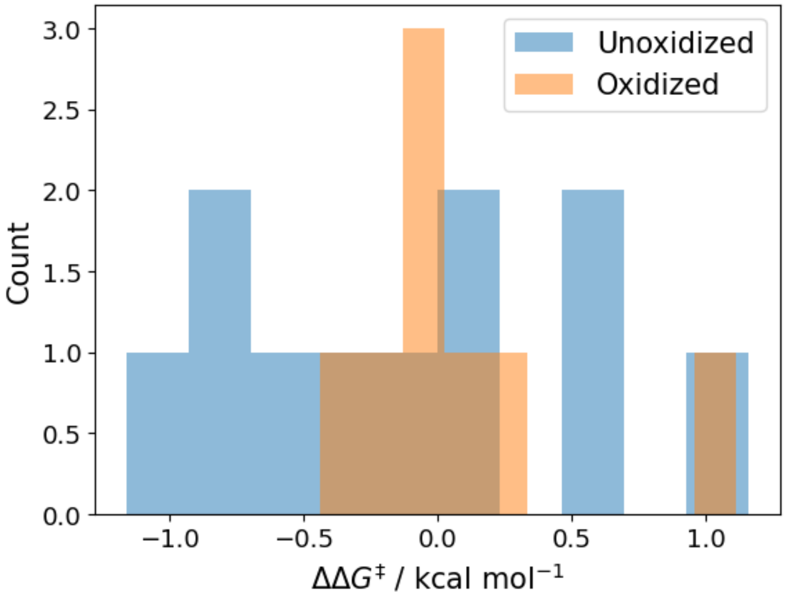

Figure S103: Histograms of enantioselectivity data ($\Delta\Delta G^{\ddagger}$) in the context of oxidised and non-oxidised bisphosphine ligands from ^{31}P NMR spectroscopic analysis.

6.2.2 Initial model search

The decision tree regression model was initially constructed using all xxx of the conformer-weighted features as implemented in scikit-learn. The data was split into training and test sets randomly with a ratio of training:test 33:9 (`test_size=0.2`). A histogram showing the overall spread of data as well as the visualization of the train:test split is given in Figure S104. Additionally, the data is tabulated in Table S6. The model hyperparameters (`max_depth`, `min_samples_split` and `min_samples_leaf`) were tuned using a randomized grid search with cross-validation (five folds using the RMSE metric). Hyperparameter effects are described in full in Section 6.2.5. After building this initial model, features were reduced by discarding those with a negative permutation feature importance in the test set (using test RMSE). This reduced the number of features down to four and were used for subsequent modeling. The features used were:

Table S6: Oxidised bisphosphine data from ^{31}P NMR spectroscopic analysis (ligands are ordered by increasing $\Delta\Delta G^{\ddagger}$).

Ligand	$\Delta\Delta G^{\ddagger}$ / kcal mol^{-1}	% Unoxidised (by ^{31}P NMR)	> 90% Unoxidised?
**pp000004**	-1.16	100	Yes
**pp000045**	-0.74	99.9	Yes
**pp000076**	-0.70	100	Yes
**pp000082**	-0.61	93.5	Yes
**pp000021**	-0.44	85.4	No
**pp000068**	-0.29	99.9	Yes
**pp000285**	-0.16	67.1	No
**pp000305**	-0.05	50.0	No
**pp000057**	-0.02	50.0	No
**pp000022**	-0.02	99.0	Yes
**pp000096**	0.00	100	Yes
**pp000001**	0.00	99.0	Yes
**pp000015**	0.02	0.00	No
**pp000270**	0.05	50.0	No
**pp000041**	0.19	88.5	No
**pp000052**	0.64	100	Yes
**pp000008**	0.64	98.0	Yes
**pp000023**	1.11	89.3	No
**pp000085**	1.16	99.9	Yes

- Cl_NBO_Max_boltz: The Boltzmann-weighted average of the maximum Cl ligand NPA charge.
- P_Pd_bond_occ_Av_boltz: The Boltzmann-weighted average of the average P–Pd bond occupancy.
- angle_R2P2Cback_boltz: The Boltzmann-weighted average of the R2P2Cback angle.
- NoPd_P_R1_bond_occ_Min_cmin: The ensemble minimum P–R1 bond occupancy (minimum)

The conformational dependence of these features is assessed in Section 6.2.6.

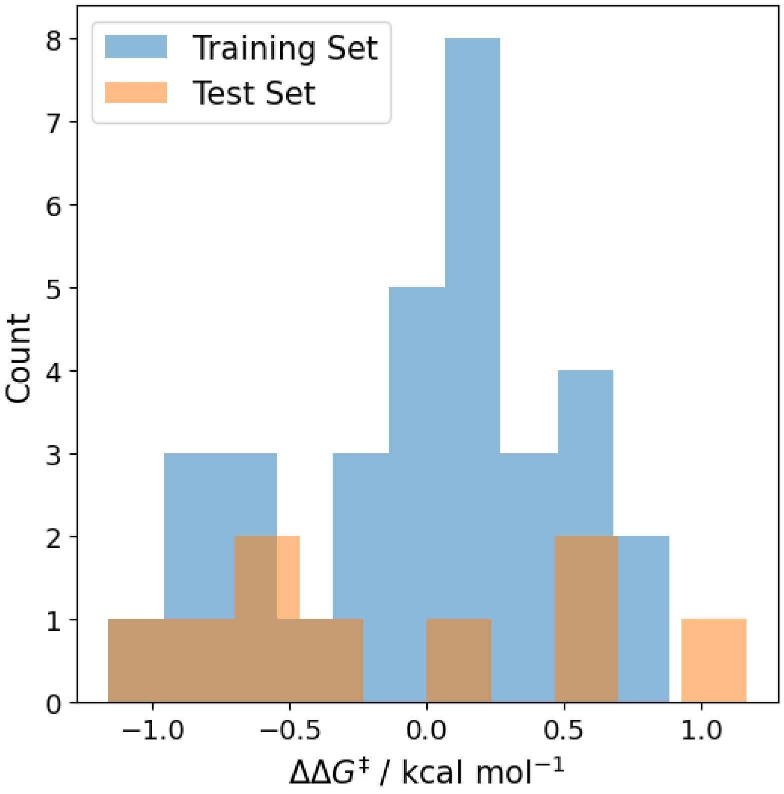

Figure S104: Sulfonimidamide aryl carbonylation enantioselectivity data histogram.

Table S7: Sulfonamidamide aryl carbonylation enantioselectivity data used for modelling.

Ligand ID	$\Delta\Delta G^{\ddagger}$ / kcal mol^{-1}	Set
**pp000061**	-0.81	Training
**pp000027**	0.34	Training
**pp000016**	-1.16	Training
**pp000101**	0.52	Training
**pp000006**	0.88	Training
**pp000134**	-0.92	Training
**pp000012**	-0.41	Training
**pp000029**	0.49	Training
**pp000043**	0.023	Training
**pp000071**	-0.047	Training
**pp000105**	0.39	Training
**pp000003**	-0.07	Training
**pp000115**	0.24	Training
**pp000025**	0.77	Training
**pp000130**	-0.19	Training
**pp000078**	0.093	Training
**pp000050**	-0.26	Training
**pp000056**	0.19	Training
**pp000008**	0.64	Training
**pp000068**	-0.29	Training
**pp000103**	0.070	Training
**pp000024**	0.14	Training
**pp000129**	-0.84	Training
**pp000495**	0.44	Training
**pp000019**	0.070	Training

**pp000111**	0.14	Training
**pp000083**	0.55	Training
**pp000076**	-0.70	Training
**pp000082**	-0.61	Training
**pp000001**	0.00	Training
**pp000045**	-0.74	Training
**pp000114**	0.26	Training
**pp000022**	-0.023	Training
**pp000085**	1.16	Test
**pp000063**	-0.39	Test
**pp000077**	0.49	Test
**pp000017**	-0.47	Test
**pp000096**	0.00	Test
**pp000069**	-0.49	Test
**pp000004**	-1.16	Test
**pp000052**	0.64	Test
**pp000055**	-0.84	Test

6.2.3 Enantioselectivity decision tree regression model and feature importance

Using the features described above, and the same training:test split, the final decision tree regression model was generated with the enantioselectivity data (as $\Delta\Delta G^{\ddagger}$). The final hyperparameters used were: `min_samples_split=3`, `min_samples_leaf=2`, `max_depth=6`, `random_state=10`. The model regression plot is given in Figure S105 and the decision tree used for the regression is shown in Figure S106.

The training statistics for this model are: $R^2 = 0.96$; MAE = 0.08 kcal mol^{-1} and RMSE = 0.10 kcal mol^{-1}. The test statistics are: $R^2 = 0.45$; MAE = 0.36 kcal mol^{-1} and RMSE = 0.53 kcal mol^{-1}. Plots displaying the model errors in Figure X of the main manuscript were

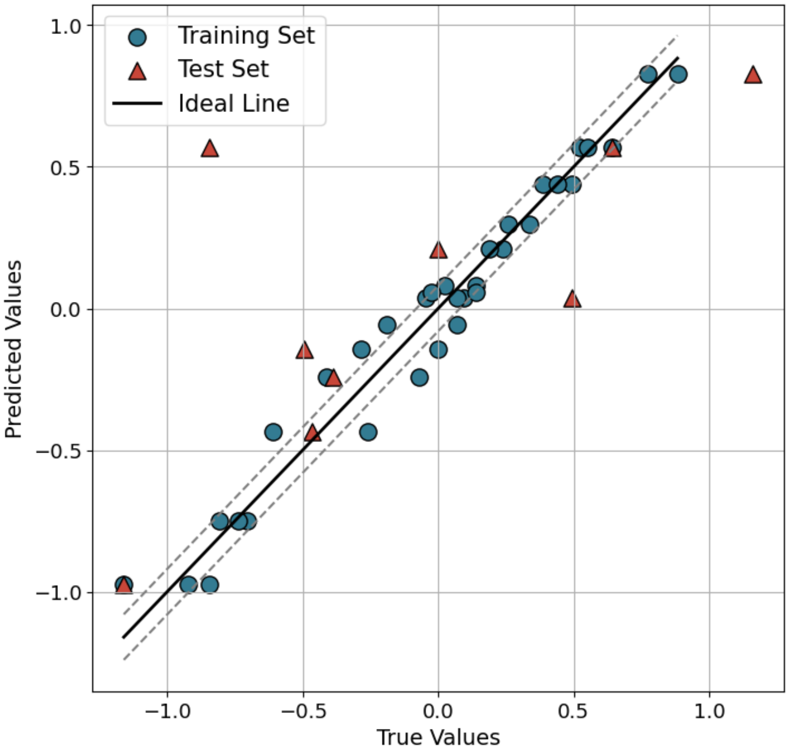

Figure S105: Sulfonimidamide aryl carbonylation enantioselectivity decision tree regression model.

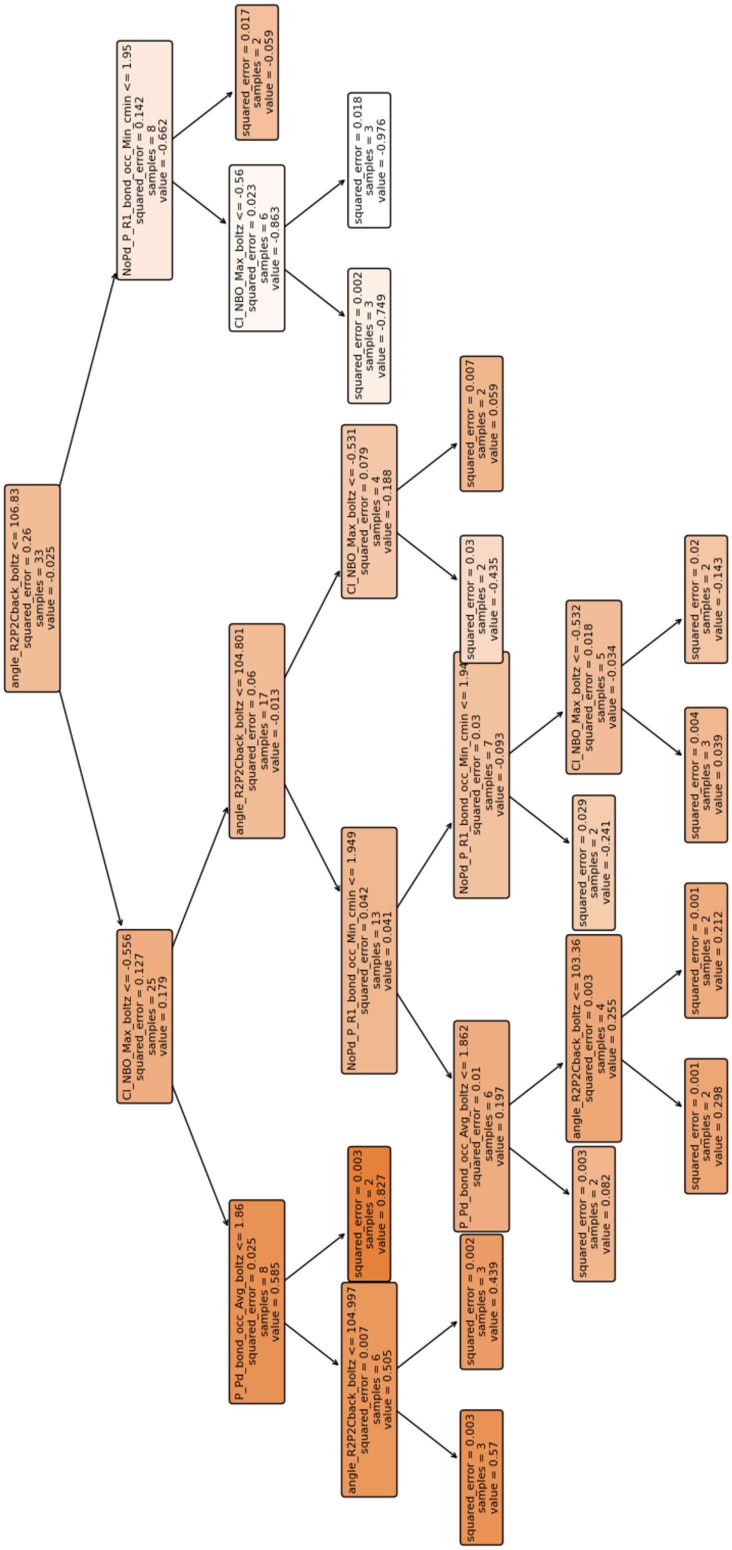

Figure S106: Sulfonimidamide aryl carbonylation enantioselectivity decision tree.

generated by taking the absolute differences in prediction and measured $\Delta\Delta G^{\ddagger}$ values and plotted as kernel density estimation distributions as implemented in seaborn.[29] The reduced statistics for the test set are due to one outlier in the test set (ligand **pp000055**). This was exemplified by removing that ligand altogether to give improved test statistics of $R^2 = 0.86$, MAE = 0.22 kcal mol^{-1} and RMSE = 0.22 kcal mol^{-1}.

Having developed a decision tree regression model with the sulfonamidamide enantioselectivity data, we next examined the importance of the four features used in the model, In addition to illustrating the actual decision tree in Figure S106, the permutation feature importance (PFI) and SHAP analysis[34] was also used. These analyses are shown for the test data in Figures S107 and S108, respectively. Both analyses indicated that `Cl_NBO_Max_boltz` and `angle_R2P2Cback_boltz` are the two most important features in the decision tree regression model. The two least important features were `NoPd_P_R1_bond_occ_Min_cmin` and `P_Pd_bond_occ_Avg_boltz`, of which the exact order of importance changes in the PFI and SHAP analyses.

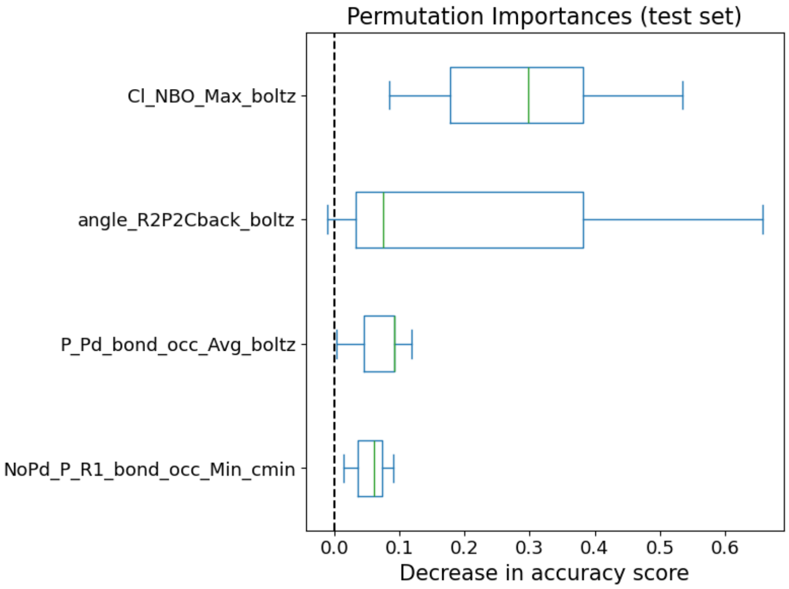

Figure S107: Permutation feature importance for the decision tree regression model.

We were curious whether examining the feature importance could help to explain the origin of ligand **pp000055** being an outlier. Firstly, the SHAP analyses were compared with

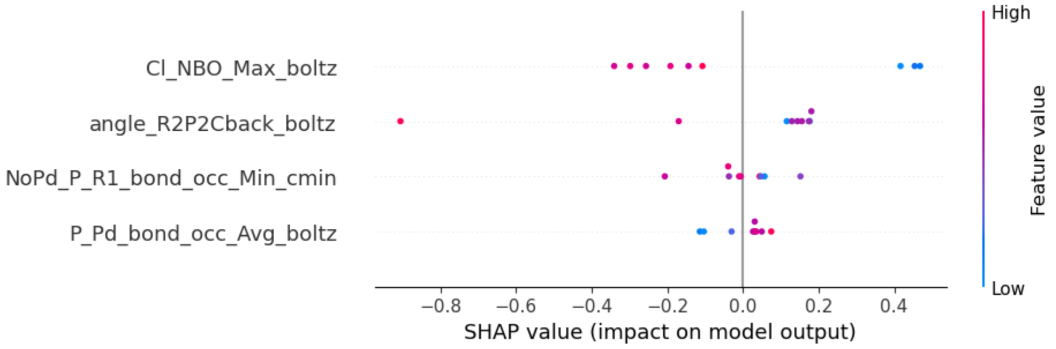

Figure S108: SHAP analysis for the decision tree regression model.

this ligand and the ligand with the lowest model error (the full SHAP analysis of each test data point is provided in the accompanying Jupyter notebook). Interestingly, for these two data points, there is an inverse SHAP value for each of the four features. The feature values for both data points are however very similar.

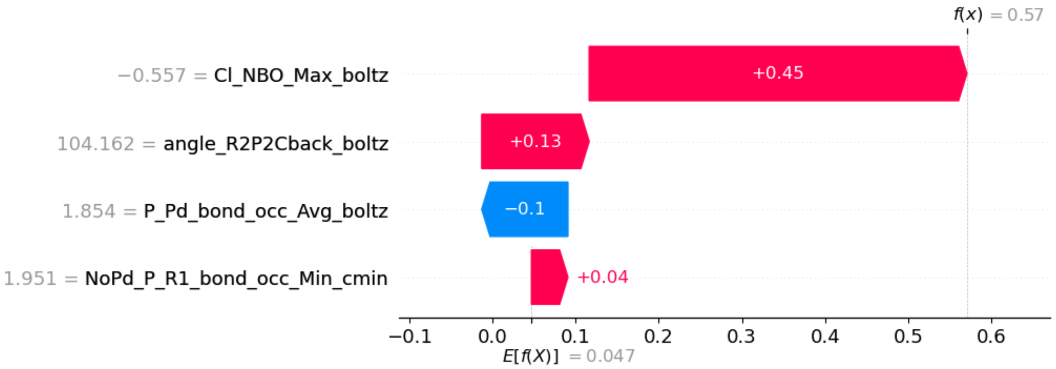

Figure S109: SHAP analysis from the decision tree regression model for the ligand with the highest error (**pp000055**).

Further to this, the decision path (shown graphically in Figure S106) followed for this data point was examined. This was as follows:

1. *Node 0:* `angle_R2P2Cback_boltz` = 104.16. Is ≤ 106.83? **TRUE**

2. *Node 1:* `Cl_NBO_Max_boltz` = -0.557. Is ≤ -0.556? **TRUE**

3. *Node 2:* `P_Pd_bond_occ_Avg_boltz` = 1.853. Is ≤ 1.86? **TRUE**

4. *Node 3:* `angle_R2P2Cback_boltz` = 104.16. Is ≤ 104.997? **TRUE**

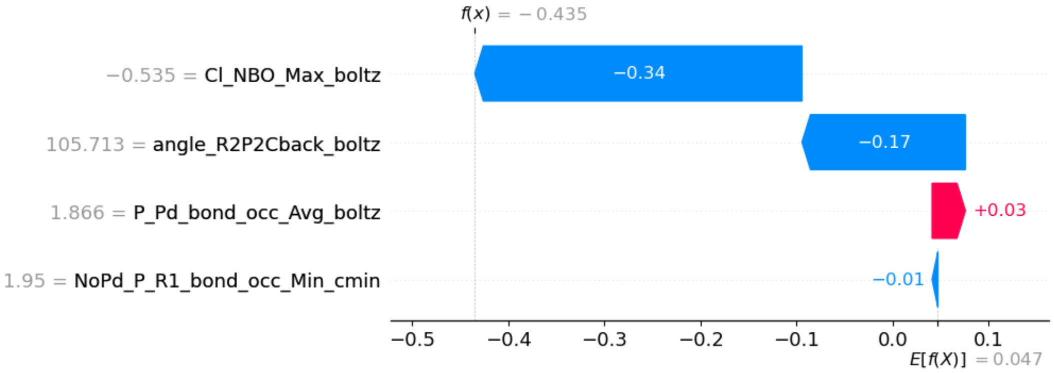

Figure S110: SHAP analysis from the decision tree regression model for the ligand with the lowest error (**pp000017**).

In addition to the above analysis, for all three features identified in the decision path, the decision thresholds were plotted along with the model error to see if any patterns could be identified. These were plotted in the context of the other test points for comparison. These plots are shown in Figure S111. Overall, no relationship was found between the point with the high model error and each of the tree features used in the decision tree.

6.2.4 Model overfitting checks

To check for decision tree regression model overfitting of the data, various tests were conducted. Firstly, cross-validation tests were performed. A leave-one-out cross-validation (LOOCV, shown in Figure S112) of the training set gave an average $R^2 = 0.45$, MAE = 0.31 kcal mol^{-1}, RMSE = 0.31 kcal mol^{-1}. Looking at Figure S112, most points which were mispredicted in the LOOCV analysis were those with low enantioselectivity, while those with high enantioselectivity (*i.e.*, more negative or more positive $\Delta\Delta G^{\ddagger}$) were predicted well. A five-fold cross-validation was also performed (with random shuffling of the data before splitting) which is shown in Figure S113. This gave an average $R^2 = 0.31$, MAE = 0.39 kcal mol^{-1} and RMSE = 0.51 kcal mol^{-1}. Similar to what was observed with the LOOCV analysis, the data points representing low enantioselectivity are not as well predicted compared to points with high enantioselectivity (this particularly notable with k-fold 2).

In addition to the cross-validation analyses described above, the effect of splitting the

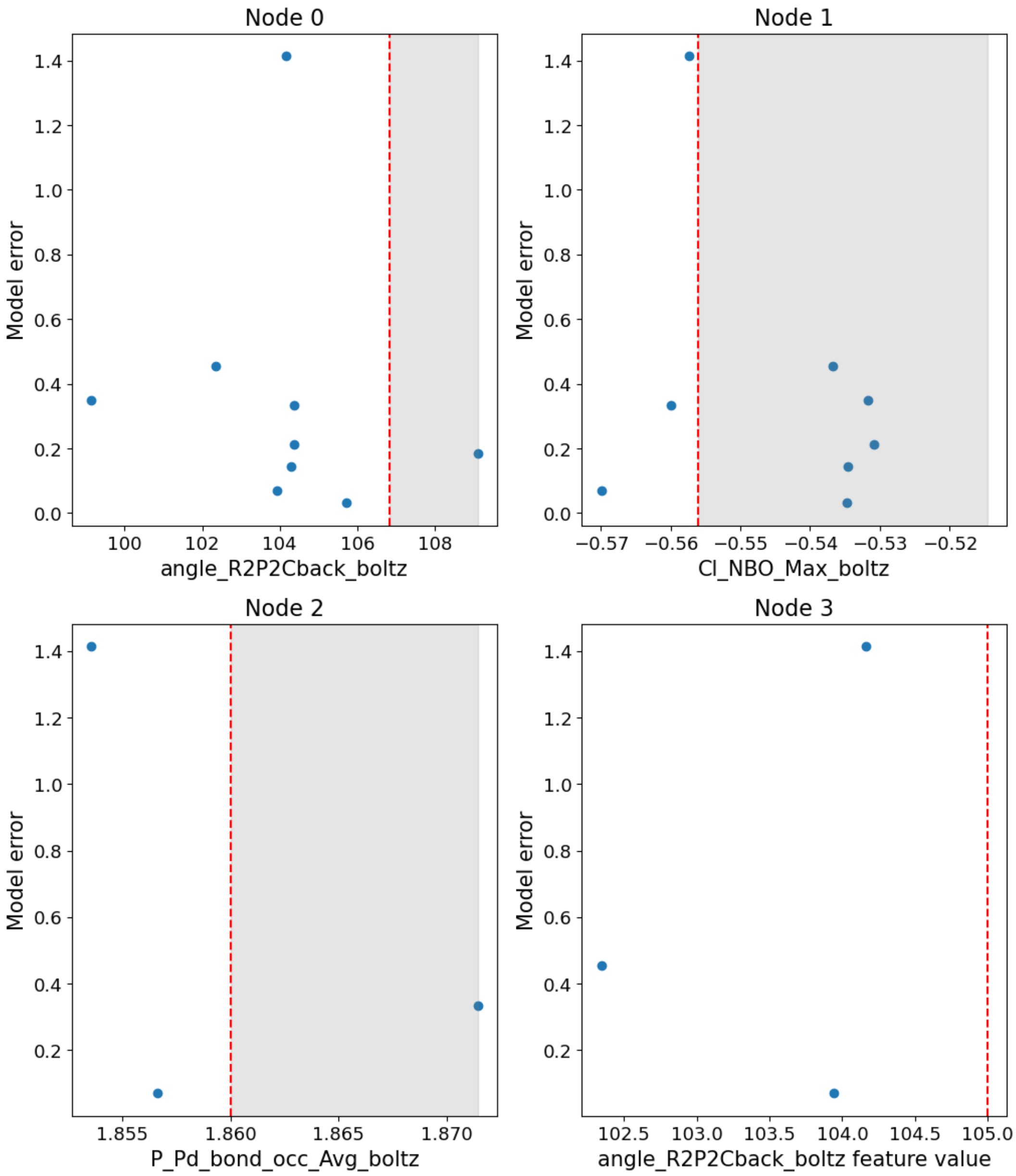

Figure S111: Decision thresholds used in the prediction of ligand **pp000055**.

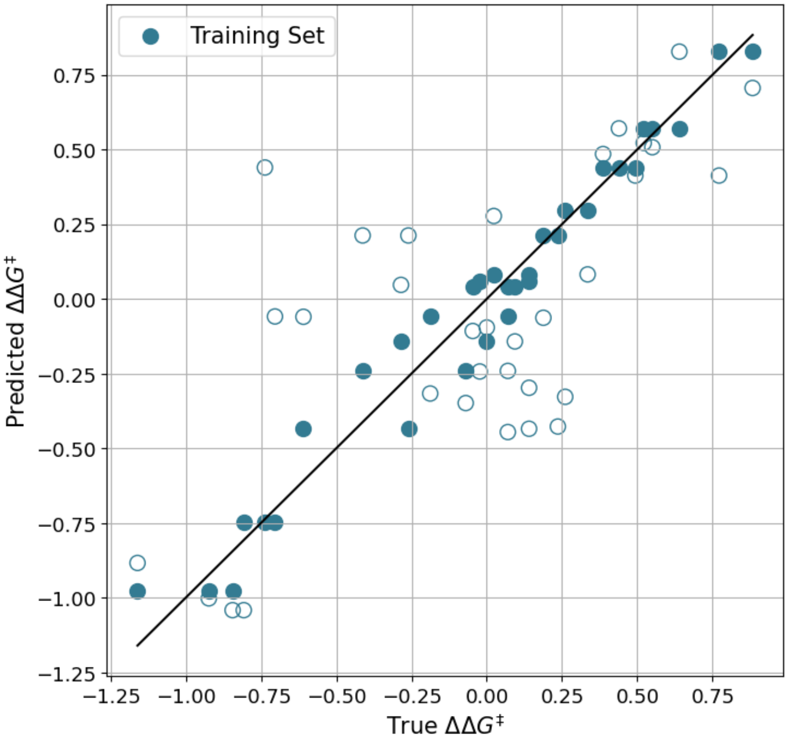

Figure S112: Leave-one-out cross-validation (LOOCV) analysis for the enantioselectivity decision tree regression model. Predicted LOOCV points are shown as hollow points on the graph.

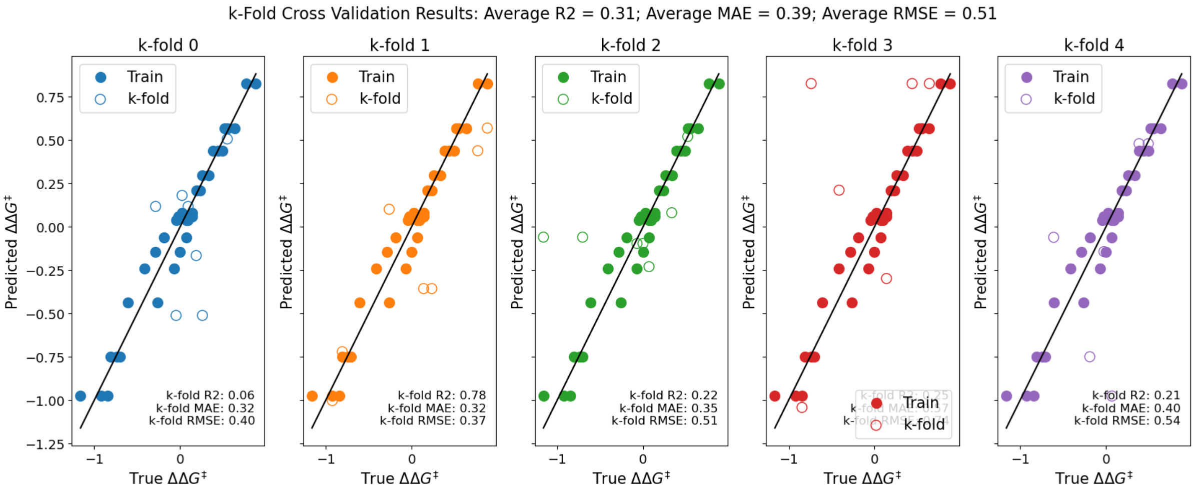

Figure S113: Five-fold cross-validation analysis for the enantioselectivity decision tree regression model. Predicted fold points for each of the five folds are shown as hollow points on the graph.

data into training and test sets on the statistical outcome was also investigated. Firstly, the random state used for the random split was changed. The data was split 100 times, each with a different randomly generated (between 1 and 1000) random state. For this metric we opted to use the test MAE and test mean squared error, MSE (the square of RMSE, and more sensitive to outliers), to examine the effect of model prediction of each train:test split. From this analysis, an average MAE = 0.38 ± 0.09 kcal mol^{-1} and MSE = 0.25 ± 0.13 was obtained. The results for each random state are given in Figure S114.

The ratio used for splitting the data into train:test bins (up to 50:50 split) was also investigated using `random_state=10`. This gave MAE = 0.43 ± 0.07 kcal mol^{-1} and MSE = 0.32 ± 0.08. The results for each split are given in Figure S115.

The results, in addition to the statistics presented in Section 6.2.3 are summarized in Table S8 for comparison of MAE. The performed checks in Entries 2-5 show similar statistics for the test set in the model in Section 6.2.3, which likely indicated a degree of overfitting of the data. However, the quality of the model was deemed sufficient for showing the difference between the conformer-weighted and lowest energy conformer features.

Table S8: Summary of overfitting checks outlined in Section 6.2.4. CV = cross-validation, LOOCV = leave-one-out cross-valdiation.

Entry	Overfit check	Train MAE / kcal mol^{-1}	CV or Test MAE / kcal mol^{-1}
**1**	**-**	**0.08**	**0.36**
2	LOOCV	-	0.31
3	5-fold CV	-	0.39
4	Random state	-	0.38
5	Split ratio	-	0.43

6.2.5 Effect of model hyperparameters

In addition to the overfitting tests performed in Section 6.2.4, the effect of the hyperparameter values was investigated as a function of model statistics. The following hyperparameters were changed:

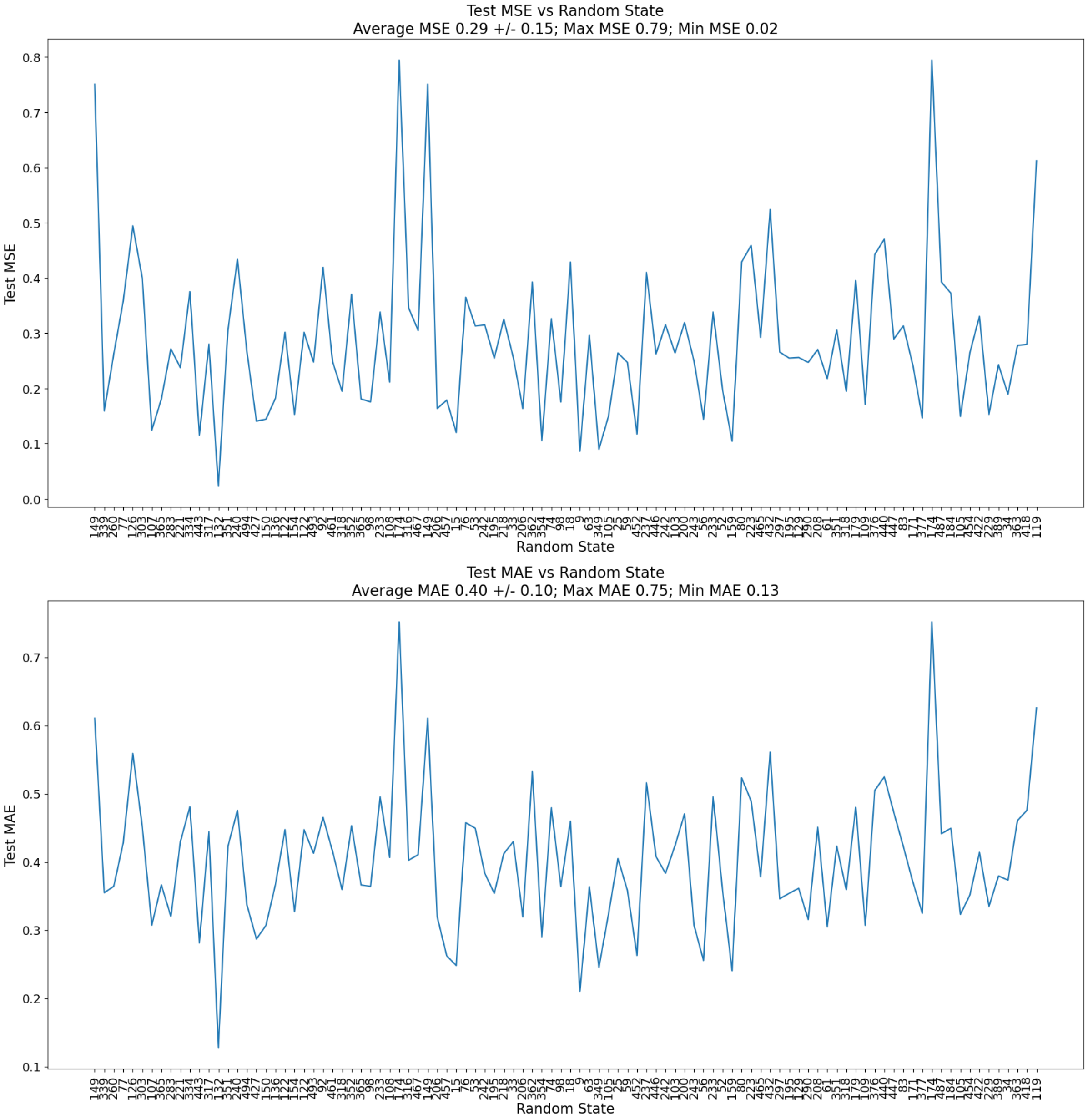

Figure S114: Effect of different random states used for the designation of training and set splits. Top shows the effect on the decision tree regression model's test MSE and the bottom shows the effect on the test MAE.

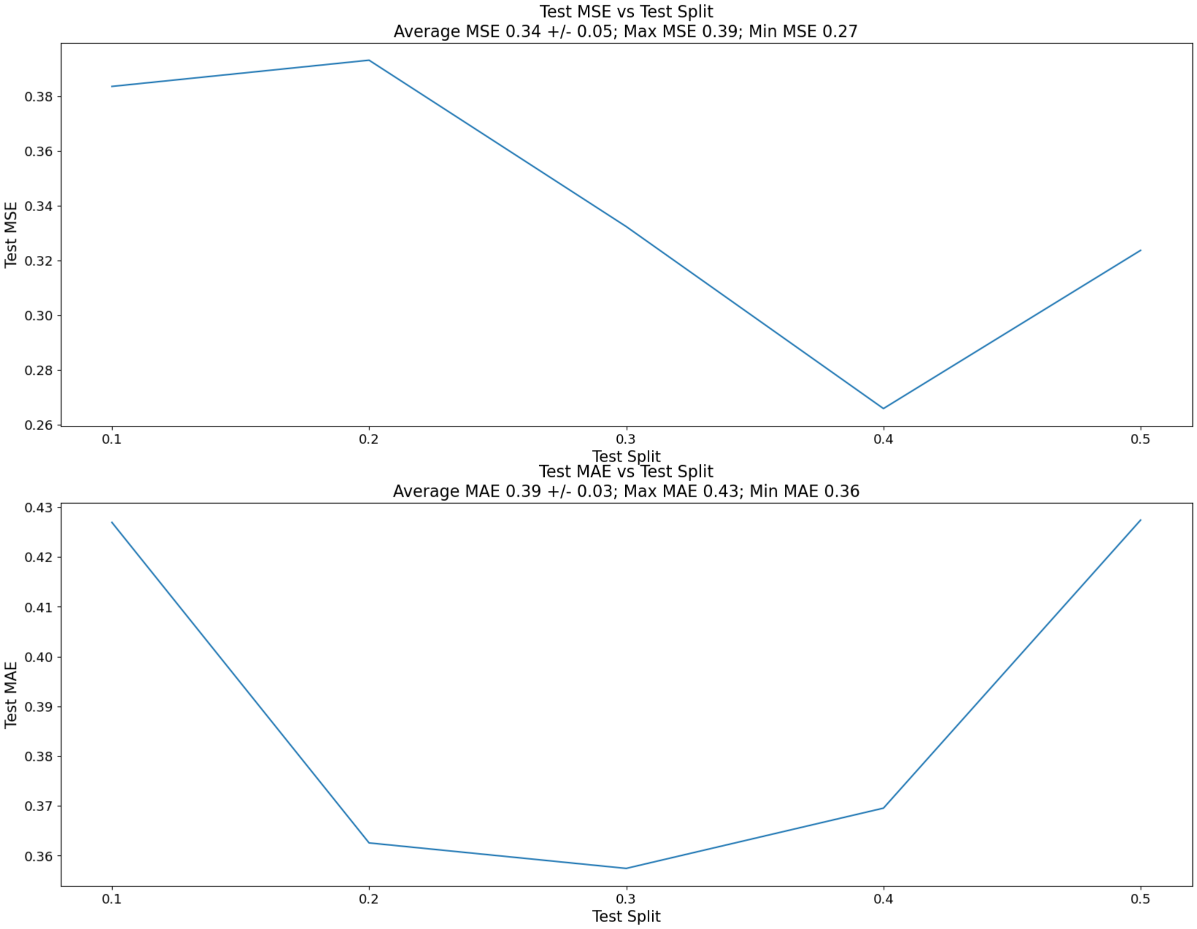

Figure S115: Effect of the split ratio used for the random train:test splitting. Top shows the effect on the decision tree regression model's test MSE and the bottom shows the effect on the test MAE.

- `max_depth`: 2-10 in increments of 1.
- `min_sample_leaf`: 1-33 in increments of 1.
- `min_samples_split`: 2-33 in increments of 1.

The results of changing these hyperparameters as a function of training and test MAE and MSE statistics are given in Figures S116 to S118.

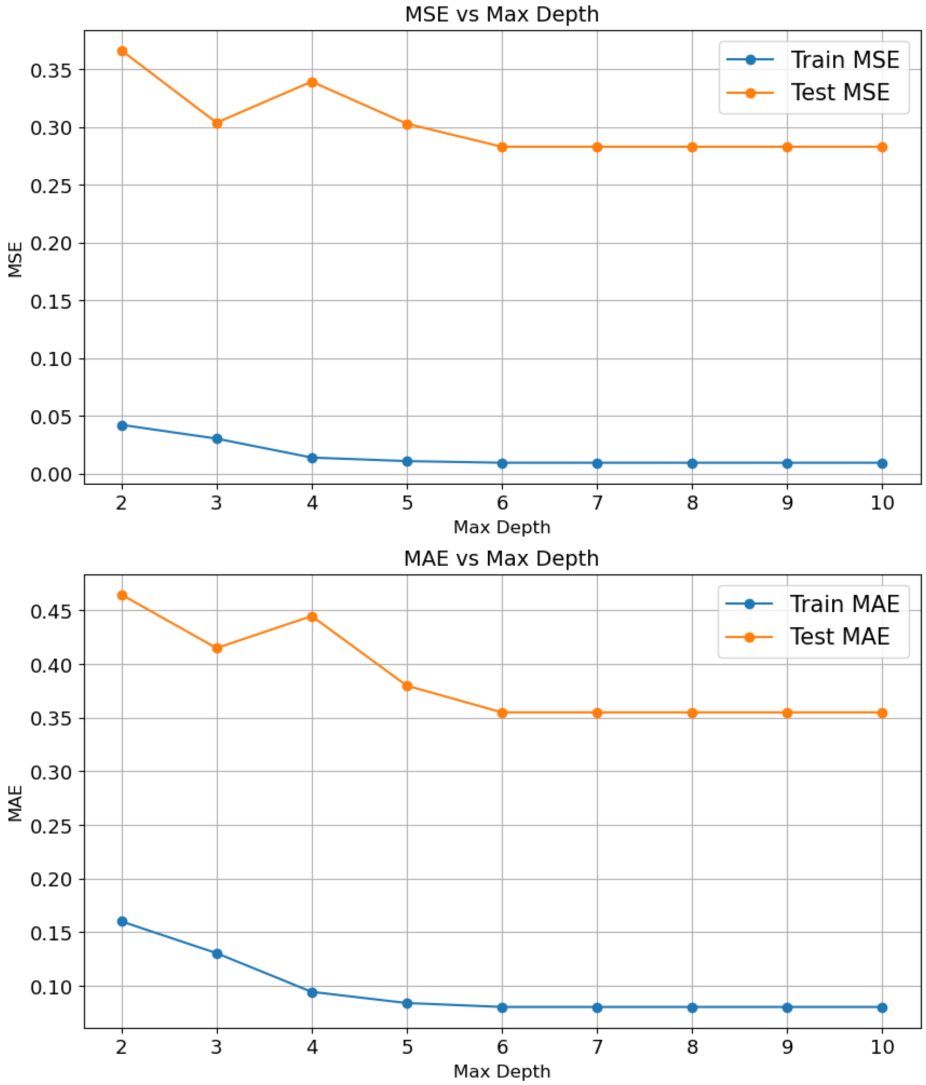

Figure S116: Effect of different hyperparameter values used for `max_depth`. Top shows the effect on the decision tree regression model's train and test MSE, The bottom shows the effect on the train and test MAE.

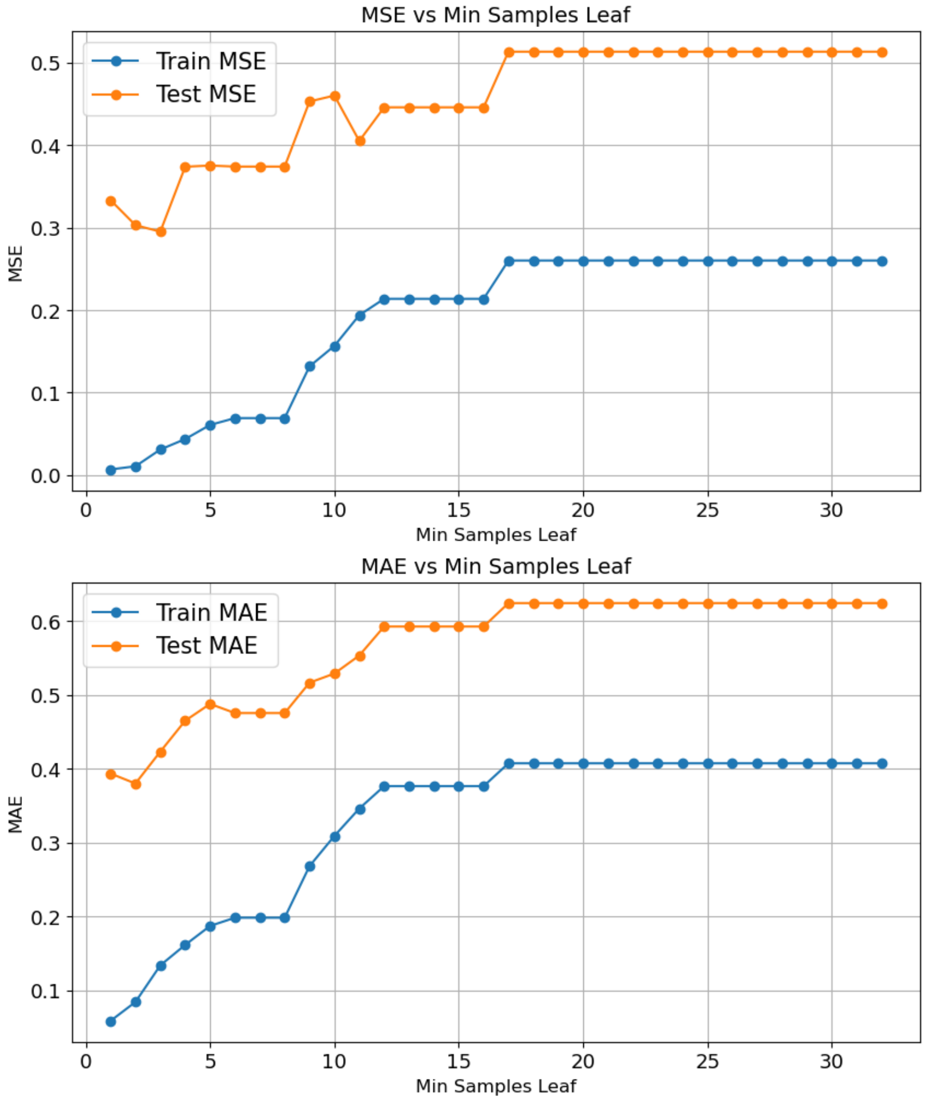

Figure S117: Effect of different hyperparameter values used for `min_samples_leaf`. Top shows the effect on the decision tree regression model's train and test MSE, The bottom shows the effect on the train and test MAE.

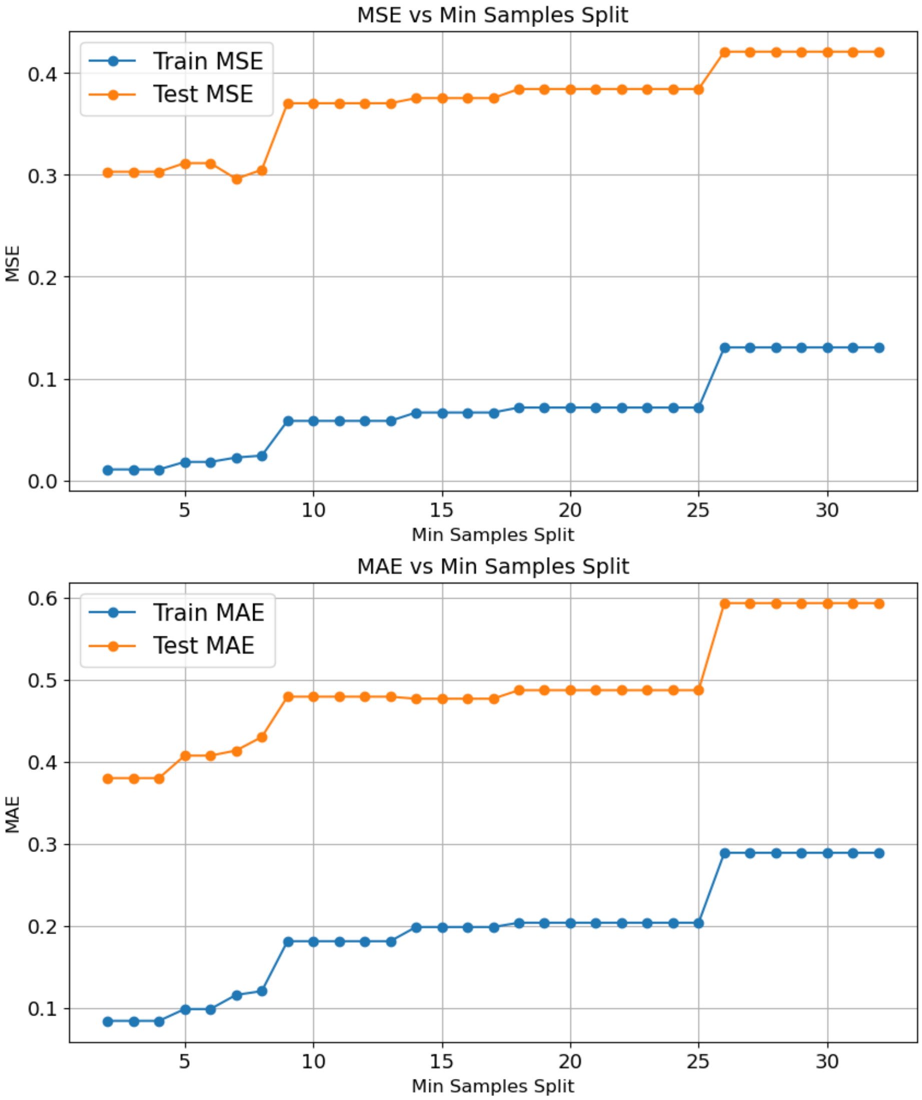

Figure S118: Effect of different hyperparameter values used for `min_samples_split`. Top shows the effect on the decision tree regression model's train and test MSE, The bottom shows the effect on the train and test MAE.

6.2.6 Analysis of feature conformer dependence

In addition to the conformational dependence of the ligands in Figure X of the manuscript, the equivalent information for all of the ligands is provided in Figures S119-S122.

6.2.7 Decision tree regression models with *only* lowest energy conformer features

The decision tree regression model outlined in Section 6.2.3 was regenerated using only the equivalent lowest energy conformer features and the same hyperparameters. This model, used towards determining the effect of conformer-weighted features is given in Figure S123. The training statistics for this model are: $R^2 = 0.94$; MAE = 0.096 kcal mol^{-1} and RMSE = 0.12 kcal mol^{-1}. The test statistics are: $R^2 = 0.14$; MAE = 0.53 kcal mol^{-1} and RMSE = 0.67 kcal mol^{-1}. With these same features, the model's hyperparameters were re-tuned as described in Section 6.2.2. This yielded an almost idential model to the one shown in Figure S123. The re-tuned model (Figure S124) had the following training set statistics: $R^2 = 0.94$; MAE = 0.096 kcal mol^{-1}; RMSE = 0.12 kcal mol^{-1}. The test set statistics were: $R^2 = 0.14$; MAE = 0.53 kcal mol^{-1} and RMSE = 0.67 kcal mol^{-1}.

Further to re-tuning the decision tree regression model hyperparameters, the model was re-run from scratch using the full set of lowest energy conformer features. This model is shown in Figure S125 and used the following five features:

- `NoPd_LP_P_s_Max`
- `P_Cback_bond_eng_range`
- `P1NBO`
- `NoPd_P_Cback_bond_eng_range`
- `NoPd_P_Cback_bond_eng_Min`

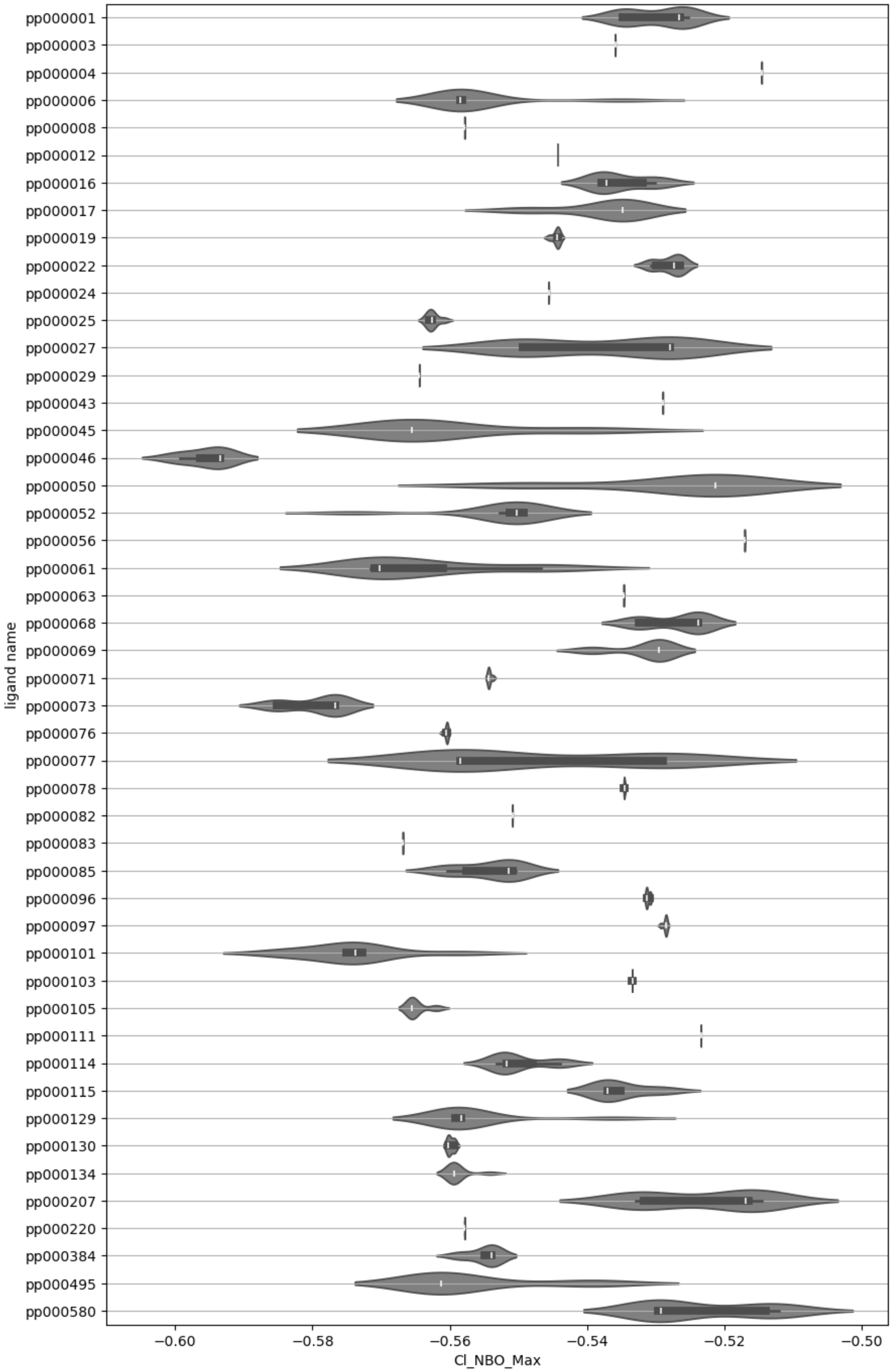

Figure S119: `Cl_NBO_Max` conformer dependence.

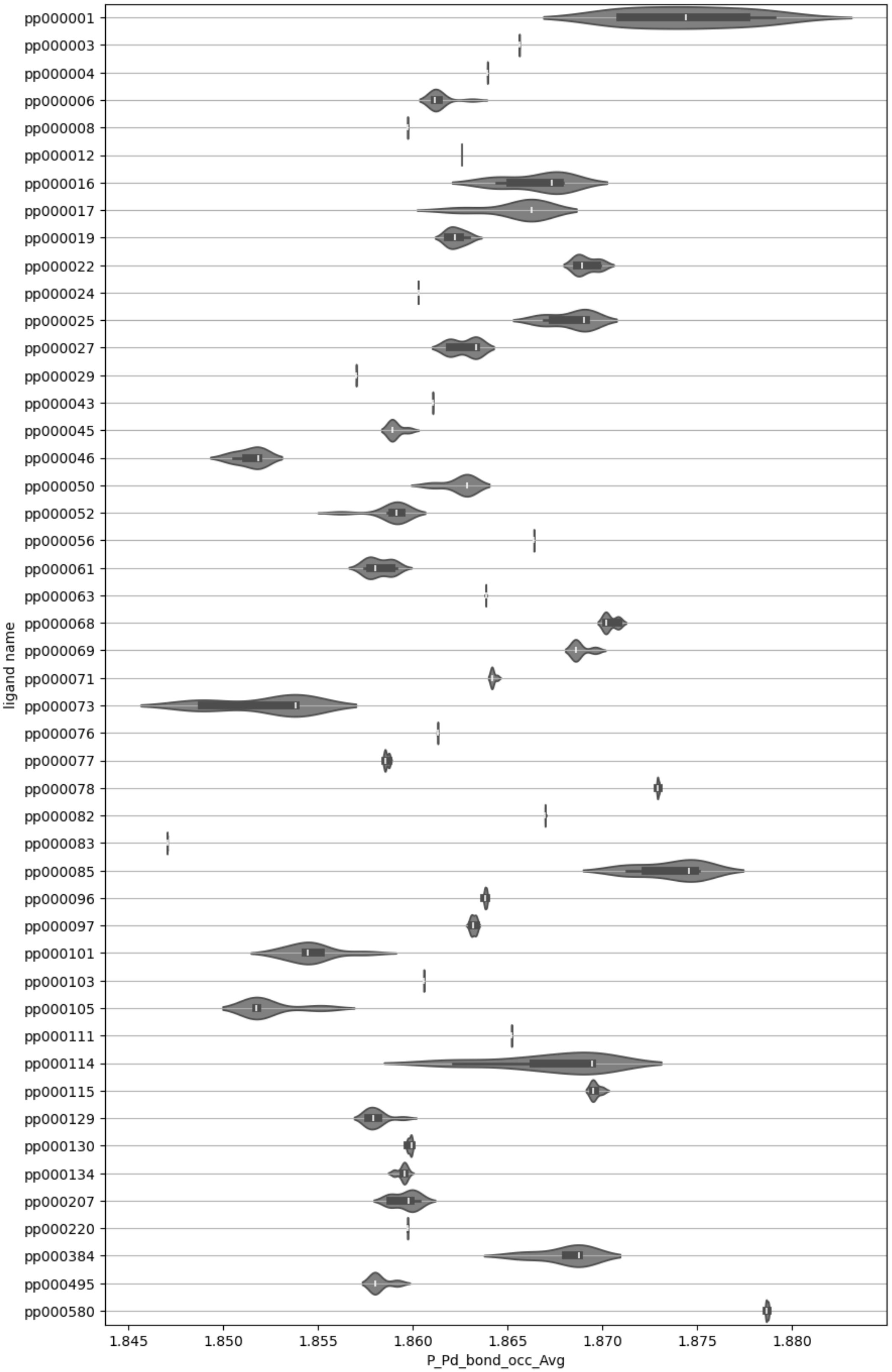

Figure S120: `P_Pd_bond_occ_Avg` conformer dependence.

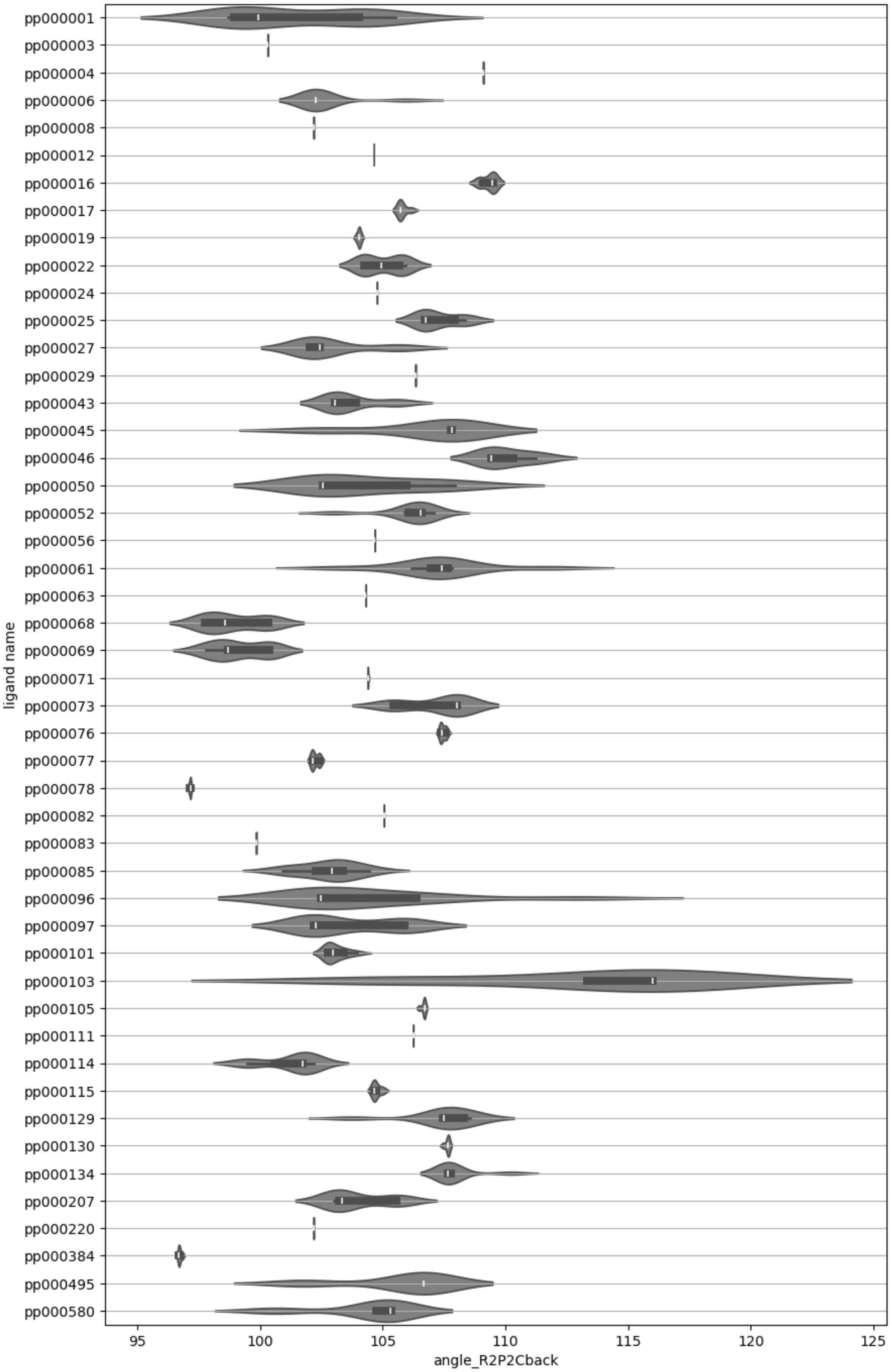

Figure S121: `angle_R2P2Cback` conformer dependence.

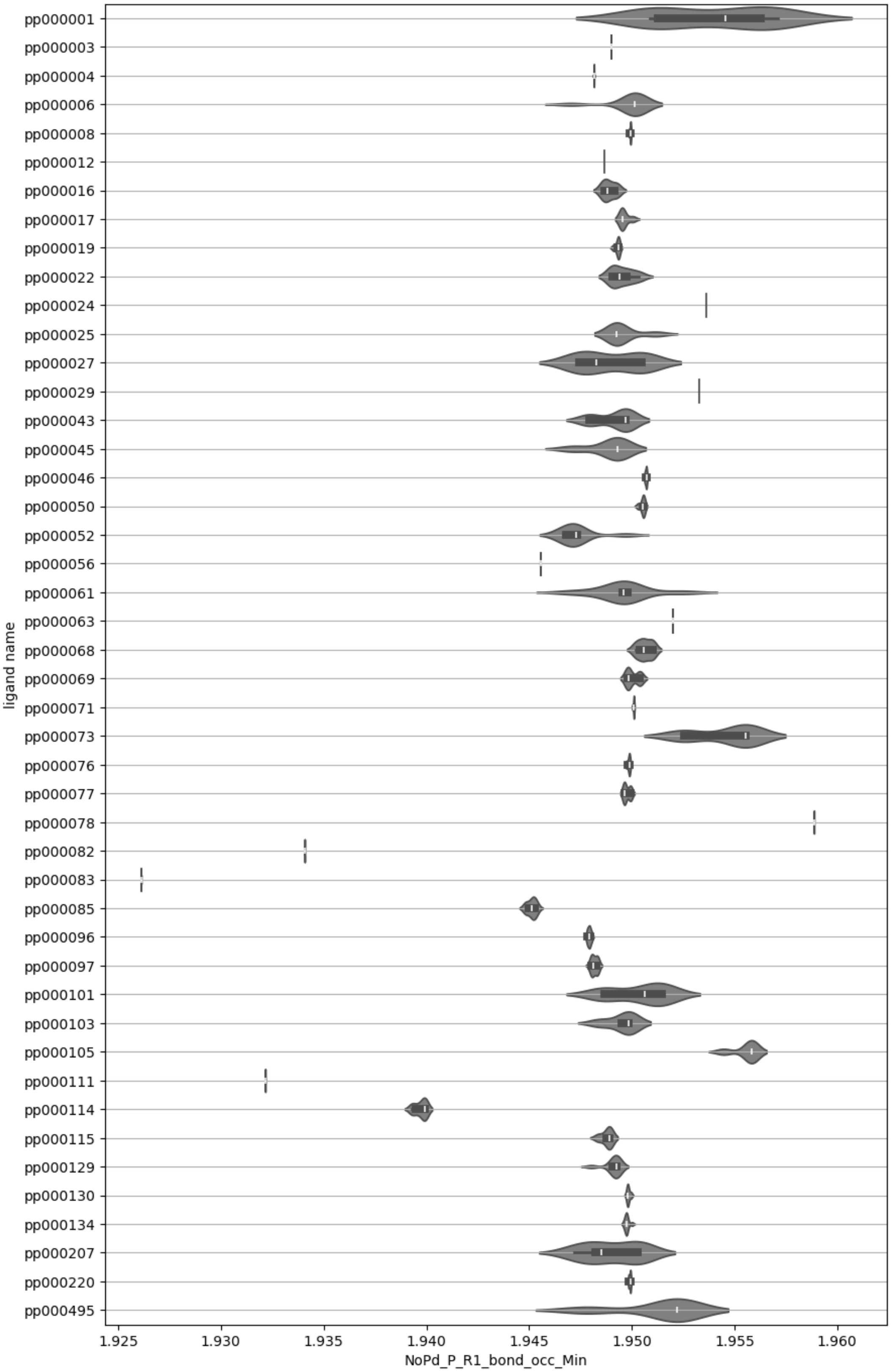

Figure S122: NoPd_P_R1_bond_occ_Min conformer dependence.

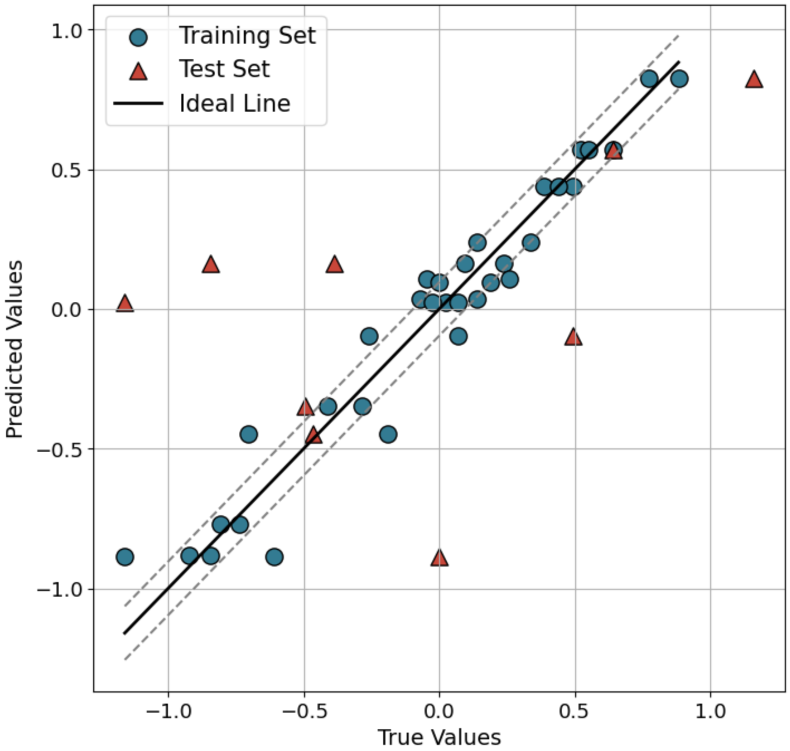

Figure S123: Sulfonimidamide aryl carbonylation enantioselectivity decision tree regression model using lowest energy conformer features.

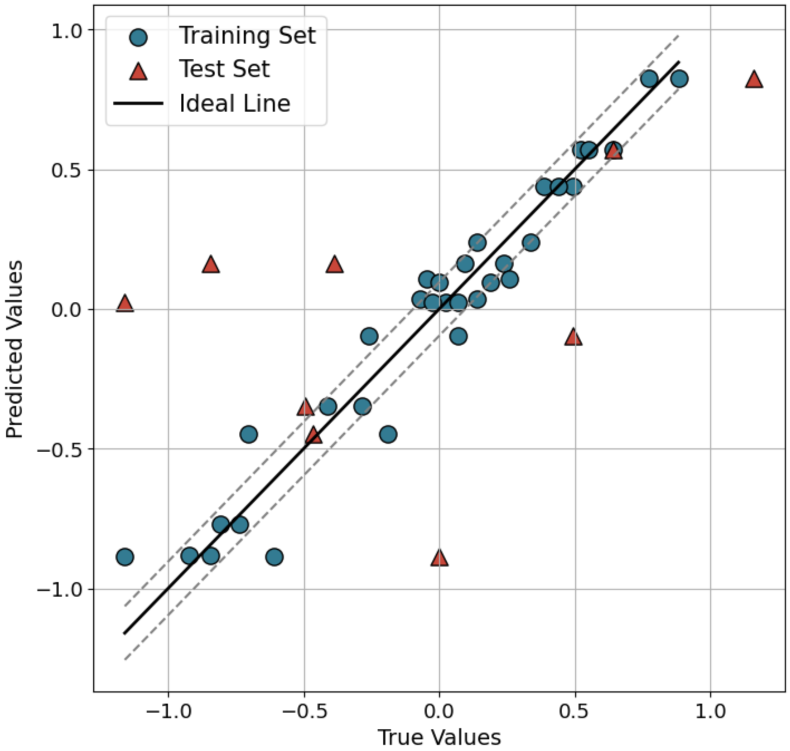

Figure S124: Sulfonimidamide aryl carbonylation enantioselectivity decision tree regression model using lowest energy conformer features and *re-tuned* hyperparameters.

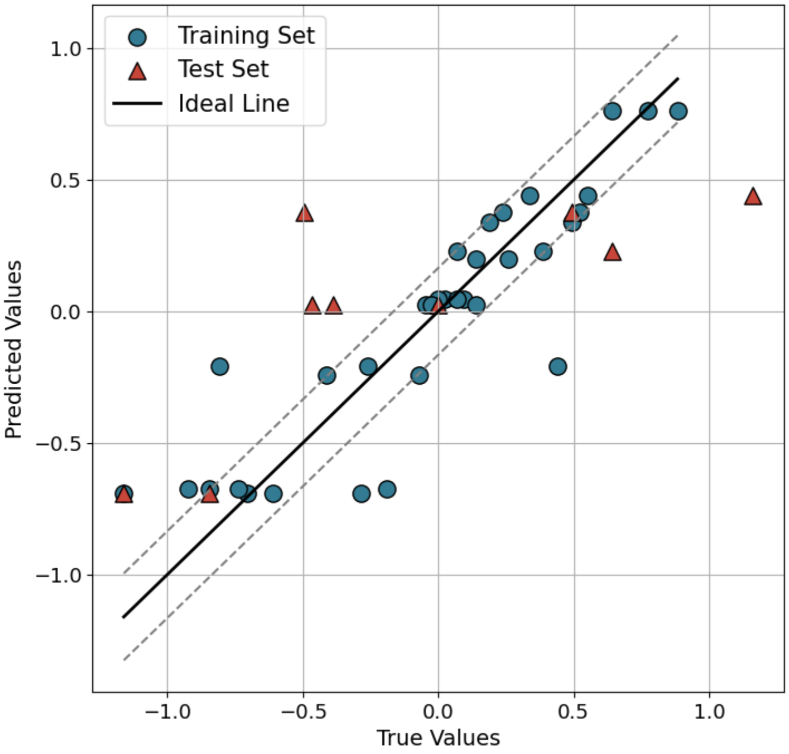

Figure S125: Sulfonimidamide aryl carbonylation enantioselectivity decision tree regression model using lowest energy conformer features. This model was re-generated from the full list of lowest energy conformer features.

Compared to the model generated from conformer-weighted features in Figure S105, this model has lower statistics for both the training and the test sets. The training set statistics are: $R^2 = 0.79$; MAE = 0.17 kcal mol^{-1}; RMSE = 0.23 kcal mol^{-1}. The test set statistics are: $R^2 = 0.54$; MAE = 0.41 kcal mol^{-1}; RMSE = 0.48 kcal mol^{-1}.

6.2.8 Alternative multivariate linear regression models

To demonstrate possible alternatives to the decision tree regression model for this dataset, two MLR models were generated with three and four components, respectively. These models were generated using a similar workflow to that outlined in Section 6.1.2 and utilised the same random train:test split ratio used in Section 6.2.2.

Three-component MLR model: The three-component MLR model for the sulfonamidamide aryl carbonylation is given in Figure S126 This model used the following equation:

$$\begin{aligned}\Delta\Delta G^{\ddagger} = \; & -0.025 \\ & -0.20 \cdot \texttt{angle\_R2PPd\_Min\_cmax} \\ & -0.33 \cdot \texttt{P1R2\_B1\_cavg} \\ & +0.37 \cdot \texttt{P2R1\_B5\_boltz}\end{aligned}$$

The training statistics for this model were: $R^2 = 0.60$; MAE = 0.23 kcal mol^{-1}; RMSE = 0.32 kcal mol^{-1}. The test statistics for this model were: $R^2 - 0.20$; MAE = 0.59 kcal mol^{-1}; RMSE = 0.78 kcal mol^{-1}.

Four-component MLR model: The four-component model for the enantioselectivity $\Delta\Delta G^{\ddagger}$ generated is shown in Figure S127. This model used the following equation:

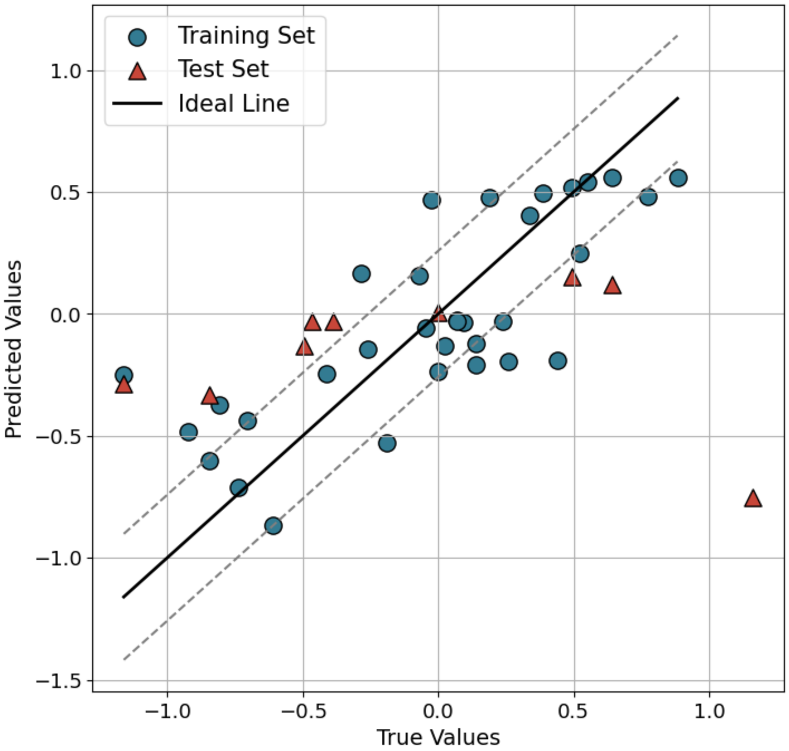

Figure S126: Sulfonimidamide aryl carbonylation enantioselectivity three-component multivariate linear regression model.

$$\Delta\Delta G^{\ddagger} = -0.025$$
$$+0.20 \cdot \texttt{Cl\_Pd\_distance\_range\_crange}$$
$$-0.29 \cdot \texttt{S\_avg\_2.5\_Ang\_crange}$$
$$+0.31 \cdot \texttt{P1R2\_B1\_cavg}$$
$$+0.34 \cdot \texttt{P2R1\_B5\_boltz}$$

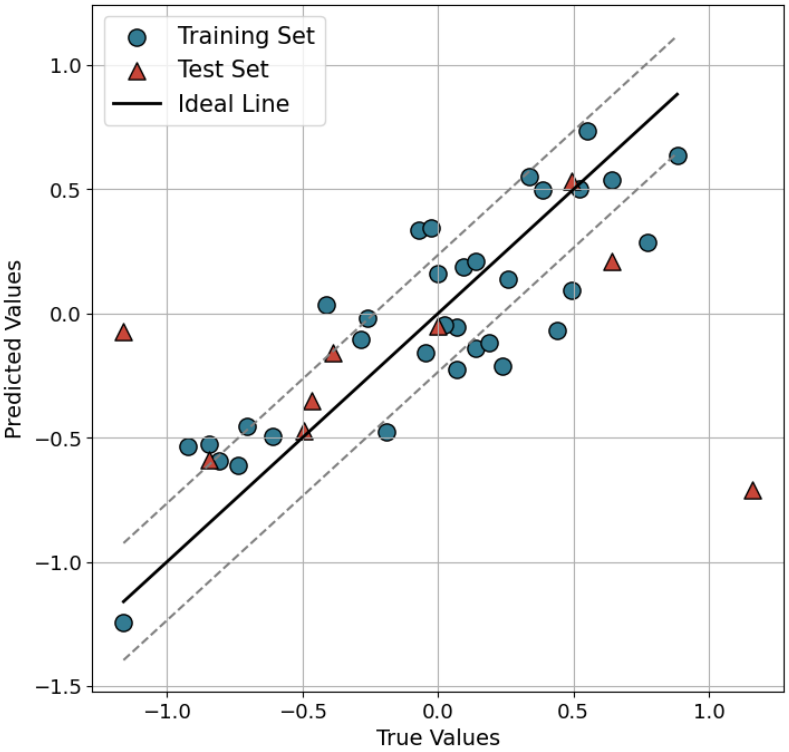

Figure S127: Sulfonimidamide aryl carbonylation enantioselectivity four-component multivariate linear regression model.

The training statistics for this model were: $R^2 = 0.72$; MAE = 0.24 kcal mol^{-1}; RMSE = 0.27 kcal mol^{-1}. The test statistics for this model were: $R^2 = -0.082$; MAE = 0.46 kcal mol^{-1}; RMSE = 0.75 kcal mol^{-1}.

6.2.9 Model comparison and discussion

For all the models generated in Sections 6.2.2-6.2.8, the test set statistics are summarised in Table S9 for comparison. In general the decision tree regression model using conformer-weighted bisphosphine ligand features (entry 1, Figure S105) gave the best test statistics. This showed that using conformer-weighted features allows better predictive power in terms of enantioselectivity. Additionally, the decision tree classification models slightly outperformed the multivariate linear regression models showing that the use of a ”non-linear” modeling technique was required for the complex nature of the data set.

Table S9: Sulfonimidamide carbonylation enantioselectivity model comparison from Sections 6.2.2-6.2.8. DTR = decision tree regression; MLR = multivariate linear regression.

Model	Model type	Figure	Test R^2	Test MAE / kcal mol^{-1}	Test RMSE / kcal mol^{-1}
**1**	**DTR**	S105	**0.45**	**0.08**	**0.10**
2	DTR	S123	0.14	0.53	0.67
3	DTR	S124	0.14	0.53	0.67
4	DTR	S125	0.54	0.41	0.48
5	MLR	S126	-0.20	0.59	0.78
6	MLR	S127	-0.082	0.46	0.75

References

(1) Dotson, J. J.; van Dijk, L.; Timmerman, J. C.; Grosslight, S.; Walroth, R. C.; Gosselin, F.; Püntener, K.; Mack, K. A.; Sigman, M. S. Data-Driven Multi-Objective Optimization Tactics for Catalytic Asymmetric Reactions Using Bisphosphine Ligands. *J. Am. Chem. Soc* **2023**, *145*, 110–121, doi: 10.1021/jacs.2c08513.

(2) Pracht, P.; Bohle, F.; Grimme, S. Automated exploration of the low-energy chemical space with fast quantum chemical methods. *Phys. Chem. Chem. Phys.* **2020**, *22*, 7169–7192.

(3) Bannwarth, C.; Caldeweyher, E.; Ehlert, S.; Hansen, A.; Pracht, P.; Seibert, J.; Spicher, S.; Grimme, S. Extended tight-binding quantum chemistry methods. *WIREs Computational Molecular Science* **2021**, *11*, e1493.

(4) Spicher, S.; Grimme, S. Robust Atomistic Modeling of Materials, Organometallic, and Biochemical Systems. *Angew. Chem. Int. Ed.* **2020**, *59*, 15665–15673.

(5) Bannwarth, C.; Ehlert, S.; Grimme, S. GFN2-xTB—An Accurate and Broadly Parametrized Self-Consistent Tight-Binding Quantum Chemical Method with Multipole Electrostatics and Density-Dependent Dispersion Contributions. *J. Chem. Theory Comput.* **2019**, *15*, 1652–1671, doi: 10.1021/acs.jctc.8b01176.

(6) Frisch, M. J. et al. Gaussian 16 Revision C.01. 2016; Gaussian Inc. Wallingford CT.

(7) Perdew, J. P.; Burke, K.; Ernzerhof, M. Generalized Gradient Approximation Made Simple. *Phys. Rev. Lett.* **1996**, *77*, 3865–3868.

(8) Perdew, J. P.; Burke, K.; Ernzerhof, M. Generalized Gradient Approximation Made Simple [Phys. Rev. Lett. 77, 3865 (1996)]. *Phys. Rev. Lett.* **1997**, *78*, 1396–1396.

(9) Grimme, S.; Antony, J.; Ehrlich, S.; Krieg, H. A consistent and accurate ab initio parametrization of density functional dispersion correction (DFT-D) for the 94 elements H-Pu. *J. Chem. Phys.* **2010**, *132*, 154104.

(10) Grimme, S.; Ehrlich, S.; Goerigk, L. Effect of the damping function in dispersion corrected density functional theory. *J. Comput. Chem.* **2011**, *32*, 1456–1465.

(11) Weigend, F.; Ahlrichs, R. Balanced basis sets of split valence, triple zeta valence and quadruple zeta valence quality for H to Rn: Design and assessment of accuracy. *Phys. Chem. Chem. Phys.* **2005**, *7*, 3297–3305.

(12) Andrae, D.; Häußermann, U.; Dolg, M.; Stoll, H.; Preuß, H. Energy-adjustedab initio pseudopotentials for the second and third row transition elements. *Theoretica chimica acta* **1990**, *77*, 123–141.

(13) Peterson, K. A.; Figgen, D.; Goll, E.; Stoll, H.; Dolg, M. Systematically convergent basis sets with relativistic pseudopotentials. II. Small-core pseudopotentials and correlation consistent basis sets for the post-d group 16–18 elements. *Journal of Chem. Phys.* **2003**, *119*, 11113–11123.

(14) Pritchard, B. P.; Altarawy, D.; Didier, B.; Gibson, T. D.; Windus, T. L. New Basis Set Exchange: An Open, Up-to-Date Resource for the Molecular Sciences Community. *J. Chem. Inf. Model.* **2019**, *59*, 4814–4820, doi: 10.1021/acs.jcim.9b00725.

(15) Adamo, C.; Barone, V. Toward reliable density functional methods without adjustable parameters: The PBE0 model. *J. Chem. Phys.* **1999**, *110*, 6158–6170.

(16) Ernzerhof, M.; Scuseria, G. E. Assessment of the Perdew–Burke–Ernzerhof exchange-correlation functional. *J. Chem. Phys.* **1999**, *110*, 5029–5036.

(17) Glendening, E. D and Badenhoop, J. K. and Reed, A. E. and Carpenter, J. E. and Bohmann, J. A. and Morales, C. M. and Karafiloglou, P. and Landis, C. R. and Weinhold, F. NBO 7.0. 2018; Theoretical Chemistry Institute, University of Wisconsin, Madison.

(18) MORFEUS: Molecular features for machine learning. https://github.com/digital-chemistry-laboratory/morfeus.

(19) Bilbrey, J. A.; Kazez, A. H.; Locklin, J.; Allen, W. D. Exact Ligand Solid Angles. *J. Chem. Theory Comput.* **2013**, *9*, 5734–5744, doi: 10.1021/ct400426e.

(20) Falivene, L.; Credendino, R.; Poater, A.; Petta, A.; Serra, L.; Oliva, R.; Scarano, V.; Cavallo, L. SambVca 2. A Web Tool for Analyzing Catalytic Pockets with Topographic Steric Maps. *Organometallics* **2016**, *35*, 2286–2293, doi: 10.1021/acs.organomet.6b00371.

(21) Shrake, A.; Rupley, J. Environment and exposure to solvent of protein atoms. Lysozyme and insulin. *Journal of Molecular Biology* **1973**, *79*, 351–371.

(22) Eisenhaber, F.; Lijnzaad, P.; Argos, P.; Sander, C.; Scharf, M. The double cubic lattice method: Efficient approaches to numerical integration of surface area and volume and to dot surface contouring of molecular assemblies. *J. Comput. Chem.* **1995**, *16*, 273–284.

(23) Wolinski, K.; Hinton, J. F.; Pulay, P. Efficient implementation of the gauge-independent atomic orbital method for NMR chemical shift calculations. *J. Am. Chem. Soc.* **1990**, *112*, 8251–8260.

(24) Cheeseman, J. R.; Trucks, G. W.; Keith, T. A.; Frisch, M. J. A comparison of models for calculating nuclear magnetic resonance shielding tensors. *J. Chem. Phys.* **1996**, *104*, 5497–5509.

(25) Pedregosa, F. et al. Scikit-learn: Machine Learning in Python. *Journal of Machine Learning Research* **2011**, *12*, 2825–2830.

(26) Buitinck, L.; Louppe, G.; Blondel, M.; Pedregosa, F.; Mueller, A.; Grisel, O.; Niculae, V.; Prettenhofer, P.; Gramfort, A.; Grobler, J.; Layton, R.; VanderPlas, J.; Joly, A.; Holt, B.; Varoquaux, G. API design for machine learning software: experiences from the scikit-learn project. ECML PKDD Workshop: Languages for Data Mining and Machine Learning. 2013; pp 108–122.

(27) Richard J. Gowers; Max Linke; Jonathan Barnoud; Tyler J. E. Reddy; Manuel N. Melo; Sean L. Seyler; Jan Domański; David L. Dotson; Sébastien Buchoux; Ian M. Kenney; Oliver Beckstein MDAnalysis: A Python Package for the Rapid Analysis of Molecular Dynamics Simulations. Proceedings of the 15th Python in Science Conference. 2016; pp 98 – 105.

(28) Michaud-Agrawal, N.; Denning, E. J.; Woolf, T. B.; Beckstein, O. MDAnalysis: A toolkit for the analysis of molecular dynamics simulations. *J. Comput. Chem.* **2011**, *32*, 2319–2327.

(29) Waskom, M. L. seaborn: statistical data visualization. *Journal of Open Source Software* **2021**, *6*, 3021.

(30) van Dijk, L.; Haas, B. C.; Lim, N.-K.; Clagg, K.; Dotson, J. J.; Treacy, S. M.; Piechowicz, K. A.; Roytman, V. A.; Zhang, H.; Toste, F. D.; Miller, S. J.; Gosselin, F.; Sigman, M. S. Data Science-Enabled Palladium-Catalyzed Enantioselective Aryl-Carbonylation of Sulfonimidamides. *J. Am. Chem. Soc.* **2023**, *145*, 20959–20967, doi: 10.1021/jacs.3c06674.

(31) Virtanen, P. et al. SciPy 1.0: Fundamental Algorithms for Scientific Computing in Python. *Nature Methods* **2020**, *17*, 261–272.

(32) Veerasamy, R.; Rajak, H.; Jain, A.; Sivadasan, S.; Christapher, P. V.; Agrawal, R. Validation of QSAR Models - Strategies and Importance. *Int J Drug Design and Discov* **2011**, *2*, 511–519.

(33) Haas, B. C.; Hardy, M. A.; Sowndarya S. V., S.; Adams, K.; Coley, C. W.; Paton, R. S.; Sigman, M. S. Rapid prediction of conformationally-dependent DFT-level descriptors using graph neural networks for carboxylic acids and alkyl amines. *Digital Discovery* **2025**, *4*, 222–233.

(34) Lundberg, S. M.; Erion, G.; Chen, H.; DeGrave, A.; Prutkin, J. M.; Nair, B.; Katz, R.; Himmelfarb, J.; Bansal, N.; Lee, S.-I. From local explanations to global understanding with explainable AI for trees. *Nature Machine Intelligence* **2020**, *2*, 56–67.
